# Spatial and temporal analysis of the risks posed by metal contamination in coastal and marine sediments of Bahrain

**DOI:** 10.1007/s10661-021-09722-7

**Published:** 2022-01-06

**Authors:** E. E. M. Nicolaus, D. L. Maxwell, A. S. Khamis, K. H. Abdulla, R. P. Harrod, M. J. Devlin, B. P. Lyons

**Affiliations:** 1 Cefas, Lowestoft Laboratory, Pakefield Road, Lowestoft, NR33 0HT UK; 2 Supreme Council for Environment, P.O. Box 18233, Manama, Bahrain; 3 Centre for Environment, Fisheries and Aquaculture Science (Cefas), Weymouth laboratory, Barrack Road, Weymouth, DT4 8UB Dorset UK; 4 British Embassy at the State of Kuwait, P.O. Box 2, 13001 Safat, Kuwait

**Keywords:** Metals, Marine sediments, Risk characterisation ratio, Environmental quality standards (OSPAR BAC, ERL, ERM, ISQG, PEL), Status and trend assessment, Bahrain

## Abstract

Nine metals including Al, Cd, Cr, Cu, Fe, Mn, Ni, Pb and Zn were analysed from sediment samples collected from 29 stations since 2007 from Bahraini waters. Within this study, it was investigated whether concentrations of these determinants are at concentrations above internationally established Assessment Criteria (AC). The majority of sites were considered not to pose a toxicological risk in terms of metal contamination. Where breaches occurred, they were mainly from historic samples related to Cr, Cu and Ni contamination. A trend assessment revealed that out of 59 significant trends, 36 were downwards and 23 upwards, indicating that some determinants like Al, Zn and Ni are improving strongly across some sites, whilst areas associated with industrial activity still see some increasing trends for Al, Cd, Pb and Zn.

**Supplementary information:**

The online version contains supplementary material available at 10.1007/s10661-021-09722-7.

## Introduction

**Fig. 1:**
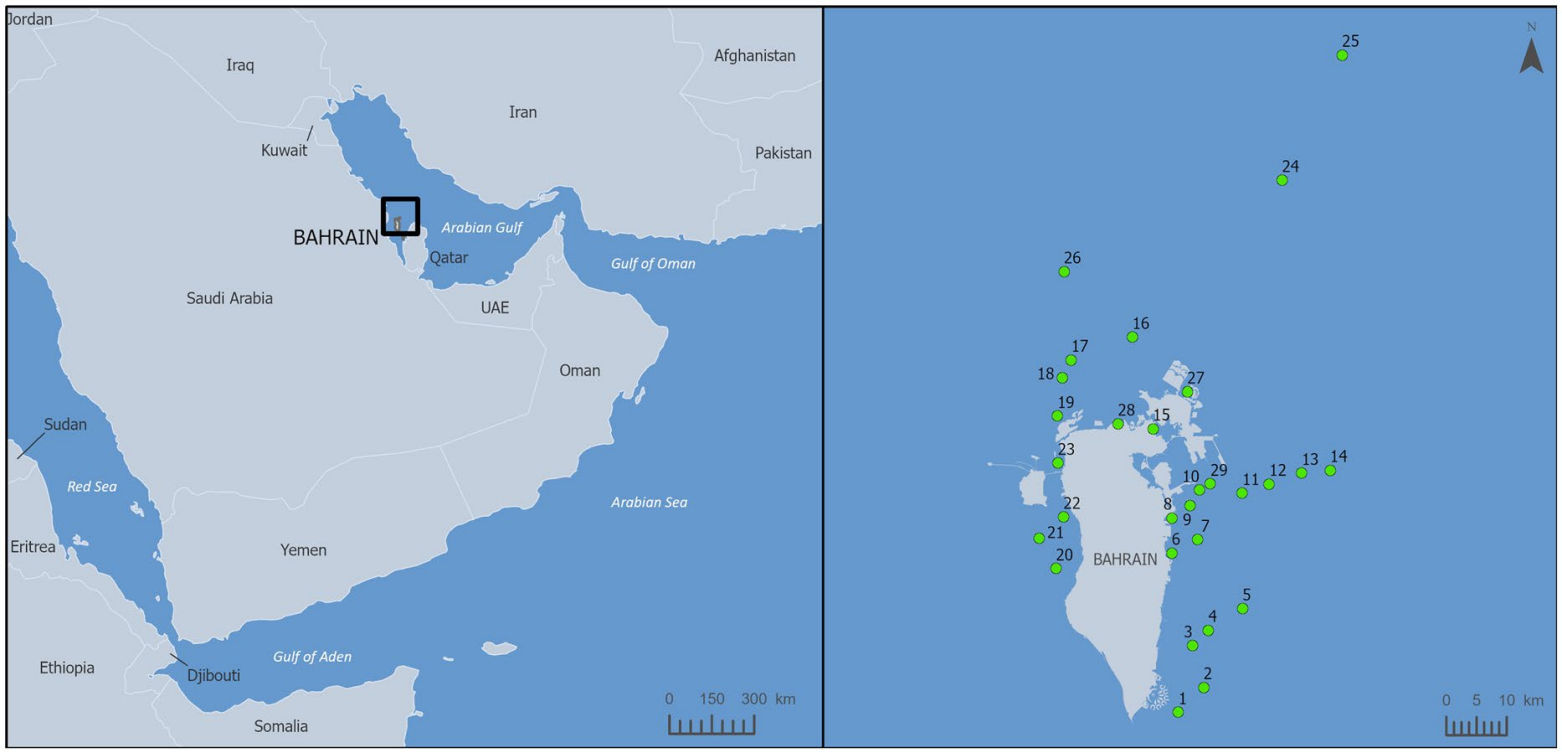
A pool of 29 sediment sampling stations around Bahrain

The Kingdom of Bahrain, located in the south-western Arabian Gulf (Fig. 1), has undergone major economic, social and industrial development since the 1970s (Elghonaimy & Mohammed, 2019; Naser, 2015). Bahrain’s population increased tenfold between 1959 and 2018 (Information & eGovernment Authority, 2021). This rapid expansion of Bahrain’s urban and industrial sectors has mainly occurred around its coasts, making it one of the most densely populated countries in the world (Zainal et al., 2009; Information and eGovernment Authority, 2021). To meet the land requirements for this population and economic growth Bahrain has now developed more than 80% of its coastline and reclaimed an additional 110 km^2^ of its coastal waters (Naser, 2015). However, this economic and social development has put ecologically important marine ecosystems, including sea grass beds, coral reefs and mangroves under pressure (Abido et al., 2011; Burt et al., 2013; Naser, 2015).

This coastal development, which includes petrochemical, power generation and desalination industries, discharges significant volumes of effluent into Bahrain’s marine waters. Consequently, a variety of contaminants have been reported in biota, sediment and water samples, including petroleum hydrocarbons, organohalogens, metals and nutrients from sewage treatment plants (Bersuder et al., 2020; de Mora et al., 2010; Naser, 2011; Tolosa et al., 2005). These contaminant inputs pose a risk to Bahrain’s marine habitats, which serve as a feeding and nursery ground for many ecologically and economically important species like dugongs and green turtles (Abdulqader, 1999; Zainal et al., 2007; Moore & Peirce, 2013; Arab Regional Centre for World Heritage & Supreme Council for Environment, 2017).

The Public Commission for the Protection of Marine Resources, Environment and Wildlife (PMEW) of Bahrain initiated a marine monitoring program in 2007 by investigating the contaminant loads in sediments to see if they pose any threat to the marine environment. This was continued from 2012 onwards by the Supreme Council for the Environment (SCE) (Abdulla & Naser, 2021). Monitoring focused on priority metal contaminants including those such as cadmium (Cd) and lead (Pb) that are known to have effects to marine organisms. Cd for example tends to accumulate in the food chain as it is being actively taken up by algae and animals in parallel with phosphate (Nicolaus et al., 2018). Whilst Pb does not bioaccumulate, it still cause adverse effects to marine life and has been added to the OSPAR priority hazardous substance list (Nicolaus et al., 2018).

In this paper, the metal data collected between 2007 and 2020 from up to 29 sediment stations have been analysed for status and trend assessment by comparing the measured values to internationally derived Assessment Criteria (AC).

## Materials and methods

Sediment monitoring started in 2007 at 20 stations and increased to a network of 29 stations by 2020 (Fig. 1; Table 1). Not every station was sampled every year, but a core of 10 stations were sampled each year between 2007 and 2020. As depicted in Fig. 1, the stations are located around the coastline apart from the south western side, where no stations are sited. The main historic reason for this was that the south west was seen not within an industrial area and ecologically less significant compared to the eastern and northern coasts which contain important habitats such as coral and sea grass beds, along with the concentration of coastal development (both urban and industrial). Overall, the monitored stations include important fishing grounds, oyster beds, marine protected areas and anthropogenically impacted areas.

**Table 1:** A pool of 29 sediment sampling stations around Bahrain and their coordinates

Station name	Station number	Latitude (decimal degrees)	Longitude (decimal degrees)
Noon	1	25.8137	50.64265
Mashtan	2	25.8512	50.68428
Jabari	3	25.91343	50.66568
Tugailib	4	25.93642	50.69192
Ghumais	5	25.96878	50.74847
Askar	6	26.05145	50.63127
Msoor	7	26.07212	50.67415
Refinery Area	8	26.10388	50.63132
Gaha	9	26.12193	50.6612
Suhain	10	26.14552	50.67635
Duwaimil	11	26.14113	50.74765
Gazara	12	26.15477	50.7923
Dam	13	26.17135	50.84602
Jaradah	14	26.17547	50.89312
Jetty 1	15	26.23675	50.59925
Al-Jarim	16	26.37308	50.56458
Khorfasht	17	26.33843	50.46312
Murwada	18	26.31138	50.44875
Bartafi	19	26.25533	50.44062
Gasar	20	26.02772	50.43943
Umm Al-Na’asan	21	26.07283	50.41108
Ya’suf	22	26.10483	50.45145
Al-Jasra	23	26.18522	50.44183
Shtaya	24	26.60762	50.81273
Bulthama	25	26.79462	50.91242
West Jarim	26	26.47017	50.4505
Jetty 2	27	26.29179	50.6560
Jetty 3	28	26.24393	50.54117
Wharf Area	29	26.15477	50.6947

The start and end years of sampling were not included in here because they are dependent on the determinant and this information can be found in Tables S5 and S6

For the collection of sediments, a hand-held Van Veen grab was deployed from the boat. The top layer (1–5 cm) of each grab sample was collected using a wooden scoop and immediately transferred to an acid rinsed 500-mL glass jar. At some stations where the sediment was coarse sand, the whole sample was retained. Samples were kept in an icebox then stored in a freezer at − 20 °C upon arrival to the laboratory. These samples were then analysed in a chemical analytical lab for the metals aluminium (Al), Cd, chromium (Cr), copper (Cu), iron (Fe), manganese (Mn), nickel (Ni), Pb and zinc (Zn).

Samples were dried using a freeze dryer (LABCONCO FreeZone 4.5) and sieved. The finest fraction (< 63 μm) was transferred to small glass vials for metal analysis. Before acid digestion, vials were shaken manually for 2 min to homogenise samples. Triplicates of around 0.1–0.3 g of each sample were placed in a Teflon bomb. Then, 5 mL of concentrated nitric acid (HNO_3_) and 2 mL of hydrofluoric acid (HF) were added. The Teflon bombs were kept at room temperature for 1 h; then, they were closed tightly and placed in a Microwave Digestion System (MILESTONE model, ETHOS PLUS). Samples were fully digested by applying the digestion routine illustrated in Table S1. When digestion was complete, samples were left to cool down to room temperature and transferred quantitatively to 50-mL polypropylene graduated tubes containing 0.8 g of boric acid (0.4 g of H_3_BO_3_ for every 1 mL of HF) and made up to 50 mL with deionised water. Digested samples were placed in an ultrasonic bath for 30 min to facilitate dissolution of boric acid.

To ensure quality control, one blank was run along with samples in each digestion batch (1 blank and 11 samples in each batch). Certified reference materials (CRMs) were also digested in triplicates and treated similarly. CRMs used were IAEA-405 (estuarine sediment), IAEA-433 (marine sediment) and IAEA-356 (polluted marine sediment). The adequacy of the CRMs and batch analysis can be seen in Table S2. Concentration of heavy metals in samples, blanks and CRMs were determined using Atomic Absorption Spectroscopy (AAS). Two different instruments were used: Thermo model, M6 SERIES during the period 2007–2018 and Thermo model, iCE 3000 SERIES during the period 2019–2020. Techniques and gases used are illustrated in Table S2. For calibration, a series of standards were prepared by diluting 1000-ppm stock standards. Calibration curves were constructed by at least 3 points along with the blank (zero) and had a fit of at least 0.995.

The raw data were then analysed for status and trend. A spatial status assessment was carried out by comparing AC (Table 2) to sampled metal concentrations in sediment to investigate if these concentrations could have any impacts on marine life in a specific area.

**Table 2:**
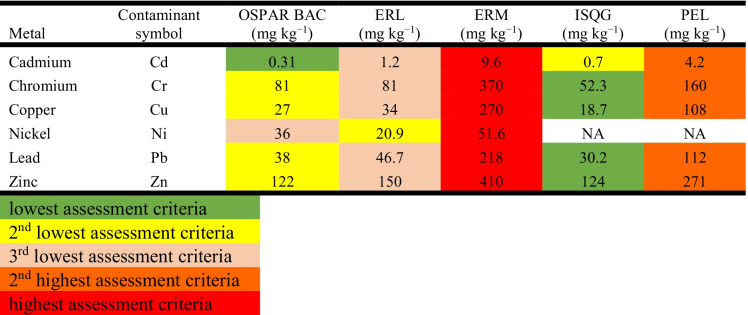
Internationally agreed assessment criteria for metals

Bahrain does not yet have formally adopted sediment quality guidelines (SQGs) (Abdulla & Naser, 2021), so the available data were assessed against a range of internationally adopted AC (Table 2). The AC used in this manuscript were recently described by Bersuder et al. (2020), which include OSPAR Background Assessment Concentrations (BACs; OSPAR, 2008), United States Environmental Protection Agency Effects Range Low (US EPA ERL; Long & MacDonald, 1998), US EPA Effects Range Median (ERM; Long & MacDonald, 1998), Canadian Interim Sediment Quality Guidelines (ISQG; Canadian Council of Ministers of the Environment, 2001) and Probable Effect Levels (PEL; Canadian Council of Ministers of the Environment, 2001). Generally, OSPAR BACs are said to be ‘near-background’ (based on samples collected from the North-East Atlantic region) and concentrations below this AC do not pose harm to the marine environment. The US and Canadian quality standards work in a similar way. Concentrations above the ERL or ISQG indicate that small effects may occur to organisms living in or near the assessed sediments, whilst concentrations above the ERM or PEL suggest that some effects are likely to occur to bottom-dwelling organisms (Bersuder et al., 2020; Nicolaus et al., 2015). These standards were established for European and American sediment backgrounds and should be adopted with care, as some baseline metal concentrations observed in the Arabian Gulf region are above various sediment quality guidelines (SQGs; Al-Sarawi et al., 2015; Alshemmari et al., 2010; Bersuder et al., 2020; Lyons et al., 2015). Al-Abdali et al. (1996) proposed metal background concentration for the Arabian Gulf region (Table 2) which was taken into consideration in the discussion. Metal data within the OSPAR region are also normally normalised against 5% Al apart from Spain, where the background concentrations are not as homogenous compared to the other OSPAR regions (OSPAR, 2009). The same approach was taken within this study. The Al mean concentrations around Bahrain (6521.4 mg kg^−1^ or 0.65%) are so low (ranging between 541.5 and 20,354 mg kg^−1^ or 0.05–2.03%), so the decision was taken not to normalise the datasets for this assessment.

The principles of Nicolaus et al. (2015), (2016) and (2017) were followed by using a Risk Characterisation Ratio (RCR) to investigate if concentrations measured are likely to pose a toxicological threat. In summary, the RCR is the ratio between the Measured Environmental Concentration (MEC) and the AC. If the RCR is above 1, then the determinant failed the contaminant specific AC, which then provides environmental managers with an indication that sediment quality in relation to metal contamination may be poor for that location. The RCR also provided a tool to rank and compare the metal concentrations with one another. The higher the RCR for a specific metal is, the greater the potential impact to the marine environment is.

**Fig. 2:**
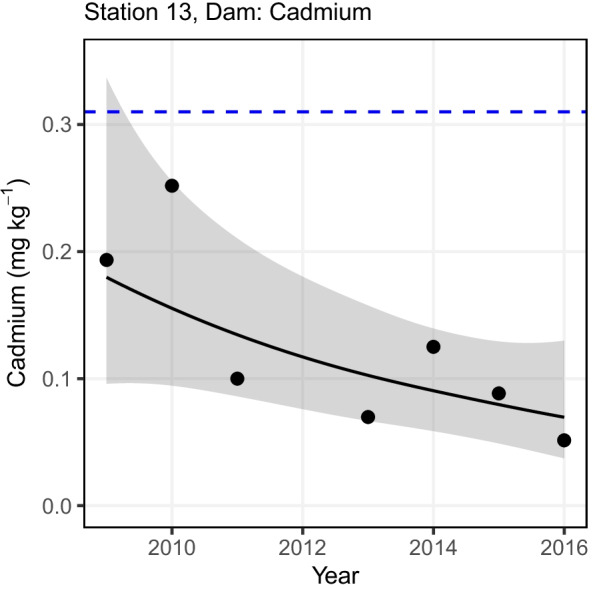
Variation in cadmium concentrations (mg kg^−1^) over time and the fitted GAM model. Also shown is the 95% confidence interval for the fitted GAM model. Blue-dotted line-lowest available assessment criteria for reference (For interpretation of the references to colour in this figure legend, the reader is referred to the web version of this article.)

Whilst the status assessment provides a considered judgment of the current risk posed by metal contamination, it is also important to analyse the direction of any detectable trend to better understand if contaminant concentrations are increasing or decreasing over time. To assess this, a trend assessment was carried out to determine if the contaminant concentrations have improved or deteriorated over the monitoring time series. The same trend assessment methods as described by Nicolaus et al. (2017) were followed by using a generalised additive model (GAM; Wood, 2006) for stations where samples were available in at least five separate years for a specific contaminant at a specific station. The GAM models were fitted using the function ‘gam’ in the R package ‘mgcv’. If only 4 years of data were available, then a linear model was used. No trend assessment was carried out if 3 years or less data were sampled. The GAM analyses incorporate temporal variation over time and are more flexible than linear regression. The gam() function allows us to compare the fitted model against a null hypothesis of ‘no trend’: the null hypothesis can be rejected, if the *p*-value is ≤ 0.05. Figure 2 gives an example for the variation in Cd concentrations over time and the fitted GAM for the station ‘Dam’ (station number 13). The *p*-value for this example was < 0.05 which allowed us to reject the null hypothesis of no trend. To judge the direction of the trend, we examined the direction of the trend at the end of the series — which was downwards in this case. This procedure was followed for all determinants at the different stations where a GAM was fitted. All trend assessment figures can be found in the supplementary section under Figure S1.

OSPAR Background Assessment Concentration (BACs; OSPAR, 2008), United States Environmental Protection Agency Effects Range Low (ES EPA ERL; Long & MacDonald, 1998), US EPA Effects Range Median (ERM; Long & MacDonald, 1998), Canadian Interim Sediment Quality Guidelines (ISQG; Canadian Council of Ministers of the Environment, 2001) and Probable Effect Levels (PEL; Canadian Council of Ministers of the Environment, 2001); proposed background concentrations according to Al-Abdali et al. (1996) are 1.2–2 mg kg^−1^ for Cd, 15–30 mg kg^−1^ for Cu and Pb, 70–80 mg kg^−1^ for Ni and 30–60 mg kg^−1^ for Zn.

## Results and discussion

A summary of the maximum measured environmental concentration (MEC_max_) for a specific metal at a specific station in a specific year and the associated RCR value in relation to the highest and lowest assessment criteria (Table 2) is presented in Table 3. The majority of MEC_max_ values recorded were from historic samples (2008–2012) with only the MEC_max_ for Cu recorded in the last 3 years (2019). Background and summary results, including the mean, minimum and maximum concentrations of all determinants at a specific station including the trend assessment, can be found in Table S4 of the supplementary section. The detailed RCRs for each determinant at each station for each sampled year can be seen in Table S5. Tables S5 and S6 also provide information on the measured environmental concentration for each station in each sampled year.

**Table 3:** Maximum Measured Environmental Concentration (MEC) values recorded and their associated RCR collected in a specific year at a specific site followed by the overall occurrence (%) of RCRs taking all collected samples into consideration since 2007

Contaminant	MEC_max_ (mg kg^−1^)	Highest RCR	Lowest RCR	Station	Sample year	% above lowest AC	% above highest AC	*N*
Al	20,400	NA	NA	Gasar (20)	2009	NA	NA	240
Cd	0.317	0.08 (ERM)	1.02 (BAC)	Mashtan (2)	2008	0.77	0.00	259
Cr	528	1.43 (ERM)	10.1 (ISQG)	Ya’suf (22)	2008	19.63	2.22	270
Cu	280	1.04 (ERM)	14.95 (ISQG)	Shtaya (24)	2019	25.08	0.72	279
Fe	24,100	NA	NA	Suhain (10)	2009	NA	NA	240
Mn	290	NA	NA	Ya’suf (22)	2008	NA	NA	279
Ni	92.6	1.79 (ERM)	4.43(ERL)	Refinery area (8)	2012	7.14	1.88	266
Pb	213	0.98 (ERM)	7.05 (ISQG)	Refinery area (8)	2009	6.45	0.00	279
Zn	92.6	0.23 (ERM)	0.76 (BAC)	Refinery area (8)	2012	NA	NA	269

*MEC* Measured Environmental Concentration, *BAC* Background Assessment Concentrations, *ERL* Effect Range Low, *ERM* Effect Range Median, *ISQG* Canadian Interim Sediment Quality Guidelines, *RCR* Risk Characterisation Ratio for either OSPAR BAC, ISQG, ERL or ERM, *AC* Assessment Criteria, *N* number of samples

As an example of the risk assessments undertaken, the MEC_max_ for Cr and Cu were 528 and 280 mg kg^−1^, respectively (Table 3). To assess these concentrations, they were compared to the lowest AC, which indicated that the Cr concentration was 10 times (RCR = 10.1) above the ISQC. Similarly, Cu was almost 15 times (RCR = 14.95) above the lowest AC (ISQG). Exceeding the lowest AC highlights that there is a potential toxicological risk to exposed marine organisms and confidence around this result increases as other, higher AC are breached. Therefore, the MECs were also compared to these AC. Table 3 highlights that individual Cr, Cu and Ni concentration had a RCR above 1 in these instances, suggesting that organisms at these stations may be at risk due to elevated pollution levels. Looking at the dates when these samples were collected, it becomes apparent that these MECs were from historic samples, apart from Cu, which was only measured in 2019 at station Shtaya (station number 24). As such the data highlights the importance on long-term data collection, which allows environmental managers to establish if such values are outliers or are indicative of new pollution sources within a site. Table 3 also shows the occurrences (%) of the various metals that breached the lowest and highest AC. As an example, 25% of all Cu samples and almost 20% of all Cr samples breached the lowest AC, whilst less than 1% of Cd samples exceeded the lowest AC, indicating that Cd contamination is not a problem in Bahrain marine waters. Some attention should be given to Cr and Ni as these two metals had historic exceedances of around 2% across all collected samples, when compared to the highest AC (Table 3).

To establish if the historic exceedances are still an issue in the present time, an overview is presented in Table , highlighting the status of a metal in the last sampled year and the trend assessment observed at a specific station. It can be seen from that, that Cr (2 out of 29 sites), Cu (1 out of 29 sites) and Ni (1 out of 29 sites) still measured concentrations in the last sampled year above the highest AC at a small number of locations (Table 4). Revisiting the above-mentioned Cu concentration sampled in 2019 at Shtaya (station number 24), which exceeded the highest AC (ERM) in this year, it can be seen that the Cu concentration has decreased (downward arrow in Table 4), but still exceeded the ERL AC in 2020.

**Table 4:**
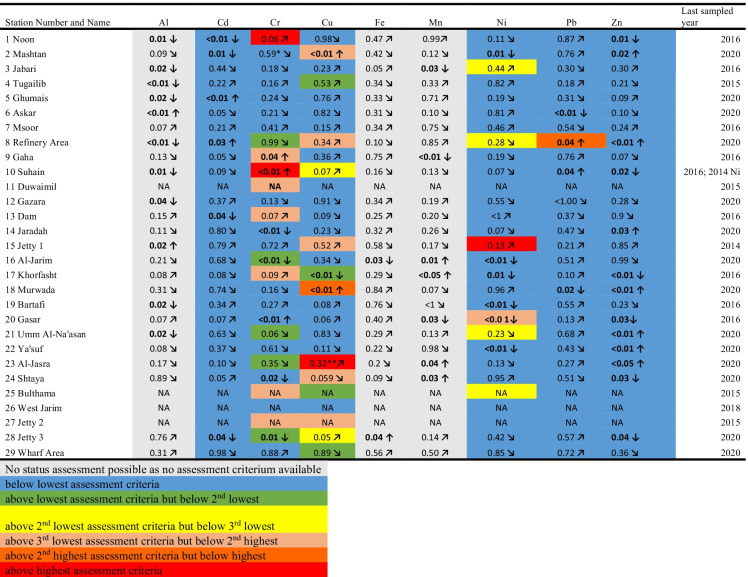
Summary results of status and trend assessment for trace elements collected in Bahrain sediments

The assessment criteria can be seen in Table 2

NA no trend assessment possible as less than 4 years of data

Numbers represent the *p*-values at the 95% Confidence Interval

^*^Cr at station 2 failed the ERL in 2019 and 2018

^**^Cu at station 23 almost 10-fold higher compared to next highest year, 2019 and 2018 below lowest EAC; For NI; if above ERL then yellow, if above BAC then orange, if above ERM then purple

↓ Significant decrease (*p*-value < 0.05)

↑ Significant increase (*p*-value < 0.05)

↘ non-significant decrease (*p*-value > 0.05)

↗ non-significant increase (*p*-value > 0.05)

Whilst the status assessment provides valuable information about the current concentrations in relation to the AC, a status assessment gives an indication if concentrations in recent years are decreasing or increasing. In total, 59 significant trends were observed for the various determinants at the 29 stations (Tables 4 and S4). Of these, 23 were significantly upwards whilst 36 were significantly downwards. The most significant upward trends were observed for Zn and Mn (7 and 4 significant upward trends, respectively). The most significant downward trends were observed for Al, Zn and Ni (9, 6 and 6 significant downward trends, respectively). Station 8 (Refinery Area) is the only station where three metals (Cd, Pb and Zn) showed significant upward trends. Stations Jetty 3 (station number 28), Gasar (station number 20), Khorfasht (station number 17), Al-Jarim (station number 16) and Noon (station number 1) showed the most significant downward trends (3 significant downward trends) for one station.

The significant decreases in Ni could be linked to the way crude oil has been extracted over the survey period, as Al-Abdali et al. (1996) highlighted that Ni is the largest metal constituent of crude oil and naturally occurs in higher concentrations than in non-oil field areas.

Such trend information allows environmental managers to focus their resources and develop a list of priority sites for further monitoring or investigative studies (Abdulla & Naser, 2021). Going forward, it would be advisable to re-sample stations Noon and Suhain, as these were last sampled in 2016 and displayed exceedances against the highest AC (ERM) for Cr in the last sampled year. The trend analysis for these metals at these sites also showed an upward trend and in the case of Cr at station Suhain, this upward trend was statistically significant (*p* ≤ 0.01; Table 4).

The results of these sites indicate that the ongoing pollution needs continued monitoring. This was addressed in a recent study by Bersuder et al. (2020), who conducted a detailed survey of sediments around the Refinery Area and found that metal contamination was relatively localised around the Refinery Area station and not widespread within that region of coast. More detail on which metal exceeded the relevant AC in the last sampled year and which trends for a specific metal are increasing or decreasing at a specific station can be seen in Table 4.

Long and MacDonald (1998) suggested that determinants that equal the ERLs or ISQGs or exceed them by a moderate amount (e.g. RCRs of 1.1 to 9.9) should be viewed as chemicals of concern. Chemicals that most frequently exceeded the ERLs or ISQGs and by the greatest amount (e.g. RCR ≥ 10) should be viewed as chemicals of highest priority for further monitoring. Within this study, Cu sampled at station Al-Jasra (station number 23) would fall within this category. The calculated Cu RCR_ISQG_ and RCR_ERL_ were 14.74 and 8.1, respectively, which would suggest that organisms like filter, suspension and deposit feeders may be at risk to contamination exposure (Bryan & Langston, 1992). Stations along the south east (one to five) are within the main habitats of dugongs, and according to the results within this study, they are in relatively pristine habitats in relation of metal contamination. However, current data within this area is sparse and it would be advisable to re-initiate sampling at these locations (stations 1, 3 and 4) as they were last sampled between 2015 and 2016 and some determinants like Cr, Ni and Cu highlighted increasing contaminant concentrations and failures for the mid-level assessment criteria.

A common industrial contaminant of concern is Cd, and it is positive that relatively low Cd concentrations were reported within this study (mean equals 0.11 mg kg^−1^). More than 99% of all Cd concentrations were below the BAC. Cd can affect sensitive benthic taxonomic groups like mollusca, polycheata, echinodermata, crustacea and platyhelminthes already at low concentrations (0.0014 mg kg^−1^ of Cd affect 5% of sensitive organisms, whilst over 90% were affected at 1.0 mg kg^−1^ according to Leung et al. (2005). The low Cd concentration in the sediment measured in this study and recently confirmed by Bersuder et al. (2020) would indicate that there is a low risk of Cd accumulating in the food chain and impacting some of the higher trophic fauna (e.g. dugongs and turtles) that graze on the extensive sea grass beds around Bahrain’s coast (Burt et al., 2013; Naser, 2015). Al-Abdali et al. (1996) suggested in his study that Cd (like Zn and Mn) are thought to be natural constituents of the Gulf marine environment and therefore not elements derived from pollutant sources.

Lead, which is seen as a priority hazardous substance within the OSPAR region (OSPAR, 2008), has also shown relatively low concentrations within this study in the last sampled year. Only the Refinery Area (station number 8) was above the second highest assessment criteria (PEL), but still below the ERM. Nevertheless, the significant upward trend at this station and at Suhain (station number 10) should be monitored carefully. Bersuder et al. (2020) sampled eight stations around the Refinery Area (station number 8) and observed Pb concentrations varying between 6.85 and 277 mg kg^−1^ within a 5-km radius in 2019. Such data indicate that spatial variation, even in closely located areas, needs to be considered to ensure that data are truly representative of the location being studied. The observed Cu concentration of 64.2 mg kg^−1^ within this study at the Refinery Area (station number 8) in 2020 also aligns with the observed concentration by Bersuder et al. (2020) at a co-located site of 70.1 mg kg^−1^.

## Conclusion

In general, the metal concentrations in sediments observed in this study were satisfactory. Only two Cr, one Cu and one Ni concentration in the last sampled year were above the ERM. Nevertheless, the trend analysis highlighted that 23 trends were significantly upwards including determinants that were already above the highest and second highest assessment criteria. The study also showed that the south east (stations two to five) is a relatively pristine area in relation to the observed metal contamination, but resampling would need to be carried out to confirm this. Cd, which is a common industrial contaminant of concern, also showed relatively low concentrations within this study. More than 99% of all Cd concentrations were below the BAC. Lead has also shown relatively low concentrations within this study in the last sampled year.

Going forward, more area specific surveys should be conducted to investigate local variation further. Bersuder et al. (2020) conducted such an intensive survey around a known impacted site on the Eastern side of Bahrain and similar studies should be conducted at other locations to get a better understanding on local contaminant variations. Additionally, samples should also be analysed for mercury. Taking benthic community samples may also help in identifying biodiversity and abundance hot spots and if these are affected by local contaminant variations. Understanding of the toxicological threat posed by chemical contamination to marine organisms in the Arabian Gulf is an emerging field (Khatir et al., 2020) and further work is required to develop a suite of biological effects tools (e.g. bioassay and biomarkers) that are adapted to local species and can support chemical led monitoring programmes such as described here (Beg et al., 2015; Butler et al., 2020; Smith et al., 2015). Overall, the data presented provides a baseline against which future assessments can be conducted which can support both national and regional efforts to assess marine environmental health (Abdulla & Naser, 2021; Devlin et al., 2019; ROPME, 2013).

## Supplementary Information

Below is the link to the electronic supplementary material.

Supplementary file1 (DOCX 614 KB)

Supplementary file2 (PDF 704 KB)
