# Supplementary material for: Spatial and temporal analysis of the risks posed by metal contamination in coastal and marine sediments of Bahrain
Source: Environ Monit Assess. 2022 Jan 6;194(2):62. doi: 10.1007/s10661-021-09722-7 (PMC8739313; doi:10.1007/s10661-021-09722-7)
Supplement: Supplementary file 1 — Supplementary file1 (DOCX 614 KB) [file 10661_2021_9722_MOESM1_ESM.docx]

Table S1. The digestion of sediment samples

| Step | Power (W) | %Power | Ramp time *(min)* | PSI | C | Hold Time *(min)* |
| --- | --- | --- | --- | --- | --- | --- |
| 1 | 1200 | 100 | 5 | 600 | 50 | 5 |
| 2 | 1200 | 100 | 5 | 600 | 100 | 5 |
| 3 | 1200 | 100 | 10 | 600 | 200 | 12 |

Table S2. Techniques and gases used for metals analysis

| Metal | Air-Acetylene | Air-Acetylene-Nitrous Oxide | Argon | Technique |
| --- | --- | --- | --- | --- |
| Zn – Zinc | 🗸 |  |  | Flame |
| Cu – Copper | 🗸 |  |  | Flame |
| Ni – Nickel | 🗸 |  |  | Flame |
| Fe – Iron | 🗸 |  |  | Flame |
| Mn - Manganese | 🗸 |  |  | Flame |
| Cr – Chromium |  | 🗸 |  | Flame |
| Al – Aluminum |  | 🗸 |  | Flame |
| Cd – Cadmium |  |  | 🗸 | Graphite Furnace |
| Pb – Lead |  |  | 🗸 | Graphite Furnace |

Figure S1. Plotted Measured Environmental Concentrations (mg kg^−1^) over time and the fitted GAM model. Also shown is the 95% confidence interval for the fitted GAM model

Table S3. Results of the Certified Reference Material and Batch analysis

| Metal | Cd (mg/kg) | | Pb (mg/kg) | | Al (mg/kg) | | Cr (mg/kg) | | Zn (mg/kg) | | Cu (mg/kg) | | Fe (mg/kg) | | Mn (mg/kg) | | Ni (mg/kg) | |
| --- | --- | --- | --- | --- | --- | --- | --- | --- | --- | --- | --- | --- | --- | --- | --- | --- | --- | --- |
| Ref Value | 0.73 | | 74.8 | | 77900 | | 84 | | 279 | | 47.7 | | 37400 | | 495 | | 32.5 | |
| Metal | Cd (mg/kg) | | Pb (mg/kg) | | Al (mg/kg) | | Cr (mg/kg) | | Zn (mg/kg) | | Cu (mg/kg) | | Fe (mg/kg) | | Mn (mg/kg) | | Ni (mg/kg) | |
| Year | Result | Recovery | Result | Recovery | Result | Recovery | Result | Recovery | Result | Recovery | Result | Recovery | Result | Recovery | Result | Recovery | Result | Recovery |
| 2007 | 0.650 | 89% | NA | NA | NA | NA | 90.4 | 108% | 260.9 | 94% | 42.2 | 88% | NA | NA | 439.8 | 89% | 29.2 | 90% |
| 2008 | NA | NA | 72.9 | 97% | NA | NA | NA | NA | 281.9 | 101% | 46.5 | 97% | NA | NA | 465.0 | 94% | 26.9 | 83% |
| 2009 | 0.709 | 97% | 74.7 | 100% | 76616 | 98% | 78.9 | 94% | 264.4 | 95% | 45.7 | 96% | NA | NA | 424.6 | 86% | 30.1 | 93% |
| 2010 | 0.770 | 105% | 64.3 | 86% | NA | NA | 84.2 | 100% | 278.4 | 100% | 44.1 | 92% | 35223 | 94% | 452.9 | 91% | 33.7 | 104% |
| 2011 | 0.824 | 113% | 72.4 | 97% | 76925 | 99% | 88.8 | 106% | 274.5 | 98% | 47.3 | 99% | 37520 | 100% | 510.0 | 103% | 30.2 | 93% |
| 2012 | NA | NA | 68.9 | 92% | 67744 | 87% | 82.8 | 99% | 269.6 | 97% | 45.3 | 95% | 38189 | 102% | 430.5 | 87% | 32.4 | 100% |
| 2013 | 0.775 | 106% | 71.3 | 95% | 83672 | 107% | 88.2 | 105% | 260.6 | 93% | 48.7 | 102% | 37528 | 100% | 516.1 | 104% | 28.6 | 88% |
| 2014 | 0.642 | 88% | 71.4 | 95% | 72664 | 93% | 85.8 | 102% | 275.6 | 99% | 51.1 | 107% | 40290 | 108% | 499.8 | 101% | 29.4 | 90% |
| 2015 | 0.709 | 97% | 60.8 | 81% | NA | NA | 79.2 | 94% | 302.1 | 108% | 49.7 | 104% | 41057 | 110% | 493.3 | 100% | 30.1 | 93% |
| 2016 | 0.836 | 115% | 61.6 | 82% | 77907 | 100% | 85.2 | 101% | 276.3 | 99% | 42.6 | 89% | 31509 | 84% | 485.4 | 98% | 28.5 | 88% |
| 2017 | 0.671 | 92% | 69.1 | 92% | NA | NA | 69.7 | 83% | 282.2 | 101% | 49.9 | 105% | NA | NA | 490.5 | 99% | 36.0 | 111% |
| 2018 | 0.765 | 105% | 65.7 | 88% | 88891 | 114% | 92.5 | 110% | 287.5 | 103% | 50.1 | 105% | NA | NA | 481.5 | 97% | 26.3 | 81% |
| 2019 | 0.779 | 107% | 66.5 | 89% | 71381 | 92% | 73.2 | 87% | 281.6 | 101% | 47.7 | 100% | 33031 | 88% | 482.3 | 97% | 29.3 | 90% |
| 2020 | 0.742 | 102% | 75.7 | 101% | 81152 | 104% | 86.8 | 103% | 290.6 | 104% | 48.7 | 102% | 36527 | 98% | 489.7 | 99% | 28.3 | 87% |
| CRM | IAEA 433 |  |  |  |  |  |  |  |  |  |  |  |  |  |  |  |  |  |
| Metal | Cd (mg/kg) | | Pb (mg/kg) | | Al (mg/kg) | | Cr (mg/kg) | | Zn (mg/kg) | | Cu (mg/kg) | | Fe (mg/kg) | | Mn (mg/kg) | | Ni (mg/kg) | |
| Ref Value | 0.153 | | 26 | | 78200 | | 136 | | 101 | | 30.8 | | 40800 | | 316 | | 39.4 | |
| Metal | Cd (mg/kg) | | Pb (mg/kg) | | Al (mg/kg) | | Cr (mg/kg) | | Zn (mg/kg) | | Cu (mg/kg) | | Fe (mg/kg) | | Mn (mg/kg) | | Ni (mg/kg) | |
| Year | Result | Recovery | Result | Recovery | Result | Recovery | Result | Recovery | Result | Recovery | Result | Recovery | Result | Recovery | Result | Recovery | Result | Recovery |
| 2007 | NA | NA | 24.3 | 93% | NA | NA | NA | NA | 93.8 | 93% | NA | NA | NA | NA | 287.7 | 91% | NA | NA |
| 2008 | 0.171 | 112% | 27.3 | 105% | NA | NA | 134.5 | 99% | 101.9 | 101% | 27.1 | 88% | NA | NA | 371.8 | 118% | 39.5 | 100% |
| 2009 | 0.142 | 93% | NA | NA | 83796 | 107% | 127.6 | 94% | 94.4 | 93% | 27.8 | 90% | 41880 | 103% | 280.8 | 89% | 35.5 | 90% |
| 2010 | 0.142 | 93% | 26.9 | 103% | 83580 | 107% | 153.9 | 113% | 101.0 | 100% | 30.2 | 98% | 37808 | 93% | 294.9 | 93% | 41.7 | 106% |
| 2011 | 0.130 | 85% | 27.1 | 104% | NA | NA | 129.9 | 96% | 100.2 | 99% | 29.1 | 94% | 39849 | 98% | 321.7 | 102% | 37.6 | 95% |
| 2012 | 0.147 | 96% | 24.7 | 95% | 77063 | 99% | 133.6 | 98% | 101.7 | 101% | 30.0 | 97% | NA | NA | 287.1 | 91% | 33.7 | 86% |
| 2013 | 0.151 | 99% | 25.8 | 99% | 84626 | 108% | 138.7 | 102% | 90.8 | 90% | 33.5 | 109% | 45927 | 113% | 324.6 | 103% | 37.3 | 95% |
| 2014 | 0.170 | 111% | 25.4 | 98% | 75914 | 97% | 147.7 | 109% | 101.9 | 101% | 29.8 | 97% | 42401 | 104% | 308.8 | 98% | 39 | 99% |
| 2015 | 0.157 | 103% | 26.8 | 103% | 73432 | 94% | 139.7 | 103% | NA | NA | NA | NA | NA | NA | 305.2 | 97% | 38.6 | 98% |
| 2016 | 0.146 | 95% | 25.8 | 99% | 78725 | 101% | 135.5 | 100% | 95.5 | 95% | 30.7 | 100% | 40973 | 100% | 303 | 96% | 36.7 | 93% |
| 2017 | 0.147 | 96% | 25.3 | 97% | 66068 | 84% | 135.9 | 100% | 105.4 | 104% | 31.7 | 103% | 41567 | 102% | 312.8 | 99% | 35.0 | 89% |
| 2018 | 0.141 | 92% | 25.6 | 99% | 84360 | 108% | 153.5 | 113% | 105.1 | 104% | 31.4 | 102% | 49735 | 122% | 282.0 | 89% | 34.0 | 86% |
| 2019 | 0.147 | 96% | 28.6 | 110% | 75484 | 97% | 141.8 | 104% | 107.7 | 107% | 30.8 | 100% | 41855 | 103% | 313.4 | 99% | 35.4 | 90% |
| 2020 | 0.155 | 101% | 25.9 | 100% | 70692 | 90% | 137.1 | 101% | 102.7 | 102% | 30.0 | 97% | 40814 | 100% | 296.2 | 94% | 31.5 | 80% |
| CRM | IAEA 356 |  |  |  |  |  |  |  |  |  |  |  |  |  |  |  |  |  |
| Metal | Cd (mg/kg) | | Pb (mg/kg) | | Al (mg/kg) | | Cr (mg/kg) | | Zn (mg/kg) | | Cu (mg/kg) | | Fe (mg/kg) | | Mn (mg/kg) | | Ni (mg/kg) | |
| Ref Value | 4.47 | | 347 | | 39000 | | 69.8 | | 977 | | 365 | | 24100 | | 312 | | 36.9 | |
| Metal | Cd (mg/kg) | | Pb (mg/kg) | | Al (mg/kg) | | Cr (mg/kg) | | Zn (mg/kg) | | Cu (mg/kg) | | Fe (mg/kg) | | Mn (mg/kg) | | Ni (mg/kg) | |
| Year | Result | Recovery | Result | Recovery | Result | Recovery | Result | Recovery | Result | Recovery | Result | Recovery | Result | Recovery | Result | Recovery | Result | Recovery |
| 2019 | 4.182 | 94% | 327.3 | 94% | 36869 | 95% | 84.9 | 122% | 1061.5 | 109% | 405.4 | 111% | 28026 | 116% | 328.1 | 105% | 29.7 | 80% |
| 2020 | 4.008 | 90% | 377.4 | 109% | 33552 | 86% | 71.5 | 102% | 1025.7 | 105% | 388.5 | 106% | 26314 | 109% | 326.4 | 105% | 29.8 | 81% |
|  | |  |  |  |  |  |  |  |  |  |  |  |  |  |  |  |  |  |
| Blank | mg/kg equivalent | |  |  |  |  |  |  |  |  |  |  |  |  |  |  |  |  |
| Metal | Cd (mg/kg) | Pb (mg/kg) | Al (mg/kg) | Cr (mg/kg) | Zn (mg/kg) | Cu (mg/kg) | Fe (mg/kg) | Mn (mg/kg) | Ni (mg/kg) |  |  |  |  |  |  |  |  |  |
| Year | Result | Result | Result | Result | Result | Result | Result | Result | Result |  |  |  |  |  |  |  |  |  |
| 2011 | 0.015 | 0.15 | -32 | 1.5 | 4.5 | 0.8 | 15 | 0.0 | 1.5 |  |  |  |  |  |  |  |  |  |
| 2012 | 0.000 | -0.05 | -12 | 1.5 | 1.4 | -1.5 | 45 | 1.5 | -1.5 |  |  |  |  |  |  |  |  |  |
| 2013 | 0.015 | 0.00 | -15 | 0.0 | 0.0 | 1.5 | 15 | 1.5 | -1.5 |  |  |  |  |  |  |  |  |  |
| 2014 | 0.030 | 0.03 | -15 | 1.5 | 0.0 | 0.0 | 0 | 0.0 | 0.0 |  |  |  |  |  |  |  |  |  |
| 2015 | 0.000 | 0.00 | -15 | -1.5 | -3.0 | 1.5 | 0 | 1.5 | 0.0 |  |  |  |  |  |  |  |  |  |
| 2016 | 0.000 | 0.00 | -11 | 1.5 | -1.5 | 1.5 | 0 | 0.0 | -1.5 |  |  |  |  |  |  |  |  |  |
| 2017 | 0.015 | 0.02 | 0 | 0.0 | -1.5 | -1.5 | 30 | 0.0 | -1.5 |  |  |  |  |  |  |  |  |  |
| 2018 | 0.000 | 0.02 | 0 | -1.5 | -1.5 | 0.0 | 0 | 1.5 | -1.5 |  |  |  |  |  |  |  |  |  |
| 2019 | 0.000 | 0.00 | 15 | 0.0 | -1.5 | -1.5 | 45 | 0.0 | 0.0 |  |  |  |  |  |  |  |  |  |
| 2020 | 0.000 | 0.00 | 0 | 0.0 | -1.5 | 0.0 | 0 | 1.5 | -1.5 |  |  |  |  |  |  |  |  |  |

Table S4. Summary data including trend assessment of each determinant sampled at each station

| Station Name | Site No. | Determinant | Mean MEC (mg kg^-1^) | SE Mean* | St Dev* | Median* | Min* | Max* | p-Value | Trend direction | Start Year | Last Year | N |
| --- | --- | --- | --- | --- | --- | --- | --- | --- | --- | --- | --- | --- | --- |
| Noon | 1 | Al | 9330 | 1110 | 3150 | 9380 | 5600 | 14300 | **0.011** | **Down** | 2009 | 2016 | 8 |
| Noon | 1 | Cd | 0.13 | 0.02 | 0.05 | 0.1 | 0.09 | 0.25 | **0.003** | **Down** | 2008 | 2016 | 9 |
| Noon | 1 | Cr | 154 | 49.3 | 156 | 72 | 49.3 | 435 | 0.06 | Up | 2007 | 2016 | 10 |
| Noon | 1 | Cu | 15.8 | 1.47 | 4.66 | 15.8 | 9.08 | 26 | 0.977 | Down | 2007 | 2016 | 10 |
| Noon | 1 | Fe | 5080 | 879 | 2480 | 4490 | 2530 | 9530 | 0.473 | Up | 2009 | 2016 | 8 |
| Noon | 1 | Mn | 107 | 14.5 | 45.8 | 108 | 51.7 | 217 | 0.992 | Up | 2007 | 2016 | 10 |
| Noon | 1 | Ni | 26.5 | 2.69 | 8.52 | 28.1 | 8.13 | 35.8 | 0.105 | Down | 2007 | 2016 | 10 |
| Noon | 1 | Pb | 3.12 | 0.49 | 1.55 | 2.59 | 0.92 | 5.69 | 0.871 | Up | 2007 | 2016 | 10 |
| Noon | 1 | Zn | 20.1 | 3.33 | 10.5 | 18.2 | 9.09 | 39.5 | **0.012** | **Down** | 2007 | 2016 | 10 |
| Mashtan | 2 | Al | 7620 | 1340 | 4650 | 5900 | 2600 | 20300 | 0.092 | Down | 2009 | 2020 | 12 |
| Mashtan | 2 | Cd | 0.13 | 0.02 | 0.06 | 0.12 | 0.07 | 0.32 | **0.01** | **Down** | 2008 | 2020 | 13 |
| Mashtan | 2 | Cr | 106 | 18.7 | 69.9 | 77.2 | 47.4 | 280 | 0.593 | Down | 2007 | 2020 | 14 |
| Mashtan | 2 | Cu | 25.6 | 5.29 | 19.8 | 20.3 | 7.16 | 82.6 | **<0.001** | **Up** | 2007 | 2020 | 14 |
| Mashtan | 2 | Fe | 3640 | 297 | 1030 | 3460 | 2030 | 5110 | 0.42 | Down | 2009 | 2020 | 12 |
| Mashtan | 2 | Mn | 93 | 9.94 | 37.2 | 75.8 | 52.3 | 167 | 0.124 | Down | 2007 | 2020 | 14 |
| Mashtan | 2 | Ni | 18.4 | 1.43 | 5.35 | 16.6 | 11.8 | 32 | **0.013** | **Down** | 2007 | 2020 | 14 |
| Mashtan | 2 | Pb | 3.72 | 0.65 | 2.42 | 2.59 | 1.42 | 7.95 | 0.76 | Up | 2007 | 2020 | 14 |
| Mashtan | 2 | Zn | 18.4 | 2.35 | 8.8 | 17.2 | 4.54 | 42.4 | **0.024** | **Up** | 2007 | 2020 | 14 |
| Jabari | 3 | Al | 8530 | 1020 | 2890 | 7740 | 5900 | 14000 | **0.022** | **Down** | 2009 | 2016 | 8 |
| Jabari | 3 | Cd | 0.1 | 0.01 | 0.02 | 0.1 | 0.06 | 0.14 | 0.445 | Down | 2008 | 2016 | 9 |
| Jabari | 3 | Cr | 48.5 | 2.6 | 8.22 | 47.5 | 38.9 | 64.6 | 0.185 | Down | 2007 | 2016 | 10 |
| Jabari | 3 | Cu | 14 | 1.51 | 4.79 | 15 | 6.01 | 21.2 | 0.226 | Up | 2007 | 2016 | 10 |
| Jabari | 3 | Fe | 5050 | 236 | 669 | 4890 | 4280 | 6350 | 0.051 | Up | 2009 | 2016 | 8 |
| Jabari | 3 | Mn | 87.1 | 2.84 | 8.99 | 85.8 | 71.7 | 101 | **0.034** | **Down** | 2007 | 2016 | 10 |
| Jabari | 3 | Ni | 25.2 | 2.65 | 8.39 | 24.3 | 9.03 | 38.8 | 0.438 | Up | 2007 | 2016 | 10 |
| Jabari | 3 | Pb | 4.15 | 0.41 | 1.29 | 4.01 | 2.46 | 6.16 | 0.3 | Down | 2007 | 2016 | 10 |
| Jabari | 3 | Zn | 23.2 | 2.8 | 8.84 | 23.5 | 4.91 | 39.5 | 0.303 | Up | 2007 | 2016 | 10 |
| Tugailib | 4 | Al | 6290 | 933 | 2470 | 5680 | 3560 | 10900 | **0.002** | **Down** | 2009 | 2015 | 7 |
| Tugailib | 4 | Cd | 0.11 | 0.02 | 0.05 | 0.1 | 0.05 | 0.21 | 0.225 | Up | 2008 | 2015 | 8 |
| Tugailib | 4 | Cr | 50.8 | 10.5 | 23.5 | 41.9 | 25.9 | 88.3 | 0.158 | Up | 2007 | 2015 | 5 |
| Tugailib | 4 | Cu | 20.4 | 2.96 | 8.89 | 21.1 | 1.65 | 30.9 | 0.535 | Up | 2007 | 2015 | 9 |
| Tugailib | 4 | Fe | 3680 | 252 | 666 | 3500 | 2510 | 4470 | 0.339 | Down | 2009 | 2015 | 7 |
| Tugailib | 4 | Mn | 78.2 | 13.9 | 41.7 | 57.6 | 34.4 | 170 | 0.33 | Up | 2007 | 2015 | 9 |
| Tugailib | 4 | Ni | 18.9 | 2.58 | 6.82 | 18.7 | 5.95 | 28.6 | 0.821 | Up | 2009 | 2015 | 7 |
| Tugailib | 4 | Pb | 5.34 | 1.19 | 3.56 | 3.98 | 1.83 | 10.7 | 0.179 | Up | 2007 | 2015 | 9 |
| Tugailib | 4 | Zn | 17.7 | 2.93 | 7.75 | 17.9 | 8.57 | 31 | 0.209 | Down | 2009 | 2015 | 7 |
| Ghumais | 5 | Al | 7380 | 664 | 2300 | 6950 | 4560 | 13300 | **0.017** | **Down** | 2009 | 2020 | 12 |
| Ghumais | 5 | Cd | 0.1 | 0.01 | 0.02 | 0.09 | 0.07 | 0.15 | **<0.001** | **Up** | 2008 | 2020 | 13 |
| Ghumais | 5 | Cr | 47 | 2.7 | 10.1 | 46.4 | 28.6 | 72 | 0.243 | Down | 2007 | 2020 | 14 |
| Ghumais | 5 | Cu | 12.2 | 0.99 | 3.69 | 11.7 | 5.89 | 18.7 | 0.758 | Up | 2007 | 2020 | 14 |
| Ghumais | 5 | Fe | 6180 | 306 | 1060 | 6020 | 4930 | 8100 | 0.331 | Down | 2009 | 2020 | 12 |
| Ghumais | 5 | Mn | 102 | 4.8 | 17.9 | 108 | 52.2 | 120 | 0.708 | Up | 2007 | 2020 | 14 |
| Ghumais | 5 | Ni | 25.4 | 2.42 | 9.07 | 25 | 5.49 | 40.8 | 0.193 | Down | 2007 | 2020 | 14 |
| Ghumais | 5 | Pb | 2.87 | 0.32 | 1.19 | 2.52 | 1.31 | 5.28 | 0.308 | Down | 2007 | 2020 | 14 |
| Ghumais | 5 | Zn | 18.6 | 1.69 | 6.32 | 19 | 6.29 | 31.8 | 0.088 | Up | 2007 | 2020 | 14 |
| Askar | 6 | Al | 6700 | 602 | 2090 | 6390 | 3950 | 12300 | **0.005** | **Up** | 2009 | 2020 | 12 |
| Askar | 6 | Cd | 0.1 | 0.01 | 0.02 | 0.1 | 0.08 | 0.14 | 0.055 | Down | 2008 | 2020 | 13 |
| Askar | 6 | Cr | 52 | 2.55 | 9.53 | 50.6 | 35 | 70.3 | 0.209 | Down | 2007 | 2020 | 14 |
| Askar | 6 | Cu | 20.6 | 2.66 | 9.94 | 17.8 | 8.7 | 49.2 | 0.819 | Down | 2007 | 2020 | 14 |
| Askar | 6 | Fe | 4530 | 271 | 939 | 4060 | 3750 | 6400 | 0.307 | Down | 2009 | 2020 | 12 |
| Askar | 6 | Mn | 76.4 | 3.01 | 11.3 | 73.9 | 59.4 | 101 | 0.1 | Down | 2007 | 2020 | 14 |
| Askar | 6 | Ni | 17.3 | 2.61 | 9.77 | 14.4 | 2.06 | 40.7 | 0.807 | Up | 2007 | 2020 | 14 |
| Askar | 6 | Pb | 11.7 | 1.18 | 4.43 | 12.3 | 4.57 | 17.2 | **0.007** | **Down** | 2007 | 2020 | 14 |
| Askar | 6 | Zn | 30.6 | 2.05 | 7.65 | 28.6 | 16.8 | 45.6 | 0.105 | Down | 2007 | 2020 | 14 |
| Msoor | 7 | Al | 5340 | 1110 | 3140 | 4750 | 2230 | 12800 | 0.068 | Up | 2009 | 2016 | 8 |
| Msoor | 7 | Cd | 0.1 | 0.01 | 0.02 | 0.1 | 0.07 | 0.13 | 0.213 | Up | 2008 | 2016 | 9 |
| Msoor | 7 | Cr | 36.4 | 3.57 | 11.3 | 34.4 | 16.5 | 54.5 | 0.406 | Up | 2007 | 2016 | 10 |
| Msoor | 7 | Cu | 12.9 | 1.43 | 4.53 | 12 | 6.88 | 19.1 | 0.15 | Up | 2007 | 2016 | 10 |
| Msoor | 7 | Fe | 4350 | 300 | 849 | 4120 | 3450 | 6270 | 0.344 | Up | 2009 | 2016 | 8 |
| Msoor | 7 | Mn | 49.5 | 2.75 | 8.7 | 48.2 | 35.7 | 68.2 | 0.753 | Down | 2007 | 2016 | 10 |
| Msoor | 7 | Ni | 16.1 | 2.71 | 8.57 | 16 | 1.23 | 26.8 | 0.463 | Up | 2007 | 2016 | 10 |
| Msoor | 7 | Pb | 2.34 | 0.33 | 1.05 | 2.69 | 0.45 | 3.82 | 0.536 | Down | 2007 | 2016 | 10 |
| Msoor | 7 | Zn | 20.2 | 2.82 | 8.92 | 21.6 | 2.36 | 36.6 | 0.24 | Up | 2007 | 2016 | 10 |
| Refinery Area | 8 | Al | 8300 | 871 | 3020 | 7380 | 4250 | 16300 | **0.001** | **Down** | 2009 | 2020 | 12 |
| Refinery Area | 8 | Cd | 0.19 | 0.02 | 0.07 | 0.18 | 0.06 | 0.31 | **0.029** | **Up** | 2008 | 2020 | 13 |
| Refinery Area | 8 | Cr | 51.8 | 2.7 | 10.1 | 53.8 | 32.2 | 72.9 | 0.99 | Down | 2007 | 2020 | 14 |
| Refinery Area | 8 | Cu | 55.5 | 1.68 | 6.29 | 56.9 | 45.3 | 64.2 | 0.338 | Up | 2007 | 2020 | 14 |
| Refinery Area | 8 | Fe | 6320 | 391 | 1350 | 5860 | 4830 | 9160 | 0.1 | Down | 2009 | 2020 | 12 |
| Refinery Area | 8 | Mn | 92.6 | 2.7 | 10.1 | 92.2 | 78.1 | 119 | 0.85 | Up | 2007 | 2020 | 14 |
| Refinery Area | 8 | Ni | 28.5 | 5.29 | 19.8 | 25.8 | 7.64 | 92.6 | 0.277 | Down | 2007 | 2020 | 14 |
| Refinery Area | 8 | Pb | 125 | 12.4 | 46.5 | 115 | 56 | 213 | **0.043** | **Up** | 2007 | 2020 | 14 |
| Refinery Area | 8 | Zn | 67.1 | 3.62 | 13.6 | 64.6 | 48.7 | 92.6 | **0.005** | **Up** | 2007 | 2020 | 14 |
| Gaha | 9 | Al | 3670 | 214 | 604 | 3490 | 3050 | 4780 | 0.131 | Down | 2009 | 2016 | 8 |
| Gaha | 9 | Cd | 0.08 | 0.01 | 0.02 | 0.08 | 0.06 | 0.13 | 0.05 | Down | 2009 | 2016 | 8 |
| Gaha | 9 | Cr | 43.2 | 8.22 | 24.7 | 36.4 | 13.4 | 84 | **0.042** | **Up** | 2007 | 2016 | 9 |
| Gaha | 9 | Cu | 14 | 1.08 | 3.23 | 14.4 | 10 | 20.5 | 0.363 | Up | 2007 | 2016 | 9 |
| Gaha | 9 | Fe | 9280 | 982 | 2780 | 10100 | 4300 | 12500 | 0.746 | Up | 2009 | 2016 | 8 |
| Gaha | 9 | Mn | 44.2 | 6.18 | 18.5 | 44.4 | 12.4 | 79 | **0.009** | **Down** | 2007 | 2016 | 9 |
| Gaha | 9 | Ni | 11.9 | 2.08 | 5.89 | 11.1 | 5.48 | 22.2 | 0.193 | Down | 2009 | 2016 | 8 |
| Gaha | 9 | Pb | 2.17 | 0.24 | 0.71 | 2.24 | 0.98 | 3.29 | 0.761 | Up | 2007 | 2016 | 9 |
| Gaha | 9 | Zn | 20.9 | 2.37 | 6.7 | 20.9 | 11.1 | 34.4 | 0.068 | Down | 2009 | 2016 | 8 |
| Suhain | 10 | Al | 4360 | 774 | 2190 | 4170 | 1600 | 8190 | **0.014** | **Down** | 2009 | 2016 | 8 |
| Suhain | 10 | Cd | 0.09 | 0.02 | 0.05 | 0.08 | 0.01 | 0.17 | 0.089 | Down | 2008 | 2016 | 9 |
| Suhain | 10 | Cr | 80.4 | 42.9 | 136 | 40.9 | 9.64 | 461 | **0.001** | **Up** | 2007 | 2016 | 10 |
| Suhain | 10 | Cu | 24.7 | 3.4 | 10.8 | 29.7 | 1.53 | 37.4 | 0.068 | Up | 2007 | 2016 | 10 |
| Suhain | 10 | Fe | 10200 | 2260 | 6400 | 10000 | 1460 | 24100 | 0.156 | Down | 2009 | 2016 | 8 |
| Suhain | 10 | Mn | 40.6 | 6.16 | 19.5 | 41.9 | 13.6 | 65 | 0.134 | Down | 2007 | 2016 | 10 |
| Suhain | 10 | Ni | 16.1 | 3.59 | 8.8 | 17.7 | 4.34 | 28.4 | 0.068 | Down | 2009 | 2014 | 6 |
| Suhain | 10 | Pb | 8.05 | 3.59 | 11.3 | 3.94 | 0.7 | 37 | **0.043** | **Up** | 2007 | 2016 | 10 |
| Suhain | 10 | Zn | 17.6 | 3.26 | 9.77 | 20.5 | 0.57 | 28.4 | **0.018** | **Down** | 2007 | 2016 | 9 |
| Duwaimil | 11 | Al | 3810 | 312 | 540 | 3930 | 3220 | 4280 | NA | NA | 2013 | 2015 | 3 |
| Duwaimil | 11 | Cd | 0.1 | 0.02 | 0.04 | 0.09 | 0.07 | 0.15 | NA | NA | 2013 | 2015 | 3 |
| Duwaimil | 11 | Cr | 50.7 | 24 | 41.6 | 31.6 | 22.1 | 98.3 | NA | NA | 2013 | 2015 | 3 |
| Duwaimil | 11 | Cu | 38.1 | 13.5 | 23.3 | 33.5 | 17.4 | 63.3 | NA | NA | 2013 | 2015 | 3 |
| Duwaimil | 11 | Fe | 7140 | 951 | 1650 | 7350 | 5400 | 8680 | NA | NA | 2013 | 2015 | 3 |
| Duwaimil | 11 | Mn | 77 | 11 | 19.1 | 74.1 | 59.5 | 97.4 | NA | NA | 2013 | 2015 | 3 |
| Duwaimil | 11 | Ni | 14.6 | 5.71 | 9.9 | 16 | 4.03 | 23.7 | NA | NA | 2013 | 2015 | 3 |
| Duwaimil | 11 | Pb | 5.38 | 1.24 | 2.15 | 4.51 | 3.81 | 7.83 | NA | NA | 2013 | 2015 | 3 |
| Duwaimil | 11 | Zn | 17.5 | 4.16 | 7.21 | 17.8 | 10.2 | 24.6 | NA | NA | 2013 | 2015 | 3 |
| Gazara | 12 | Al | 7350 | 600 | 2080 | 7010 | 4120 | 12000 | **0.042** | **Down** | 2009 | 2020 | 12 |
| Gazara | 12 | Cd | 0.09 | 0.01 | 0.02 | 0.09 | 0.07 | 0.14 | 0.366 | Up | 2009 | 2020 | 12 |
| Gazara | 12 | Cr | 53.7 | 2.03 | 5.75 | 52.1 | 47.7 | 62.4 | 0.125 | Down | 2009 | 2020 | 8 |
| Gazara | 12 | Cu | 11.6 | 0.77 | 2.67 | 11.6 | 7.38 | 15.4 | 0.913 | Down | 2009 | 2020 | 12 |
| Gazara | 12 | Fe | 5950 | 343 | 1190 | 5760 | 3320 | 7990 | 0.338 | Up | 2009 | 2020 | 12 |
| Gazara | 12 | Mn | 91.2 | 5.31 | 18.4 | 98.9 | 51 | 112 | 0.192 | Up | 2009 | 2020 | 12 |
| Gazara | 12 | Ni | 24.5 | 1.79 | 6.2 | 22.4 | 15.5 | 37.4 | 0.547 | Down | 2009 | 2020 | 12 |
| Gazara | 12 | Pb | 2.14 | 0.26 | 0.88 | 2.22 | 0.66 | 3.63 | 1 | Down | 2009 | 2020 | 12 |
| Gazara | 12 | Zn | 21.8 | 1.58 | 5.46 | 21.2 | 14.2 | 36.7 | 0.283 | Down | 2009 | 2020 | 12 |
| Dam | 13 | Al | 6300 | 1020 | 2690 | 5960 | 3240 | 11800 | 0.154 | Up | 2009 | 2016 | 7 |
| Dam | 13 | Cd | 0.13 | 0.03 | 0.07 | 0.1 | 0.05 | 0.25 | **0.038** | **Down** | 2009 | 2016 | 7 |
| Dam | 13 | Cr | 62.3 | 10.3 | 27.2 | 56.5 | 24.6 | 103 | 0.073 | Up | 2009 | 2016 | 7 |
| Dam | 13 | Cu | 22.3 | 3.86 | 10.2 | 15.1 | 13.8 | 37.8 | 0.091 | Down | 2009 | 2016 | 7 |
| Dam | 13 | Fe | 3690 | 540 | 1430 | 3410 | 1530 | 5940 | 0.253 | Up | 2009 | 2016 | 7 |
| Dam | 13 | Mn | 76.5 | 7.99 | 21.1 | 73.8 | 50.1 | 109 | 0.202 | Down | 2009 | 2016 | 7 |
| Dam | 13 | Ni | 24.9 | 3.58 | 9.46 | 25.3 | 12.5 | 36.8 | 0.995 | Up | 2009 | 2016 | 7 |
| Dam | 13 | Pb | 2.61 | 0.49 | 1.28 | 2.39 | 1.69 | 5.37 | 0.37 | Down | 2009 | 2016 | 7 |
| Dam | 13 | Zn | 15.8 | 3.58 | 9.47 | 13.5 | 9.1 | 36.5 | 0.902 | Down | 2009 | 2016 | 7 |
| Jaradah | 14 | Al | 4430 | 391 | 1350 | 4350 | 2170 | 7200 | 0.11 | Down | 2009 | 2020 | 12 |
| Jaradah | 14 | Cd | 0.1 | 0.01 | 0.03 | 0.08 | 0.07 | 0.16 | 0.798 | Down | 2008 | 2020 | 13 |
| Jaradah | 14 | Cr | 57.6 | 14.3 | 53.4 | 41 | 23.7 | 232 | **0.006** | **Down** | 2007 | 2020 | 14 |
| Jaradah | 14 | Cu | 9.2 | 0.88 | 3.3 | 9.62 | 4.2 | 14.6 | 0.231 | Down | 2007 | 2020 | 14 |
| Jaradah | 14 | Fe | 2980 | 224 | 777 | 3050 | 1380 | 4020 | 0.32 | Up | 2009 | 2020 | 12 |
| Jaradah | 14 | Mn | 57.1 | 3.28 | 12.3 | 59.4 | 33.4 | 84.8 | 0.255 | Down | 2007 | 2020 | 14 |
| Jaradah | 14 | Ni | 16.1 | 1.91 | 6.88 | 14 | 8.44 | 34.2 | 0.074 | Down | 2008 | 2020 | 13 |
| Jaradah | 14 | Pb | 1.6 | 0.24 | 0.91 | 1.71 | 0.33 | 3.4 | 0.469 | Down | 2007 | 2020 | 14 |
| Jaradah | 14 | Zn | 12.2 | 2.15 | 8.05 | 12.7 | 1.68 | 33.6 | **0.027** | **Up** | 2007 | 2020 | 14 |
| Jetty 1 | 15 | Al | 9910 | 1930 | 4720 | 8010 | 6530 | 18800 | **0.019** | **Up** | 2009 | 2014 | 6 |
| Jetty 1 | 15 | Cd | 0.15 | 0.02 | 0.05 | 0.16 | 0.06 | 0.2 | 0.786 | Up | 2008 | 2014 | 7 |
| Jetty 1 | 15 | Cr | 41.4 | 5.07 | 14.4 | 39.3 | 19.9 | 69 | 0.716 | Up | 2007 | 2014 | 8 |
| Jetty 1 | 15 | Cu | 50.9 | 4.12 | 11.7 | 46.4 | 43.6 | 77.6 | 0.524 | Up | 2007 | 2014 | 8 |
| Jetty 1 | 15 | Fe | 7120 | 998 | 2440 | 6330 | 5050 | 11800 | 0.58 | Down | 2009 | 2014 | 6 |
| Jetty 1 | 15 | Mn | 103 | 10.5 | 29.7 | 101 | 67.1 | 155 | 0.17 | Down | 2007 | 2014 | 8 |
| Jetty 1 | 15 | Ni | 32.2 | 8.23 | 23.3 | 22.7 | 13.4 | 79.4 | 0.132 | Up | 2007 | 2014 | 8 |
| Jetty 1 | 15 | Pb | 15.3 | 5.48 | 15.5 | 10.1 | 6.66 | 52.8 | 0.215 | Up | 2007 | 2014 | 8 |
| Jetty 1 | 15 | Zn | 81.7 | 2.59 | 7.34 | 80.6 | 72.9 | 92.1 | 0.846 | Up | 2007 | 2014 | 8 |
| Al-Jarim | 16 | Al | 5500 | 470 | 1630 | 5070 | 4050 | 8930 | 0.211 | Down | 2009 | 2020 | 12 |
| Al-Jarim | 16 | Cd | 0.1 | 0.02 | 0.06 | 0.08 | 0.02 | 0.23 | 0.68 | Down | 2008 | 2020 | 13 |
| Al-Jarim | 16 | Cr | 57.4 | 4.42 | 16.5 | 55.6 | 33.4 | 97.6 | **0.001** | **Down** | 2007 | 2020 | 14 |
| Al-Jarim | 16 | Cu | 18.2 | 6.57 | 24.6 | 10.2 | 2.51 | 92 | 0.343 | Down | 2007 | 2020 | 14 |
| Al-Jarim | 16 | Fe | 3670 | 254 | 879 | 3850 | 2120 | 5270 | **0.029** | **Down** | 2009 | 2020 | 12 |
| Al-Jarim | 16 | Mn | 64.8 | 6.97 | 26.1 | 76.7 | 8.13 | 94.3 | **0.014** | **Up** | 2007 | 2020 | 14 |
| Al-Jarim | 16 | Ni | 16.8 | 2.32 | 8.37 | 15.4 | 1.57 | 30.9 | **0.005** | **Down** | 2008 | 2020 | 13 |
| Al-Jarim | 16 | Pb | 1.32 | 0.18 | 0.67 | 1.07 | 0.17 | 2.43 | 0.507 | Up | 2007 | 2020 | 14 |
| Al-Jarim | 16 | Zn | 16.8 | 1.99 | 7.18 | 15.9 | 6.85 | 34.9 | 0.992 | Down | 2008 | 2020 | 13 |
| Khorfasht | 17 | Al | 3130 | 557 | 1580 | 2420 | 1490 | 5400 | 0.078 | Up | 2009 | 2016 | 8 |
| Khorfasht | 17 | Cd | 0.1 | 0.01 | 0.04 | 0.09 | 0.05 | 0.18 | 0.081 | Down | 2008 | 2016 | 9 |
| Khorfasht | 17 | Cr | 144 | 47.3 | 150 | 83 | 15.1 | 499 | 0.092 | Up | 2007 | 2016 | 10 |
| Khorfasht | 17 | Cu | 23.2 | 5.07 | 16 | 22.4 | 3.52 | 52.4 | **0.004** | **Down** | 2007 | 2016 | 10 |
| Khorfasht | 17 | Fe | 1900 | 261 | 737 | 2010 | 873 | 2720 | 0.29 | Down | 2009 | 2016 | 8 |
| Khorfasht | 17 | Mn | 72.1 | 16 | 50.6 | 54.9 | 24.1 | 204 | **0.048** | **Up** | 2007 | 2016 | 10 |
| Khorfasht | 17 | Ni | 14.7 | 3.25 | 9.76 | 11 | 1.74 | 29 | **0.014** | **Down** | 2007 | 2016 | 9 |
| Khorfasht | 17 | Pb | 7.44 | 3.6 | 11.4 | 2.84 | 0.69 | 36.4 | 0.098 | Up | 2007 | 2016 | 10 |
| Khorfasht | 17 | Zn | 18.1 | 3.64 | 10.3 | 15 | 7.48 | 38.4 | **0.005** | **Down** | 2009 | 2016 | 8 |
| Murwada | 18 | Al | 1510 | 304 | 1050 | 995 | 542 | 3940 | 0.313 | Down | 2009 | 2020 | 12 |
| Murwada | 18 | Cd | 0.08 | 0.01 | 0.04 | 0.08 | 0.02 | 0.16 | 0.736 | Down | 2008 | 2020 | 13 |
| Murwada | 18 | Cr | 49.9 | 12.9 | 46.5 | 28.9 | 9.22 | 169 | 0.162 | Down | 2007 | 2020 | 13 |
| Murwada | 18 | Cu | 47.3 | 11.8 | 44.2 | 30.4 | 0.93 | 152 | **0.007** | **Up** | 2007 | 2020 | 14 |
| Murwada | 18 | Fe | 1290 | 165 | 571 | 1040 | 371 | 2260 | 0.844 | Up | 2009 | 2020 | 12 |
| Murwada | 18 | Mn | 27 | 4.03 | 15.1 | 23.4 | 4.16 | 60.3 | 0.074 | Down | 2007 | 2020 | 14 |
| Murwada | 18 | Ni | 14 | 2.95 | 10.2 | 12.9 | 1.5 | 34.5 | 0.959 | Up | 2009 | 2020 | 12 |
| Murwada | 18 | Pb | 6.92 | 2.69 | 10.1 | 3.37 | 0.25 | 34.6 | **0.019** | **Down** | 2007 | 2020 | 14 |
| Murwada | 18 | Zn | 21.5 | 3.49 | 12.6 | 19.5 | 1.45 | 45.5 | **<0.001** | **Up** | 2008 | 2020 | 13 |
| Bartafi | 19 | Al | 4940 | 1380 | 3910 | 3830 | 2310 | 14100 | **0.015** | **Down** | 2009 | 2016 | 8 |
| Bartafi | 19 | Cd | 0.08 | 0.01 | 0.04 | 0.07 | 0.01 | 0.14 | 0.335 | Up | 2008 | 2016 | 9 |
| Bartafi | 19 | Cr | 161 | 47.3 | 150 | 125 | 8.29 | 499 | 0.267 | Up | 2007 | 2016 | 10 |
| Bartafi | 19 | Cu | 18.1 | 3.22 | 10.2 | 17.2 | 0.71 | 38.9 | 0.082 | Up | 2007 | 2016 | 10 |
| Bartafi | 19 | Fe | 2680 | 350 | 990 | 2330 | 1690 | 4600 | 0.764 | Down | 2009 | 2016 | 8 |
| Bartafi | 19 | Mn | 95.1 | 19.9 | 62.9 | 71.4 | 31.3 | 211 | 1 | Down | 2007 | 2016 | 10 |
| Bartafi | 19 | Ni | 12.4 | 1.7 | 5.11 | 13.1 | 5.64 | 20 | **0.004** | **Down** | 2007 | 2016 | 9 |
| Bartafi | 19 | Pb | 6.29 | 2.32 | 7.34 | 3.4 | 0.28 | 19.6 | 0.55 | Up | 2007 | 2016 | 10 |
| Bartafi | 19 | Zn | 15 | 2.86 | 8.57 | 16 | 4.8 | 28.2 | 0.228 | Down | 2007 | 2016 | 9 |
| Gasar | 20 | Al | 10100 | 1510 | 4260 | 8870 | 6390 | 20400 | 0.073 | Up | 2009 | 2016 | 8 |
| Gasar | 20 | Cd | 0.12 | 0.02 | 0.05 | 0.11 | 0.06 | 0.2 | 0.067 | Up | 2008 | 2016 | 9 |
| Gasar | 20 | Cr | 51.8 | 5.59 | 17.7 | 53.5 | 15.9 | 78.2 | **<0.001** | **Up** | 2007 | 2016 | 10 |
| Gasar | 20 | Cu | 15.4 | 1.33 | 4.19 | 16.1 | 8.99 | 21.7 | 0.057 | Up | 2007 | 2016 | 10 |
| Gasar | 20 | Fe | 5660 | 217 | 614 | 5660 | 4820 | 6390 | 0.396 | Up | 2009 | 2016 | 8 |
| Gasar | 20 | Mn | 91.1 | 6.65 | 21 | 94.9 | 35.9 | 109 | **0.028** | **Down** | 2007 | 2016 | 10 |
| Gasar | 20 | Ni | 31.2 | 3.89 | 12.3 | 32.5 | 12.2 | 51.7 | **0.003** | **Down** | 2007 | 2016 | 10 |
| Gasar | 20 | Pb | 2.02 | 0.45 | 1.43 | 1.55 | 0.72 | 4.84 | 0.13 | Up | 2007 | 2016 | 10 |
| Gasar | 20 | Zn | 21.1 | 2.91 | 8.72 | 19.4 | 8.23 | 39.4 | **0.032** | **Down** | 2008 | 2016 | 9 |
| Umm Al-Na'asan | 21 | Al | 10900 | 798 | 2760 | 10700 | 7510 | 18600 | **0.017** | **Down** | 2009 | 2020 | 12 |
| Umm Al-Na'asan | 21 | Cd | 0.08 | 0.01 | 0.03 | 0.07 | 0.04 | 0.13 | 0.629 | Down | 2008 | 2020 | 13 |
| Umm Al-Na'asan | 21 | Cr | 68.4 | 2.14 | 8 | 66.4 | 57.6 | 87.9 | 0.057 | Down | 2007 | 2020 | 14 |
| Umm Al-Na'asan | 21 | Cu | 11 | 0.62 | 2.31 | 11.2 | 7.08 | 15.1 | 0.828 | Down | 2007 | 2020 | 14 |
| Umm Al-Na'asan | 21 | Fe | 6570 | 250 | 867 | 6480 | 5450 | 7830 | 0.294 | Up | 2009 | 2020 | 12 |
| Umm Al-Na'asan | 21 | Mn | 140 | 5.29 | 19.8 | 147 | 107 | 169 | 0.128 | Up | 2007 | 2020 | 14 |
| Umm Al-Na'asan | 21 | Ni | 32.6 | 2.19 | 8.2 | 30.1 | 21.2 | 54.4 | 0.235 | Down | 2007 | 2020 | 14 |
| Umm Al-Na'asan | 21 | Pb | 1.66 | 0.23 | 0.87 | 1.57 | 0.21 | 3.21 | 0.679 | Up | 2007 | 2020 | 14 |
| Umm Al-Na'asan | 21 | Zn | 22.2 | 2.39 | 8.93 | 21.9 | 10.1 | 40.9 | **0.009** | **Up** | 2007 | 2020 | 14 |
| Ya'suf | 22 | Al | 9150 | 1000 | 3480 | 7500 | 5440 | 15700 | 0.078 | Down | 2009 | 2020 | 12 |
| Ya'suf | 22 | Cd | 0.09 | 0.01 | 0.03 | 0.08 | 0.05 | 0.17 | 0.369 | Down | 2008 | 2020 | 13 |
| Ya'suf | 22 | Cr | 118 | 34.7 | 130 | 68.4 | 35.9 | 528 | 0.606 | Down | 2007 | 2020 | 14 |
| Ya'suf | 22 | Cu | 12.1 | 1.74 | 6.52 | 9.71 | 3.76 | 21 | 0.108 | Down | 2007 | 2020 | 14 |
| Ya'suf | 22 | Fe | 5270 | 483 | 1670 | 4900 | 3760 | 9560 | 0.215 | Down | 2009 | 2020 | 12 |
| Ya'suf | 22 | Mn | 117 | 15.7 | 58.7 | 99 | 68.6 | 290 | 0.976 | Down | 2007 | 2020 | 14 |
| Ya'suf | 22 | Ni | 23.7 | 2.33 | 8.73 | 23.7 | 9.17 | 44.9 | **<0.001** | **Down** | 2007 | 2020 | 14 |
| Ya'suf | 22 | Pb | 2.84 | 0.59 | 2.21 | 1.81 | 0.3 | 7.4 | 0.43 | Down | 2007 | 2020 | 14 |
| Ya'suf | 22 | Zn | 19.1 | 2.34 | 8.76 | 20.7 | 0.69 | 37.7 | **<0.001** | **Up** | 2007 | 2020 | 14 |
| Al-Jasra | 23 | Al | 7280 | 631 | 2190 | 6530 | 4870 | 13000 | 0.168 | Down | 2009 | 2020 | 12 |
| Al-Jasra | 23 | Cd | 0.11 | 0.02 | 0.06 | 0.1 | 0.06 | 0.31 | 0.104 | Down | 2008 | 2020 | 13 |
| Al-Jasra | 23 | Cr | 101 | 16.1 | 60.2 | 81.2 | 34.7 | 240 | 0.35 | Down | 2007 | 2020 | 14 |
| Al-Jasra | 23 | Cu | 37.6 | 18.7 | 69.8 | 15.2 | 5.76 | 276 | 0.322 | Up | 2007 | 2020 | 14 |
| Al-Jasra | 23 | Fe | 4420 | 184 | 637 | 4720 | 3280 | 5060 | 0.213 | Down | 2009 | 2020 | 12 |
| Al-Jasra | 23 | Mn | 90.9 | 4.14 | 15.5 | 88 | 68.9 | 121 | **0.042** | **Up** | 2007 | 2020 | 14 |
| Al-Jasra | 23 | Ni | 20.3 | 2.01 | 7.51 | 17.3 | 9.07 | 35.3 | 0.132 | Down | 2007 | 2020 | 14 |
| Al-Jasra | 23 | Pb | 2.7 | 0.54 | 2 | 1.99 | 0.42 | 7.93 | 0.267 | Up | 2007 | 2020 | 14 |
| Al-Jasra | 23 | Zn | 28.6 | 2.08 | 7.78 | 27.6 | 17.2 | 46.6 | **0.047** | **Up** | 2007 | 2020 | 14 |
| Shtaya | 24 | Al | 2580 | 444 | 1090 | 2490 | 1460 | 4600 | 0.894 | Down | 2015 | 2020 | 6 |
| Shtaya | 24 | Cd | 0.07 |  | 0.01 | 0.07 | 0.05 | 0.08 | 0.051 | Up | 2015 | 2020 | 6 |
| Shtaya | 24 | Cr | 60.2 | 10.7 | 26.2 | 69.2 | 24.3 | 89.9 | **0.021** | **Down** | 2015 | 2020 | 6 |
| Shtaya | 24 | Cu | 88.3 | 41.5 | 102 | 65.6 | 7.89 | 280 | 0.059 | Down | 2015 | 2020 | 6 |
| Shtaya | 24 | Fe | 2110 | 106 | 261 | 2110 | 1730 | 2470 | 0.088 | Down | 2015 | 2020 | 6 |
| Shtaya | 24 | Mn | 42.4 | 2.24 | 5.5 | 40.5 | 37.4 | 49.5 | **0.029** | **Up** | 2015 | 2020 | 6 |
| Shtaya | 24 | Ni | 10.3 | 1.74 | 4.26 | 9.84 | 5.82 | 17.1 | 0.953 | Up | 2015 | 2020 | 6 |
| Shtaya | 24 | Pb | 5.51 | 1.27 | 3.1 | 5.58 | 2.27 | 9.1 | 0.511 | Down | 2015 | 2020 | 6 |
| Shtaya | 24 | Zn | 14.8 | 2.44 | 5.99 | 16.1 | 4.79 | 22 | **0.03** | **Down** | 2015 | 2020 | 6 |
| Bulthama | 25 | Al | 6470 | 454 | 642 | 6470 | 6010 | 6920 | NA | NA | 2014 | 2015 | 2 |
| Bulthama | 25 | Cd | 0.12 | 0.02 | 0.03 | 0.12 | 0.1 | 0.14 | NA | NA | 2014 | 2015 | 2 |
| Bulthama | 25 | Cr | 106 | 55 | 77.7 | 106 | 50.8 | 161 | NA | NA | 2014 | 2015 | 2 |
| Bulthama | 25 | Cu | 20.9 | 4.94 | 6.99 | 20.9 | 16 | 25.9 | NA | NA | 2014 | 2015 | 2 |
| Bulthama | 25 | Fe | 3920 | 324 | 458 | 3920 | 3600 | 4250 | NA | NA | 2014 | 2015 | 2 |
| Bulthama | 25 | Mn | 108 | 9.9 | 14 | 108 | 98.4 | 118 | NA | NA | 2014 | 2015 | 2 |
| Bulthama | 25 | Ni | 34 | 7.38 | 10.4 | 34 | 26.7 | 41.4 | NA | NA | 2014 | 2015 | 2 |
| Bulthama | 25 | Pb | 10.9 | 8.82 | 12.5 | 10.9 | 2.03 | 19.7 | NA | NA | 2014 | 2015 | 2 |
| Bulthama | 25 | Zn | 13.2 | 5.44 | 7.7 | 13.2 | 7.71 | 18.6 | NA | NA | 2014 | 2015 | 2 |
| West Jarim | 26 | Al | 3920 | 199 | 345 | 4100 | 3530 | 4140 | NA | NA | 2014 | 2016 | 3 |
| West Jarim | 26 | Cd | 0.08 | 0.02 | 0.03 | 0.07 | 0.05 | 0.1 | NA | NA | 2014 | 2016 | 3 |
| West Jarim | 26 | Cr | 34.4 | 3.99 | 6.9 | 34.7 | 27.4 | 41.2 | NA | NA | 2014 | 2016 | 3 |
| West Jarim | 26 | Cu | 6.48 | 0.07 | 0.12 | 6.44 | 6.38 | 6.61 | NA | NA | 2014 | 2016 | 3 |
| West Jarim | 26 | Fe | 2890 | 438 | 758 | 2850 | 2140 | 3660 | NA | NA | 2014 | 2016 | 3 |
| West Jarim | 26 | Mn | 64.3 | 4.19 | 7.26 | 61.4 | 59 | 72.6 | NA | NA | 2014 | 2016 | 3 |
| West Jarim | 26 | Ni | 14.8 | 3.58 | 6.2 | 17.9 | 7.67 | 18.9 | NA | NA | 2014 | 2016 | 3 |
| West Jarim | 26 | Pb | 1.05 | 0.2 | 0.34 | 1.12 | 0.67 | 1.34 | NA | NA | 2014 | 2016 | 3 |
| West Jarim | 26 | Zn | 16 | 1.55 | 2.68 | 14.5 | 14.4 | 19.1 | NA | NA | 2014 | 2016 | 3 |
| Jetty 2 | 27 | Al | 7070 |  |  | 7070 | 7070 | 7070 | NA | NA | 2015 | 2015 | 1 |
| Jetty 2 | 27 | Cd | 0.14 |  |  | 0.14 | 0.14 | 0.14 | NA | NA | 2015 | 2015 | 1 |
| Jetty 2 | 27 | Cr | 140 |  |  | 140 | 140 | 140 | NA | NA | 2015 | 2015 | 1 |
| Jetty 2 | 27 | Cu | 54 |  |  | 54 | 54 | 54 | NA | NA | 2015 | 2015 | 1 |
| Jetty 2 | 27 | Fe | 9090 |  |  | 9090 | 9090 | 9090 | NA | NA | 2015 | 2015 | 1 |
| Jetty 2 | 27 | Mn | 126 |  |  | 126 | 126 | 126 | NA | NA | 2015 | 2015 | 1 |
| Jetty 2 | 27 | Ni | 17.3 |  |  | 17.3 | 17.3 | 17.3 | NA | NA | 2015 | 2015 | 1 |
| Jetty 2 | 27 | Pb | 4 |  |  | 4 | 4 | 4 | NA | NA | 2015 | 2015 | 1 |
| Jetty 2 | 27 | Zn | 78.9 |  |  | 78.9 | 78.9 | 78.9 | NA | NA | 2015 | 2015 | 1 |
| Jetty 3 | 28 | Al | 6380 | 347 | 776 | 6550 | 5080 | 7140 | 0.765 | Up | 2016 | 2020 | 5 |
| Jetty 3 | 28 | Cd | 0.12 | 0.01 | 0.03 | 0.13 | 0.07 | 0.16 | **0.041** | **Down** | 2016 | 2020 | 5 |
| Jetty 3 | 28 | Cr | 71.4 | 4.28 | 9.57 | 71.7 | 57.6 | 81.9 | **0.011** | **Down** | 2016 | 2020 | 5 |
| Jetty 3 | 28 | Cu | 30.3 | 5.64 | 12.6 | 27.3 | 21.2 | 52.2 | 0.052 | Up | 2016 | 2020 | 5 |
| Jetty 3 | 28 | Fe | 5110 | 348 | 777 | 5380 | 3960 | 5960 | **0.036** | **Up** | 2016 | 2020 | 5 |
| Jetty 3 | 28 | Mn | 114 | 6.06 | 13.5 | 122 | 93.4 | 126 | 0.139 | Up | 2016 | 2020 | 5 |
| Jetty 3 | 28 | Ni | 19.2 | 1.53 | 3.42 | 18 | 17.1 | 25.2 | 0.423 | Down | 2016 | 2020 | 5 |
| Jetty 3 | 28 | Pb | 2.75 | 0.75 | 1.69 | 2.09 | 1.79 | 5.75 | 0.573 | Up | 2016 | 2020 | 5 |
| Jetty 3 | 28 | Zn | 47 | 3.34 | 7.47 | 49.8 | 35.9 | 53.8 | **0.045** | **Down** | 2016 | 2020 | 5 |
| Wharf Area | 29 | Al | 4670 | 226 | 451 | 4840 | 4000 | 4990 | 0.308 | Up | 2017 | 2020 | 4 |
| Wharf Area | 29 | Cd | 0.07 |  | 0.01 | 0.08 | 0.06 | 0.08 | 0.983 | Down | 2017 | 2020 | 4 |
| Wharf Area | 29 | Cr | 63.3 | 17.4 | 34.8 | 48.1 | 41.9 | 115 | 0.879 | Up | 2017 | 2020 | 4 |
| Wharf Area | 29 | Cu | 27.8 | 3.59 | 7.18 | 24.9 | 22.9 | 38.5 | 0.887 | Down | 2017 | 2020 | 4 |
| Wharf Area | 29 | Fe | 18500 | 2080 | 4170 | 19000 | 13100 | 23100 | 0.558 | Up | 2017 | 2020 | 4 |
| Wharf Area | 29 | Mn | 86.2 | 5.16 | 10.3 | 87.4 | 72.8 | 97.2 | 0.497 | Up | 2017 | 2020 | 4 |
| Wharf Area | 29 | Ni | 22.8 | 6.69 | 13.4 | 18.4 | 12.9 | 41.5 | 0.846 | Down | 2017 | 2020 | 4 |
| Wharf Area | 29 | Pb | 3.22 | 1.03 | 2.05 | 2.38 | 1.85 | 6.28 | 0.717 | Up | 2017 | 2020 | 4 |
| Wharf Area | 29 | Zn | 29.5 | 0.82 | 1.64 | 29.4 | 27.6 | 31.6 | 0.363 | Down | 2017 | 2020 | 4 |

*MEC is the Measured Environmental Concentrations in mg kg^-1^; p-Value for the trend analysis at the 95% confidence interval (bold p-Values highlight statistically significant trends, a minimum of 4 years needed for a linear trend analysis, and 5 years for a GAM analysis); SE Mean is the Standard Error around the mean; St Dev is the Standard Deviation around the mean; Min is the lowest measured concentration at this station over all sampled years; Max is the highest measured concentration at this station over all sampled years; N is the sample number of a specific determinant and the number of years samples

Table S5. Measured Environmental Concentration and the associated Risk Characterisation Ratio in response to the internationally recognised assessment criteria

| Station | Site No. | Year | MEC (mg kg^-1^) | Determinant | BAC RCR | BAC RCR>1 | ERL RCR | ERL RCR>1 | ERM RCR | ERM RCR>1 | ISQG RCR | ISQG RCR>1 | PEL RCR | PEL RCR>1 |
| --- | --- | --- | --- | --- | --- | --- | --- | --- | --- | --- | --- | --- | --- | --- |
| Noon | 1 | 2016 | 0.093 | Cd | 0.3 | FALSE | 0.08 | FALSE | 0.01 | FALSE | 0.13 | FALSE | 0.02 | FALSE |
| Noon | 1 | 2016 | 435 | Cr | 5.37 | TRUE | 5.37 | TRUE | 1.18 | TRUE | 8.31 | TRUE | 2.72 | TRUE |
| Noon | 1 | 2016 | 16.1 | Cu | 0.6 | FALSE | 0.47 | FALSE | 0.06 | FALSE | 0.86 | FALSE | 0.15 | FALSE |
| Noon | 1 | 2016 | 8.1 | Ni | 0.23 | FALSE | 0.39 | FALSE | 0.16 | FALSE | NA | NA | NA | NA |
| Noon | 1 | 2016 | 1.9 | Pb | 0.05 | FALSE | 0.04 | FALSE | 0.01 | FALSE | 0.06 | FALSE | 0.02 | FALSE |
| Noon | 1 | 2016 | 9.1 | Zn | 0.07 | FALSE | 0.06 | FALSE | 0.02 | FALSE | 0.07 | FALSE | 0.03 | FALSE |
| Noon | 1 | 2015 | 0.099 | Cd | 0.32 | FALSE | 0.08 | FALSE | 0.01 | FALSE | 0.14 | FALSE | 0.02 | FALSE |
| Noon | 1 | 2015 | 426 | Cr | 5.26 | TRUE | 5.26 | TRUE | 1.15 | TRUE | 8.15 | TRUE | 2.66 | TRUE |
| Noon | 1 | 2015 | 15.5 | Cu | 0.57 | FALSE | 0.46 | FALSE | 0.06 | FALSE | 0.83 | FALSE | 0.14 | FALSE |
| Noon | 1 | 2015 | 35.2 | Ni | 0.98 | FALSE | 1.68 | TRUE | 0.68 | FALSE | NA | NA | NA | NA |
| Noon | 1 | 2015 | 4.96 | Pb | 0.13 | FALSE | 0.11 | FALSE | 0.02 | FALSE | 0.16 | FALSE | 0.04 | FALSE |
| Noon | 1 | 2015 | 14 | Zn | 0.11 | FALSE | 0.09 | FALSE | 0.03 | FALSE | 0.11 | FALSE | 0.05 | FALSE |
| Noon | 1 | 2014 | 0.1 | Cd | 0.32 | FALSE | 0.08 | FALSE | 0.01 | FALSE | 0.14 | FALSE | 0.02 | FALSE |
| Noon | 1 | 2014 | 54.7 | Cr | 0.68 | FALSE | 0.68 | FALSE | 0.15 | FALSE | 1.05 | TRUE | 0.34 | FALSE |
| Noon | 1 | 2014 | 16.6 | Cu | 0.61 | FALSE | 0.49 | FALSE | 0.06 | FALSE | 0.89 | FALSE | 0.15 | FALSE |
| Noon | 1 | 2014 | 22.8 | Ni | 0.63 | FALSE | 1.09 | TRUE | 0.44 | FALSE | NA | NA | NA | NA |
| Noon | 1 | 2014 | 2.02 | Pb | 0.05 | FALSE | 0.04 | FALSE | 0.01 | FALSE | 0.07 | FALSE | 0.02 | FALSE |
| Noon | 1 | 2014 | 18.8 | Zn | 0.15 | FALSE | 0.13 | FALSE | 0.05 | FALSE | 0.15 | FALSE | 0.07 | FALSE |
| Noon | 1 | 2013 | 0.123 | Cd | 0.4 | FALSE | 0.1 | FALSE | 0.01 | FALSE | 0.18 | FALSE | 0.03 | FALSE |
| Noon | 1 | 2013 | 57 | Cr | 0.7 | FALSE | 0.7 | FALSE | 0.15 | FALSE | 1.09 | TRUE | 0.36 | FALSE |
| Noon | 1 | 2013 | 12.9 | Cu | 0.48 | FALSE | 0.38 | FALSE | 0.05 | FALSE | 0.69 | FALSE | 0.12 | FALSE |
| Noon | 1 | 2013 | 31.1 | Ni | 0.86 | FALSE | 1.49 | TRUE | 0.6 | FALSE | NA | NA | NA | NA |
| Noon | 1 | 2013 | 5.69 | Pb | 0.15 | FALSE | 0.12 | FALSE | 0.03 | FALSE | 0.19 | FALSE | 0.05 | FALSE |
| Noon | 1 | 2013 | 20.2 | Zn | 0.17 | FALSE | 0.13 | FALSE | 0.05 | FALSE | 0.16 | FALSE | 0.07 | FALSE |
| Noon | 1 | 2012 | 0.156 | Cd | 0.5 | FALSE | 0.13 | FALSE | 0.02 | FALSE | 0.22 | FALSE | 0.04 | FALSE |
| Noon | 1 | 2012 | 79.3 | Cr | 0.98 | FALSE | 0.98 | FALSE | 0.21 | FALSE | 1.52 | TRUE | 0.5 | FALSE |
| Noon | 1 | 2012 | 12.1 | Cu | 0.45 | FALSE | 0.36 | FALSE | 0.04 | FALSE | 0.65 | FALSE | 0.11 | FALSE |
| Noon | 1 | 2012 | 35.8 | Ni | 0.99 | FALSE | 1.71 | TRUE | 0.69 | FALSE | NA | NA | NA | NA |
| Noon | 1 | 2012 | 4.84 | Pb | 0.13 | FALSE | 0.1 | FALSE | 0.02 | FALSE | 0.16 | FALSE | 0.04 | FALSE |
| Noon | 1 | 2012 | 35.8 | Zn | 0.29 | FALSE | 0.24 | FALSE | 0.09 | FALSE | 0.29 | FALSE | 0.13 | FALSE |
| Noon | 1 | 2011 | 0.096 | Cd | 0.31 | FALSE | 0.08 | FALSE | 0.01 | FALSE | 0.14 | FALSE | 0.02 | FALSE |
| Noon | 1 | 2011 | 240 | Cr | 2.96 | TRUE | 2.96 | TRUE | 0.65 | FALSE | 4.58 | TRUE | 1.5 | TRUE |
| Noon | 1 | 2011 | 19.1 | Cu | 0.71 | FALSE | 0.56 | FALSE | 0.07 | FALSE | 1.02 | TRUE | 0.18 | FALSE |
| Noon | 1 | 2011 | 33.2 | Ni | 0.92 | FALSE | 1.59 | TRUE | 0.64 | FALSE | NA | NA | NA | NA |
| Noon | 1 | 2011 | 0.92 | Pb | 0.02 | FALSE | 0.02 | FALSE | 0 | FALSE | 0.03 | FALSE | 0.01 | FALSE |
| Noon | 1 | 2011 | 39.5 | Zn | 0.32 | FALSE | 0.26 | FALSE | 0.1 | FALSE | 0.32 | FALSE | 0.15 | FALSE |
| Noon | 1 | 2010 | 0.095 | Cd | 0.31 | FALSE | 0.08 | FALSE | 0.01 | FALSE | 0.14 | FALSE | 0.02 | FALSE |
| Noon | 1 | 2010 | 73.7 | Cr | 0.91 | FALSE | 0.91 | FALSE | 0.2 | FALSE | 1.41 | TRUE | 0.46 | FALSE |
| Noon | 1 | 2010 | 17.6 | Cu | 0.65 | FALSE | 0.52 | FALSE | 0.07 | FALSE | 0.94 | FALSE | 0.16 | FALSE |
| Noon | 1 | 2010 | 26.3 | Ni | 0.73 | FALSE | 1.26 | TRUE | 0.51 | FALSE | NA | NA | NA | NA |
| Noon | 1 | 2010 | 2.34 | Pb | 0.06 | FALSE | 0.05 | FALSE | 0.01 | FALSE | 0.08 | FALSE | 0.02 | FALSE |
| Noon | 1 | 2010 | 17.7 | Zn | 0.14 | FALSE | 0.12 | FALSE | 0.04 | FALSE | 0.14 | FALSE | 0.07 | FALSE |
| Noon | 1 | 2009 | 0.131 | Cd | 0.42 | FALSE | 0.11 | FALSE | 0.01 | FALSE | 0.19 | FALSE | 0.03 | FALSE |
| Noon | 1 | 2009 | 70.4 | Cr | 0.87 | FALSE | 0.87 | FALSE | 0.19 | FALSE | 1.35 | TRUE | 0.44 | FALSE |
| Noon | 1 | 2009 | 12.5 | Cu | 0.46 | FALSE | 0.37 | FALSE | 0.05 | FALSE | 0.67 | FALSE | 0.12 | FALSE |
| Noon | 1 | 2009 | 21.5 | Ni | 0.6 | FALSE | 1.03 | TRUE | 0.42 | FALSE | NA | NA | NA | NA |
| Noon | 1 | 2009 | 2.56 | Pb | 0.07 | FALSE | 0.05 | FALSE | 0.01 | FALSE | 0.08 | FALSE | 0.02 | FALSE |
| Noon | 1 | 2009 | 10.9 | Zn | 0.09 | FALSE | 0.07 | FALSE | 0.03 | FALSE | 0.09 | FALSE | 0.04 | FALSE |
| Noon | 1 | 2008 | 0.247 | Cd | 0.8 | FALSE | 0.21 | FALSE | 0.03 | FALSE | 0.35 | FALSE | 0.06 | FALSE |
| Noon | 1 | 2008 | 56.8 | Cr | 0.7 | FALSE | 0.7 | FALSE | 0.15 | FALSE | 1.09 | TRUE | 0.35 | FALSE |
| Noon | 1 | 2008 | 9.1 | Cu | 0.34 | FALSE | 0.27 | FALSE | 0.03 | FALSE | 0.49 | FALSE | 0.08 | FALSE |
| Noon | 1 | 2008 | 20.5 | Ni | 0.57 | FALSE | 0.98 | FALSE | 0.4 | FALSE | NA | NA | NA | NA |
| Noon | 1 | 2008 | 2.61 | Pb | 0.07 | FALSE | 0.06 | FALSE | 0.01 | FALSE | 0.09 | FALSE | 0.02 | FALSE |
| Noon | 1 | 2008 | 10.1 | Zn | 0.08 | FALSE | 0.07 | FALSE | 0.02 | FALSE | 0.08 | FALSE | 0.04 | FALSE |
| Noon | 1 | 2007 | 49.3 | Cr | 0.61 | FALSE | 0.61 | FALSE | 0.13 | FALSE | 0.94 | FALSE | 0.31 | FALSE |
| Noon | 1 | 2007 | 26 | Cu | 0.96 | FALSE | 0.76 | FALSE | 0.1 | FALSE | 1.39 | TRUE | 0.24 | FALSE |
| Noon | 1 | 2007 | 30 | Ni | 0.83 | FALSE | 1.43 | TRUE | 0.58 | FALSE | NA | NA | NA | NA |
| Noon | 1 | 2007 | 3.35 | Pb | 0.09 | FALSE | 0.07 | FALSE | 0.02 | FALSE | 0.11 | FALSE | 0.03 | FALSE |
| Noon | 1 | 2007 | 24.8 | Zn | 0.2 | FALSE | 0.17 | FALSE | 0.06 | FALSE | 0.2 | FALSE | 0.09 | FALSE |
| Mashtan | 2 | 2020 | 0.107 | Cd | 0.35 | FALSE | 0.09 | FALSE | 0.01 | FALSE | 0.15 | FALSE | 0.03 | FALSE |
| Mashtan | 2 | 2020 | 47.4 | Cr | 0.59 | FALSE | 0.59 | FALSE | 0.13 | FALSE | 0.91 | FALSE | 0.3 | FALSE |
| Mashtan | 2 | 2020 | 82.6 | Cu | 3.06 | TRUE | 2.43 | TRUE | 0.31 | FALSE | 4.41 | TRUE | 0.76 | FALSE |
| Mashtan | 2 | 2020 | 13.8 | Ni | 0.38 | FALSE | 0.66 | FALSE | 0.27 | FALSE | NA | NA | NA | NA |
| Mashtan | 2 | 2020 | 1.42 | Pb | 0.04 | FALSE | 0.03 | FALSE | 0.01 | FALSE | 0.05 | FALSE | 0.01 | FALSE |
| Mashtan | 2 | 2020 | 20.5 | Zn | 0.17 | FALSE | 0.14 | FALSE | 0.05 | FALSE | 0.17 | FALSE | 0.08 | FALSE |
| Mashtan | 2 | 2019 | 0.066 | Cd | 0.21 | FALSE | 0.06 | FALSE | 0.01 | FALSE | 0.09 | FALSE | 0.02 | FALSE |
| Mashtan | 2 | 2019 | 140 | Cr | 1.73 | TRUE | 1.73 | TRUE | 0.38 | FALSE | 2.67 | TRUE | 0.87 | FALSE |
| Mashtan | 2 | 2019 | 37.3 | Cu | 1.38 | TRUE | 1.1 | TRUE | 0.14 | FALSE | 1.99 | TRUE | 0.35 | FALSE |
| Mashtan | 2 | 2019 | 15.7 | Ni | 0.44 | FALSE | 0.75 | FALSE | 0.3 | FALSE | NA | NA | NA | NA |
| Mashtan | 2 | 2019 | 7.74 | Pb | 0.2 | FALSE | 0.17 | FALSE | 0.04 | FALSE | 0.26 | FALSE | 0.07 | FALSE |
| Mashtan | 2 | 2019 | 20.4 | Zn | 0.17 | FALSE | 0.14 | FALSE | 0.05 | FALSE | 0.16 | FALSE | 0.08 | FALSE |
| Mashtan | 2 | 2018 | 0.077 | Cd | 0.25 | FALSE | 0.06 | FALSE | 0.01 | FALSE | 0.11 | FALSE | 0.02 | FALSE |
| Mashtan | 2 | 2018 | 280 | Cr | 3.45 | TRUE | 3.45 | TRUE | 0.76 | FALSE | 5.34 | TRUE | 1.75 | TRUE |
| Mashtan | 2 | 2018 | 41 | Cu | 1.52 | TRUE | 1.2 | TRUE | 0.15 | FALSE | 2.19 | TRUE | 0.38 | FALSE |
| Mashtan | 2 | 2018 | 11.8 | Ni | 0.33 | FALSE | 0.56 | FALSE | 0.23 | FALSE | NA | NA | NA | NA |
| Mashtan | 2 | 2018 | 7.95 | Pb | 0.21 | FALSE | 0.17 | FALSE | 0.04 | FALSE | 0.26 | FALSE | 0.07 | FALSE |
| Mashtan | 2 | 2018 | 19 | Zn | 0.16 | FALSE | 0.13 | FALSE | 0.05 | FALSE | 0.15 | FALSE | 0.07 | FALSE |
| Mashtan | 2 | 2017 | 0.119 | Cd | 0.38 | FALSE | 0.1 | FALSE | 0.01 | FALSE | 0.17 | FALSE | 0.03 | FALSE |
| Mashtan | 2 | 2017 | 53.8 | Cr | 0.66 | FALSE | 0.66 | FALSE | 0.15 | FALSE | 1.03 | TRUE | 0.34 | FALSE |
| Mashtan | 2 | 2017 | 37.5 | Cu | 1.39 | TRUE | 1.1 | TRUE | 0.14 | FALSE | 2 | TRUE | 0.35 | FALSE |
| Mashtan | 2 | 2017 | 13.5 | Ni | 0.37 | FALSE | 0.64 | FALSE | 0.26 | FALSE | NA | NA | NA | NA |
| Mashtan | 2 | 2017 | 1.45 | Pb | 0.04 | FALSE | 0.03 | FALSE | 0.01 | FALSE | 0.05 | FALSE | 0.01 | FALSE |
| Mashtan | 2 | 2017 | 13.6 | Zn | 0.11 | FALSE | 0.09 | FALSE | 0.03 | FALSE | 0.11 | FALSE | 0.05 | FALSE |
| Mashtan | 2 | 2016 | 0.115 | Cd | 0.37 | FALSE | 0.1 | FALSE | 0.01 | FALSE | 0.16 | FALSE | 0.03 | FALSE |
| Mashtan | 2 | 2016 | 49.2 | Cr | 0.61 | FALSE | 0.61 | FALSE | 0.13 | FALSE | 0.94 | FALSE | 0.31 | FALSE |
| Mashtan | 2 | 2016 | 22.3 | Cu | 0.83 | FALSE | 0.66 | FALSE | 0.08 | FALSE | 1.19 | TRUE | 0.21 | FALSE |
| Mashtan | 2 | 2016 | 18.9 | Ni | 0.52 | FALSE | 0.9 | FALSE | 0.37 | FALSE | NA | NA | NA | NA |
| Mashtan | 2 | 2016 | 2.02 | Pb | 0.05 | FALSE | 0.04 | FALSE | 0.01 | FALSE | 0.07 | FALSE | 0.02 | FALSE |
| Mashtan | 2 | 2016 | 23.6 | Zn | 0.19 | FALSE | 0.16 | FALSE | 0.06 | FALSE | 0.19 | FALSE | 0.09 | FALSE |
| Mashtan | 2 | 2015 | 0.13 | Cd | 0.42 | FALSE | 0.11 | FALSE | 0.01 | FALSE | 0.19 | FALSE | 0.03 | FALSE |
| Mashtan | 2 | 2015 | 56.1 | Cr | 0.69 | FALSE | 0.69 | FALSE | 0.15 | FALSE | 1.07 | TRUE | 0.35 | FALSE |
| Mashtan | 2 | 2015 | 22.4 | Cu | 0.83 | FALSE | 0.66 | FALSE | 0.08 | FALSE | 1.2 | TRUE | 0.21 | FALSE |
| Mashtan | 2 | 2015 | 16.5 | Ni | 0.46 | FALSE | 0.79 | FALSE | 0.32 | FALSE | NA | NA | NA | NA |
| Mashtan | 2 | 2015 | 5.37 | Pb | 0.14 | FALSE | 0.12 | FALSE | 0.02 | FALSE | 0.18 | FALSE | 0.05 | FALSE |
| Mashtan | 2 | 2015 | 11.2 | Zn | 0.09 | FALSE | 0.07 | FALSE | 0.03 | FALSE | 0.09 | FALSE | 0.04 | FALSE |
| Mashtan | 2 | 2014 | 0.13 | Cd | 0.42 | FALSE | 0.11 | FALSE | 0.01 | FALSE | 0.19 | FALSE | 0.03 | FALSE |
| Mashtan | 2 | 2014 | 49.9 | Cr | 0.62 | FALSE | 0.62 | FALSE | 0.13 | FALSE | 0.95 | FALSE | 0.31 | FALSE |
| Mashtan | 2 | 2014 | 13.2 | Cu | 0.49 | FALSE | 0.39 | FALSE | 0.05 | FALSE | 0.71 | FALSE | 0.12 | FALSE |
| Mashtan | 2 | 2014 | 16.6 | Ni | 0.46 | FALSE | 0.8 | FALSE | 0.32 | FALSE | NA | NA | NA | NA |
| Mashtan | 2 | 2014 | 1.97 | Pb | 0.05 | FALSE | 0.04 | FALSE | 0.01 | FALSE | 0.07 | FALSE | 0.02 | FALSE |
| Mashtan | 2 | 2014 | 18.8 | Zn | 0.15 | FALSE | 0.13 | FALSE | 0.05 | FALSE | 0.15 | FALSE | 0.07 | FALSE |
| Mashtan | 2 | 2013 | 0.096 | Cd | 0.31 | FALSE | 0.08 | FALSE | 0.01 | FALSE | 0.14 | FALSE | 0.02 | FALSE |
| Mashtan | 2 | 2013 | 201 | Cr | 2.48 | TRUE | 2.48 | TRUE | 0.54 | FALSE | 3.84 | TRUE | 1.25 | TRUE |
| Mashtan | 2 | 2013 | 13.9 | Cu | 0.51 | FALSE | 0.41 | FALSE | 0.05 | FALSE | 0.74 | FALSE | 0.13 | FALSE |
| Mashtan | 2 | 2013 | 16.1 | Ni | 0.45 | FALSE | 0.77 | FALSE | 0.31 | FALSE | NA | NA | NA | NA |
| Mashtan | 2 | 2013 | 4.59 | Pb | 0.12 | FALSE | 0.1 | FALSE | 0.02 | FALSE | 0.15 | FALSE | 0.04 | FALSE |
| Mashtan | 2 | 2013 | 14.3 | Zn | 0.12 | FALSE | 0.1 | FALSE | 0.03 | FALSE | 0.12 | FALSE | 0.05 | FALSE |
| Mashtan | 2 | 2012 | 0.144 | Cd | 0.46 | FALSE | 0.12 | FALSE | 0.01 | FALSE | 0.21 | FALSE | 0.03 | FALSE |
| Mashtan | 2 | 2012 | 60.7 | Cr | 0.75 | FALSE | 0.75 | FALSE | 0.16 | FALSE | 1.16 | TRUE | 0.38 | FALSE |
| Mashtan | 2 | 2012 | 28.5 | Cu | 1.05 | TRUE | 0.84 | FALSE | 0.11 | FALSE | 1.52 | TRUE | 0.26 | FALSE |
| Mashtan | 2 | 2012 | 26 | Ni | 0.72 | FALSE | 1.24 | TRUE | 0.5 | FALSE | NA | NA | NA | NA |
| Mashtan | 2 | 2012 | 6.78 | Pb | 0.18 | FALSE | 0.15 | FALSE | 0.03 | FALSE | 0.22 | FALSE | 0.06 | FALSE |
| Mashtan | 2 | 2012 | 26 | Zn | 0.21 | FALSE | 0.17 | FALSE | 0.06 | FALSE | 0.21 | FALSE | 0.1 | FALSE |
| Mashtan | 2 | 2011 | 0.139 | Cd | 0.45 | FALSE | 0.12 | FALSE | 0.01 | FALSE | 0.2 | FALSE | 0.03 | FALSE |
| Mashtan | 2 | 2011 | 84 | Cr | 1.04 | TRUE | 1.04 | TRUE | 0.23 | FALSE | 1.61 | TRUE | 0.52 | FALSE |
| Mashtan | 2 | 2011 | 18.3 | Cu | 0.68 | FALSE | 0.54 | FALSE | 0.07 | FALSE | 0.98 | FALSE | 0.17 | FALSE |
| Mashtan | 2 | 2011 | 32 | Ni | 0.89 | FALSE | 1.53 | TRUE | 0.62 | FALSE | NA | NA | NA | NA |
| Mashtan | 2 | 2011 | 1.42 | Pb | 0.04 | FALSE | 0.03 | FALSE | 0.01 | FALSE | 0.05 | FALSE | 0.01 | FALSE |
| Mashtan | 2 | 2011 | 42.4 | Zn | 0.35 | FALSE | 0.28 | FALSE | 0.1 | FALSE | 0.34 | FALSE | 0.16 | FALSE |
| Mashtan | 2 | 2010 | 0.105 | Cd | 0.34 | FALSE | 0.09 | FALSE | 0.01 | FALSE | 0.15 | FALSE | 0.02 | FALSE |
| Mashtan | 2 | 2010 | 70.4 | Cr | 0.87 | FALSE | 0.87 | FALSE | 0.19 | FALSE | 1.35 | TRUE | 0.44 | FALSE |
| Mashtan | 2 | 2010 | 15.5 | Cu | 0.57 | FALSE | 0.46 | FALSE | 0.06 | FALSE | 0.83 | FALSE | 0.14 | FALSE |
| Mashtan | 2 | 2010 | 20.3 | Ni | 0.56 | FALSE | 0.97 | FALSE | 0.39 | FALSE | NA | NA | NA | NA |
| Mashtan | 2 | 2010 | 1.65 | Pb | 0.04 | FALSE | 0.04 | FALSE | 0.01 | FALSE | 0.05 | FALSE | 0.01 | FALSE |
| Mashtan | 2 | 2010 | 15.7 | Zn | 0.13 | FALSE | 0.1 | FALSE | 0.04 | FALSE | 0.13 | FALSE | 0.06 | FALSE |
| Mashtan | 2 | 2009 | 0.171 | Cd | 0.55 | FALSE | 0.14 | FALSE | 0.02 | FALSE | 0.24 | FALSE | 0.04 | FALSE |
| Mashtan | 2 | 2009 | 171 | Cr | 2.11 | TRUE | 2.11 | TRUE | 0.46 | FALSE | 3.27 | TRUE | 1.07 | TRUE |
| Mashtan | 2 | 2009 | 7.2 | Cu | 0.27 | FALSE | 0.21 | FALSE | 0.03 | FALSE | 0.38 | FALSE | 0.07 | FALSE |
| Mashtan | 2 | 2009 | 20.2 | Ni | 0.56 | FALSE | 0.97 | FALSE | 0.39 | FALSE | NA | NA | NA | NA |
| Mashtan | 2 | 2009 | 2.61 | Pb | 0.07 | FALSE | 0.06 | FALSE | 0.01 | FALSE | 0.09 | FALSE | 0.02 | FALSE |
| Mashtan | 2 | 2009 | 12.4 | Zn | 0.1 | FALSE | 0.08 | FALSE | 0.03 | FALSE | 0.1 | FALSE | 0.05 | FALSE |
| Mashtan | 2 | 2008 | 0.317 | Cd | 1.02 | TRUE | 0.26 | FALSE | 0.03 | FALSE | 0.45 | FALSE | 0.08 | FALSE |
| Mashtan | 2 | 2008 | 100 | Cr | 1.24 | TRUE | 1.24 | TRUE | 0.27 | FALSE | 1.92 | TRUE | 0.63 | FALSE |
| Mashtan | 2 | 2008 | 8.7 | Cu | 0.32 | FALSE | 0.26 | FALSE | 0.03 | FALSE | 0.47 | FALSE | 0.08 | FALSE |
| Mashtan | 2 | 2008 | 20.4 | Ni | 0.57 | FALSE | 0.98 | FALSE | 0.4 | FALSE | NA | NA | NA | NA |
| Mashtan | 2 | 2008 | 4.56 | Pb | 0.12 | FALSE | 0.1 | FALSE | 0.02 | FALSE | 0.15 | FALSE | 0.04 | FALSE |
| Mashtan | 2 | 2008 | 14.8 | Zn | 0.12 | FALSE | 0.1 | FALSE | 0.04 | FALSE | 0.12 | FALSE | 0.05 | FALSE |
| Mashtan | 2 | 2007 | 117 | Cr | 1.45 | TRUE | 1.45 | TRUE | 0.32 | FALSE | 2.24 | TRUE | 0.73 | FALSE |
| Mashtan | 2 | 2007 | 10.4 | Cu | 0.39 | FALSE | 0.31 | FALSE | 0.04 | FALSE | 0.56 | FALSE | 0.1 | FALSE |
| Mashtan | 2 | 2007 | 15.4 | Ni | 0.43 | FALSE | 0.74 | FALSE | 0.3 | FALSE | NA | NA | NA | NA |
| Mashtan | 2 | 2007 | 2.57 | Pb | 0.07 | FALSE | 0.05 | FALSE | 0.01 | FALSE | 0.09 | FALSE | 0.02 | FALSE |
| Mashtan | 2 | 2007 | 4.5 | Zn | 0.04 | FALSE | 0.03 | FALSE | 0.01 | FALSE | 0.04 | FALSE | 0.02 | FALSE |
| Jabari | 3 | 2016 | 0.093 | Cd | 0.3 | FALSE | 0.08 | FALSE | 0.01 | FALSE | 0.13 | FALSE | 0.02 | FALSE |
| Jabari | 3 | 2016 | 41.3 | Cr | 0.51 | FALSE | 0.51 | FALSE | 0.11 | FALSE | 0.79 | FALSE | 0.26 | FALSE |
| Jabari | 3 | 2016 | 17.6 | Cu | 0.65 | FALSE | 0.52 | FALSE | 0.07 | FALSE | 0.94 | FALSE | 0.16 | FALSE |
| Jabari | 3 | 2016 | 22.5 | Ni | 0.62 | FALSE | 1.08 | TRUE | 0.44 | FALSE | NA | NA | NA | NA |
| Jabari | 3 | 2016 | 2.83 | Pb | 0.07 | FALSE | 0.06 | FALSE | 0.01 | FALSE | 0.09 | FALSE | 0.03 | FALSE |
| Jabari | 3 | 2016 | 25.4 | Zn | 0.21 | FALSE | 0.17 | FALSE | 0.06 | FALSE | 0.2 | FALSE | 0.09 | FALSE |
| Jabari | 3 | 2015 | 0.096 | Cd | 0.31 | FALSE | 0.08 | FALSE | 0.01 | FALSE | 0.14 | FALSE | 0.02 | FALSE |
| Jabari | 3 | 2015 | 49.4 | Cr | 0.61 | FALSE | 0.61 | FALSE | 0.13 | FALSE | 0.94 | FALSE | 0.31 | FALSE |
| Jabari | 3 | 2015 | 17.5 | Cu | 0.65 | FALSE | 0.51 | FALSE | 0.06 | FALSE | 0.93 | FALSE | 0.16 | FALSE |
| Jabari | 3 | 2015 | 28.9 | Ni | 0.8 | FALSE | 1.38 | TRUE | 0.56 | FALSE | NA | NA | NA | NA |
| Jabari | 3 | 2015 | 5.2 | Pb | 0.14 | FALSE | 0.11 | FALSE | 0.02 | FALSE | 0.17 | FALSE | 0.05 | FALSE |
| Jabari | 3 | 2015 | 18.5 | Zn | 0.15 | FALSE | 0.12 | FALSE | 0.05 | FALSE | 0.15 | FALSE | 0.07 | FALSE |
| Jabari | 3 | 2014 | 0.137 | Cd | 0.44 | FALSE | 0.11 | FALSE | 0.01 | FALSE | 0.2 | FALSE | 0.03 | FALSE |
| Jabari | 3 | 2014 | 44.4 | Cr | 0.55 | FALSE | 0.55 | FALSE | 0.12 | FALSE | 0.85 | FALSE | 0.28 | FALSE |
| Jabari | 3 | 2014 | 14.5 | Cu | 0.54 | FALSE | 0.43 | FALSE | 0.05 | FALSE | 0.78 | FALSE | 0.13 | FALSE |
| Jabari | 3 | 2014 | 38.8 | Ni | 1.08 | TRUE | 1.86 | TRUE | 0.75 | FALSE | NA | NA | NA | NA |
| Jabari | 3 | 2014 | 4.53 | Pb | 0.12 | FALSE | 0.1 | FALSE | 0.02 | FALSE | 0.15 | FALSE | 0.04 | FALSE |
| Jabari | 3 | 2014 | 30.4 | Zn | 0.25 | FALSE | 0.2 | FALSE | 0.07 | FALSE | 0.25 | FALSE | 0.11 | FALSE |
| Jabari | 3 | 2013 | 0.103 | Cd | 0.33 | FALSE | 0.09 | FALSE | 0.01 | FALSE | 0.15 | FALSE | 0.02 | FALSE |
| Jabari | 3 | 2013 | 48.8 | Cr | 0.6 | FALSE | 0.6 | FALSE | 0.13 | FALSE | 0.93 | FALSE | 0.3 | FALSE |
| Jabari | 3 | 2013 | 15.4 | Cu | 0.57 | FALSE | 0.45 | FALSE | 0.06 | FALSE | 0.82 | FALSE | 0.14 | FALSE |
| Jabari | 3 | 2013 | 23.4 | Ni | 0.65 | FALSE | 1.12 | TRUE | 0.45 | FALSE | NA | NA | NA | NA |
| Jabari | 3 | 2013 | 6.16 | Pb | 0.16 | FALSE | 0.13 | FALSE | 0.03 | FALSE | 0.2 | FALSE | 0.06 | FALSE |
| Jabari | 3 | 2013 | 19.3 | Zn | 0.16 | FALSE | 0.13 | FALSE | 0.05 | FALSE | 0.16 | FALSE | 0.07 | FALSE |
| Jabari | 3 | 2012 | 0.103 | Cd | 0.33 | FALSE | 0.09 | FALSE | 0.01 | FALSE | 0.15 | FALSE | 0.02 | FALSE |
| Jabari | 3 | 2012 | 38.9 | Cr | 0.48 | FALSE | 0.48 | FALSE | 0.11 | FALSE | 0.74 | FALSE | 0.24 | FALSE |
| Jabari | 3 | 2012 | 11.2 | Cu | 0.42 | FALSE | 0.33 | FALSE | 0.04 | FALSE | 0.6 | FALSE | 0.1 | FALSE |
| Jabari | 3 | 2012 | 23.5 | Ni | 0.65 | FALSE | 1.13 | TRUE | 0.46 | FALSE | NA | NA | NA | NA |
| Jabari | 3 | 2012 | 5.87 | Pb | 0.15 | FALSE | 0.13 | FALSE | 0.03 | FALSE | 0.19 | FALSE | 0.05 | FALSE |
| Jabari | 3 | 2012 | 23.5 | Zn | 0.19 | FALSE | 0.16 | FALSE | 0.06 | FALSE | 0.19 | FALSE | 0.09 | FALSE |
| Jabari | 3 | 2011 | 0.105 | Cd | 0.34 | FALSE | 0.09 | FALSE | 0.01 | FALSE | 0.15 | FALSE | 0.03 | FALSE |
| Jabari | 3 | 2011 | 57 | Cr | 0.7 | FALSE | 0.7 | FALSE | 0.15 | FALSE | 1.09 | TRUE | 0.36 | FALSE |
| Jabari | 3 | 2011 | 17.5 | Cu | 0.65 | FALSE | 0.52 | FALSE | 0.06 | FALSE | 0.94 | FALSE | 0.16 | FALSE |
| Jabari | 3 | 2011 | 35 | Ni | 0.97 | FALSE | 1.67 | TRUE | 0.68 | FALSE | NA | NA | NA | NA |
| Jabari | 3 | 2011 | 2.46 | Pb | 0.06 | FALSE | 0.05 | FALSE | 0.01 | FALSE | 0.08 | FALSE | 0.02 | FALSE |
| Jabari | 3 | 2011 | 39.5 | Zn | 0.32 | FALSE | 0.26 | FALSE | 0.1 | FALSE | 0.32 | FALSE | 0.15 | FALSE |
| Jabari | 3 | 2010 | 0.057 | Cd | 0.18 | FALSE | 0.05 | FALSE | 0.01 | FALSE | 0.08 | FALSE | 0.01 | FALSE |
| Jabari | 3 | 2010 | 64.6 | Cr | 0.8 | FALSE | 0.8 | FALSE | 0.17 | FALSE | 1.24 | TRUE | 0.4 | FALSE |
| Jabari | 3 | 2010 | 9.7 | Cu | 0.36 | FALSE | 0.28 | FALSE | 0.04 | FALSE | 0.52 | FALSE | 0.09 | FALSE |
| Jabari | 3 | 2010 | 9 | Ni | 0.25 | FALSE | 0.43 | FALSE | 0.17 | FALSE | NA | NA | NA | NA |
| Jabari | 3 | 2010 | 2.82 | Pb | 0.07 | FALSE | 0.06 | FALSE | 0.01 | FALSE | 0.09 | FALSE | 0.03 | FALSE |
| Jabari | 3 | 2010 | 25.5 | Zn | 0.21 | FALSE | 0.17 | FALSE | 0.06 | FALSE | 0.21 | FALSE | 0.09 | FALSE |
| Jabari | 3 | 2009 | 0.114 | Cd | 0.37 | FALSE | 0.09 | FALSE | 0.01 | FALSE | 0.16 | FALSE | 0.03 | FALSE |
| Jabari | 3 | 2009 | 54.3 | Cr | 0.67 | FALSE | 0.67 | FALSE | 0.15 | FALSE | 1.04 | TRUE | 0.34 | FALSE |
| Jabari | 3 | 2009 | 9 | Cu | 0.33 | FALSE | 0.27 | FALSE | 0.03 | FALSE | 0.48 | FALSE | 0.08 | FALSE |
| Jabari | 3 | 2009 | 25.1 | Ni | 0.7 | FALSE | 1.2 | TRUE | 0.49 | FALSE | NA | NA | NA | NA |
| Jabari | 3 | 2009 | 3.92 | Pb | 0.1 | FALSE | 0.08 | FALSE | 0.02 | FALSE | 0.13 | FALSE | 0.03 | FALSE |
| Jabari | 3 | 2009 | 23.4 | Zn | 0.19 | FALSE | 0.16 | FALSE | 0.06 | FALSE | 0.19 | FALSE | 0.09 | FALSE |
| Jabari | 3 | 2008 | 0.129 | Cd | 0.42 | FALSE | 0.11 | FALSE | 0.01 | FALSE | 0.18 | FALSE | 0.03 | FALSE |
| Jabari | 3 | 2008 | 46.2 | Cr | 0.57 | FALSE | 0.57 | FALSE | 0.12 | FALSE | 0.88 | FALSE | 0.29 | FALSE |
| Jabari | 3 | 2008 | 6 | Cu | 0.22 | FALSE | 0.18 | FALSE | 0.02 | FALSE | 0.32 | FALSE | 0.06 | FALSE |
| Jabari | 3 | 2008 | 17.7 | Ni | 0.49 | FALSE | 0.85 | FALSE | 0.34 | FALSE | NA | NA | NA | NA |
| Jabari | 3 | 2008 | 4.11 | Pb | 0.11 | FALSE | 0.09 | FALSE | 0.02 | FALSE | 0.14 | FALSE | 0.04 | FALSE |
| Jabari | 3 | 2008 | 4.9 | Zn | 0.04 | FALSE | 0.03 | FALSE | 0.01 | FALSE | 0.04 | FALSE | 0.02 | FALSE |
| Jabari | 3 | 2007 | 40 | Cr | 0.49 | FALSE | 0.49 | FALSE | 0.11 | FALSE | 0.76 | FALSE | 0.25 | FALSE |
| Jabari | 3 | 2007 | 21.2 | Cu | 0.79 | FALSE | 0.62 | FALSE | 0.08 | FALSE | 1.13 | TRUE | 0.2 | FALSE |
| Jabari | 3 | 2007 | 27.8 | Ni | 0.77 | FALSE | 1.33 | TRUE | 0.54 | FALSE | NA | NA | NA | NA |
| Jabari | 3 | 2007 | 3.55 | Pb | 0.09 | FALSE | 0.08 | FALSE | 0.02 | FALSE | 0.12 | FALSE | 0.03 | FALSE |
| Jabari | 3 | 2007 | 21.2 | Zn | 0.17 | FALSE | 0.14 | FALSE | 0.05 | FALSE | 0.17 | FALSE | 0.08 | FALSE |
| Tugailib | 4 | 2015 | 0.089 | Cd | 0.29 | FALSE | 0.07 | FALSE | 0.01 | FALSE | 0.13 | FALSE | 0.02 | FALSE |
| Tugailib | 4 | 2015 | 41.7 | Cr | 0.52 | FALSE | 0.52 | FALSE | 0.11 | FALSE | 0.8 | FALSE | 0.26 | FALSE |
| Tugailib | 4 | 2015 | 20.2 | Cu | 0.75 | FALSE | 0.59 | FALSE | 0.07 | FALSE | 1.08 | TRUE | 0.19 | FALSE |
| Tugailib | 4 | 2015 | 18.5 | Ni | 0.51 | FALSE | 0.89 | FALSE | 0.36 | FALSE | NA | NA | NA | NA |
| Tugailib | 4 | 2015 | 8.43 | Pb | 0.22 | FALSE | 0.18 | FALSE | 0.04 | FALSE | 0.28 | FALSE | 0.08 | FALSE |
| Tugailib | 4 | 2015 | 8.6 | Zn | 0.07 | FALSE | 0.06 | FALSE | 0.02 | FALSE | 0.07 | FALSE | 0.03 | FALSE |
| Tugailib | 4 | 2014 | 0.095 | Cd | 0.31 | FALSE | 0.08 | FALSE | 0.01 | FALSE | 0.14 | FALSE | 0.02 | FALSE |
| Tugailib | 4 | 2014 | 41.9 | Cr | 0.52 | FALSE | 0.52 | FALSE | 0.11 | FALSE | 0.8 | FALSE | 0.26 | FALSE |
| Tugailib | 4 | 2014 | 16.3 | Cu | 0.6 | FALSE | 0.48 | FALSE | 0.06 | FALSE | 0.87 | FALSE | 0.15 | FALSE |
| Tugailib | 4 | 2014 | 17.9 | Ni | 0.5 | FALSE | 0.86 | FALSE | 0.35 | FALSE | NA | NA | NA | NA |
| Tugailib | 4 | 2014 | 2.82 | Pb | 0.07 | FALSE | 0.06 | FALSE | 0.01 | FALSE | 0.09 | FALSE | 0.03 | FALSE |
| Tugailib | 4 | 2014 | 17.9 | Zn | 0.15 | FALSE | 0.12 | FALSE | 0.04 | FALSE | 0.14 | FALSE | 0.07 | FALSE |
| Tugailib | 4 | 2013 | 0.076 | Cd | 0.24 | FALSE | 0.06 | FALSE | 0.01 | FALSE | 0.11 | FALSE | 0.02 | FALSE |
| Tugailib | 4 | 2013 | 14.3 | Cu | 0.53 | FALSE | 0.42 | FALSE | 0.05 | FALSE | 0.76 | FALSE | 0.13 | FALSE |
| Tugailib | 4 | 2013 | 19.4 | Ni | 0.54 | FALSE | 0.93 | FALSE | 0.38 | FALSE | NA | NA | NA | NA |
| Tugailib | 4 | 2013 | 10.7 | Pb | 0.28 | FALSE | 0.23 | FALSE | 0.05 | FALSE | 0.35 | FALSE | 0.1 | FALSE |
| Tugailib | 4 | 2013 | 9.3 | Zn | 0.08 | FALSE | 0.06 | FALSE | 0.02 | FALSE | 0.07 | FALSE | 0.03 | FALSE |
| Tugailib | 4 | 2012 | 0.116 | Cd | 0.37 | FALSE | 0.1 | FALSE | 0.01 | FALSE | 0.17 | FALSE | 0.03 | FALSE |
| Tugailib | 4 | 2012 | 56.2 | Cr | 0.69 | FALSE | 0.69 | FALSE | 0.15 | FALSE | 1.07 | TRUE | 0.35 | FALSE |
| Tugailib | 4 | 2012 | 21.1 | Cu | 0.78 | FALSE | 0.62 | FALSE | 0.08 | FALSE | 1.13 | TRUE | 0.2 | FALSE |
| Tugailib | 4 | 2012 | 22.9 | Ni | 0.64 | FALSE | 1.1 | TRUE | 0.44 | FALSE | NA | NA | NA | NA |
| Tugailib | 4 | 2012 | 5.01 | Pb | 0.13 | FALSE | 0.11 | FALSE | 0.02 | FALSE | 0.17 | FALSE | 0.04 | FALSE |
| Tugailib | 4 | 2012 | 22.9 | Zn | 0.19 | FALSE | 0.15 | FALSE | 0.06 | FALSE | 0.18 | FALSE | 0.08 | FALSE |
| Tugailib | 4 | 2011 | 0.111 | Cd | 0.36 | FALSE | 0.09 | FALSE | 0.01 | FALSE | 0.16 | FALSE | 0.03 | FALSE |
| Tugailib | 4 | 2011 | 30.9 | Cu | 1.14 | TRUE | 0.91 | FALSE | 0.11 | FALSE | 1.65 | TRUE | 0.29 | FALSE |
| Tugailib | 4 | 2011 | 18.7 | Ni | 0.52 | FALSE | 0.9 | FALSE | 0.36 | FALSE | NA | NA | NA | NA |
| Tugailib | 4 | 2011 | 3.98 | Pb | 0.1 | FALSE | 0.09 | FALSE | 0.02 | FALSE | 0.13 | FALSE | 0.04 | FALSE |
| Tugailib | 4 | 2011 | 31 | Zn | 0.25 | FALSE | 0.21 | FALSE | 0.08 | FALSE | 0.25 | FALSE | 0.11 | FALSE |
| Tugailib | 4 | 2010 | 0.158 | Cd | 0.51 | FALSE | 0.13 | FALSE | 0.02 | FALSE | 0.23 | FALSE | 0.04 | FALSE |
| Tugailib | 4 | 2010 | 27 | Cu | 1 | TRUE | 0.79 | FALSE | 0.1 | FALSE | 1.44 | TRUE | 0.25 | FALSE |
| Tugailib | 4 | 2010 | 5.9 | Ni | 0.17 | FALSE | 0.28 | FALSE | 0.12 | FALSE | NA | NA | NA | NA |
| Tugailib | 4 | 2010 | 2.52 | Pb | 0.07 | FALSE | 0.05 | FALSE | 0.01 | FALSE | 0.08 | FALSE | 0.02 | FALSE |
| Tugailib | 4 | 2010 | 16.4 | Zn | 0.13 | FALSE | 0.11 | FALSE | 0.04 | FALSE | 0.13 | FALSE | 0.06 | FALSE |
| Tugailib | 4 | 2009 | 0.208 | Cd | 0.67 | FALSE | 0.17 | FALSE | 0.02 | FALSE | 0.3 | FALSE | 0.05 | FALSE |
| Tugailib | 4 | 2009 | 88.3 | Cr | 1.09 | TRUE | 1.09 | TRUE | 0.24 | FALSE | 1.69 | TRUE | 0.55 | FALSE |
| Tugailib | 4 | 2009 | 28.4 | Cu | 1.05 | TRUE | 0.84 | FALSE | 0.11 | FALSE | 1.52 | TRUE | 0.26 | FALSE |
| Tugailib | 4 | 2009 | 28.6 | Ni | 0.79 | FALSE | 1.37 | TRUE | 0.55 | FALSE | NA | NA | NA | NA |
| Tugailib | 4 | 2009 | 10.4 | Pb | 0.27 | FALSE | 0.22 | FALSE | 0.05 | FALSE | 0.35 | FALSE | 0.09 | FALSE |
| Tugailib | 4 | 2009 | 17.9 | Zn | 0.15 | FALSE | 0.12 | FALSE | 0.04 | FALSE | 0.14 | FALSE | 0.07 | FALSE |
| Tugailib | 4 | 2008 | 0.051 | Cd | 0.16 | FALSE | 0.04 | FALSE | 0.01 | FALSE | 0.07 | FALSE | 0.01 | FALSE |
| Tugailib | 4 | 2008 | 1.6 | Cu | 0.06 | FALSE | 0.05 | FALSE | 0.01 | FALSE | 0.09 | FALSE | 0.02 | FALSE |
| Tugailib | 4 | 2008 | 2.35 | Pb | 0.06 | FALSE | 0.05 | FALSE | 0.01 | FALSE | 0.08 | FALSE | 0.02 | FALSE |
| Tugailib | 4 | 2007 | 25.9 | Cr | 0.32 | FALSE | 0.32 | FALSE | 0.07 | FALSE | 0.5 | FALSE | 0.16 | FALSE |
| Tugailib | 4 | 2007 | 23.5 | Cu | 0.87 | FALSE | 0.69 | FALSE | 0.09 | FALSE | 1.25 | TRUE | 0.22 | FALSE |
| Tugailib | 4 | 2007 | 1.83 | Pb | 0.05 | FALSE | 0.04 | FALSE | 0.01 | FALSE | 0.06 | FALSE | 0.02 | FALSE |
| Ghumais | 5 | 2020 | 0.095 | Cd | 0.3 | FALSE | 0.08 | FALSE | 0.01 | FALSE | 0.14 | FALSE | 0.02 | FALSE |
| Ghumais | 5 | 2020 | 45.1 | Cr | 0.56 | FALSE | 0.56 | FALSE | 0.12 | FALSE | 0.86 | FALSE | 0.28 | FALSE |
| Ghumais | 5 | 2020 | 10.2 | Cu | 0.38 | FALSE | 0.3 | FALSE | 0.04 | FALSE | 0.54 | FALSE | 0.09 | FALSE |
| Ghumais | 5 | 2020 | 19.4 | Ni | 0.54 | FALSE | 0.93 | FALSE | 0.38 | FALSE | NA | NA | NA | NA |
| Ghumais | 5 | 2020 | 1.83 | Pb | 0.05 | FALSE | 0.04 | FALSE | 0.01 | FALSE | 0.06 | FALSE | 0.02 | FALSE |
| Ghumais | 5 | 2020 | 21.6 | Zn | 0.18 | FALSE | 0.14 | FALSE | 0.05 | FALSE | 0.17 | FALSE | 0.08 | FALSE |
| Ghumais | 5 | 2019 | 0.099 | Cd | 0.32 | FALSE | 0.08 | FALSE | 0.01 | FALSE | 0.14 | FALSE | 0.02 | FALSE |
| Ghumais | 5 | 2019 | 45.5 | Cr | 0.56 | FALSE | 0.56 | FALSE | 0.12 | FALSE | 0.87 | FALSE | 0.28 | FALSE |
| Ghumais | 5 | 2019 | 10.2 | Cu | 0.38 | FALSE | 0.3 | FALSE | 0.04 | FALSE | 0.55 | FALSE | 0.09 | FALSE |
| Ghumais | 5 | 2019 | 21.6 | Ni | 0.6 | FALSE | 1.03 | TRUE | 0.42 | FALSE | NA | NA | NA | NA |
| Ghumais | 5 | 2019 | 4.94 | Pb | 0.13 | FALSE | 0.11 | FALSE | 0.02 | FALSE | 0.16 | FALSE | 0.04 | FALSE |
| Ghumais | 5 | 2019 | 22.8 | Zn | 0.19 | FALSE | 0.15 | FALSE | 0.06 | FALSE | 0.18 | FALSE | 0.08 | FALSE |
| Ghumais | 5 | 2018 | 0.084 | Cd | 0.27 | FALSE | 0.07 | FALSE | 0.01 | FALSE | 0.12 | FALSE | 0.02 | FALSE |
| Ghumais | 5 | 2018 | 49.9 | Cr | 0.62 | FALSE | 0.62 | FALSE | 0.13 | FALSE | 0.95 | FALSE | 0.31 | FALSE |
| Ghumais | 5 | 2018 | 15.4 | Cu | 0.57 | FALSE | 0.45 | FALSE | 0.06 | FALSE | 0.82 | FALSE | 0.14 | FALSE |
| Ghumais | 5 | 2018 | 19.7 | Ni | 0.55 | FALSE | 0.94 | FALSE | 0.38 | FALSE | NA | NA | NA | NA |
| Ghumais | 5 | 2018 | 1.31 | Pb | 0.03 | FALSE | 0.03 | FALSE | 0.01 | FALSE | 0.04 | FALSE | 0.01 | FALSE |
| Ghumais | 5 | 2018 | 20 | Zn | 0.16 | FALSE | 0.13 | FALSE | 0.05 | FALSE | 0.16 | FALSE | 0.07 | FALSE |
| Ghumais | 5 | 2017 | 0.083 | Cd | 0.27 | FALSE | 0.07 | FALSE | 0.01 | FALSE | 0.12 | FALSE | 0.02 | FALSE |
| Ghumais | 5 | 2017 | 49.7 | Cr | 0.61 | FALSE | 0.61 | FALSE | 0.13 | FALSE | 0.95 | FALSE | 0.31 | FALSE |
| Ghumais | 5 | 2017 | 11.6 | Cu | 0.43 | FALSE | 0.34 | FALSE | 0.04 | FALSE | 0.62 | FALSE | 0.11 | FALSE |
| Ghumais | 5 | 2017 | 34.2 | Ni | 0.95 | FALSE | 1.63 | TRUE | 0.66 | FALSE | NA | NA | NA | NA |
| Ghumais | 5 | 2017 | 2.17 | Pb | 0.06 | FALSE | 0.05 | FALSE | 0.01 | FALSE | 0.07 | FALSE | 0.02 | FALSE |
| Ghumais | 5 | 2017 | 18.6 | Zn | 0.15 | FALSE | 0.12 | FALSE | 0.05 | FALSE | 0.15 | FALSE | 0.07 | FALSE |
| Ghumais | 5 | 2016 | 0.08 | Cd | 0.26 | FALSE | 0.07 | FALSE | 0.01 | FALSE | 0.11 | FALSE | 0.02 | FALSE |
| Ghumais | 5 | 2016 | 45.2 | Cr | 0.56 | FALSE | 0.56 | FALSE | 0.12 | FALSE | 0.87 | FALSE | 0.28 | FALSE |
| Ghumais | 5 | 2016 | 15.6 | Cu | 0.58 | FALSE | 0.46 | FALSE | 0.06 | FALSE | 0.83 | FALSE | 0.14 | FALSE |
| Ghumais | 5 | 2016 | 25.4 | Ni | 0.7 | FALSE | 1.21 | TRUE | 0.49 | FALSE | NA | NA | NA | NA |
| Ghumais | 5 | 2016 | 2.13 | Pb | 0.06 | FALSE | 0.05 | FALSE | 0.01 | FALSE | 0.07 | FALSE | 0.02 | FALSE |
| Ghumais | 5 | 2016 | 15.3 | Zn | 0.13 | FALSE | 0.1 | FALSE | 0.04 | FALSE | 0.12 | FALSE | 0.06 | FALSE |
| Ghumais | 5 | 2015 | 0.101 | Cd | 0.32 | FALSE | 0.08 | FALSE | 0.01 | FALSE | 0.14 | FALSE | 0.02 | FALSE |
| Ghumais | 5 | 2015 | 45.6 | Cr | 0.56 | FALSE | 0.56 | FALSE | 0.12 | FALSE | 0.87 | FALSE | 0.29 | FALSE |
| Ghumais | 5 | 2015 | 16 | Cu | 0.59 | FALSE | 0.47 | FALSE | 0.06 | FALSE | 0.86 | FALSE | 0.15 | FALSE |
| Ghumais | 5 | 2015 | 40.8 | Ni | 1.13 | TRUE | 1.95 | TRUE | 0.79 | FALSE | NA | NA | NA | NA |
| Ghumais | 5 | 2015 | 2.07 | Pb | 0.05 | FALSE | 0.04 | FALSE | 0.01 | FALSE | 0.07 | FALSE | 0.02 | FALSE |
| Ghumais | 5 | 2015 | 11 | Zn | 0.09 | FALSE | 0.07 | FALSE | 0.03 | FALSE | 0.09 | FALSE | 0.04 | FALSE |
| Ghumais | 5 | 2014 | 0.111 | Cd | 0.36 | FALSE | 0.09 | FALSE | 0.01 | FALSE | 0.16 | FALSE | 0.03 | FALSE |
| Ghumais | 5 | 2014 | 50.2 | Cr | 0.62 | FALSE | 0.62 | FALSE | 0.14 | FALSE | 0.96 | FALSE | 0.31 | FALSE |
| Ghumais | 5 | 2014 | 7.3 | Cu | 0.27 | FALSE | 0.21 | FALSE | 0.03 | FALSE | 0.39 | FALSE | 0.07 | FALSE |
| Ghumais | 5 | 2014 | 34.4 | Ni | 0.96 | FALSE | 1.65 | TRUE | 0.67 | FALSE | NA | NA | NA | NA |
| Ghumais | 5 | 2014 | 3.66 | Pb | 0.1 | FALSE | 0.08 | FALSE | 0.02 | FALSE | 0.12 | FALSE | 0.03 | FALSE |
| Ghumais | 5 | 2014 | 19.4 | Zn | 0.16 | FALSE | 0.13 | FALSE | 0.05 | FALSE | 0.16 | FALSE | 0.07 | FALSE |
| Ghumais | 5 | 2013 | 0.099 | Cd | 0.32 | FALSE | 0.08 | FALSE | 0.01 | FALSE | 0.14 | FALSE | 0.02 | FALSE |
| Ghumais | 5 | 2013 | 28.6 | Cr | 0.35 | FALSE | 0.35 | FALSE | 0.08 | FALSE | 0.55 | FALSE | 0.18 | FALSE |
| Ghumais | 5 | 2013 | 12.3 | Cu | 0.46 | FALSE | 0.36 | FALSE | 0.05 | FALSE | 0.66 | FALSE | 0.11 | FALSE |
| Ghumais | 5 | 2013 | 33.8 | Ni | 0.94 | FALSE | 1.62 | TRUE | 0.66 | FALSE | NA | NA | NA | NA |
| Ghumais | 5 | 2013 | 5.28 | Pb | 0.14 | FALSE | 0.11 | FALSE | 0.02 | FALSE | 0.17 | FALSE | 0.05 | FALSE |
| Ghumais | 5 | 2013 | 16.2 | Zn | 0.13 | FALSE | 0.11 | FALSE | 0.04 | FALSE | 0.13 | FALSE | 0.06 | FALSE |
| Ghumais | 5 | 2012 | 0.086 | Cd | 0.28 | FALSE | 0.07 | FALSE | 0.01 | FALSE | 0.12 | FALSE | 0.02 | FALSE |
| Ghumais | 5 | 2012 | 32.2 | Cr | 0.4 | FALSE | 0.4 | FALSE | 0.09 | FALSE | 0.62 | FALSE | 0.2 | FALSE |
| Ghumais | 5 | 2012 | 9.2 | Cu | 0.34 | FALSE | 0.27 | FALSE | 0.03 | FALSE | 0.49 | FALSE | 0.08 | FALSE |
| Ghumais | 5 | 2012 | 25.4 | Ni | 0.71 | FALSE | 1.21 | TRUE | 0.49 | FALSE | NA | NA | NA | NA |
| Ghumais | 5 | 2012 | 2.56 | Pb | 0.07 | FALSE | 0.05 | FALSE | 0.01 | FALSE | 0.08 | FALSE | 0.02 | FALSE |
| Ghumais | 5 | 2012 | 25.4 | Zn | 0.21 | FALSE | 0.17 | FALSE | 0.06 | FALSE | 0.2 | FALSE | 0.09 | FALSE |
| Ghumais | 5 | 2011 | 0.07 | Cd | 0.23 | FALSE | 0.06 | FALSE | 0.01 | FALSE | 0.1 | FALSE | 0.02 | FALSE |
| Ghumais | 5 | 2011 | 47.1 | Cr | 0.58 | FALSE | 0.58 | FALSE | 0.13 | FALSE | 0.9 | FALSE | 0.29 | FALSE |
| Ghumais | 5 | 2011 | 18.7 | Cu | 0.69 | FALSE | 0.55 | FALSE | 0.07 | FALSE | 1 | TRUE | 0.17 | FALSE |
| Ghumais | 5 | 2011 | 30.4 | Ni | 0.84 | FALSE | 1.45 | TRUE | 0.59 | FALSE | NA | NA | NA | NA |
| Ghumais | 5 | 2011 | 1.75 | Pb | 0.05 | FALSE | 0.04 | FALSE | 0.01 | FALSE | 0.06 | FALSE | 0.02 | FALSE |
| Ghumais | 5 | 2011 | 31.8 | Zn | 0.26 | FALSE | 0.21 | FALSE | 0.08 | FALSE | 0.26 | FALSE | 0.12 | FALSE |
| Ghumais | 5 | 2010 | 0.08 | Cd | 0.26 | FALSE | 0.07 | FALSE | 0.01 | FALSE | 0.11 | FALSE | 0.02 | FALSE |
| Ghumais | 5 | 2010 | 72 | Cr | 0.89 | FALSE | 0.89 | FALSE | 0.19 | FALSE | 1.38 | TRUE | 0.45 | FALSE |
| Ghumais | 5 | 2010 | 11.9 | Cu | 0.44 | FALSE | 0.35 | FALSE | 0.04 | FALSE | 0.64 | FALSE | 0.11 | FALSE |
| Ghumais | 5 | 2010 | 5.5 | Ni | 0.15 | FALSE | 0.26 | FALSE | 0.11 | FALSE | NA | NA | NA | NA |
| Ghumais | 5 | 2010 | 2.48 | Pb | 0.07 | FALSE | 0.05 | FALSE | 0.01 | FALSE | 0.08 | FALSE | 0.02 | FALSE |
| Ghumais | 5 | 2010 | 21.1 | Zn | 0.17 | FALSE | 0.14 | FALSE | 0.05 | FALSE | 0.17 | FALSE | 0.08 | FALSE |
| Ghumais | 5 | 2009 | 0.121 | Cd | 0.39 | FALSE | 0.1 | FALSE | 0.01 | FALSE | 0.17 | FALSE | 0.03 | FALSE |
| Ghumais | 5 | 2009 | 56.6 | Cr | 0.7 | FALSE | 0.7 | FALSE | 0.15 | FALSE | 1.08 | TRUE | 0.35 | FALSE |
| Ghumais | 5 | 2009 | 10.7 | Cu | 0.4 | FALSE | 0.32 | FALSE | 0.04 | FALSE | 0.57 | FALSE | 0.1 | FALSE |
| Ghumais | 5 | 2009 | 24.3 | Ni | 0.67 | FALSE | 1.16 | TRUE | 0.47 | FALSE | NA | NA | NA | NA |
| Ghumais | 5 | 2009 | 2.85 | Pb | 0.07 | FALSE | 0.06 | FALSE | 0.01 | FALSE | 0.09 | FALSE | 0.03 | FALSE |
| Ghumais | 5 | 2009 | 18 | Zn | 0.15 | FALSE | 0.12 | FALSE | 0.04 | FALSE | 0.15 | FALSE | 0.07 | FALSE |
| Ghumais | 5 | 2008 | 0.152 | Cd | 0.49 | FALSE | 0.13 | FALSE | 0.02 | FALSE | 0.22 | FALSE | 0.04 | FALSE |
| Ghumais | 5 | 2008 | 47.3 | Cr | 0.58 | FALSE | 0.58 | FALSE | 0.13 | FALSE | 0.91 | FALSE | 0.3 | FALSE |
| Ghumais | 5 | 2008 | 5.9 | Cu | 0.22 | FALSE | 0.17 | FALSE | 0.02 | FALSE | 0.31 | FALSE | 0.05 | FALSE |
| Ghumais | 5 | 2008 | 15.5 | Ni | 0.43 | FALSE | 0.74 | FALSE | 0.3 | FALSE | NA | NA | NA | NA |
| Ghumais | 5 | 2008 | 3.43 | Pb | 0.09 | FALSE | 0.07 | FALSE | 0.02 | FALSE | 0.11 | FALSE | 0.03 | FALSE |
| Ghumais | 5 | 2008 | 6.3 | Zn | 0.05 | FALSE | 0.04 | FALSE | 0.02 | FALSE | 0.05 | FALSE | 0.02 | FALSE |
| Ghumais | 5 | 2007 | 42.7 | Cr | 0.53 | FALSE | 0.53 | FALSE | 0.12 | FALSE | 0.82 | FALSE | 0.27 | FALSE |
| Ghumais | 5 | 2007 | 15.9 | Cu | 0.59 | FALSE | 0.47 | FALSE | 0.06 | FALSE | 0.85 | FALSE | 0.15 | FALSE |
| Ghumais | 5 | 2007 | 24.6 | Ni | 0.68 | FALSE | 1.18 | TRUE | 0.48 | FALSE | NA | NA | NA | NA |
| Ghumais | 5 | 2007 | 3.72 | Pb | 0.1 | FALSE | 0.08 | FALSE | 0.02 | FALSE | 0.12 | FALSE | 0.03 | FALSE |
| Ghumais | 5 | 2007 | 12.3 | Zn | 0.1 | FALSE | 0.08 | FALSE | 0.03 | FALSE | 0.1 | FALSE | 0.05 | FALSE |
| Askar | 6 | 2020 | 0.089 | Cd | 0.29 | FALSE | 0.07 | FALSE | 0.01 | FALSE | 0.13 | FALSE | 0.02 | FALSE |
| Askar | 6 | 2020 | 46.8 | Cr | 0.58 | FALSE | 0.58 | FALSE | 0.13 | FALSE | 0.9 | FALSE | 0.29 | FALSE |
| Askar | 6 | 2020 | 13.7 | Cu | 0.51 | FALSE | 0.4 | FALSE | 0.05 | FALSE | 0.74 | FALSE | 0.13 | FALSE |
| Askar | 6 | 2020 | 11.2 | Ni | 0.31 | FALSE | 0.54 | FALSE | 0.22 | FALSE | NA | NA | NA | NA |
| Askar | 6 | 2020 | 4.57 | Pb | 0.12 | FALSE | 0.1 | FALSE | 0.02 | FALSE | 0.15 | FALSE | 0.04 | FALSE |
| Askar | 6 | 2020 | 26.8 | Zn | 0.22 | FALSE | 0.18 | FALSE | 0.07 | FALSE | 0.22 | FALSE | 0.1 | FALSE |
| Askar | 6 | 2019 | 0.082 | Cd | 0.26 | FALSE | 0.07 | FALSE | 0.01 | FALSE | 0.12 | FALSE | 0.02 | FALSE |
| Askar | 6 | 2019 | 55.5 | Cr | 0.69 | FALSE | 0.69 | FALSE | 0.15 | FALSE | 1.06 | TRUE | 0.35 | FALSE |
| Askar | 6 | 2019 | 14.9 | Cu | 0.55 | FALSE | 0.44 | FALSE | 0.06 | FALSE | 0.8 | FALSE | 0.14 | FALSE |
| Askar | 6 | 2019 | 14.1 | Ni | 0.39 | FALSE | 0.67 | FALSE | 0.27 | FALSE | NA | NA | NA | NA |
| Askar | 6 | 2019 | 12.2 | Pb | 0.32 | FALSE | 0.26 | FALSE | 0.06 | FALSE | 0.41 | FALSE | 0.11 | FALSE |
| Askar | 6 | 2019 | 29.1 | Zn | 0.24 | FALSE | 0.19 | FALSE | 0.07 | FALSE | 0.23 | FALSE | 0.11 | FALSE |
| Askar | 6 | 2018 | 0.079 | Cd | 0.26 | FALSE | 0.07 | FALSE | 0.01 | FALSE | 0.11 | FALSE | 0.02 | FALSE |
| Askar | 6 | 2018 | 57.7 | Cr | 0.71 | FALSE | 0.71 | FALSE | 0.16 | FALSE | 1.1 | TRUE | 0.36 | FALSE |
| Askar | 6 | 2018 | 24.2 | Cu | 0.9 | FALSE | 0.71 | FALSE | 0.09 | FALSE | 1.29 | TRUE | 0.22 | FALSE |
| Askar | 6 | 2018 | 13.8 | Ni | 0.38 | FALSE | 0.66 | FALSE | 0.27 | FALSE | NA | NA | NA | NA |
| Askar | 6 | 2018 | 4.66 | Pb | 0.12 | FALSE | 0.1 | FALSE | 0.02 | FALSE | 0.15 | FALSE | 0.04 | FALSE |
| Askar | 6 | 2018 | 30.6 | Zn | 0.25 | FALSE | 0.2 | FALSE | 0.07 | FALSE | 0.25 | FALSE | 0.11 | FALSE |
| Askar | 6 | 2017 | 0.08 | Cd | 0.26 | FALSE | 0.07 | FALSE | 0.01 | FALSE | 0.11 | FALSE | 0.02 | FALSE |
| Askar | 6 | 2017 | 60.2 | Cr | 0.74 | FALSE | 0.74 | FALSE | 0.16 | FALSE | 1.15 | TRUE | 0.38 | FALSE |
| Askar | 6 | 2017 | 16.9 | Cu | 0.63 | FALSE | 0.5 | FALSE | 0.06 | FALSE | 0.91 | FALSE | 0.16 | FALSE |
| Askar | 6 | 2017 | 9.4 | Ni | 0.26 | FALSE | 0.45 | FALSE | 0.18 | FALSE | NA | NA | NA | NA |
| Askar | 6 | 2017 | 4.96 | Pb | 0.13 | FALSE | 0.11 | FALSE | 0.02 | FALSE | 0.16 | FALSE | 0.04 | FALSE |
| Askar | 6 | 2017 | 23.5 | Zn | 0.19 | FALSE | 0.16 | FALSE | 0.06 | FALSE | 0.19 | FALSE | 0.09 | FALSE |
| Askar | 6 | 2016 | 0.099 | Cd | 0.32 | FALSE | 0.08 | FALSE | 0.01 | FALSE | 0.14 | FALSE | 0.02 | FALSE |
| Askar | 6 | 2016 | 47.5 | Cr | 0.59 | FALSE | 0.59 | FALSE | 0.13 | FALSE | 0.91 | FALSE | 0.3 | FALSE |
| Askar | 6 | 2016 | 23.6 | Cu | 0.87 | FALSE | 0.69 | FALSE | 0.09 | FALSE | 1.26 | TRUE | 0.22 | FALSE |
| Askar | 6 | 2016 | 11.3 | Ni | 0.31 | FALSE | 0.54 | FALSE | 0.22 | FALSE | NA | NA | NA | NA |
| Askar | 6 | 2016 | 8.13 | Pb | 0.21 | FALSE | 0.17 | FALSE | 0.04 | FALSE | 0.27 | FALSE | 0.07 | FALSE |
| Askar | 6 | 2016 | 28.8 | Zn | 0.24 | FALSE | 0.19 | FALSE | 0.07 | FALSE | 0.23 | FALSE | 0.11 | FALSE |
| Askar | 6 | 2015 | 0.102 | Cd | 0.33 | FALSE | 0.09 | FALSE | 0.01 | FALSE | 0.15 | FALSE | 0.02 | FALSE |
| Askar | 6 | 2015 | 54.1 | Cr | 0.67 | FALSE | 0.67 | FALSE | 0.15 | FALSE | 1.03 | TRUE | 0.34 | FALSE |
| Askar | 6 | 2015 | 24.1 | Cu | 0.89 | FALSE | 0.71 | FALSE | 0.09 | FALSE | 1.29 | TRUE | 0.22 | FALSE |
| Askar | 6 | 2015 | 26.9 | Ni | 0.75 | FALSE | 1.29 | TRUE | 0.52 | FALSE | NA | NA | NA | NA |
| Askar | 6 | 2015 | 16.3 | Pb | 0.43 | FALSE | 0.35 | FALSE | 0.07 | FALSE | 0.54 | FALSE | 0.15 | FALSE |
| Askar | 6 | 2015 | 28.3 | Zn | 0.23 | FALSE | 0.19 | FALSE | 0.07 | FALSE | 0.23 | FALSE | 0.1 | FALSE |
| Askar | 6 | 2014 | 0.145 | Cd | 0.47 | FALSE | 0.12 | FALSE | 0.02 | FALSE | 0.21 | FALSE | 0.03 | FALSE |
| Askar | 6 | 2014 | 43 | Cr | 0.53 | FALSE | 0.53 | FALSE | 0.12 | FALSE | 0.82 | FALSE | 0.27 | FALSE |
| Askar | 6 | 2014 | 21.2 | Cu | 0.79 | FALSE | 0.62 | FALSE | 0.08 | FALSE | 1.13 | TRUE | 0.2 | FALSE |
| Askar | 6 | 2014 | 12.8 | Ni | 0.35 | FALSE | 0.61 | FALSE | 0.25 | FALSE | NA | NA | NA | NA |
| Askar | 6 | 2014 | 11.5 | Pb | 0.3 | FALSE | 0.25 | FALSE | 0.05 | FALSE | 0.38 | FALSE | 0.1 | FALSE |
| Askar | 6 | 2014 | 39.4 | Zn | 0.32 | FALSE | 0.26 | FALSE | 0.1 | FALSE | 0.32 | FALSE | 0.15 | FALSE |
| Askar | 6 | 2013 | 0.105 | Cd | 0.34 | FALSE | 0.09 | FALSE | 0.01 | FALSE | 0.15 | FALSE | 0.02 | FALSE |
| Askar | 6 | 2013 | 43.1 | Cr | 0.53 | FALSE | 0.53 | FALSE | 0.12 | FALSE | 0.82 | FALSE | 0.27 | FALSE |
| Askar | 6 | 2013 | 18.6 | Cu | 0.69 | FALSE | 0.55 | FALSE | 0.07 | FALSE | 0.99 | FALSE | 0.17 | FALSE |
| Askar | 6 | 2013 | 19.4 | Ni | 0.54 | FALSE | 0.93 | FALSE | 0.38 | FALSE | NA | NA | NA | NA |
| Askar | 6 | 2013 | 16.3 | Pb | 0.43 | FALSE | 0.35 | FALSE | 0.07 | FALSE | 0.54 | FALSE | 0.15 | FALSE |
| Askar | 6 | 2013 | 27.2 | Zn | 0.22 | FALSE | 0.18 | FALSE | 0.07 | FALSE | 0.22 | FALSE | 0.1 | FALSE |
| Askar | 6 | 2012 | 0.125 | Cd | 0.4 | FALSE | 0.1 | FALSE | 0.01 | FALSE | 0.18 | FALSE | 0.03 | FALSE |
| Askar | 6 | 2012 | 50.7 | Cr | 0.63 | FALSE | 0.63 | FALSE | 0.14 | FALSE | 0.97 | FALSE | 0.32 | FALSE |
| Askar | 6 | 2012 | 15.5 | Cu | 0.57 | FALSE | 0.46 | FALSE | 0.06 | FALSE | 0.83 | FALSE | 0.14 | FALSE |
| Askar | 6 | 2012 | 40.7 | Ni | 1.13 | TRUE | 1.95 | TRUE | 0.79 | FALSE | NA | NA | NA | NA |
| Askar | 6 | 2012 | 13.7 | Pb | 0.36 | FALSE | 0.29 | FALSE | 0.06 | FALSE | 0.45 | FALSE | 0.12 | FALSE |
| Askar | 6 | 2012 | 40.7 | Zn | 0.33 | FALSE | 0.27 | FALSE | 0.1 | FALSE | 0.33 | FALSE | 0.15 | FALSE |
| Askar | 6 | 2011 | 0.097 | Cd | 0.31 | FALSE | 0.08 | FALSE | 0.01 | FALSE | 0.14 | FALSE | 0.02 | FALSE |
| Askar | 6 | 2011 | 50.5 | Cr | 0.62 | FALSE | 0.62 | FALSE | 0.14 | FALSE | 0.97 | FALSE | 0.32 | FALSE |
| Askar | 6 | 2011 | 29.3 | Cu | 1.09 | TRUE | 0.86 | FALSE | 0.11 | FALSE | 1.57 | TRUE | 0.27 | FALSE |
| Askar | 6 | 2011 | 30.1 | Ni | 0.84 | FALSE | 1.44 | TRUE | 0.58 | FALSE | NA | NA | NA | NA |
| Askar | 6 | 2011 | 17.2 | Pb | 0.45 | FALSE | 0.37 | FALSE | 0.08 | FALSE | 0.57 | FALSE | 0.15 | FALSE |
| Askar | 6 | 2011 | 45.6 | Zn | 0.37 | FALSE | 0.3 | FALSE | 0.11 | FALSE | 0.37 | FALSE | 0.17 | FALSE |
| Askar | 6 | 2010 | 0.107 | Cd | 0.35 | FALSE | 0.09 | FALSE | 0.01 | FALSE | 0.15 | FALSE | 0.03 | FALSE |
| Askar | 6 | 2010 | 70.3 | Cr | 0.87 | FALSE | 0.87 | FALSE | 0.19 | FALSE | 1.34 | TRUE | 0.44 | FALSE |
| Askar | 6 | 2010 | 16 | Cu | 0.59 | FALSE | 0.47 | FALSE | 0.06 | FALSE | 0.86 | FALSE | 0.15 | FALSE |
| Askar | 6 | 2010 | 14.7 | Ni | 0.41 | FALSE | 0.7 | FALSE | 0.28 | FALSE | NA | NA | NA | NA |
| Askar | 6 | 2010 | 15.7 | Pb | 0.41 | FALSE | 0.34 | FALSE | 0.07 | FALSE | 0.52 | FALSE | 0.14 | FALSE |
| Askar | 6 | 2010 | 37.3 | Zn | 0.31 | FALSE | 0.25 | FALSE | 0.09 | FALSE | 0.3 | FALSE | 0.14 | FALSE |
| Askar | 6 | 2009 | 0.129 | Cd | 0.42 | FALSE | 0.11 | FALSE | 0.01 | FALSE | 0.18 | FALSE | 0.03 | FALSE |
| Askar | 6 | 2009 | 66.3 | Cr | 0.82 | FALSE | 0.82 | FALSE | 0.18 | FALSE | 1.27 | TRUE | 0.41 | FALSE |
| Askar | 6 | 2009 | 12.3 | Cu | 0.45 | FALSE | 0.36 | FALSE | 0.05 | FALSE | 0.66 | FALSE | 0.11 | FALSE |
| Askar | 6 | 2009 | 15.3 | Ni | 0.43 | FALSE | 0.73 | FALSE | 0.3 | FALSE | NA | NA | NA | NA |
| Askar | 6 | 2009 | 12.2 | Pb | 0.32 | FALSE | 0.26 | FALSE | 0.06 | FALSE | 0.4 | FALSE | 0.11 | FALSE |
| Askar | 6 | 2009 | 28.1 | Zn | 0.23 | FALSE | 0.19 | FALSE | 0.07 | FALSE | 0.23 | FALSE | 0.1 | FALSE |
| Askar | 6 | 2008 | 0.084 | Cd | 0.27 | FALSE | 0.07 | FALSE | 0.01 | FALSE | 0.12 | FALSE | 0.02 | FALSE |
| Askar | 6 | 2008 | 35 | Cr | 0.43 | FALSE | 0.43 | FALSE | 0.09 | FALSE | 0.67 | FALSE | 0.22 | FALSE |
| Askar | 6 | 2008 | 8.7 | Cu | 0.32 | FALSE | 0.26 | FALSE | 0.03 | FALSE | 0.47 | FALSE | 0.08 | FALSE |
| Askar | 6 | 2008 | 2.1 | Ni | 0.06 | FALSE | 0.1 | FALSE | 0.04 | FALSE | NA | NA | NA | NA |
| Askar | 6 | 2008 | 12.3 | Pb | 0.32 | FALSE | 0.26 | FALSE | 0.06 | FALSE | 0.41 | FALSE | 0.11 | FALSE |
| Askar | 6 | 2008 | 16.8 | Zn | 0.14 | FALSE | 0.11 | FALSE | 0.04 | FALSE | 0.14 | FALSE | 0.06 | FALSE |
| Askar | 6 | 2007 | 46.9 | Cr | 0.58 | FALSE | 0.58 | FALSE | 0.13 | FALSE | 0.9 | FALSE | 0.29 | FALSE |
| Askar | 6 | 2007 | 49.2 | Cu | 1.82 | TRUE | 1.45 | TRUE | 0.18 | FALSE | 2.63 | TRUE | 0.46 | FALSE |
| Askar | 6 | 2007 | 20.5 | Ni | 0.57 | FALSE | 0.98 | FALSE | 0.4 | FALSE | NA | NA | NA | NA |
| Askar | 6 | 2007 | 13.5 | Pb | 0.36 | FALSE | 0.29 | FALSE | 0.06 | FALSE | 0.45 | FALSE | 0.12 | FALSE |
| Askar | 6 | 2007 | 25.6 | Zn | 0.21 | FALSE | 0.17 | FALSE | 0.06 | FALSE | 0.21 | FALSE | 0.09 | FALSE |
| Msoor | 7 | 2016 | 0.094 | Cd | 0.3 | FALSE | 0.08 | FALSE | 0.01 | FALSE | 0.13 | FALSE | 0.02 | FALSE |
| Msoor | 7 | 2016 | 32.4 | Cr | 0.4 | FALSE | 0.4 | FALSE | 0.09 | FALSE | 0.62 | FALSE | 0.2 | FALSE |
| Msoor | 7 | 2016 | 16 | Cu | 0.59 | FALSE | 0.47 | FALSE | 0.06 | FALSE | 0.86 | FALSE | 0.15 | FALSE |
| Msoor | 7 | 2016 | 7.8 | Ni | 0.22 | FALSE | 0.37 | FALSE | 0.15 | FALSE | NA | NA | NA | NA |
| Msoor | 7 | 2016 | 1.44 | Pb | 0.04 | FALSE | 0.03 | FALSE | 0.01 | FALSE | 0.05 | FALSE | 0.01 | FALSE |
| Msoor | 7 | 2016 | 20.2 | Zn | 0.17 | FALSE | 0.13 | FALSE | 0.05 | FALSE | 0.16 | FALSE | 0.07 | FALSE |
| Msoor | 7 | 2015 | 0.13 | Cd | 0.42 | FALSE | 0.11 | FALSE | 0.01 | FALSE | 0.19 | FALSE | 0.03 | FALSE |
| Msoor | 7 | 2015 | 36.4 | Cr | 0.45 | FALSE | 0.45 | FALSE | 0.1 | FALSE | 0.7 | FALSE | 0.23 | FALSE |
| Msoor | 7 | 2015 | 16.6 | Cu | 0.62 | FALSE | 0.49 | FALSE | 0.06 | FALSE | 0.89 | FALSE | 0.15 | FALSE |
| Msoor | 7 | 2015 | 26.8 | Ni | 0.75 | FALSE | 1.28 | TRUE | 0.52 | FALSE | NA | NA | NA | NA |
| Msoor | 7 | 2015 | 1.33 | Pb | 0.03 | FALSE | 0.03 | FALSE | 0.01 | FALSE | 0.04 | FALSE | 0.01 | FALSE |
| Msoor | 7 | 2015 | 16.9 | Zn | 0.14 | FALSE | 0.11 | FALSE | 0.04 | FALSE | 0.14 | FALSE | 0.06 | FALSE |
| Msoor | 7 | 2014 | 0.127 | Cd | 0.41 | FALSE | 0.11 | FALSE | 0.01 | FALSE | 0.18 | FALSE | 0.03 | FALSE |
| Msoor | 7 | 2014 | 46.1 | Cr | 0.57 | FALSE | 0.57 | FALSE | 0.12 | FALSE | 0.88 | FALSE | 0.29 | FALSE |
| Msoor | 7 | 2014 | 9.4 | Cu | 0.35 | FALSE | 0.28 | FALSE | 0.03 | FALSE | 0.5 | FALSE | 0.09 | FALSE |
| Msoor | 7 | 2014 | 11.3 | Ni | 0.31 | FALSE | 0.54 | FALSE | 0.22 | FALSE | NA | NA | NA | NA |
| Msoor | 7 | 2014 | 2.79 | Pb | 0.07 | FALSE | 0.06 | FALSE | 0.01 | FALSE | 0.09 | FALSE | 0.02 | FALSE |
| Msoor | 7 | 2014 | 23.2 | Zn | 0.19 | FALSE | 0.15 | FALSE | 0.06 | FALSE | 0.19 | FALSE | 0.09 | FALSE |
| Msoor | 7 | 2013 | 0.1 | Cd | 0.32 | FALSE | 0.08 | FALSE | 0.01 | FALSE | 0.14 | FALSE | 0.02 | FALSE |
| Msoor | 7 | 2013 | 31.5 | Cr | 0.39 | FALSE | 0.39 | FALSE | 0.09 | FALSE | 0.6 | FALSE | 0.2 | FALSE |
| Msoor | 7 | 2013 | 19.1 | Cu | 0.71 | FALSE | 0.56 | FALSE | 0.07 | FALSE | 1.02 | TRUE | 0.18 | FALSE |
| Msoor | 7 | 2013 | 15.2 | Ni | 0.42 | FALSE | 0.73 | FALSE | 0.3 | FALSE | NA | NA | NA | NA |
| Msoor | 7 | 2013 | 3.82 | Pb | 0.1 | FALSE | 0.08 | FALSE | 0.02 | FALSE | 0.13 | FALSE | 0.03 | FALSE |
| Msoor | 7 | 2013 | 17.4 | Zn | 0.14 | FALSE | 0.12 | FALSE | 0.04 | FALSE | 0.14 | FALSE | 0.06 | FALSE |
| Msoor | 7 | 2012 | 0.103 | Cd | 0.33 | FALSE | 0.09 | FALSE | 0.01 | FALSE | 0.15 | FALSE | 0.02 | FALSE |
| Msoor | 7 | 2012 | 31.8 | Cr | 0.39 | FALSE | 0.39 | FALSE | 0.09 | FALSE | 0.61 | FALSE | 0.2 | FALSE |
| Msoor | 7 | 2012 | 9.6 | Cu | 0.36 | FALSE | 0.28 | FALSE | 0.04 | FALSE | 0.51 | FALSE | 0.09 | FALSE |
| Msoor | 7 | 2012 | 24.7 | Ni | 0.68 | FALSE | 1.18 | TRUE | 0.48 | FALSE | NA | NA | NA | NA |
| Msoor | 7 | 2012 | 2.79 | Pb | 0.07 | FALSE | 0.06 | FALSE | 0.01 | FALSE | 0.09 | FALSE | 0.02 | FALSE |
| Msoor | 7 | 2012 | 24.7 | Zn | 0.2 | FALSE | 0.16 | FALSE | 0.06 | FALSE | 0.2 | FALSE | 0.09 | FALSE |
| Msoor | 7 | 2011 | 0.087 | Cd | 0.28 | FALSE | 0.07 | FALSE | 0.01 | FALSE | 0.12 | FALSE | 0.02 | FALSE |
| Msoor | 7 | 2011 | 39.8 | Cr | 0.49 | FALSE | 0.49 | FALSE | 0.11 | FALSE | 0.76 | FALSE | 0.25 | FALSE |
| Msoor | 7 | 2011 | 19 | Cu | 0.71 | FALSE | 0.56 | FALSE | 0.07 | FALSE | 1.02 | TRUE | 0.18 | FALSE |
| Msoor | 7 | 2011 | 26.6 | Ni | 0.74 | FALSE | 1.27 | TRUE | 0.52 | FALSE | NA | NA | NA | NA |
| Msoor | 7 | 2011 | 0.45 | Pb | 0.01 | FALSE | 0.01 | FALSE | 0 | FALSE | 0.01 | FALSE | 0 | FALSE |
| Msoor | 7 | 2011 | 36.6 | Zn | 0.3 | FALSE | 0.24 | FALSE | 0.09 | FALSE | 0.29 | FALSE | 0.13 | FALSE |
| Msoor | 7 | 2010 | 0.079 | Cd | 0.26 | FALSE | 0.07 | FALSE | 0.01 | FALSE | 0.11 | FALSE | 0.02 | FALSE |
| Msoor | 7 | 2010 | 54.5 | Cr | 0.67 | FALSE | 0.67 | FALSE | 0.15 | FALSE | 1.04 | TRUE | 0.34 | FALSE |
| Msoor | 7 | 2010 | 8.5 | Cu | 0.31 | FALSE | 0.25 | FALSE | 0.03 | FALSE | 0.45 | FALSE | 0.08 | FALSE |
| Msoor | 7 | 2010 | 10.6 | Ni | 0.29 | FALSE | 0.51 | FALSE | 0.2 | FALSE | NA | NA | NA | NA |
| Msoor | 7 | 2010 | 1.81 | Pb | 0.05 | FALSE | 0.04 | FALSE | 0.01 | FALSE | 0.06 | FALSE | 0.02 | FALSE |
| Msoor | 7 | 2010 | 24.9 | Zn | 0.2 | FALSE | 0.17 | FALSE | 0.06 | FALSE | 0.2 | FALSE | 0.09 | FALSE |
| Msoor | 7 | 2009 | 0.128 | Cd | 0.41 | FALSE | 0.11 | FALSE | 0.01 | FALSE | 0.18 | FALSE | 0.03 | FALSE |
| Msoor | 7 | 2009 | 48.6 | Cr | 0.6 | FALSE | 0.6 | FALSE | 0.13 | FALSE | 0.93 | FALSE | 0.3 | FALSE |
| Msoor | 7 | 2009 | 13.4 | Cu | 0.5 | FALSE | 0.4 | FALSE | 0.05 | FALSE | 0.72 | FALSE | 0.12 | FALSE |
| Msoor | 7 | 2009 | 20.1 | Ni | 0.56 | FALSE | 0.96 | FALSE | 0.39 | FALSE | NA | NA | NA | NA |
| Msoor | 7 | 2009 | 3.44 | Pb | 0.09 | FALSE | 0.07 | FALSE | 0.02 | FALSE | 0.11 | FALSE | 0.03 | FALSE |
| Msoor | 7 | 2009 | 22.9 | Zn | 0.19 | FALSE | 0.15 | FALSE | 0.06 | FALSE | 0.18 | FALSE | 0.08 | FALSE |
| Msoor | 7 | 2008 | 0.072 | Cd | 0.23 | FALSE | 0.06 | FALSE | 0.01 | FALSE | 0.1 | FALSE | 0.02 | FALSE |
| Msoor | 7 | 2008 | 16.5 | Cr | 0.2 | FALSE | 0.2 | FALSE | 0.04 | FALSE | 0.32 | FALSE | 0.1 | FALSE |
| Msoor | 7 | 2008 | 6.9 | Cu | 0.25 | FALSE | 0.2 | FALSE | 0.03 | FALSE | 0.37 | FALSE | 0.06 | FALSE |
| Msoor | 7 | 2008 | 1.2 | Ni | 0.03 | FALSE | 0.06 | FALSE | 0.02 | FALSE | NA | NA | NA | NA |
| Msoor | 7 | 2008 | 2.95 | Pb | 0.08 | FALSE | 0.06 | FALSE | 0.01 | FALSE | 0.1 | FALSE | 0.03 | FALSE |
| Msoor | 7 | 2008 | 2.4 | Zn | 0.02 | FALSE | 0.02 | FALSE | 0.01 | FALSE | 0.02 | FALSE | 0.01 | FALSE |
| Msoor | 7 | 2007 | 26.1 | Cr | 0.32 | FALSE | 0.32 | FALSE | 0.07 | FALSE | 0.5 | FALSE | 0.16 | FALSE |
| Msoor | 7 | 2007 | 10.5 | Cu | 0.39 | FALSE | 0.31 | FALSE | 0.04 | FALSE | 0.56 | FALSE | 0.1 | FALSE |
| Msoor | 7 | 2007 | 16.8 | Ni | 0.47 | FALSE | 0.8 | FALSE | 0.32 | FALSE | NA | NA | NA | NA |
| Msoor | 7 | 2007 | 2.59 | Pb | 0.07 | FALSE | 0.06 | FALSE | 0.01 | FALSE | 0.09 | FALSE | 0.02 | FALSE |
| Msoor | 7 | 2007 | 13 | Zn | 0.11 | FALSE | 0.09 | FALSE | 0.03 | FALSE | 0.1 | FALSE | 0.05 | FALSE |
| Refinery Area | 8 | 2020 | 0.193 | Cd | 0.62 | FALSE | 0.16 | FALSE | 0.02 | FALSE | 0.28 | FALSE | 0.05 | FALSE |
| Refinery Area | 8 | 2020 | 54.5 | Cr | 0.67 | FALSE | 0.67 | FALSE | 0.15 | FALSE | 1.04 | TRUE | 0.34 | FALSE |
| Refinery Area | 8 | 2020 | 64.2 | Cu | 2.38 | TRUE | 1.89 | TRUE | 0.24 | FALSE | 3.44 | TRUE | 0.59 | FALSE |
| Refinery Area | 8 | 2020 | 21.9 | Ni | 0.61 | FALSE | 1.05 | TRUE | 0.42 | FALSE | NA | NA | NA | NA |
| Refinery Area | 8 | 2020 | 159 | Pb | 4.19 | TRUE | 3.41 | TRUE | 0.73 | FALSE | 5.27 | TRUE | 1.42 | TRUE |
| Refinery Area | 8 | 2020 | 67 | Zn | 0.55 | FALSE | 0.45 | FALSE | 0.16 | FALSE | 0.54 | FALSE | 0.25 | FALSE |
| Refinery Area | 8 | 2019 | 0.309 | Cd | 1 | FALSE | 0.26 | FALSE | 0.03 | FALSE | 0.44 | FALSE | 0.07 | FALSE |
| Refinery Area | 8 | 2019 | 54 | Cr | 0.67 | FALSE | 0.67 | FALSE | 0.15 | FALSE | 1.03 | TRUE | 0.34 | FALSE |
| Refinery Area | 8 | 2019 | 50.6 | Cu | 1.87 | TRUE | 1.49 | TRUE | 0.19 | FALSE | 2.71 | TRUE | 0.47 | FALSE |
| Refinery Area | 8 | 2019 | 19 | Ni | 0.53 | FALSE | 0.91 | FALSE | 0.37 | FALSE | NA | NA | NA | NA |
| Refinery Area | 8 | 2019 | 151 | Pb | 3.96 | TRUE | 3.22 | TRUE | 0.69 | FALSE | 4.98 | TRUE | 1.34 | TRUE |
| Refinery Area | 8 | 2019 | 59.3 | Zn | 0.49 | FALSE | 0.4 | FALSE | 0.14 | FALSE | 0.48 | FALSE | 0.22 | FALSE |
| Refinery Area | 8 | 2018 | 0.13 | Cd | 0.42 | FALSE | 0.11 | FALSE | 0.01 | FALSE | 0.19 | FALSE | 0.03 | FALSE |
| Refinery Area | 8 | 2018 | 54.1 | Cr | 0.67 | FALSE | 0.67 | FALSE | 0.15 | FALSE | 1.04 | TRUE | 0.34 | FALSE |
| Refinery Area | 8 | 2018 | 48.6 | Cu | 1.8 | TRUE | 1.43 | TRUE | 0.18 | FALSE | 2.6 | TRUE | 0.45 | FALSE |
| Refinery Area | 8 | 2018 | 18.7 | Ni | 0.52 | FALSE | 0.9 | FALSE | 0.36 | FALSE | NA | NA | NA | NA |
| Refinery Area | 8 | 2018 | 105 | Pb | 2.75 | TRUE | 2.24 | TRUE | 0.48 | FALSE | 3.46 | TRUE | 0.93 | FALSE |
| Refinery Area | 8 | 2018 | 50.9 | Zn | 0.42 | FALSE | 0.34 | FALSE | 0.12 | FALSE | 0.41 | FALSE | 0.19 | FALSE |
| Refinery Area | 8 | 2017 | 0.121 | Cd | 0.39 | FALSE | 0.1 | FALSE | 0.01 | FALSE | 0.17 | FALSE | 0.03 | FALSE |
| Refinery Area | 8 | 2017 | 41.2 | Cr | 0.51 | FALSE | 0.51 | FALSE | 0.11 | FALSE | 0.79 | FALSE | 0.26 | FALSE |
| Refinery Area | 8 | 2017 | 45.3 | Cu | 1.68 | TRUE | 1.33 | TRUE | 0.17 | FALSE | 2.42 | TRUE | 0.42 | FALSE |
| Refinery Area | 8 | 2017 | 20.7 | Ni | 0.57 | FALSE | 0.99 | FALSE | 0.4 | FALSE | NA | NA | NA | NA |
| Refinery Area | 8 | 2017 | 64.6 | Pb | 1.7 | TRUE | 1.38 | TRUE | 0.3 | FALSE | 2.14 | TRUE | 0.58 | FALSE |
| Refinery Area | 8 | 2017 | 48.7 | Zn | 0.4 | FALSE | 0.32 | FALSE | 0.12 | FALSE | 0.39 | FALSE | 0.18 | FALSE |
| Refinery Area | 8 | 2016 | 0.176 | Cd | 0.57 | FALSE | 0.15 | FALSE | 0.02 | FALSE | 0.25 | FALSE | 0.04 | FALSE |
| Refinery Area | 8 | 2016 | 42.5 | Cr | 0.52 | FALSE | 0.52 | FALSE | 0.11 | FALSE | 0.81 | FALSE | 0.27 | FALSE |
| Refinery Area | 8 | 2016 | 56 | Cu | 2.07 | TRUE | 1.65 | TRUE | 0.21 | FALSE | 2.99 | TRUE | 0.52 | FALSE |
| Refinery Area | 8 | 2016 | 26.5 | Ni | 0.74 | FALSE | 1.27 | TRUE | 0.51 | FALSE | NA | NA | NA | NA |
| Refinery Area | 8 | 2016 | 97.3 | Pb | 2.56 | TRUE | 2.08 | TRUE | 0.45 | FALSE | 3.22 | TRUE | 0.87 | FALSE |
| Refinery Area | 8 | 2016 | 61.1 | Zn | 0.5 | FALSE | 0.41 | FALSE | 0.15 | FALSE | 0.49 | FALSE | 0.23 | FALSE |
| Refinery Area | 8 | 2015 | 0.166 | Cd | 0.54 | FALSE | 0.14 | FALSE | 0.02 | FALSE | 0.24 | FALSE | 0.04 | FALSE |
| Refinery Area | 8 | 2015 | 49.3 | Cr | 0.61 | FALSE | 0.61 | FALSE | 0.13 | FALSE | 0.94 | FALSE | 0.31 | FALSE |
| Refinery Area | 8 | 2015 | 57.1 | Cu | 2.11 | TRUE | 1.68 | TRUE | 0.21 | FALSE | 3.05 | TRUE | 0.53 | FALSE |
| Refinery Area | 8 | 2015 | 25.6 | Ni | 0.71 | FALSE | 1.22 | TRUE | 0.5 | FALSE | NA | NA | NA | NA |
| Refinery Area | 8 | 2015 | 72.6 | Pb | 1.91 | TRUE | 1.55 | TRUE | 0.33 | FALSE | 2.4 | TRUE | 0.65 | FALSE |
| Refinery Area | 8 | 2015 | 52.6 | Zn | 0.43 | FALSE | 0.35 | FALSE | 0.13 | FALSE | 0.42 | FALSE | 0.19 | FALSE |
| Refinery Area | 8 | 2014 | 0.283 | Cd | 0.91 | FALSE | 0.24 | FALSE | 0.03 | FALSE | 0.4 | FALSE | 0.07 | FALSE |
| Refinery Area | 8 | 2014 | 54.7 | Cr | 0.67 | FALSE | 0.67 | FALSE | 0.15 | FALSE | 1.05 | TRUE | 0.34 | FALSE |
| Refinery Area | 8 | 2014 | 57.5 | Cu | 2.13 | TRUE | 1.69 | TRUE | 0.21 | FALSE | 3.08 | TRUE | 0.53 | FALSE |
| Refinery Area | 8 | 2014 | 33.1 | Ni | 0.92 | FALSE | 1.59 | TRUE | 0.64 | FALSE | NA | NA | NA | NA |
| Refinery Area | 8 | 2014 | 56 | Pb | 1.47 | TRUE | 1.2 | TRUE | 0.26 | FALSE | 1.85 | TRUE | 0.5 | FALSE |
| Refinery Area | 8 | 2014 | 81.2 | Zn | 0.67 | FALSE | 0.54 | FALSE | 0.2 | FALSE | 0.65 | FALSE | 0.3 | FALSE |
| Refinery Area | 8 | 2013 | 0.243 | Cd | 0.78 | FALSE | 0.2 | FALSE | 0.03 | FALSE | 0.35 | FALSE | 0.06 | FALSE |
| Refinery Area | 8 | 2013 | 48.2 | Cr | 0.6 | FALSE | 0.6 | FALSE | 0.13 | FALSE | 0.92 | FALSE | 0.3 | FALSE |
| Refinery Area | 8 | 2013 | 56.8 | Cu | 2.1 | TRUE | 1.67 | TRUE | 0.21 | FALSE | 3.04 | TRUE | 0.53 | FALSE |
| Refinery Area | 8 | 2013 | 32 | Ni | 0.89 | FALSE | 1.53 | TRUE | 0.62 | FALSE | NA | NA | NA | NA |
| Refinery Area | 8 | 2013 | 161 | Pb | 4.23 | TRUE | 3.44 | TRUE | 0.74 | FALSE | 5.32 | TRUE | 1.43 | TRUE |
| Refinery Area | 8 | 2013 | 81.6 | Zn | 0.67 | FALSE | 0.54 | FALSE | 0.2 | FALSE | 0.66 | FALSE | 0.3 | FALSE |
| Refinery Area | 8 | 2012 | 0.282 | Cd | 0.91 | FALSE | 0.23 | FALSE | 0.03 | FALSE | 0.4 | FALSE | 0.07 | FALSE |
| Refinery Area | 8 | 2012 | 44.7 | Cr | 0.55 | FALSE | 0.55 | FALSE | 0.12 | FALSE | 0.86 | FALSE | 0.28 | FALSE |
| Refinery Area | 8 | 2012 | 56.9 | Cu | 2.11 | TRUE | 1.67 | TRUE | 0.21 | FALSE | 3.05 | TRUE | 0.53 | FALSE |
| Refinery Area | 8 | 2012 | 92.6 | Ni | 2.57 | TRUE | 4.43 | TRUE | 1.79 | TRUE | NA | NA | NA | NA |
| Refinery Area | 8 | 2012 | 107 | Pb | 2.82 | TRUE | 2.29 | TRUE | 0.49 | FALSE | 3.55 | TRUE | 0.96 | FALSE |
| Refinery Area | 8 | 2012 | 92.6 | Zn | 0.76 | FALSE | 0.62 | FALSE | 0.23 | FALSE | 0.75 | FALSE | 0.34 | FALSE |
| Refinery Area | 8 | 2011 | 0.19 | Cd | 0.61 | FALSE | 0.16 | FALSE | 0.02 | FALSE | 0.27 | FALSE | 0.05 | FALSE |
| Refinery Area | 8 | 2011 | 62.1 | Cr | 0.77 | FALSE | 0.77 | FALSE | 0.17 | FALSE | 1.19 | TRUE | 0.39 | FALSE |
| Refinery Area | 8 | 2011 | 62.2 | Cu | 2.3 | TRUE | 1.83 | TRUE | 0.23 | FALSE | 3.32 | TRUE | 0.58 | FALSE |
| Refinery Area | 8 | 2011 | 33 | Ni | 0.92 | FALSE | 1.58 | TRUE | 0.64 | FALSE | NA | NA | NA | NA |
| Refinery Area | 8 | 2011 | 188 | Pb | 4.95 | TRUE | 4.03 | TRUE | 0.86 | FALSE | 6.23 | TRUE | 1.68 | TRUE |
| Refinery Area | 8 | 2011 | 85.2 | Zn | 0.7 | FALSE | 0.57 | FALSE | 0.21 | FALSE | 0.69 | FALSE | 0.31 | FALSE |
| Refinery Area | 8 | 2010 | 0.154 | Cd | 0.5 | FALSE | 0.13 | FALSE | 0.02 | FALSE | 0.22 | FALSE | 0.04 | FALSE |
| Refinery Area | 8 | 2010 | 72.9 | Cr | 0.9 | FALSE | 0.9 | FALSE | 0.2 | FALSE | 1.39 | TRUE | 0.46 | FALSE |
| Refinery Area | 8 | 2010 | 45.5 | Cu | 1.69 | TRUE | 1.34 | TRUE | 0.17 | FALSE | 2.43 | TRUE | 0.42 | FALSE |
| Refinery Area | 8 | 2010 | 15.1 | Ni | 0.42 | FALSE | 0.72 | FALSE | 0.29 | FALSE | NA | NA | NA | NA |
| Refinery Area | 8 | 2010 | 118 | Pb | 3.1 | TRUE | 2.52 | TRUE | 0.54 | FALSE | 3.9 | TRUE | 1.05 | TRUE |
| Refinery Area | 8 | 2010 | 62.2 | Zn | 0.51 | FALSE | 0.41 | FALSE | 0.15 | FALSE | 0.5 | FALSE | 0.23 | FALSE |
| Refinery Area | 8 | 2009 | 0.185 | Cd | 0.6 | FALSE | 0.15 | FALSE | 0.02 | FALSE | 0.26 | FALSE | 0.04 | FALSE |
| Refinery Area | 8 | 2009 | 61.4 | Cr | 0.76 | FALSE | 0.76 | FALSE | 0.17 | FALSE | 1.17 | TRUE | 0.38 | FALSE |
| Refinery Area | 8 | 2009 | 63.3 | Cu | 2.34 | TRUE | 1.86 | TRUE | 0.23 | FALSE | 3.39 | TRUE | 0.59 | FALSE |
| Refinery Area | 8 | 2009 | 25.9 | Ni | 0.72 | FALSE | 1.24 | TRUE | 0.5 | FALSE | NA | NA | NA | NA |
| Refinery Area | 8 | 2009 | 213 | Pb | 5.61 | TRUE | 4.56 | TRUE | 0.98 | FALSE | 7.05 | TRUE | 1.9 | TRUE |
| Refinery Area | 8 | 2009 | 68 | Zn | 0.56 | FALSE | 0.45 | FALSE | 0.17 | FALSE | 0.55 | FALSE | 0.25 | FALSE |
| Refinery Area | 8 | 2008 | 0.06 | Cd | 0.19 | FALSE | 0.05 | FALSE | 0.01 | FALSE | 0.09 | FALSE | 0.01 | FALSE |
| Refinery Area | 8 | 2008 | 32.2 | Cr | 0.4 | FALSE | 0.4 | FALSE | 0.09 | FALSE | 0.62 | FALSE | 0.2 | FALSE |
| Refinery Area | 8 | 2008 | 52.1 | Cu | 1.93 | TRUE | 1.53 | TRUE | 0.19 | FALSE | 2.79 | TRUE | 0.48 | FALSE |
| Refinery Area | 8 | 2008 | 7.6 | Ni | 0.21 | FALSE | 0.37 | FALSE | 0.15 | FALSE | NA | NA | NA | NA |
| Refinery Area | 8 | 2008 | 146 | Pb | 3.83 | TRUE | 3.12 | TRUE | 0.67 | FALSE | 4.82 | TRUE | 1.3 | TRUE |
| Refinery Area | 8 | 2008 | 59.4 | Zn | 0.49 | FALSE | 0.4 | FALSE | 0.14 | FALSE | 0.48 | FALSE | 0.22 | FALSE |
| Refinery Area | 8 | 2007 | 53.5 | Cr | 0.66 | FALSE | 0.66 | FALSE | 0.14 | FALSE | 1.02 | TRUE | 0.33 | FALSE |
| Refinery Area | 8 | 2007 | 61.4 | Cu | 2.27 | TRUE | 1.81 | TRUE | 0.23 | FALSE | 3.28 | TRUE | 0.57 | FALSE |
| Refinery Area | 8 | 2007 | 27.7 | Ni | 0.77 | FALSE | 1.32 | TRUE | 0.54 | FALSE | NA | NA | NA | NA |
| Refinery Area | 8 | 2007 | 113 | Pb | 2.97 | TRUE | 2.42 | TRUE | 0.52 | FALSE | 3.74 | TRUE | 1.01 | TRUE |
| Refinery Area | 8 | 2007 | 70.2 | Zn | 0.58 | FALSE | 0.47 | FALSE | 0.17 | FALSE | 0.57 | FALSE | 0.26 | FALSE |
| Gaha | 9 | 2016 | 0.063 | Cd | 0.2 | FALSE | 0.05 | FALSE | 0.01 | FALSE | 0.09 | FALSE | 0.02 | FALSE |
| Gaha | 9 | 2016 | 84 | Cr | 1.04 | TRUE | 1.04 | TRUE | 0.23 | FALSE | 1.61 | TRUE | 0.52 | FALSE |
| Gaha | 9 | 2016 | 14.3 | Cu | 0.53 | FALSE | 0.42 | FALSE | 0.05 | FALSE | 0.77 | FALSE | 0.13 | FALSE |
| Gaha | 9 | 2016 | 6 | Ni | 0.17 | FALSE | 0.28 | FALSE | 0.12 | FALSE | NA | NA | NA | NA |
| Gaha | 9 | 2016 | 1.65 | Pb | 0.04 | FALSE | 0.04 | FALSE | 0.01 | FALSE | 0.05 | FALSE | 0.01 | FALSE |
| Gaha | 9 | 2016 | 15.8 | Zn | 0.13 | FALSE | 0.11 | FALSE | 0.04 | FALSE | 0.13 | FALSE | 0.06 | FALSE |
| Gaha | 9 | 2015 | 0.089 | Cd | 0.29 | FALSE | 0.07 | FALSE | 0.01 | FALSE | 0.13 | FALSE | 0.02 | FALSE |
| Gaha | 9 | 2015 | 82.7 | Cr | 1.02 | TRUE | 1.02 | TRUE | 0.22 | FALSE | 1.58 | TRUE | 0.52 | FALSE |
| Gaha | 9 | 2015 | 14.6 | Cu | 0.54 | FALSE | 0.43 | FALSE | 0.05 | FALSE | 0.78 | FALSE | 0.14 | FALSE |
| Gaha | 9 | 2015 | 11.4 | Ni | 0.32 | FALSE | 0.55 | FALSE | 0.22 | FALSE | NA | NA | NA | NA |
| Gaha | 9 | 2015 | 2.24 | Pb | 0.06 | FALSE | 0.05 | FALSE | 0.01 | FALSE | 0.07 | FALSE | 0.02 | FALSE |
| Gaha | 9 | 2015 | 11.1 | Zn | 0.09 | FALSE | 0.07 | FALSE | 0.03 | FALSE | 0.09 | FALSE | 0.04 | FALSE |
| Gaha | 9 | 2014 | 0.075 | Cd | 0.24 | FALSE | 0.06 | FALSE | 0.01 | FALSE | 0.11 | FALSE | 0.02 | FALSE |
| Gaha | 9 | 2014 | 26.9 | Cr | 0.33 | FALSE | 0.33 | FALSE | 0.07 | FALSE | 0.51 | FALSE | 0.17 | FALSE |
| Gaha | 9 | 2014 | 15.6 | Cu | 0.58 | FALSE | 0.46 | FALSE | 0.06 | FALSE | 0.84 | FALSE | 0.14 | FALSE |
| Gaha | 9 | 2014 | 5.5 | Ni | 0.15 | FALSE | 0.26 | FALSE | 0.11 | FALSE | NA | NA | NA | NA |
| Gaha | 9 | 2014 | 2.05 | Pb | 0.05 | FALSE | 0.04 | FALSE | 0.01 | FALSE | 0.07 | FALSE | 0.02 | FALSE |
| Gaha | 9 | 2014 | 20.5 | Zn | 0.17 | FALSE | 0.14 | FALSE | 0.05 | FALSE | 0.17 | FALSE | 0.08 | FALSE |
| Gaha | 9 | 2013 | 0.083 | Cd | 0.27 | FALSE | 0.07 | FALSE | 0.01 | FALSE | 0.12 | FALSE | 0.02 | FALSE |
| Gaha | 9 | 2013 | 23.8 | Cr | 0.29 | FALSE | 0.29 | FALSE | 0.06 | FALSE | 0.45 | FALSE | 0.15 | FALSE |
| Gaha | 9 | 2013 | 13.8 | Cu | 0.51 | FALSE | 0.41 | FALSE | 0.05 | FALSE | 0.74 | FALSE | 0.13 | FALSE |
| Gaha | 9 | 2013 | 10.7 | Ni | 0.3 | FALSE | 0.51 | FALSE | 0.21 | FALSE | NA | NA | NA | NA |
| Gaha | 9 | 2013 | 3.29 | Pb | 0.09 | FALSE | 0.07 | FALSE | 0.02 | FALSE | 0.11 | FALSE | 0.03 | FALSE |
| Gaha | 9 | 2013 | 18.9 | Zn | 0.15 | FALSE | 0.13 | FALSE | 0.05 | FALSE | 0.15 | FALSE | 0.07 | FALSE |
| Gaha | 9 | 2012 | 0.067 | Cd | 0.22 | FALSE | 0.06 | FALSE | 0.01 | FALSE | 0.1 | FALSE | 0.02 | FALSE |
| Gaha | 9 | 2012 | 44.4 | Cr | 0.55 | FALSE | 0.55 | FALSE | 0.12 | FALSE | 0.85 | FALSE | 0.28 | FALSE |
| Gaha | 9 | 2012 | 10.9 | Cu | 0.4 | FALSE | 0.32 | FALSE | 0.04 | FALSE | 0.58 | FALSE | 0.1 | FALSE |
| Gaha | 9 | 2012 | 22.1 | Ni | 0.62 | FALSE | 1.06 | TRUE | 0.43 | FALSE | NA | NA | NA | NA |
| Gaha | 9 | 2012 | 2.69 | Pb | 0.07 | FALSE | 0.06 | FALSE | 0.01 | FALSE | 0.09 | FALSE | 0.02 | FALSE |
| Gaha | 9 | 2012 | 22.1 | Zn | 0.18 | FALSE | 0.15 | FALSE | 0.05 | FALSE | 0.18 | FALSE | 0.08 | FALSE |
| Gaha | 9 | 2011 | 0.07 | Cd | 0.23 | FALSE | 0.06 | FALSE | 0.01 | FALSE | 0.1 | FALSE | 0.02 | FALSE |
| Gaha | 9 | 2011 | 35.5 | Cr | 0.44 | FALSE | 0.44 | FALSE | 0.1 | FALSE | 0.68 | FALSE | 0.22 | FALSE |
| Gaha | 9 | 2011 | 15.6 | Cu | 0.58 | FALSE | 0.46 | FALSE | 0.06 | FALSE | 0.83 | FALSE | 0.14 | FALSE |
| Gaha | 9 | 2011 | 18.8 | Ni | 0.52 | FALSE | 0.9 | FALSE | 0.36 | FALSE | NA | NA | NA | NA |
| Gaha | 9 | 2011 | 0.98 | Pb | 0.03 | FALSE | 0.02 | FALSE | 0 | FALSE | 0.03 | FALSE | 0.01 | FALSE |
| Gaha | 9 | 2011 | 34.4 | Zn | 0.28 | FALSE | 0.23 | FALSE | 0.08 | FALSE | 0.28 | FALSE | 0.13 | FALSE |
| Gaha | 9 | 2010 | 0.077 | Cd | 0.25 | FALSE | 0.06 | FALSE | 0.01 | FALSE | 0.11 | FALSE | 0.02 | FALSE |
| Gaha | 9 | 2010 | 36.4 | Cr | 0.45 | FALSE | 0.45 | FALSE | 0.1 | FALSE | 0.7 | FALSE | 0.23 | FALSE |
| Gaha | 9 | 2010 | 20.5 | Cu | 0.76 | FALSE | 0.6 | FALSE | 0.08 | FALSE | 1.1 | TRUE | 0.19 | FALSE |
| Gaha | 9 | 2010 | 12.1 | Ni | 0.34 | FALSE | 0.58 | FALSE | 0.23 | FALSE | NA | NA | NA | NA |
| Gaha | 9 | 2010 | 2.64 | Pb | 0.07 | FALSE | 0.06 | FALSE | 0.01 | FALSE | 0.09 | FALSE | 0.02 | FALSE |
| Gaha | 9 | 2010 | 23.1 | Zn | 0.19 | FALSE | 0.15 | FALSE | 0.06 | FALSE | 0.19 | FALSE | 0.09 | FALSE |
| Gaha | 9 | 2009 | 0.132 | Cd | 0.43 | FALSE | 0.11 | FALSE | 0.01 | FALSE | 0.19 | FALSE | 0.03 | FALSE |
| Gaha | 9 | 2009 | 41.4 | Cr | 0.51 | FALSE | 0.51 | FALSE | 0.11 | FALSE | 0.79 | FALSE | 0.26 | FALSE |
| Gaha | 9 | 2009 | 10 | Cu | 0.37 | FALSE | 0.29 | FALSE | 0.04 | FALSE | 0.54 | FALSE | 0.09 | FALSE |
| Gaha | 9 | 2009 | 8.4 | Ni | 0.23 | FALSE | 0.4 | FALSE | 0.16 | FALSE | NA | NA | NA | NA |
| Gaha | 9 | 2009 | 2.49 | Pb | 0.07 | FALSE | 0.05 | FALSE | 0.01 | FALSE | 0.08 | FALSE | 0.02 | FALSE |
| Gaha | 9 | 2009 | 21.4 | Zn | 0.18 | FALSE | 0.14 | FALSE | 0.05 | FALSE | 0.17 | FALSE | 0.08 | FALSE |
| Gaha | 9 | 2007 | 13.4 | Cr | 0.17 | FALSE | 0.17 | FALSE | 0.04 | FALSE | 0.26 | FALSE | 0.08 | FALSE |
| Gaha | 9 | 2007 | 10.8 | Cu | 0.4 | FALSE | 0.32 | FALSE | 0.04 | FALSE | 0.58 | FALSE | 0.1 | FALSE |
| Gaha | 9 | 2007 | 1.51 | Pb | 0.04 | FALSE | 0.03 | FALSE | 0.01 | FALSE | 0.05 | FALSE | 0.01 | FALSE |
| Suhain | 10 | 2016 | 0.03 | Cd | 0.1 | FALSE | 0.03 | FALSE | 0 | FALSE | 0.04 | FALSE | 0.01 | FALSE |
| Suhain | 10 | 2016 | 461 | Cr | 5.69 | TRUE | 5.69 | TRUE | 1.25 | TRUE | 8.81 | TRUE | 2.88 | TRUE |
| Suhain | 10 | 2016 | 29.9 | Cu | 1.11 | TRUE | 0.88 | FALSE | 0.11 | FALSE | 1.6 | TRUE | 0.28 | FALSE |
| Suhain | 10 | 2016 | 5.53 | Pb | 0.15 | FALSE | 0.12 | FALSE | 0.03 | FALSE | 0.18 | FALSE | 0.05 | FALSE |
| Suhain | 10 | 2016 | 17.6 | Zn | 0.14 | FALSE | 0.12 | FALSE | 0.04 | FALSE | 0.14 | FALSE | 0.06 | FALSE |
| Suhain | 10 | 2015 | 0.039 | Cd | 0.13 | FALSE | 0.03 | FALSE | 0 | FALSE | 0.06 | FALSE | 0.01 | FALSE |
| Suhain | 10 | 2015 | 85.6 | Cr | 1.06 | TRUE | 1.06 | TRUE | 0.23 | FALSE | 1.64 | TRUE | 0.54 | FALSE |
| Suhain | 10 | 2015 | 29.5 | Cu | 1.09 | TRUE | 0.87 | FALSE | 0.11 | FALSE | 1.58 | TRUE | 0.27 | FALSE |
| Suhain | 10 | 2015 | 17.9 | Pb | 0.47 | FALSE | 0.38 | FALSE | 0.08 | FALSE | 0.59 | FALSE | 0.16 | FALSE |
| Suhain | 10 | 2015 | 3.5 | Zn | 0.03 | FALSE | 0.02 | FALSE | 0.01 | FALSE | 0.03 | FALSE | 0.01 | FALSE |
| Suhain | 10 | 2014 | 0.125 | Cd | 0.4 | FALSE | 0.1 | FALSE | 0.01 | FALSE | 0.18 | FALSE | 0.03 | FALSE |
| Suhain | 10 | 2014 | 38 | Cr | 0.47 | FALSE | 0.47 | FALSE | 0.1 | FALSE | 0.73 | FALSE | 0.24 | FALSE |
| Suhain | 10 | 2014 | 37.4 | Cu | 1.39 | TRUE | 1.1 | TRUE | 0.14 | FALSE | 2 | TRUE | 0.35 | FALSE |
| Suhain | 10 | 2014 | 4.3 | Ni | 0.12 | FALSE | 0.21 | FALSE | 0.08 | FALSE | NA | NA | NA | NA |
| Suhain | 10 | 2014 | 6.66 | Pb | 0.18 | FALSE | 0.14 | FALSE | 0.03 | FALSE | 0.22 | FALSE | 0.06 | FALSE |
| Suhain | 10 | 2014 | 23 | Zn | 0.19 | FALSE | 0.15 | FALSE | 0.06 | FALSE | 0.19 | FALSE | 0.08 | FALSE |
| Suhain | 10 | 2013 | 0.079 | Cd | 0.25 | FALSE | 0.07 | FALSE | 0.01 | FALSE | 0.11 | FALSE | 0.02 | FALSE |
| Suhain | 10 | 2013 | 15 | Cr | 0.18 | FALSE | 0.18 | FALSE | 0.04 | FALSE | 0.29 | FALSE | 0.09 | FALSE |
| Suhain | 10 | 2013 | 30.5 | Cu | 1.13 | TRUE | 0.9 | FALSE | 0.11 | FALSE | 1.63 | TRUE | 0.28 | FALSE |
| Suhain | 10 | 2013 | 17.7 | Ni | 0.49 | FALSE | 0.85 | FALSE | 0.34 | FALSE | NA | NA | NA | NA |
| Suhain | 10 | 2013 | 4.64 | Pb | 0.12 | FALSE | 0.1 | FALSE | 0.02 | FALSE | 0.15 | FALSE | 0.04 | FALSE |
| Suhain | 10 | 2013 | 20.5 | Zn | 0.17 | FALSE | 0.14 | FALSE | 0.05 | FALSE | 0.17 | FALSE | 0.08 | FALSE |
| Suhain | 10 | 2012 | 0.114 | Cd | 0.37 | FALSE | 0.1 | FALSE | 0.01 | FALSE | 0.16 | FALSE | 0.03 | FALSE |
| Suhain | 10 | 2012 | 45.6 | Cr | 0.56 | FALSE | 0.56 | FALSE | 0.12 | FALSE | 0.87 | FALSE | 0.29 | FALSE |
| Suhain | 10 | 2012 | 31.6 | Cu | 1.17 | TRUE | 0.93 | FALSE | 0.12 | FALSE | 1.69 | TRUE | 0.29 | FALSE |
| Suhain | 10 | 2012 | 28.4 | Ni | 0.79 | FALSE | 1.36 | TRUE | 0.55 | FALSE | NA | NA | NA | NA |
| Suhain | 10 | 2012 | 37 | Pb | 0.97 | FALSE | 0.79 | FALSE | 0.17 | FALSE | 1.23 | TRUE | 0.33 | FALSE |
| Suhain | 10 | 2012 | 28.4 | Zn | 0.23 | FALSE | 0.19 | FALSE | 0.07 | FALSE | 0.23 | FALSE | 0.1 | FALSE |
| Suhain | 10 | 2011 | 0.076 | Cd | 0.25 | FALSE | 0.06 | FALSE | 0.01 | FALSE | 0.11 | FALSE | 0.02 | FALSE |
| Suhain | 10 | 2011 | 32.6 | Cr | 0.4 | FALSE | 0.4 | FALSE | 0.09 | FALSE | 0.62 | FALSE | 0.2 | FALSE |
| Suhain | 10 | 2011 | 18.7 | Cu | 0.69 | FALSE | 0.55 | FALSE | 0.07 | FALSE | 1 | FALSE | 0.17 | FALSE |
| Suhain | 10 | 2011 | 20.7 | Ni | 0.58 | FALSE | 0.99 | FALSE | 0.4 | FALSE | NA | NA | NA | NA |
| Suhain | 10 | 2011 | 0.99 | Pb | 0.03 | FALSE | 0.02 | FALSE | 0 | FALSE | 0.03 | FALSE | 0.01 | FALSE |
| Suhain | 10 | 2011 | 27.5 | Zn | 0.23 | FALSE | 0.18 | FALSE | 0.07 | FALSE | 0.22 | FALSE | 0.1 | FALSE |
| Suhain | 10 | 2010 | 0.173 | Cd | 0.56 | FALSE | 0.14 | FALSE | 0.02 | FALSE | 0.25 | FALSE | 0.04 | FALSE |
| Suhain | 10 | 2010 | 43.7 | Cr | 0.54 | FALSE | 0.54 | FALSE | 0.12 | FALSE | 0.84 | FALSE | 0.27 | FALSE |
| Suhain | 10 | 2010 | 29.9 | Cu | 1.11 | TRUE | 0.88 | FALSE | 0.11 | FALSE | 1.6 | TRUE | 0.28 | FALSE |
| Suhain | 10 | 2010 | 17.8 | Ni | 0.49 | FALSE | 0.85 | FALSE | 0.34 | FALSE | NA | NA | NA | NA |
| Suhain | 10 | 2010 | 3.24 | Pb | 0.09 | FALSE | 0.07 | FALSE | 0.01 | FALSE | 0.11 | FALSE | 0.03 | FALSE |
| Suhain | 10 | 2010 | 22.1 | Zn | 0.18 | FALSE | 0.15 | FALSE | 0.05 | FALSE | 0.18 | FALSE | 0.08 | FALSE |
| Suhain | 10 | 2009 | 0.136 | Cd | 0.44 | FALSE | 0.11 | FALSE | 0.01 | FALSE | 0.19 | FALSE | 0.03 | FALSE |
| Suhain | 10 | 2009 | 54.5 | Cr | 0.67 | FALSE | 0.67 | FALSE | 0.15 | FALSE | 1.04 | TRUE | 0.34 | FALSE |
| Suhain | 10 | 2009 | 25.3 | Cu | 0.94 | FALSE | 0.75 | FALSE | 0.09 | FALSE | 1.35 | TRUE | 0.23 | FALSE |
| Suhain | 10 | 2009 | 7.7 | Ni | 0.21 | FALSE | 0.37 | FALSE | 0.15 | FALSE | NA | NA | NA | NA |
| Suhain | 10 | 2009 | 2.44 | Pb | 0.06 | FALSE | 0.05 | FALSE | 0.01 | FALSE | 0.08 | FALSE | 0.02 | FALSE |
| Suhain | 10 | 2009 | 15.5 | Zn | 0.13 | FALSE | 0.1 | FALSE | 0.04 | FALSE | 0.13 | FALSE | 0.06 | FALSE |
| Suhain | 10 | 2008 | 0.014 | Cd | 0.05 | FALSE | 0.01 | FALSE | 0 | FALSE | 0.02 | FALSE | 0 | FALSE |
| Suhain | 10 | 2008 | 18.3 | Cr | 0.23 | FALSE | 0.23 | FALSE | 0.05 | FALSE | 0.35 | FALSE | 0.11 | FALSE |
| Suhain | 10 | 2008 | 1.5 | Cu | 0.06 | FALSE | 0.04 | FALSE | 0.01 | FALSE | 0.08 | FALSE | 0.01 | FALSE |
| Suhain | 10 | 2008 | 0.7 | Pb | 0.02 | FALSE | 0.01 | FALSE | 0 | FALSE | 0.02 | FALSE | 0.01 | FALSE |
| Suhain | 10 | 2007 | 9.6 | Cr | 0.12 | FALSE | 0.12 | FALSE | 0.03 | FALSE | 0.18 | FALSE | 0.06 | FALSE |
| Suhain | 10 | 2007 | 12.6 | Cu | 0.47 | FALSE | 0.37 | FALSE | 0.05 | FALSE | 0.67 | FALSE | 0.12 | FALSE |
| Suhain | 10 | 2007 | 1.45 | Pb | 0.04 | FALSE | 0.03 | FALSE | 0.01 | FALSE | 0.05 | FALSE | 0.01 | FALSE |
| Suhain | 10 | 2007 | 0.6 | Zn | 0 | FALSE | 0 | FALSE | 0 | FALSE | 0 | FALSE | 0 | FALSE |
| Duwaimil | 11 | 2015 | 0.065 | Cd | 0.21 | FALSE | 0.05 | FALSE | 0.01 | FALSE | 0.09 | FALSE | 0.02 | FALSE |
| Duwaimil | 11 | 2015 | 98.3 | Cr | 1.21 | TRUE | 1.21 | TRUE | 0.27 | FALSE | 1.88 | TRUE | 0.61 | FALSE |
| Duwaimil | 11 | 2015 | 17.4 | Cu | 0.64 | FALSE | 0.51 | FALSE | 0.06 | FALSE | 0.93 | FALSE | 0.16 | FALSE |
| Duwaimil | 11 | 2015 | 4 | Ni | 0.11 | FALSE | 0.19 | FALSE | 0.08 | FALSE | NA | NA | NA | NA |
| Duwaimil | 11 | 2015 | 7.83 | Pb | 0.21 | FALSE | 0.17 | FALSE | 0.04 | FALSE | 0.26 | FALSE | 0.07 | FALSE |
| Duwaimil | 11 | 2015 | 10.2 | Zn | 0.08 | FALSE | 0.07 | FALSE | 0.02 | FALSE | 0.08 | FALSE | 0.04 | FALSE |
| Duwaimil | 11 | 2014 | 0.149 | Cd | 0.48 | FALSE | 0.12 | FALSE | 0.02 | FALSE | 0.21 | FALSE | 0.04 | FALSE |
| Duwaimil | 11 | 2014 | 31.6 | Cr | 0.39 | FALSE | 0.39 | FALSE | 0.09 | FALSE | 0.6 | FALSE | 0.2 | FALSE |
| Duwaimil | 11 | 2014 | 63.3 | Cu | 2.35 | TRUE | 1.86 | TRUE | 0.23 | FALSE | 3.39 | TRUE | 0.59 | FALSE |
| Duwaimil | 11 | 2014 | 23.7 | Ni | 0.66 | FALSE | 1.13 | TRUE | 0.46 | FALSE | NA | NA | NA | NA |
| Duwaimil | 11 | 2014 | 3.81 | Pb | 0.1 | FALSE | 0.08 | FALSE | 0.02 | FALSE | 0.13 | FALSE | 0.03 | FALSE |
| Duwaimil | 11 | 2014 | 24.6 | Zn | 0.2 | FALSE | 0.16 | FALSE | 0.06 | FALSE | 0.2 | FALSE | 0.09 | FALSE |
| Duwaimil | 11 | 2013 | 0.088 | Cd | 0.28 | FALSE | 0.07 | FALSE | 0.01 | FALSE | 0.13 | FALSE | 0.02 | FALSE |
| Duwaimil | 11 | 2013 | 22 | Cr | 0.27 | FALSE | 0.27 | FALSE | 0.06 | FALSE | 0.42 | FALSE | 0.14 | FALSE |
| Duwaimil | 11 | 2013 | 33.5 | Cu | 1.24 | TRUE | 0.98 | FALSE | 0.12 | FALSE | 1.79 | TRUE | 0.31 | FALSE |
| Duwaimil | 11 | 2013 | 16 | Ni | 0.44 | FALSE | 0.77 | FALSE | 0.31 | FALSE | NA | NA | NA | NA |
| Duwaimil | 11 | 2013 | 4.51 | Pb | 0.12 | FALSE | 0.1 | FALSE | 0.02 | FALSE | 0.15 | FALSE | 0.04 | FALSE |
| Duwaimil | 11 | 2013 | 17.8 | Zn | 0.15 | FALSE | 0.12 | FALSE | 0.04 | FALSE | 0.14 | FALSE | 0.07 | FALSE |
| Gazara | 12 | 2020 | 0.12 | Cd | 0.39 | FALSE | 0.1 | FALSE | 0.01 | FALSE | 0.17 | FALSE | 0.03 | FALSE |
| Gazara | 12 | 2020 | 48.9 | Cr | 0.6 | FALSE | 0.6 | FALSE | 0.13 | FALSE | 0.93 | FALSE | 0.31 | FALSE |
| Gazara | 12 | 2020 | 9.2 | Cu | 0.34 | FALSE | 0.27 | FALSE | 0.03 | FALSE | 0.49 | FALSE | 0.08 | FALSE |
| Gazara | 12 | 2020 | 20.3 | Ni | 0.56 | FALSE | 0.97 | FALSE | 0.39 | FALSE | NA | NA | NA | NA |
| Gazara | 12 | 2020 | 1.68 | Pb | 0.04 | FALSE | 0.04 | FALSE | 0.01 | FALSE | 0.06 | FALSE | 0.01 | FALSE |
| Gazara | 12 | 2020 | 21.5 | Zn | 0.18 | FALSE | 0.14 | FALSE | 0.05 | FALSE | 0.17 | FALSE | 0.08 | FALSE |
| Gazara | 12 | 2019 | 0.074 | Cd | 0.24 | FALSE | 0.06 | FALSE | 0.01 | FALSE | 0.11 | FALSE | 0.02 | FALSE |
| Gazara | 12 | 2019 | 49.5 | Cr | 0.61 | FALSE | 0.61 | FALSE | 0.13 | FALSE | 0.95 | FALSE | 0.31 | FALSE |
| Gazara | 12 | 2019 | 9.7 | Cu | 0.36 | FALSE | 0.29 | FALSE | 0.04 | FALSE | 0.52 | FALSE | 0.09 | FALSE |
| Gazara | 12 | 2019 | 21.6 | Ni | 0.6 | FALSE | 1.03 | TRUE | 0.42 | FALSE | NA | NA | NA | NA |
| Gazara | 12 | 2019 | 3.23 | Pb | 0.09 | FALSE | 0.07 | FALSE | 0.01 | FALSE | 0.11 | FALSE | 0.03 | FALSE |
| Gazara | 12 | 2019 | 22.7 | Zn | 0.19 | FALSE | 0.15 | FALSE | 0.06 | FALSE | 0.18 | FALSE | 0.08 | FALSE |
| Gazara | 12 | 2018 | 0.096 | Cd | 0.31 | FALSE | 0.08 | FALSE | 0.01 | FALSE | 0.14 | FALSE | 0.02 | FALSE |
| Gazara | 12 | 2018 | 54.6 | Cr | 0.67 | FALSE | 0.67 | FALSE | 0.15 | FALSE | 1.04 | TRUE | 0.34 | FALSE |
| Gazara | 12 | 2018 | 13.6 | Cu | 0.5 | FALSE | 0.4 | FALSE | 0.05 | FALSE | 0.73 | FALSE | 0.13 | FALSE |
| Gazara | 12 | 2018 | 20.7 | Ni | 0.57 | FALSE | 0.99 | FALSE | 0.4 | FALSE | NA | NA | NA | NA |
| Gazara | 12 | 2018 | 1.23 | Pb | 0.03 | FALSE | 0.03 | FALSE | 0.01 | FALSE | 0.04 | FALSE | 0.01 | FALSE |
| Gazara | 12 | 2018 | 21.1 | Zn | 0.17 | FALSE | 0.14 | FALSE | 0.05 | FALSE | 0.17 | FALSE | 0.08 | FALSE |
| Gazara | 12 | 2017 | 0.093 | Cd | 0.3 | FALSE | 0.08 | FALSE | 0.01 | FALSE | 0.13 | FALSE | 0.02 | FALSE |
| Gazara | 12 | 2017 | 10.5 | Cu | 0.39 | FALSE | 0.31 | FALSE | 0.04 | FALSE | 0.56 | FALSE | 0.1 | FALSE |
| Gazara | 12 | 2017 | 34 | Ni | 0.94 | FALSE | 1.63 | TRUE | 0.66 | FALSE | NA | NA | NA | NA |
| Gazara | 12 | 2017 | 1.51 | Pb | 0.04 | FALSE | 0.03 | FALSE | 0.01 | FALSE | 0.05 | FALSE | 0.01 | FALSE |
| Gazara | 12 | 2017 | 20.3 | Zn | 0.17 | FALSE | 0.14 | FALSE | 0.05 | FALSE | 0.16 | FALSE | 0.08 | FALSE |
| Gazara | 12 | 2016 | 0.069 | Cd | 0.22 | FALSE | 0.06 | FALSE | 0.01 | FALSE | 0.1 | FALSE | 0.02 | FALSE |
| Gazara | 12 | 2016 | 13.7 | Cu | 0.51 | FALSE | 0.4 | FALSE | 0.05 | FALSE | 0.73 | FALSE | 0.13 | FALSE |
| Gazara | 12 | 2016 | 21.4 | Ni | 0.59 | FALSE | 1.02 | TRUE | 0.41 | FALSE | NA | NA | NA | NA |
| Gazara | 12 | 2016 | 1.58 | Pb | 0.04 | FALSE | 0.03 | FALSE | 0.01 | FALSE | 0.05 | FALSE | 0.01 | FALSE |
| Gazara | 12 | 2016 | 21.4 | Zn | 0.18 | FALSE | 0.14 | FALSE | 0.05 | FALSE | 0.17 | FALSE | 0.08 | FALSE |
| Gazara | 12 | 2015 | 0.092 | Cd | 0.3 | FALSE | 0.08 | FALSE | 0.01 | FALSE | 0.13 | FALSE | 0.02 | FALSE |
| Gazara | 12 | 2015 | 14.4 | Cu | 0.53 | FALSE | 0.42 | FALSE | 0.05 | FALSE | 0.77 | FALSE | 0.13 | FALSE |
| Gazara | 12 | 2015 | 15.5 | Ni | 0.43 | FALSE | 0.74 | FALSE | 0.3 | FALSE | NA | NA | NA | NA |
| Gazara | 12 | 2015 | 2.3 | Pb | 0.06 | FALSE | 0.05 | FALSE | 0.01 | FALSE | 0.08 | FALSE | 0.02 | FALSE |
| Gazara | 12 | 2015 | 14.2 | Zn | 0.12 | FALSE | 0.09 | FALSE | 0.03 | FALSE | 0.11 | FALSE | 0.05 | FALSE |
| Gazara | 12 | 2014 | 0.14 | Cd | 0.45 | FALSE | 0.12 | FALSE | 0.01 | FALSE | 0.2 | FALSE | 0.03 | FALSE |
| Gazara | 12 | 2014 | 11.4 | Cu | 0.42 | FALSE | 0.33 | FALSE | 0.04 | FALSE | 0.61 | FALSE | 0.11 | FALSE |
| Gazara | 12 | 2014 | 37.4 | Ni | 1.04 | TRUE | 1.79 | TRUE | 0.73 | FALSE | NA | NA | NA | NA |
| Gazara | 12 | 2014 | 3.24 | Pb | 0.09 | FALSE | 0.07 | FALSE | 0.01 | FALSE | 0.11 | FALSE | 0.03 | FALSE |
| Gazara | 12 | 2014 | 22.6 | Zn | 0.19 | FALSE | 0.15 | FALSE | 0.06 | FALSE | 0.18 | FALSE | 0.08 | FALSE |
| Gazara | 12 | 2013 | 0.083 | Cd | 0.27 | FALSE | 0.07 | FALSE | 0.01 | FALSE | 0.12 | FALSE | 0.02 | FALSE |
| Gazara | 12 | 2013 | 47.7 | Cr | 0.59 | FALSE | 0.59 | FALSE | 0.13 | FALSE | 0.91 | FALSE | 0.3 | FALSE |
| Gazara | 12 | 2013 | 11.7 | Cu | 0.43 | FALSE | 0.34 | FALSE | 0.04 | FALSE | 0.63 | FALSE | 0.11 | FALSE |
| Gazara | 12 | 2013 | 22.4 | Ni | 0.62 | FALSE | 1.07 | TRUE | 0.43 | FALSE | NA | NA | NA | NA |
| Gazara | 12 | 2013 | 3.63 | Pb | 0.1 | FALSE | 0.08 | FALSE | 0.02 | FALSE | 0.12 | FALSE | 0.03 | FALSE |
| Gazara | 12 | 2013 | 17.6 | Zn | 0.14 | FALSE | 0.12 | FALSE | 0.04 | FALSE | 0.14 | FALSE | 0.07 | FALSE |
| Gazara | 12 | 2012 | 0.094 | Cd | 0.3 | FALSE | 0.08 | FALSE | 0.01 | FALSE | 0.13 | FALSE | 0.02 | FALSE |
| Gazara | 12 | 2012 | 56 | Cr | 0.69 | FALSE | 0.69 | FALSE | 0.15 | FALSE | 1.07 | TRUE | 0.35 | FALSE |
| Gazara | 12 | 2012 | 7.4 | Cu | 0.27 | FALSE | 0.22 | FALSE | 0.03 | FALSE | 0.39 | FALSE | 0.07 | FALSE |
| Gazara | 12 | 2012 | 24.6 | Ni | 0.68 | FALSE | 1.18 | TRUE | 0.48 | FALSE | NA | NA | NA | NA |
| Gazara | 12 | 2012 | 2.24 | Pb | 0.06 | FALSE | 0.05 | FALSE | 0.01 | FALSE | 0.07 | FALSE | 0.02 | FALSE |
| Gazara | 12 | 2012 | 24.6 | Zn | 0.2 | FALSE | 0.16 | FALSE | 0.06 | FALSE | 0.2 | FALSE | 0.09 | FALSE |
| Gazara | 12 | 2011 | 0.082 | Cd | 0.26 | FALSE | 0.07 | FALSE | 0.01 | FALSE | 0.12 | FALSE | 0.02 | FALSE |
| Gazara | 12 | 2011 | 49.4 | Cr | 0.61 | FALSE | 0.61 | FALSE | 0.13 | FALSE | 0.94 | FALSE | 0.31 | FALSE |
| Gazara | 12 | 2011 | 13.9 | Cu | 0.51 | FALSE | 0.41 | FALSE | 0.05 | FALSE | 0.74 | FALSE | 0.13 | FALSE |
| Gazara | 12 | 2011 | 29.6 | Ni | 0.82 | FALSE | 1.42 | TRUE | 0.57 | FALSE | NA | NA | NA | NA |
| Gazara | 12 | 2011 | 0.66 | Pb | 0.02 | FALSE | 0.01 | FALSE | 0 | FALSE | 0.02 | FALSE | 0.01 | FALSE |
| Gazara | 12 | 2011 | 36.7 | Zn | 0.3 | FALSE | 0.24 | FALSE | 0.09 | FALSE | 0.3 | FALSE | 0.14 | FALSE |
| Gazara | 12 | 2010 | 0.08 | Cd | 0.26 | FALSE | 0.07 | FALSE | 0.01 | FALSE | 0.11 | FALSE | 0.02 | FALSE |
| Gazara | 12 | 2010 | 62.4 | Cr | 0.77 | FALSE | 0.77 | FALSE | 0.17 | FALSE | 1.19 | TRUE | 0.39 | FALSE |
| Gazara | 12 | 2010 | 15.4 | Cu | 0.57 | FALSE | 0.45 | FALSE | 0.06 | FALSE | 0.82 | FALSE | 0.14 | FALSE |
| Gazara | 12 | 2010 | 23.5 | Ni | 0.65 | FALSE | 1.12 | TRUE | 0.46 | FALSE | NA | NA | NA | NA |
| Gazara | 12 | 2010 | 2.22 | Pb | 0.06 | FALSE | 0.05 | FALSE | 0.01 | FALSE | 0.07 | FALSE | 0.02 | FALSE |
| Gazara | 12 | 2010 | 20.6 | Zn | 0.17 | FALSE | 0.14 | FALSE | 0.05 | FALSE | 0.17 | FALSE | 0.08 | FALSE |
| Gazara | 12 | 2009 | 0.074 | Cd | 0.24 | FALSE | 0.06 | FALSE | 0.01 | FALSE | 0.11 | FALSE | 0.02 | FALSE |
| Gazara | 12 | 2009 | 61.1 | Cr | 0.75 | FALSE | 0.75 | FALSE | 0.17 | FALSE | 1.17 | TRUE | 0.38 | FALSE |
| Gazara | 12 | 2009 | 7.9 | Cu | 0.29 | FALSE | 0.23 | FALSE | 0.03 | FALSE | 0.42 | FALSE | 0.07 | FALSE |
| Gazara | 12 | 2009 | 22.4 | Ni | 0.62 | FALSE | 1.07 | TRUE | 0.43 | FALSE | NA | NA | NA | NA |
| Gazara | 12 | 2009 | 2.22 | Pb | 0.06 | FALSE | 0.05 | FALSE | 0.01 | FALSE | 0.07 | FALSE | 0.02 | FALSE |
| Gazara | 12 | 2009 | 17.8 | Zn | 0.15 | FALSE | 0.12 | FALSE | 0.04 | FALSE | 0.14 | FALSE | 0.07 | FALSE |
| Dam | 13 | 2016 | 0.051 | Cd | 0.17 | FALSE | 0.04 | FALSE | 0.01 | FALSE | 0.07 | FALSE | 0.01 | FALSE |
| Dam | 13 | 2016 | 103 | Cr | 1.27 | TRUE | 1.27 | TRUE | 0.28 | FALSE | 1.97 | TRUE | 0.64 | FALSE |
| Dam | 13 | 2016 | 13.8 | Cu | 0.51 | FALSE | 0.4 | FALSE | 0.05 | FALSE | 0.74 | FALSE | 0.13 | FALSE |
| Dam | 13 | 2016 | 17.3 | Ni | 0.48 | FALSE | 0.83 | FALSE | 0.34 | FALSE | NA | NA | NA | NA |
| Dam | 13 | 2016 | 1.69 | Pb | 0.04 | FALSE | 0.04 | FALSE | 0.01 | FALSE | 0.06 | FALSE | 0.02 | FALSE |
| Dam | 13 | 2016 | 13.5 | Zn | 0.11 | FALSE | 0.09 | FALSE | 0.03 | FALSE | 0.11 | FALSE | 0.05 | FALSE |
| Dam | 13 | 2015 | 0.088 | Cd | 0.29 | FALSE | 0.07 | FALSE | 0.01 | FALSE | 0.13 | FALSE | 0.02 | FALSE |
| Dam | 13 | 2015 | 45.1 | Cr | 0.56 | FALSE | 0.56 | FALSE | 0.12 | FALSE | 0.86 | FALSE | 0.28 | FALSE |
| Dam | 13 | 2015 | 14.1 | Cu | 0.52 | FALSE | 0.41 | FALSE | 0.05 | FALSE | 0.75 | FALSE | 0.13 | FALSE |
| Dam | 13 | 2015 | 36.8 | Ni | 1.02 | TRUE | 1.76 | TRUE | 0.71 | FALSE | NA | NA | NA | NA |
| Dam | 13 | 2015 | 2.77 | Pb | 0.07 | FALSE | 0.06 | FALSE | 0.01 | FALSE | 0.09 | FALSE | 0.02 | FALSE |
| Dam | 13 | 2015 | 11.8 | Zn | 0.1 | FALSE | 0.08 | FALSE | 0.03 | FALSE | 0.1 | FALSE | 0.04 | FALSE |
| Dam | 13 | 2014 | 0.125 | Cd | 0.4 | FALSE | 0.1 | FALSE | 0.01 | FALSE | 0.18 | FALSE | 0.03 | FALSE |
| Dam | 13 | 2014 | 48.8 | Cr | 0.6 | FALSE | 0.6 | FALSE | 0.13 | FALSE | 0.93 | FALSE | 0.31 | FALSE |
| Dam | 13 | 2014 | 15 | Cu | 0.56 | FALSE | 0.44 | FALSE | 0.06 | FALSE | 0.8 | FALSE | 0.14 | FALSE |
| Dam | 13 | 2014 | 33.7 | Ni | 0.93 | FALSE | 1.61 | TRUE | 0.65 | FALSE | NA | NA | NA | NA |
| Dam | 13 | 2014 | 2.39 | Pb | 0.06 | FALSE | 0.05 | FALSE | 0.01 | FALSE | 0.08 | FALSE | 0.02 | FALSE |
| Dam | 13 | 2014 | 16.3 | Zn | 0.13 | FALSE | 0.11 | FALSE | 0.04 | FALSE | 0.13 | FALSE | 0.06 | FALSE |
| Dam | 13 | 2013 | 0.07 | Cd | 0.23 | FALSE | 0.06 | FALSE | 0.01 | FALSE | 0.1 | FALSE | 0.02 | FALSE |
| Dam | 13 | 2013 | 24.6 | Cr | 0.3 | FALSE | 0.3 | FALSE | 0.07 | FALSE | 0.47 | FALSE | 0.15 | FALSE |
| Dam | 13 | 2013 | 28.2 | Cu | 1.04 | TRUE | 0.83 | FALSE | 0.1 | FALSE | 1.51 | TRUE | 0.26 | FALSE |
| Dam | 13 | 2013 | 12.5 | Ni | 0.35 | FALSE | 0.6 | FALSE | 0.24 | FALSE | NA | NA | NA | NA |
| Dam | 13 | 2013 | 5.37 | Pb | 0.14 | FALSE | 0.12 | FALSE | 0.02 | FALSE | 0.18 | FALSE | 0.05 | FALSE |
| Dam | 13 | 2013 | 9.6 | Zn | 0.08 | FALSE | 0.06 | FALSE | 0.02 | FALSE | 0.08 | FALSE | 0.04 | FALSE |
| Dam | 13 | 2011 | 0.1 | Cd | 0.32 | FALSE | 0.08 | FALSE | 0.01 | FALSE | 0.14 | FALSE | 0.02 | FALSE |
| Dam | 13 | 2011 | 56.5 | Cr | 0.7 | FALSE | 0.7 | FALSE | 0.15 | FALSE | 1.08 | TRUE | 0.35 | FALSE |
| Dam | 13 | 2011 | 32.5 | Cu | 1.21 | TRUE | 0.96 | FALSE | 0.12 | FALSE | 1.74 | TRUE | 0.3 | FALSE |
| Dam | 13 | 2011 | 31.5 | Ni | 0.88 | FALSE | 1.51 | TRUE | 0.61 | FALSE | NA | NA | NA | NA |
| Dam | 13 | 2011 | 1.74 | Pb | 0.05 | FALSE | 0.04 | FALSE | 0.01 | FALSE | 0.06 | FALSE | 0.02 | FALSE |
| Dam | 13 | 2011 | 36.5 | Zn | 0.3 | FALSE | 0.24 | FALSE | 0.09 | FALSE | 0.29 | FALSE | 0.13 | FALSE |
| Dam | 13 | 2010 | 0.252 | Cd | 0.81 | FALSE | 0.21 | FALSE | 0.03 | FALSE | 0.36 | FALSE | 0.06 | FALSE |
| Dam | 13 | 2010 | 67.3 | Cr | 0.83 | FALSE | 0.83 | FALSE | 0.18 | FALSE | 1.29 | TRUE | 0.42 | FALSE |
| Dam | 13 | 2010 | 37.8 | Cu | 1.4 | TRUE | 1.11 | TRUE | 0.14 | FALSE | 2.02 | TRUE | 0.35 | FALSE |
| Dam | 13 | 2010 | 16.9 | Ni | 0.47 | FALSE | 0.81 | FALSE | 0.33 | FALSE | NA | NA | NA | NA |
| Dam | 13 | 2010 | 1.87 | Pb | 0.05 | FALSE | 0.04 | FALSE | 0.01 | FALSE | 0.06 | FALSE | 0.02 | FALSE |
| Dam | 13 | 2010 | 13.7 | Zn | 0.11 | FALSE | 0.09 | FALSE | 0.03 | FALSE | 0.11 | FALSE | 0.05 | FALSE |
| Dam | 13 | 2009 | 0.193 | Cd | 0.62 | FALSE | 0.16 | FALSE | 0.02 | FALSE | 0.28 | FALSE | 0.05 | FALSE |
| Dam | 13 | 2009 | 90.9 | Cr | 1.12 | TRUE | 1.12 | TRUE | 0.25 | FALSE | 1.74 | TRUE | 0.57 | FALSE |
| Dam | 13 | 2009 | 15 | Cu | 0.56 | FALSE | 0.44 | FALSE | 0.06 | FALSE | 0.8 | FALSE | 0.14 | FALSE |
| Dam | 13 | 2009 | 25.3 | Ni | 0.7 | FALSE | 1.21 | TRUE | 0.49 | FALSE | NA | NA | NA | NA |
| Dam | 13 | 2009 | 2.45 | Pb | 0.06 | FALSE | 0.05 | FALSE | 0.01 | FALSE | 0.08 | FALSE | 0.02 | FALSE |
| Dam | 13 | 2009 | 9.1 | Zn | 0.07 | FALSE | 0.06 | FALSE | 0.02 | FALSE | 0.07 | FALSE | 0.03 | FALSE |
| Jaradah | 14 | 2020 | 0.08 | Cd | 0.26 | FALSE | 0.07 | FALSE | 0.01 | FALSE | 0.11 | FALSE | 0.02 | FALSE |
| Jaradah | 14 | 2020 | 37.5 | Cr | 0.46 | FALSE | 0.46 | FALSE | 0.1 | FALSE | 0.72 | FALSE | 0.23 | FALSE |
| Jaradah | 14 | 2020 | 4.9 | Cu | 0.18 | FALSE | 0.14 | FALSE | 0.02 | FALSE | 0.26 | FALSE | 0.05 | FALSE |
| Jaradah | 14 | 2020 | 10.7 | Ni | 0.3 | FALSE | 0.51 | FALSE | 0.21 | FALSE | NA | NA | NA | NA |
| Jaradah | 14 | 2020 | 0.77 | Pb | 0.02 | FALSE | 0.02 | FALSE | 0 | FALSE | 0.03 | FALSE | 0.01 | FALSE |
| Jaradah | 14 | 2020 | 14.8 | Zn | 0.12 | FALSE | 0.1 | FALSE | 0.04 | FALSE | 0.12 | FALSE | 0.05 | FALSE |
| Jaradah | 14 | 2019 | 0.08 | Cd | 0.26 | FALSE | 0.07 | FALSE | 0.01 | FALSE | 0.11 | FALSE | 0.02 | FALSE |
| Jaradah | 14 | 2019 | 30.7 | Cr | 0.38 | FALSE | 0.38 | FALSE | 0.08 | FALSE | 0.59 | FALSE | 0.19 | FALSE |
| Jaradah | 14 | 2019 | 4.2 | Cu | 0.16 | FALSE | 0.12 | FALSE | 0.02 | FALSE | 0.22 | FALSE | 0.04 | FALSE |
| Jaradah | 14 | 2019 | 10.8 | Ni | 0.3 | FALSE | 0.52 | FALSE | 0.21 | FALSE | NA | NA | NA | NA |
| Jaradah | 14 | 2019 | 2.26 | Pb | 0.06 | FALSE | 0.05 | FALSE | 0.01 | FALSE | 0.07 | FALSE | 0.02 | FALSE |
| Jaradah | 14 | 2019 | 14.3 | Zn | 0.12 | FALSE | 0.1 | FALSE | 0.03 | FALSE | 0.12 | FALSE | 0.05 | FALSE |
| Jaradah | 14 | 2018 | 0.096 | Cd | 0.31 | FALSE | 0.08 | FALSE | 0.01 | FALSE | 0.14 | FALSE | 0.02 | FALSE |
| Jaradah | 14 | 2018 | 44.7 | Cr | 0.55 | FALSE | 0.55 | FALSE | 0.12 | FALSE | 0.85 | FALSE | 0.28 | FALSE |
| Jaradah | 14 | 2018 | 14.6 | Cu | 0.54 | FALSE | 0.43 | FALSE | 0.05 | FALSE | 0.78 | FALSE | 0.14 | FALSE |
| Jaradah | 14 | 2018 | 13.7 | Ni | 0.38 | FALSE | 0.65 | FALSE | 0.27 | FALSE | NA | NA | NA | NA |
| Jaradah | 14 | 2018 | 0.92 | Pb | 0.02 | FALSE | 0.02 | FALSE | 0 | FALSE | 0.03 | FALSE | 0.01 | FALSE |
| Jaradah | 14 | 2018 | 16.7 | Zn | 0.14 | FALSE | 0.11 | FALSE | 0.04 | FALSE | 0.13 | FALSE | 0.06 | FALSE |
| Jaradah | 14 | 2017 | 0.093 | Cd | 0.3 | FALSE | 0.08 | FALSE | 0.01 | FALSE | 0.13 | FALSE | 0.02 | FALSE |
| Jaradah | 14 | 2017 | 50.7 | Cr | 0.63 | FALSE | 0.63 | FALSE | 0.14 | FALSE | 0.97 | FALSE | 0.32 | FALSE |
| Jaradah | 14 | 2017 | 6 | Cu | 0.22 | FALSE | 0.18 | FALSE | 0.02 | FALSE | 0.32 | FALSE | 0.06 | FALSE |
| Jaradah | 14 | 2017 | 11 | Ni | 0.31 | FALSE | 0.53 | FALSE | 0.21 | FALSE | NA | NA | NA | NA |
| Jaradah | 14 | 2017 | 1.06 | Pb | 0.03 | FALSE | 0.02 | FALSE | 0 | FALSE | 0.04 | FALSE | 0.01 | FALSE |
| Jaradah | 14 | 2017 | 11.9 | Zn | 0.1 | FALSE | 0.08 | FALSE | 0.03 | FALSE | 0.1 | FALSE | 0.04 | FALSE |
| Jaradah | 14 | 2016 | 0.068 | Cd | 0.22 | FALSE | 0.06 | FALSE | 0.01 | FALSE | 0.1 | FALSE | 0.02 | FALSE |
| Jaradah | 14 | 2016 | 40.5 | Cr | 0.5 | FALSE | 0.5 | FALSE | 0.11 | FALSE | 0.78 | FALSE | 0.25 | FALSE |
| Jaradah | 14 | 2016 | 10.9 | Cu | 0.4 | FALSE | 0.32 | FALSE | 0.04 | FALSE | 0.58 | FALSE | 0.1 | FALSE |
| Jaradah | 14 | 2016 | 8.4 | Ni | 0.23 | FALSE | 0.4 | FALSE | 0.16 | FALSE | NA | NA | NA | NA |
| Jaradah | 14 | 2016 | 0.96 | Pb | 0.03 | FALSE | 0.02 | FALSE | 0 | FALSE | 0.03 | FALSE | 0.01 | FALSE |
| Jaradah | 14 | 2016 | 13.5 | Zn | 0.11 | FALSE | 0.09 | FALSE | 0.03 | FALSE | 0.11 | FALSE | 0.05 | FALSE |
| Jaradah | 14 | 2015 | 0.161 | Cd | 0.52 | FALSE | 0.13 | FALSE | 0.02 | FALSE | 0.23 | FALSE | 0.04 | FALSE |
| Jaradah | 14 | 2015 | 29.1 | Cr | 0.36 | FALSE | 0.36 | FALSE | 0.08 | FALSE | 0.56 | FALSE | 0.18 | FALSE |
| Jaradah | 14 | 2015 | 11.1 | Cu | 0.41 | FALSE | 0.33 | FALSE | 0.04 | FALSE | 0.59 | FALSE | 0.1 | FALSE |
| Jaradah | 14 | 2015 | 19.9 | Ni | 0.55 | FALSE | 0.95 | FALSE | 0.39 | FALSE | NA | NA | NA | NA |
| Jaradah | 14 | 2015 | 2.11 | Pb | 0.06 | FALSE | 0.05 | FALSE | 0.01 | FALSE | 0.07 | FALSE | 0.02 | FALSE |
| Jaradah | 14 | 2015 | 8.3 | Zn | 0.07 | FALSE | 0.06 | FALSE | 0.02 | FALSE | 0.07 | FALSE | 0.03 | FALSE |
| Jaradah | 14 | 2014 | 0.151 | Cd | 0.49 | FALSE | 0.13 | FALSE | 0.02 | FALSE | 0.22 | FALSE | 0.04 | FALSE |
| Jaradah | 14 | 2014 | 42.4 | Cr | 0.52 | FALSE | 0.52 | FALSE | 0.11 | FALSE | 0.81 | FALSE | 0.26 | FALSE |
| Jaradah | 14 | 2014 | 9.1 | Cu | 0.34 | FALSE | 0.27 | FALSE | 0.03 | FALSE | 0.49 | FALSE | 0.08 | FALSE |
| Jaradah | 14 | 2014 | 22.8 | Ni | 0.63 | FALSE | 1.09 | TRUE | 0.44 | FALSE | NA | NA | NA | NA |
| Jaradah | 14 | 2014 | 1.87 | Pb | 0.05 | FALSE | 0.04 | FALSE | 0.01 | FALSE | 0.06 | FALSE | 0.02 | FALSE |
| Jaradah | 14 | 2014 | 17.1 | Zn | 0.14 | FALSE | 0.11 | FALSE | 0.04 | FALSE | 0.14 | FALSE | 0.06 | FALSE |
| Jaradah | 14 | 2013 | 0.074 | Cd | 0.24 | FALSE | 0.06 | FALSE | 0.01 | FALSE | 0.11 | FALSE | 0.02 | FALSE |
| Jaradah | 14 | 2013 | 23.7 | Cr | 0.29 | FALSE | 0.29 | FALSE | 0.06 | FALSE | 0.45 | FALSE | 0.15 | FALSE |
| Jaradah | 14 | 2013 | 9.5 | Cu | 0.35 | FALSE | 0.28 | FALSE | 0.04 | FALSE | 0.51 | FALSE | 0.09 | FALSE |
| Jaradah | 14 | 2013 | 14 | Ni | 0.39 | FALSE | 0.67 | FALSE | 0.27 | FALSE | NA | NA | NA | NA |
| Jaradah | 14 | 2013 | 1.96 | Pb | 0.05 | FALSE | 0.04 | FALSE | 0.01 | FALSE | 0.07 | FALSE | 0.02 | FALSE |
| Jaradah | 14 | 2013 | 3.4 | Zn | 0.03 | FALSE | 0.02 | FALSE | 0.01 | FALSE | 0.03 | FALSE | 0.01 | FALSE |
| Jaradah | 14 | 2012 | 0.115 | Cd | 0.37 | FALSE | 0.1 | FALSE | 0.01 | FALSE | 0.16 | FALSE | 0.03 | FALSE |
| Jaradah | 14 | 2012 | 41.4 | Cr | 0.51 | FALSE | 0.51 | FALSE | 0.11 | FALSE | 0.79 | FALSE | 0.26 | FALSE |
| Jaradah | 14 | 2012 | 6.4 | Cu | 0.24 | FALSE | 0.19 | FALSE | 0.02 | FALSE | 0.34 | FALSE | 0.06 | FALSE |
| Jaradah | 14 | 2012 | 14.3 | Ni | 0.4 | FALSE | 0.69 | FALSE | 0.28 | FALSE | NA | NA | NA | NA |
| Jaradah | 14 | 2012 | 3.4 | Pb | 0.09 | FALSE | 0.07 | FALSE | 0.02 | FALSE | 0.11 | FALSE | 0.03 | FALSE |
| Jaradah | 14 | 2012 | 14.3 | Zn | 0.12 | FALSE | 0.1 | FALSE | 0.03 | FALSE | 0.12 | FALSE | 0.05 | FALSE |
| Jaradah | 14 | 2011 | 0.082 | Cd | 0.26 | FALSE | 0.07 | FALSE | 0.01 | FALSE | 0.12 | FALSE | 0.02 | FALSE |
| Jaradah | 14 | 2011 | 37 | Cr | 0.46 | FALSE | 0.46 | FALSE | 0.1 | FALSE | 0.71 | FALSE | 0.23 | FALSE |
| Jaradah | 14 | 2011 | 12.7 | Cu | 0.47 | FALSE | 0.37 | FALSE | 0.05 | FALSE | 0.68 | FALSE | 0.12 | FALSE |
| Jaradah | 14 | 2011 | 34.1 | Ni | 0.95 | FALSE | 1.63 | TRUE | 0.66 | FALSE | NA | NA | NA | NA |
| Jaradah | 14 | 2011 | 0.33 | Pb | 0.01 | FALSE | 0.01 | FALSE | 0 | FALSE | 0.01 | FALSE | 0 | FALSE |
| Jaradah | 14 | 2011 | 33.6 | Zn | 0.28 | FALSE | 0.22 | FALSE | 0.08 | FALSE | 0.27 | FALSE | 0.12 | FALSE |
| Jaradah | 14 | 2010 | 0.073 | Cd | 0.23 | FALSE | 0.06 | FALSE | 0.01 | FALSE | 0.1 | FALSE | 0.02 | FALSE |
| Jaradah | 14 | 2010 | 35.7 | Cr | 0.44 | FALSE | 0.44 | FALSE | 0.1 | FALSE | 0.68 | FALSE | 0.22 | FALSE |
| Jaradah | 14 | 2010 | 12.2 | Cu | 0.45 | FALSE | 0.36 | FALSE | 0.05 | FALSE | 0.65 | FALSE | 0.11 | FALSE |
| Jaradah | 14 | 2010 | 20.5 | Ni | 0.57 | FALSE | 0.98 | FALSE | 0.4 | FALSE | NA | NA | NA | NA |
| Jaradah | 14 | 2010 | 0.42 | Pb | 0.01 | FALSE | 0.01 | FALSE | 0 | FALSE | 0.01 | FALSE | 0 | FALSE |
| Jaradah | 14 | 2010 | 11 | Zn | 0.09 | FALSE | 0.07 | FALSE | 0.03 | FALSE | 0.09 | FALSE | 0.04 | FALSE |
| Jaradah | 14 | 2009 | 0.105 | Cd | 0.34 | FALSE | 0.09 | FALSE | 0.01 | FALSE | 0.15 | FALSE | 0.02 | FALSE |
| Jaradah | 14 | 2009 | 62 | Cr | 0.77 | FALSE | 0.77 | FALSE | 0.17 | FALSE | 1.19 | TRUE | 0.39 | FALSE |
| Jaradah | 14 | 2009 | 9.8 | Cu | 0.36 | FALSE | 0.29 | FALSE | 0.04 | FALSE | 0.52 | FALSE | 0.09 | FALSE |
| Jaradah | 14 | 2009 | 15.7 | Ni | 0.44 | FALSE | 0.75 | FALSE | 0.3 | FALSE | NA | NA | NA | NA |
| Jaradah | 14 | 2009 | 1.58 | Pb | 0.04 | FALSE | 0.03 | FALSE | 0.01 | FALSE | 0.05 | FALSE | 0.01 | FALSE |
| Jaradah | 14 | 2009 | 7.7 | Zn | 0.06 | FALSE | 0.05 | FALSE | 0.02 | FALSE | 0.06 | FALSE | 0.03 | FALSE |
| Jaradah | 14 | 2008 | 0.079 | Cd | 0.25 | FALSE | 0.07 | FALSE | 0.01 | FALSE | 0.11 | FALSE | 0.02 | FALSE |
| Jaradah | 14 | 2008 | 232 | Cr | 2.86 | TRUE | 2.86 | TRUE | 0.63 | FALSE | 4.43 | TRUE | 1.45 | TRUE |
| Jaradah | 14 | 2008 | 5.4 | Cu | 0.2 | FALSE | 0.16 | FALSE | 0.02 | FALSE | 0.29 | FALSE | 0.05 | FALSE |
| Jaradah | 14 | 2008 | 13.4 | Ni | 0.37 | FALSE | 0.64 | FALSE | 0.26 | FALSE | NA | NA | NA | NA |
| Jaradah | 14 | 2008 | 2.94 | Pb | 0.08 | FALSE | 0.06 | FALSE | 0.01 | FALSE | 0.1 | FALSE | 0.03 | FALSE |
| Jaradah | 14 | 2008 | 1.7 | Zn | 0.01 | FALSE | 0.01 | FALSE | 0 | FALSE | 0.01 | FALSE | 0.01 | FALSE |
| Jaradah | 14 | 2007 | 99.7 | Cr | 1.23 | TRUE | 1.23 | TRUE | 0.27 | FALSE | 1.91 | TRUE | 0.62 | FALSE |
| Jaradah | 14 | 2007 | 12.1 | Cu | 0.45 | FALSE | 0.35 | FALSE | 0.04 | FALSE | 0.64 | FALSE | 0.11 | FALSE |
| Jaradah | 14 | 2007 | 1.84 | Pb | 0.05 | FALSE | 0.04 | FALSE | 0.01 | FALSE | 0.06 | FALSE | 0.02 | FALSE |
| Jaradah | 14 | 2007 | 2.4 | Zn | 0.02 | FALSE | 0.02 | FALSE | 0.01 | FALSE | 0.02 | FALSE | 0.01 | FALSE |
| Jetty 1 | 15 | 2014 | 0.177 | Cd | 0.57 | FALSE | 0.15 | FALSE | 0.02 | FALSE | 0.25 | FALSE | 0.04 | FALSE |
| Jetty 1 | 15 | 2014 | 47.7 | Cr | 0.59 | FALSE | 0.59 | FALSE | 0.13 | FALSE | 0.91 | FALSE | 0.3 | FALSE |
| Jetty 1 | 15 | 2014 | 57.3 | Cu | 2.12 | TRUE | 1.68 | TRUE | 0.21 | FALSE | 3.06 | TRUE | 0.53 | FALSE |
| Jetty 1 | 15 | 2014 | 55.6 | Ni | 1.54 | TRUE | 2.66 | TRUE | 1.08 | TRUE | NA | NA | NA | NA |
| Jetty 1 | 15 | 2014 | 6.81 | Pb | 0.18 | FALSE | 0.15 | FALSE | 0.03 | FALSE | 0.23 | FALSE | 0.06 | FALSE |
| Jetty 1 | 15 | 2014 | 91.8 | Zn | 0.75 | FALSE | 0.61 | FALSE | 0.22 | FALSE | 0.74 | FALSE | 0.34 | FALSE |
| Jetty 1 | 15 | 2013 | 0.153 | Cd | 0.49 | FALSE | 0.13 | FALSE | 0.02 | FALSE | 0.22 | FALSE | 0.04 | FALSE |
| Jetty 1 | 15 | 2013 | 34.7 | Cr | 0.43 | FALSE | 0.43 | FALSE | 0.09 | FALSE | 0.66 | FALSE | 0.22 | FALSE |
| Jetty 1 | 15 | 2013 | 46.4 | Cu | 1.72 | TRUE | 1.36 | TRUE | 0.17 | FALSE | 2.48 | TRUE | 0.43 | FALSE |
| Jetty 1 | 15 | 2013 | 20.9 | Ni | 0.58 | FALSE | 1 | FALSE | 0.4 | FALSE | NA | NA | NA | NA |
| Jetty 1 | 15 | 2013 | 9.73 | Pb | 0.26 | FALSE | 0.21 | FALSE | 0.04 | FALSE | 0.32 | FALSE | 0.09 | FALSE |
| Jetty 1 | 15 | 2013 | 72.9 | Zn | 0.6 | FALSE | 0.49 | FALSE | 0.18 | FALSE | 0.59 | FALSE | 0.27 | FALSE |
| Jetty 1 | 15 | 2012 | 0.161 | Cd | 0.52 | FALSE | 0.13 | FALSE | 0.02 | FALSE | 0.23 | FALSE | 0.04 | FALSE |
| Jetty 1 | 15 | 2012 | 35.8 | Cr | 0.44 | FALSE | 0.44 | FALSE | 0.1 | FALSE | 0.69 | FALSE | 0.22 | FALSE |
| Jetty 1 | 15 | 2012 | 44 | Cu | 1.63 | TRUE | 1.29 | TRUE | 0.16 | FALSE | 2.35 | TRUE | 0.41 | FALSE |
| Jetty 1 | 15 | 2012 | 79.4 | Ni | 2.2 | TRUE | 3.8 | TRUE | 1.54 | TRUE | NA | NA | NA | NA |
| Jetty 1 | 15 | 2012 | 6.66 | Pb | 0.18 | FALSE | 0.14 | FALSE | 0.03 | FALSE | 0.22 | FALSE | 0.06 | FALSE |
| Jetty 1 | 15 | 2012 | 79.4 | Zn | 0.65 | FALSE | 0.53 | FALSE | 0.19 | FALSE | 0.64 | FALSE | 0.29 | FALSE |
| Jetty 1 | 15 | 2011 | 0.057 | Cd | 0.18 | FALSE | 0.05 | FALSE | 0.01 | FALSE | 0.08 | FALSE | 0.01 | FALSE |
| Jetty 1 | 15 | 2011 | 34 | Cr | 0.42 | FALSE | 0.42 | FALSE | 0.09 | FALSE | 0.65 | FALSE | 0.21 | FALSE |
| Jetty 1 | 15 | 2011 | 77.6 | Cu | 2.87 | TRUE | 2.28 | TRUE | 0.29 | FALSE | 4.15 | TRUE | 0.72 | FALSE |
| Jetty 1 | 15 | 2011 | 29.2 | Ni | 0.81 | FALSE | 1.4 | TRUE | 0.57 | FALSE | NA | NA | NA | NA |
| Jetty 1 | 15 | 2011 | 7.45 | Pb | 0.2 | FALSE | 0.16 | FALSE | 0.03 | FALSE | 0.25 | FALSE | 0.07 | FALSE |
| Jetty 1 | 15 | 2011 | 92.1 | Zn | 0.75 | FALSE | 0.61 | FALSE | 0.22 | FALSE | 0.74 | FALSE | 0.34 | FALSE |
| Jetty 1 | 15 | 2010 | 0.135 | Cd | 0.43 | FALSE | 0.11 | FALSE | 0.01 | FALSE | 0.19 | FALSE | 0.03 | FALSE |
| Jetty 1 | 15 | 2010 | 69 | Cr | 0.85 | FALSE | 0.85 | FALSE | 0.19 | FALSE | 1.32 | TRUE | 0.43 | FALSE |
| Jetty 1 | 15 | 2010 | 43.9 | Cu | 1.62 | TRUE | 1.29 | TRUE | 0.16 | FALSE | 2.35 | TRUE | 0.41 | FALSE |
| Jetty 1 | 15 | 2010 | 13.5 | Ni | 0.37 | FALSE | 0.65 | FALSE | 0.26 | FALSE | NA | NA | NA | NA |
| Jetty 1 | 15 | 2010 | 16.2 | Pb | 0.43 | FALSE | 0.35 | FALSE | 0.07 | FALSE | 0.54 | FALSE | 0.14 | FALSE |
| Jetty 1 | 15 | 2010 | 74.4 | Zn | 0.61 | FALSE | 0.5 | FALSE | 0.18 | FALSE | 0.6 | FALSE | 0.27 | FALSE |
| Jetty 1 | 15 | 2009 | 0.196 | Cd | 0.63 | FALSE | 0.16 | FALSE | 0.02 | FALSE | 0.28 | FALSE | 0.05 | FALSE |
| Jetty 1 | 15 | 2009 | 47.6 | Cr | 0.59 | FALSE | 0.59 | FALSE | 0.13 | FALSE | 0.91 | FALSE | 0.3 | FALSE |
| Jetty 1 | 15 | 2009 | 43.6 | Cu | 1.61 | TRUE | 1.28 | TRUE | 0.16 | FALSE | 2.33 | TRUE | 0.4 | FALSE |
| Jetty 1 | 15 | 2009 | 21.4 | Ni | 0.6 | FALSE | 1.03 | TRUE | 0.42 | FALSE | NA | NA | NA | NA |
| Jetty 1 | 15 | 2009 | 52.8 | Pb | 1.39 | TRUE | 1.13 | TRUE | 0.24 | FALSE | 1.75 | TRUE | 0.47 | FALSE |
| Jetty 1 | 15 | 2009 | 77 | Zn | 0.63 | FALSE | 0.51 | FALSE | 0.19 | FALSE | 0.62 | FALSE | 0.28 | FALSE |
| Jetty 1 | 15 | 2008 | 0.171 | Cd | 0.55 | FALSE | 0.14 | FALSE | 0.02 | FALSE | 0.24 | FALSE | 0.04 | FALSE |
| Jetty 1 | 15 | 2008 | 19.8 | Cr | 0.25 | FALSE | 0.25 | FALSE | 0.05 | FALSE | 0.38 | FALSE | 0.12 | FALSE |
| Jetty 1 | 15 | 2008 | 46.5 | Cu | 1.72 | TRUE | 1.37 | TRUE | 0.17 | FALSE | 2.48 | TRUE | 0.43 | FALSE |
| Jetty 1 | 15 | 2008 | 13.4 | Ni | 0.37 | FALSE | 0.64 | FALSE | 0.26 | FALSE | NA | NA | NA | NA |
| Jetty 1 | 15 | 2008 | 12.6 | Pb | 0.33 | FALSE | 0.27 | FALSE | 0.06 | FALSE | 0.42 | FALSE | 0.11 | FALSE |
| Jetty 1 | 15 | 2008 | 81.8 | Zn | 0.67 | FALSE | 0.55 | FALSE | 0.2 | FALSE | 0.66 | FALSE | 0.3 | FALSE |
| Jetty 1 | 15 | 2007 | 42.7 | Cr | 0.53 | FALSE | 0.53 | FALSE | 0.12 | FALSE | 0.82 | FALSE | 0.27 | FALSE |
| Jetty 1 | 15 | 2007 | 48 | Cu | 1.78 | TRUE | 1.41 | TRUE | 0.18 | FALSE | 2.57 | TRUE | 0.44 | FALSE |
| Jetty 1 | 15 | 2007 | 23.9 | Ni | 0.66 | FALSE | 1.14 | TRUE | 0.46 | FALSE | NA | NA | NA | NA |
| Jetty 1 | 15 | 2007 | 10.4 | Pb | 0.27 | FALSE | 0.22 | FALSE | 0.05 | FALSE | 0.35 | FALSE | 0.09 | FALSE |
| Jetty 1 | 15 | 2007 | 84.6 | Zn | 0.69 | FALSE | 0.56 | FALSE | 0.21 | FALSE | 0.68 | FALSE | 0.31 | FALSE |
| Al-Jarim | 16 | 2020 | 0.065 | Cd | 0.21 | FALSE | 0.05 | FALSE | 0.01 | FALSE | 0.09 | FALSE | 0.02 | FALSE |
| Al-Jarim | 16 | 2020 | 52.9 | Cr | 0.65 | FALSE | 0.65 | FALSE | 0.14 | FALSE | 1.01 | TRUE | 0.33 | FALSE |
| Al-Jarim | 16 | 2020 | 4.7 | Cu | 0.17 | FALSE | 0.14 | FALSE | 0.02 | FALSE | 0.25 | FALSE | 0.04 | FALSE |
| Al-Jarim | 16 | 2020 | 11.1 | Ni | 0.31 | FALSE | 0.53 | FALSE | 0.21 | FALSE | NA | NA | NA | NA |
| Al-Jarim | 16 | 2020 | 0.9 | Pb | 0.02 | FALSE | 0.02 | FALSE | 0 | FALSE | 0.03 | FALSE | 0.01 | FALSE |
| Al-Jarim | 16 | 2020 | 17.2 | Zn | 0.14 | FALSE | 0.11 | FALSE | 0.04 | FALSE | 0.14 | FALSE | 0.06 | FALSE |
| Al-Jarim | 16 | 2019 | 0.08 | Cd | 0.26 | FALSE | 0.07 | FALSE | 0.01 | FALSE | 0.11 | FALSE | 0.02 | FALSE |
| Al-Jarim | 16 | 2019 | 58.3 | Cr | 0.72 | FALSE | 0.72 | FALSE | 0.16 | FALSE | 1.11 | TRUE | 0.36 | FALSE |
| Al-Jarim | 16 | 2019 | 5.8 | Cu | 0.21 | FALSE | 0.17 | FALSE | 0.02 | FALSE | 0.31 | FALSE | 0.05 | FALSE |
| Al-Jarim | 16 | 2019 | 15.4 | Ni | 0.43 | FALSE | 0.74 | FALSE | 0.3 | FALSE | NA | NA | NA | NA |
| Al-Jarim | 16 | 2019 | 2.11 | Pb | 0.06 | FALSE | 0.05 | FALSE | 0.01 | FALSE | 0.07 | FALSE | 0.02 | FALSE |
| Al-Jarim | 16 | 2019 | 17.4 | Zn | 0.14 | FALSE | 0.12 | FALSE | 0.04 | FALSE | 0.14 | FALSE | 0.06 | FALSE |
| Al-Jarim | 16 | 2018 | 0.079 | Cd | 0.26 | FALSE | 0.07 | FALSE | 0.01 | FALSE | 0.11 | FALSE | 0.02 | FALSE |
| Al-Jarim | 16 | 2018 | 74 | Cr | 0.91 | FALSE | 0.91 | FALSE | 0.2 | FALSE | 1.41 | TRUE | 0.46 | FALSE |
| Al-Jarim | 16 | 2018 | 92 | Cu | 3.41 | TRUE | 2.71 | TRUE | 0.34 | FALSE | 4.92 | TRUE | 0.85 | FALSE |
| Al-Jarim | 16 | 2018 | 12.3 | Ni | 0.34 | FALSE | 0.59 | FALSE | 0.24 | FALSE | NA | NA | NA | NA |
| Al-Jarim | 16 | 2018 | 0.88 | Pb | 0.02 | FALSE | 0.02 | FALSE | 0 | FALSE | 0.03 | FALSE | 0.01 | FALSE |
| Al-Jarim | 16 | 2018 | 15.2 | Zn | 0.12 | FALSE | 0.1 | FALSE | 0.04 | FALSE | 0.12 | FALSE | 0.06 | FALSE |
| Al-Jarim | 16 | 2017 | 0.078 | Cd | 0.25 | FALSE | 0.07 | FALSE | 0.01 | FALSE | 0.11 | FALSE | 0.02 | FALSE |
| Al-Jarim | 16 | 2017 | 97.6 | Cr | 1.21 | TRUE | 1.21 | TRUE | 0.26 | FALSE | 1.87 | TRUE | 0.61 | FALSE |
| Al-Jarim | 16 | 2017 | 6.1 | Cu | 0.23 | FALSE | 0.18 | FALSE | 0.02 | FALSE | 0.33 | FALSE | 0.06 | FALSE |
| Al-Jarim | 16 | 2017 | 20.8 | Ni | 0.58 | FALSE | 0.99 | FALSE | 0.4 | FALSE | NA | NA | NA | NA |
| Al-Jarim | 16 | 2017 | 1.29 | Pb | 0.03 | FALSE | 0.03 | FALSE | 0.01 | FALSE | 0.04 | FALSE | 0.01 | FALSE |
| Al-Jarim | 16 | 2017 | 13.8 | Zn | 0.11 | FALSE | 0.09 | FALSE | 0.03 | FALSE | 0.11 | FALSE | 0.05 | FALSE |
| Al-Jarim | 16 | 2016 | 0.087 | Cd | 0.28 | FALSE | 0.07 | FALSE | 0.01 | FALSE | 0.12 | FALSE | 0.02 | FALSE |
| Al-Jarim | 16 | 2016 | 64.1 | Cr | 0.79 | FALSE | 0.79 | FALSE | 0.17 | FALSE | 1.23 | TRUE | 0.4 | FALSE |
| Al-Jarim | 16 | 2016 | 10 | Cu | 0.37 | FALSE | 0.3 | FALSE | 0.04 | FALSE | 0.54 | FALSE | 0.09 | FALSE |
| Al-Jarim | 16 | 2016 | 12.6 | Ni | 0.35 | FALSE | 0.6 | FALSE | 0.24 | FALSE | NA | NA | NA | NA |
| Al-Jarim | 16 | 2016 | 1.02 | Pb | 0.03 | FALSE | 0.02 | FALSE | 0 | FALSE | 0.03 | FALSE | 0.01 | FALSE |
| Al-Jarim | 16 | 2016 | 14 | Zn | 0.11 | FALSE | 0.09 | FALSE | 0.03 | FALSE | 0.11 | FALSE | 0.05 | FALSE |
| Al-Jarim | 16 | 2015 | 0.073 | Cd | 0.24 | FALSE | 0.06 | FALSE | 0.01 | FALSE | 0.1 | FALSE | 0.02 | FALSE |
| Al-Jarim | 16 | 2015 | 52.7 | Cr | 0.65 | FALSE | 0.65 | FALSE | 0.14 | FALSE | 1.01 | TRUE | 0.33 | FALSE |
| Al-Jarim | 16 | 2015 | 10.3 | Cu | 0.38 | FALSE | 0.3 | FALSE | 0.04 | FALSE | 0.55 | FALSE | 0.1 | FALSE |
| Al-Jarim | 16 | 2015 | 21.2 | Ni | 0.59 | FALSE | 1.01 | TRUE | 0.41 | FALSE | NA | NA | NA | NA |
| Al-Jarim | 16 | 2015 | 1.93 | Pb | 0.05 | FALSE | 0.04 | FALSE | 0.01 | FALSE | 0.06 | FALSE | 0.02 | FALSE |
| Al-Jarim | 16 | 2015 | 9.1 | Zn | 0.07 | FALSE | 0.06 | FALSE | 0.02 | FALSE | 0.07 | FALSE | 0.03 | FALSE |
| Al-Jarim | 16 | 2014 | 0.102 | Cd | 0.33 | FALSE | 0.08 | FALSE | 0.01 | FALSE | 0.15 | FALSE | 0.02 | FALSE |
| Al-Jarim | 16 | 2014 | 50.8 | Cr | 0.63 | FALSE | 0.63 | FALSE | 0.14 | FALSE | 0.97 | FALSE | 0.32 | FALSE |
| Al-Jarim | 16 | 2014 | 10.8 | Cu | 0.4 | FALSE | 0.32 | FALSE | 0.04 | FALSE | 0.58 | FALSE | 0.1 | FALSE |
| Al-Jarim | 16 | 2014 | 30.9 | Ni | 0.86 | FALSE | 1.48 | TRUE | 0.6 | FALSE | NA | NA | NA | NA |
| Al-Jarim | 16 | 2014 | 1.53 | Pb | 0.04 | FALSE | 0.03 | FALSE | 0.01 | FALSE | 0.05 | FALSE | 0.01 | FALSE |
| Al-Jarim | 16 | 2014 | 15.9 | Zn | 0.13 | FALSE | 0.11 | FALSE | 0.04 | FALSE | 0.13 | FALSE | 0.06 | FALSE |
| Al-Jarim | 16 | 2013 | 0.086 | Cd | 0.28 | FALSE | 0.07 | FALSE | 0.01 | FALSE | 0.12 | FALSE | 0.02 | FALSE |
| Al-Jarim | 16 | 2013 | 42.8 | Cr | 0.53 | FALSE | 0.53 | FALSE | 0.12 | FALSE | 0.82 | FALSE | 0.27 | FALSE |
| Al-Jarim | 16 | 2013 | 11.9 | Cu | 0.44 | FALSE | 0.35 | FALSE | 0.04 | FALSE | 0.64 | FALSE | 0.11 | FALSE |
| Al-Jarim | 16 | 2013 | 16.2 | Ni | 0.45 | FALSE | 0.78 | FALSE | 0.31 | FALSE | NA | NA | NA | NA |
| Al-Jarim | 16 | 2013 | 2.43 | Pb | 0.06 | FALSE | 0.05 | FALSE | 0.01 | FALSE | 0.08 | FALSE | 0.02 | FALSE |
| Al-Jarim | 16 | 2013 | 11.9 | Zn | 0.1 | FALSE | 0.08 | FALSE | 0.03 | FALSE | 0.1 | FALSE | 0.04 | FALSE |
| Al-Jarim | 16 | 2012 | 0.093 | Cd | 0.3 | FALSE | 0.08 | FALSE | 0.01 | FALSE | 0.13 | FALSE | 0.02 | FALSE |
| Al-Jarim | 16 | 2012 | 66.4 | Cr | 0.82 | FALSE | 0.82 | FALSE | 0.18 | FALSE | 1.27 | TRUE | 0.42 | FALSE |
| Al-Jarim | 16 | 2012 | 6.2 | Cu | 0.23 | FALSE | 0.18 | FALSE | 0.02 | FALSE | 0.33 | FALSE | 0.06 | FALSE |
| Al-Jarim | 16 | 2012 | 25.9 | Ni | 0.72 | FALSE | 1.24 | TRUE | 0.5 | FALSE | NA | NA | NA | NA |
| Al-Jarim | 16 | 2012 | 1.13 | Pb | 0.03 | FALSE | 0.02 | FALSE | 0.01 | FALSE | 0.04 | FALSE | 0.01 | FALSE |
| Al-Jarim | 16 | 2012 | 25.9 | Zn | 0.21 | FALSE | 0.17 | FALSE | 0.06 | FALSE | 0.21 | FALSE | 0.1 | FALSE |
| Al-Jarim | 16 | 2011 | 0.076 | Cd | 0.25 | FALSE | 0.06 | FALSE | 0.01 | FALSE | 0.11 | FALSE | 0.02 | FALSE |
| Al-Jarim | 16 | 2011 | 62.8 | Cr | 0.77 | FALSE | 0.77 | FALSE | 0.17 | FALSE | 1.2 | TRUE | 0.39 | FALSE |
| Al-Jarim | 16 | 2011 | 11 | Cu | 0.41 | FALSE | 0.32 | FALSE | 0.04 | FALSE | 0.59 | FALSE | 0.1 | FALSE |
| Al-Jarim | 16 | 2011 | 28.7 | Ni | 0.8 | FALSE | 1.37 | TRUE | 0.56 | FALSE | NA | NA | NA | NA |
| Al-Jarim | 16 | 2011 | 0.17 | Pb | 0 | FALSE | 0 | FALSE | 0 | FALSE | 0.01 | FALSE | 0 | FALSE |
| Al-Jarim | 16 | 2011 | 34.9 | Zn | 0.29 | FALSE | 0.23 | FALSE | 0.09 | FALSE | 0.28 | FALSE | 0.13 | FALSE |
| Al-Jarim | 16 | 2010 | 0.212 | Cd | 0.68 | FALSE | 0.18 | FALSE | 0.02 | FALSE | 0.3 | FALSE | 0.05 | FALSE |
| Al-Jarim | 16 | 2010 | 63.1 | Cr | 0.78 | FALSE | 0.78 | FALSE | 0.17 | FALSE | 1.21 | TRUE | 0.39 | FALSE |
| Al-Jarim | 16 | 2010 | 52.5 | Cu | 1.94 | TRUE | 1.54 | TRUE | 0.19 | FALSE | 2.81 | TRUE | 0.49 | FALSE |
| Al-Jarim | 16 | 2010 | 8.8 | Ni | 0.24 | FALSE | 0.42 | FALSE | 0.17 | FALSE | NA | NA | NA | NA |
| Al-Jarim | 16 | 2010 | 2.41 | Pb | 0.06 | FALSE | 0.05 | FALSE | 0.01 | FALSE | 0.08 | FALSE | 0.02 | FALSE |
| Al-Jarim | 16 | 2010 | 17.9 | Zn | 0.15 | FALSE | 0.12 | FALSE | 0.04 | FALSE | 0.14 | FALSE | 0.07 | FALSE |
| Al-Jarim | 16 | 2009 | 0.227 | Cd | 0.73 | FALSE | 0.19 | FALSE | 0.02 | FALSE | 0.32 | FALSE | 0.05 | FALSE |
| Al-Jarim | 16 | 2009 | 50 | Cr | 0.62 | FALSE | 0.62 | FALSE | 0.14 | FALSE | 0.96 | FALSE | 0.31 | FALSE |
| Al-Jarim | 16 | 2009 | 20.9 | Cu | 0.78 | FALSE | 0.62 | FALSE | 0.08 | FALSE | 1.12 | TRUE | 0.19 | FALSE |
| Al-Jarim | 16 | 2009 | 13.1 | Ni | 0.37 | FALSE | 0.63 | FALSE | 0.25 | FALSE | NA | NA | NA | NA |
| Al-Jarim | 16 | 2009 | 0.89 | Pb | 0.02 | FALSE | 0.02 | FALSE | 0 | FALSE | 0.03 | FALSE | 0.01 | FALSE |
| Al-Jarim | 16 | 2009 | 6.9 | Zn | 0.06 | FALSE | 0.05 | FALSE | 0.02 | FALSE | 0.06 | FALSE | 0.03 | FALSE |
| Al-Jarim | 16 | 2008 | 0.023 | Cd | 0.08 | FALSE | 0.02 | FALSE | 0 | FALSE | 0.03 | FALSE | 0.01 | FALSE |
| Al-Jarim | 16 | 2008 | 34.1 | Cr | 0.42 | FALSE | 0.42 | FALSE | 0.09 | FALSE | 0.65 | FALSE | 0.21 | FALSE |
| Al-Jarim | 16 | 2008 | 9.4 | Cu | 0.35 | FALSE | 0.28 | FALSE | 0.03 | FALSE | 0.5 | FALSE | 0.09 | FALSE |
| Al-Jarim | 16 | 2008 | 1.6 | Ni | 0.04 | FALSE | 0.08 | FALSE | 0.03 | FALSE | NA | NA | NA | NA |
| Al-Jarim | 16 | 2008 | 0.87 | Pb | 0.02 | FALSE | 0.02 | FALSE | 0 | FALSE | 0.03 | FALSE | 0.01 | FALSE |
| Al-Jarim | 16 | 2008 | 18.7 | Zn | 0.15 | FALSE | 0.12 | FALSE | 0.05 | FALSE | 0.15 | FALSE | 0.07 | FALSE |
| Al-Jarim | 16 | 2007 | 33.4 | Cr | 0.41 | FALSE | 0.41 | FALSE | 0.09 | FALSE | 0.64 | FALSE | 0.21 | FALSE |
| Al-Jarim | 16 | 2007 | 2.5 | Cu | 0.09 | FALSE | 0.07 | FALSE | 0.01 | FALSE | 0.13 | FALSE | 0.02 | FALSE |
| Al-Jarim | 16 | 2007 | 0.99 | Pb | 0.03 | FALSE | 0.02 | FALSE | 0 | FALSE | 0.03 | FALSE | 0.01 | FALSE |
| Khorfasht | 17 | 2016 | 0.05 | Cd | 0.16 | FALSE | 0.04 | FALSE | 0.01 | FALSE | 0.07 | FALSE | 0.01 | FALSE |
| Khorfasht | 17 | 2016 | 282 | Cr | 3.48 | TRUE | 3.48 | TRUE | 0.76 | FALSE | 5.38 | TRUE | 1.76 | TRUE |
| Khorfasht | 17 | 2016 | 26.4 | Cu | 0.98 | FALSE | 0.78 | FALSE | 0.1 | FALSE | 1.41 | TRUE | 0.24 | FALSE |
| Khorfasht | 17 | 2016 | 1.7 | Ni | 0.05 | FALSE | 0.08 | FALSE | 0.03 | FALSE | NA | NA | NA | NA |
| Khorfasht | 17 | 2016 | 2.48 | Pb | 0.07 | FALSE | 0.05 | FALSE | 0.01 | FALSE | 0.08 | FALSE | 0.02 | FALSE |
| Khorfasht | 17 | 2016 | 11 | Zn | 0.09 | FALSE | 0.07 | FALSE | 0.03 | FALSE | 0.09 | FALSE | 0.04 | FALSE |
| Khorfasht | 17 | 2015 | 0.066 | Cd | 0.21 | FALSE | 0.05 | FALSE | 0.01 | FALSE | 0.09 | FALSE | 0.02 | FALSE |
| Khorfasht | 17 | 2015 | 173 | Cr | 2.14 | TRUE | 2.14 | TRUE | 0.47 | FALSE | 3.32 | TRUE | 1.08 | TRUE |
| Khorfasht | 17 | 2015 | 26.6 | Cu | 0.99 | FALSE | 0.78 | FALSE | 0.1 | FALSE | 1.42 | TRUE | 0.25 | FALSE |
| Khorfasht | 17 | 2015 | 7.4 | Ni | 0.21 | FALSE | 0.35 | FALSE | 0.14 | FALSE | NA | NA | NA | NA |
| Khorfasht | 17 | 2015 | 36.4 | Pb | 0.96 | FALSE | 0.78 | FALSE | 0.17 | FALSE | 1.2 | TRUE | 0.32 | FALSE |
| Khorfasht | 17 | 2015 | 10.9 | Zn | 0.09 | FALSE | 0.07 | FALSE | 0.03 | FALSE | 0.09 | FALSE | 0.04 | FALSE |
| Khorfasht | 17 | 2014 | 0.118 | Cd | 0.38 | FALSE | 0.1 | FALSE | 0.01 | FALSE | 0.17 | FALSE | 0.03 | FALSE |
| Khorfasht | 17 | 2014 | 18.3 | Cr | 0.23 | FALSE | 0.23 | FALSE | 0.05 | FALSE | 0.35 | FALSE | 0.11 | FALSE |
| Khorfasht | 17 | 2014 | 40.9 | Cu | 1.52 | TRUE | 1.2 | TRUE | 0.15 | FALSE | 2.19 | TRUE | 0.38 | FALSE |
| Khorfasht | 17 | 2014 | 21.6 | Ni | 0.6 | FALSE | 1.03 | TRUE | 0.42 | FALSE | NA | NA | NA | NA |
| Khorfasht | 17 | 2014 | 3.2 | Pb | 0.08 | FALSE | 0.07 | FALSE | 0.01 | FALSE | 0.11 | FALSE | 0.03 | FALSE |
| Khorfasht | 17 | 2014 | 13.4 | Zn | 0.11 | FALSE | 0.09 | FALSE | 0.03 | FALSE | 0.11 | FALSE | 0.05 | FALSE |
| Khorfasht | 17 | 2013 | 0.086 | Cd | 0.28 | FALSE | 0.07 | FALSE | 0.01 | FALSE | 0.12 | FALSE | 0.02 | FALSE |
| Khorfasht | 17 | 2013 | 15.1 | Cr | 0.19 | FALSE | 0.19 | FALSE | 0.04 | FALSE | 0.29 | FALSE | 0.09 | FALSE |
| Khorfasht | 17 | 2013 | 18.5 | Cu | 0.68 | FALSE | 0.54 | FALSE | 0.07 | FALSE | 0.99 | FALSE | 0.17 | FALSE |
| Khorfasht | 17 | 2013 | 17.9 | Ni | 0.5 | FALSE | 0.86 | FALSE | 0.35 | FALSE | NA | NA | NA | NA |
| Khorfasht | 17 | 2013 | 4.55 | Pb | 0.12 | FALSE | 0.1 | FALSE | 0.02 | FALSE | 0.15 | FALSE | 0.04 | FALSE |
| Khorfasht | 17 | 2013 | 19.3 | Zn | 0.16 | FALSE | 0.13 | FALSE | 0.05 | FALSE | 0.16 | FALSE | 0.07 | FALSE |
| Khorfasht | 17 | 2012 | 0.134 | Cd | 0.43 | FALSE | 0.11 | FALSE | 0.01 | FALSE | 0.19 | FALSE | 0.03 | FALSE |
| Khorfasht | 17 | 2012 | 78.7 | Cr | 0.97 | FALSE | 0.97 | FALSE | 0.21 | FALSE | 1.5 | TRUE | 0.49 | FALSE |
| Khorfasht | 17 | 2012 | 52.4 | Cu | 1.94 | TRUE | 1.54 | TRUE | 0.19 | FALSE | 2.8 | TRUE | 0.49 | FALSE |
| Khorfasht | 17 | 2012 | 27.6 | Ni | 0.77 | FALSE | 1.32 | TRUE | 0.54 | FALSE | NA | NA | NA | NA |
| Khorfasht | 17 | 2012 | 18.5 | Pb | 0.49 | FALSE | 0.4 | FALSE | 0.08 | FALSE | 0.61 | FALSE | 0.16 | FALSE |
| Khorfasht | 17 | 2012 | 27.6 | Zn | 0.23 | FALSE | 0.18 | FALSE | 0.07 | FALSE | 0.22 | FALSE | 0.1 | FALSE |
| Khorfasht | 17 | 2011 | 0.103 | Cd | 0.33 | FALSE | 0.09 | FALSE | 0.01 | FALSE | 0.15 | FALSE | 0.02 | FALSE |
| Khorfasht | 17 | 2011 | 42.1 | Cr | 0.52 | FALSE | 0.52 | FALSE | 0.11 | FALSE | 0.81 | FALSE | 0.26 | FALSE |
| Khorfasht | 17 | 2011 | 34.5 | Cu | 1.28 | TRUE | 1.02 | TRUE | 0.13 | FALSE | 1.85 | TRUE | 0.32 | FALSE |
| Khorfasht | 17 | 2011 | 29 | Ni | 0.81 | FALSE | 1.39 | TRUE | 0.56 | FALSE | NA | NA | NA | NA |
| Khorfasht | 17 | 2011 | 3.55 | Pb | 0.09 | FALSE | 0.08 | FALSE | 0.02 | FALSE | 0.12 | FALSE | 0.03 | FALSE |
| Khorfasht | 17 | 2011 | 38.4 | Zn | 0.31 | FALSE | 0.26 | FALSE | 0.09 | FALSE | 0.31 | FALSE | 0.14 | FALSE |
| Khorfasht | 17 | 2010 | 0.178 | Cd | 0.58 | FALSE | 0.15 | FALSE | 0.02 | FALSE | 0.25 | FALSE | 0.04 | FALSE |
| Khorfasht | 17 | 2010 | 87.3 | Cr | 1.08 | TRUE | 1.08 | TRUE | 0.24 | FALSE | 1.67 | TRUE | 0.55 | FALSE |
| Khorfasht | 17 | 2010 | 14.4 | Cu | 0.53 | FALSE | 0.42 | FALSE | 0.05 | FALSE | 0.77 | FALSE | 0.13 | FALSE |
| Khorfasht | 17 | 2010 | 10.6 | Ni | 0.3 | FALSE | 0.51 | FALSE | 0.21 | FALSE | NA | NA | NA | NA |
| Khorfasht | 17 | 2010 | 1.14 | Pb | 0.03 | FALSE | 0.02 | FALSE | 0.01 | FALSE | 0.04 | FALSE | 0.01 | FALSE |
| Khorfasht | 17 | 2010 | 16.5 | Zn | 0.14 | FALSE | 0.11 | FALSE | 0.04 | FALSE | 0.13 | FALSE | 0.06 | FALSE |
| Khorfasht | 17 | 2009 | 0.069 | Cd | 0.22 | FALSE | 0.06 | FALSE | 0.01 | FALSE | 0.1 | FALSE | 0.02 | FALSE |
| Khorfasht | 17 | 2009 | 168 | Cr | 2.08 | TRUE | 2.08 | TRUE | 0.46 | FALSE | 3.22 | TRUE | 1.05 | TRUE |
| Khorfasht | 17 | 2009 | 9 | Cu | 0.33 | FALSE | 0.27 | FALSE | 0.03 | FALSE | 0.48 | FALSE | 0.08 | FALSE |
| Khorfasht | 17 | 2009 | 5.7 | Ni | 0.16 | FALSE | 0.27 | FALSE | 0.11 | FALSE | NA | NA | NA | NA |
| Khorfasht | 17 | 2009 | 0.69 | Pb | 0.02 | FALSE | 0.01 | FALSE | 0 | FALSE | 0.02 | FALSE | 0.01 | FALSE |
| Khorfasht | 17 | 2009 | 7.5 | Zn | 0.06 | FALSE | 0.05 | FALSE | 0.02 | FALSE | 0.06 | FALSE | 0.03 | FALSE |
| Khorfasht | 17 | 2008 | 0.063 | Cd | 0.2 | FALSE | 0.05 | FALSE | 0.01 | FALSE | 0.09 | FALSE | 0.02 | FALSE |
| Khorfasht | 17 | 2008 | 499 | Cr | 6.17 | TRUE | 6.17 | TRUE | 1.35 | TRUE | 9.55 | TRUE | 3.12 | TRUE |
| Khorfasht | 17 | 2008 | 3.5 | Cu | 0.13 | FALSE | 0.1 | FALSE | 0.01 | FALSE | 0.19 | FALSE | 0.03 | FALSE |
| Khorfasht | 17 | 2008 | 2.46 | Pb | 0.06 | FALSE | 0.05 | FALSE | 0.01 | FALSE | 0.08 | FALSE | 0.02 | FALSE |
| Khorfasht | 17 | 2007 | 75.8 | Cr | 0.94 | FALSE | 0.94 | FALSE | 0.2 | FALSE | 1.45 | TRUE | 0.47 | FALSE |
| Khorfasht | 17 | 2007 | 5.7 | Cu | 0.21 | FALSE | 0.17 | FALSE | 0.02 | FALSE | 0.31 | FALSE | 0.05 | FALSE |
| Khorfasht | 17 | 2007 | 11 | Ni | 0.31 | FALSE | 0.53 | FALSE | 0.21 | FALSE | NA | NA | NA | NA |
| Khorfasht | 17 | 2007 | 1.49 | Pb | 0.04 | FALSE | 0.03 | FALSE | 0.01 | FALSE | 0.05 | FALSE | 0.01 | FALSE |
| Murwada | 18 | 2020 | 0.027 | Cd | 0.09 | FALSE | 0.02 | FALSE | 0 | FALSE | 0.04 | FALSE | 0.01 | FALSE |
| Murwada | 18 | 2020 | 23 | Cr | 0.28 | FALSE | 0.28 | FALSE | 0.06 | FALSE | 0.44 | FALSE | 0.14 | FALSE |
| Murwada | 18 | 2020 | 103 | Cu | 3.83 | TRUE | 3.04 | TRUE | 0.38 | FALSE | 5.53 | TRUE | 0.96 | FALSE |
| Murwada | 18 | 2020 | 9.5 | Ni | 0.26 | FALSE | 0.45 | FALSE | 0.18 | FALSE | NA | NA | NA | NA |
| Murwada | 18 | 2020 | 2.14 | Pb | 0.06 | FALSE | 0.05 | FALSE | 0.01 | FALSE | 0.07 | FALSE | 0.02 | FALSE |
| Murwada | 18 | 2020 | 23.9 | Zn | 0.2 | FALSE | 0.16 | FALSE | 0.06 | FALSE | 0.19 | FALSE | 0.09 | FALSE |
| Murwada | 18 | 2019 | 0.066 | Cd | 0.21 | FALSE | 0.06 | FALSE | 0.01 | FALSE | 0.09 | FALSE | 0.02 | FALSE |
| Murwada | 18 | 2019 | 17.9 | Cr | 0.22 | FALSE | 0.22 | FALSE | 0.05 | FALSE | 0.34 | FALSE | 0.11 | FALSE |
| Murwada | 18 | 2019 | 76.3 | Cu | 2.83 | TRUE | 2.24 | TRUE | 0.28 | FALSE | 4.08 | TRUE | 0.71 | FALSE |
| Murwada | 18 | 2019 | 9.7 | Ni | 0.27 | FALSE | 0.46 | FALSE | 0.19 | FALSE | NA | NA | NA | NA |
| Murwada | 18 | 2019 | 10.7 | Pb | 0.28 | FALSE | 0.23 | FALSE | 0.05 | FALSE | 0.35 | FALSE | 0.1 | FALSE |
| Murwada | 18 | 2019 | 45.5 | Zn | 0.37 | FALSE | 0.3 | FALSE | 0.11 | FALSE | 0.37 | FALSE | 0.17 | FALSE |
| Murwada | 18 | 2018 | 0.089 | Cd | 0.29 | FALSE | 0.07 | FALSE | 0.01 | FALSE | 0.13 | FALSE | 0.02 | FALSE |
| Murwada | 18 | 2018 | 111 | Cr | 1.37 | TRUE | 1.37 | TRUE | 0.3 | FALSE | 2.11 | TRUE | 0.69 | FALSE |
| Murwada | 18 | 2018 | 53.9 | Cu | 2 | TRUE | 1.58 | TRUE | 0.2 | FALSE | 2.88 | TRUE | 0.5 | FALSE |
| Murwada | 18 | 2018 | 21.9 | Ni | 0.61 | FALSE | 1.05 | TRUE | 0.42 | FALSE | NA | NA | NA | NA |
| Murwada | 18 | 2018 | 3.35 | Pb | 0.09 | FALSE | 0.07 | FALSE | 0.02 | FALSE | 0.11 | FALSE | 0.03 | FALSE |
| Murwada | 18 | 2018 | 32.2 | Zn | 0.26 | FALSE | 0.21 | FALSE | 0.08 | FALSE | 0.26 | FALSE | 0.12 | FALSE |
| Murwada | 18 | 2017 | 0.094 | Cd | 0.3 | FALSE | 0.08 | FALSE | 0.01 | FALSE | 0.13 | FALSE | 0.02 | FALSE |
| Murwada | 18 | 2017 | 48.2 | Cr | 0.59 | FALSE | 0.59 | FALSE | 0.13 | FALSE | 0.92 | FALSE | 0.3 | FALSE |
| Murwada | 18 | 2017 | 152 | Cu | 5.64 | TRUE | 4.48 | TRUE | 0.56 | FALSE | 8.15 | TRUE | 1.41 | TRUE |
| Murwada | 18 | 2017 | 16.1 | Ni | 0.45 | FALSE | 0.77 | FALSE | 0.31 | FALSE | NA | NA | NA | NA |
| Murwada | 18 | 2017 | 4.17 | Pb | 0.11 | FALSE | 0.09 | FALSE | 0.02 | FALSE | 0.14 | FALSE | 0.04 | FALSE |
| Murwada | 18 | 2017 | 18.2 | Zn | 0.15 | FALSE | 0.12 | FALSE | 0.04 | FALSE | 0.15 | FALSE | 0.07 | FALSE |
| Murwada | 18 | 2016 | 0.038 | Cd | 0.12 | FALSE | 0.03 | FALSE | 0 | FALSE | 0.05 | FALSE | 0.01 | FALSE |
| Murwada | 18 | 2016 | 169 | Cr | 2.08 | TRUE | 2.08 | TRUE | 0.46 | FALSE | 3.23 | TRUE | 1.05 | TRUE |
| Murwada | 18 | 2016 | 18.5 | Cu | 0.69 | FALSE | 0.55 | FALSE | 0.07 | FALSE | 0.99 | FALSE | 0.17 | FALSE |
| Murwada | 18 | 2016 | 2.5 | Ni | 0.07 | FALSE | 0.12 | FALSE | 0.05 | FALSE | NA | NA | NA | NA |
| Murwada | 18 | 2016 | 3.39 | Pb | 0.09 | FALSE | 0.07 | FALSE | 0.02 | FALSE | 0.11 | FALSE | 0.03 | FALSE |
| Murwada | 18 | 2016 | 17.3 | Zn | 0.14 | FALSE | 0.12 | FALSE | 0.04 | FALSE | 0.14 | FALSE | 0.06 | FALSE |
| Murwada | 18 | 2015 | 0.05 | Cd | 0.16 | FALSE | 0.04 | FALSE | 0.01 | FALSE | 0.07 | FALSE | 0.01 | FALSE |
| Murwada | 18 | 2015 | 82.4 | Cr | 1.02 | TRUE | 1.02 | TRUE | 0.22 | FALSE | 1.58 | TRUE | 0.52 | FALSE |
| Murwada | 18 | 2015 | 19.1 | Cu | 0.71 | FALSE | 0.56 | FALSE | 0.07 | FALSE | 1.02 | TRUE | 0.18 | FALSE |
| Murwada | 18 | 2015 | 5.8 | Ni | 0.16 | FALSE | 0.28 | FALSE | 0.11 | FALSE | NA | NA | NA | NA |
| Murwada | 18 | 2015 | 34.6 | Pb | 0.91 | FALSE | 0.74 | FALSE | 0.16 | FALSE | 1.14 | TRUE | 0.31 | FALSE |
| Murwada | 18 | 2015 | 9.9 | Zn | 0.08 | FALSE | 0.07 | FALSE | 0.02 | FALSE | 0.08 | FALSE | 0.04 | FALSE |
| Murwada | 18 | 2014 | 0.119 | Cd | 0.38 | FALSE | 0.1 | FALSE | 0.01 | FALSE | 0.17 | FALSE | 0.03 | FALSE |
| Murwada | 18 | 2014 | 27.3 | Cr | 0.34 | FALSE | 0.34 | FALSE | 0.07 | FALSE | 0.52 | FALSE | 0.17 | FALSE |
| Murwada | 18 | 2014 | 35.1 | Cu | 1.3 | TRUE | 1.03 | TRUE | 0.13 | FALSE | 1.88 | TRUE | 0.33 | FALSE |
| Murwada | 18 | 2014 | 26.8 | Ni | 0.74 | FALSE | 1.28 | TRUE | 0.52 | FALSE | NA | NA | NA | NA |
| Murwada | 18 | 2014 | 2.98 | Pb | 0.08 | FALSE | 0.06 | FALSE | 0.01 | FALSE | 0.1 | FALSE | 0.03 | FALSE |
| Murwada | 18 | 2014 | 19.5 | Zn | 0.16 | FALSE | 0.13 | FALSE | 0.05 | FALSE | 0.16 | FALSE | 0.07 | FALSE |
| Murwada | 18 | 2013 | 0.095 | Cd | 0.31 | FALSE | 0.08 | FALSE | 0.01 | FALSE | 0.14 | FALSE | 0.02 | FALSE |
| Murwada | 18 | 2013 | 10.4 | Cr | 0.13 | FALSE | 0.13 | FALSE | 0.03 | FALSE | 0.2 | FALSE | 0.06 | FALSE |
| Murwada | 18 | 2013 | 94.8 | Cu | 3.51 | TRUE | 2.79 | TRUE | 0.35 | FALSE | 5.07 | TRUE | 0.88 | FALSE |
| Murwada | 18 | 2013 | 5 | Ni | 0.14 | FALSE | 0.24 | FALSE | 0.1 | FALSE | NA | NA | NA | NA |
| Murwada | 18 | 2013 | 5.24 | Pb | 0.14 | FALSE | 0.11 | FALSE | 0.02 | FALSE | 0.17 | FALSE | 0.05 | FALSE |
| Murwada | 18 | 2013 | 24.3 | Zn | 0.2 | FALSE | 0.16 | FALSE | 0.06 | FALSE | 0.2 | FALSE | 0.09 | FALSE |
| Murwada | 18 | 2012 | 0.114 | Cd | 0.37 | FALSE | 0.09 | FALSE | 0.01 | FALSE | 0.16 | FALSE | 0.03 | FALSE |
| Murwada | 18 | 2012 | 27.6 | Cr | 0.34 | FALSE | 0.34 | FALSE | 0.07 | FALSE | 0.53 | FALSE | 0.17 | FALSE |
| Murwada | 18 | 2012 | 41.7 | Cu | 1.54 | TRUE | 1.23 | TRUE | 0.15 | FALSE | 2.23 | TRUE | 0.39 | FALSE |
| Murwada | 18 | 2012 | 34.5 | Ni | 0.96 | FALSE | 1.65 | TRUE | 0.67 | FALSE | NA | NA | NA | NA |
| Murwada | 18 | 2012 | 24.2 | Pb | 0.64 | FALSE | 0.52 | FALSE | 0.11 | FALSE | 0.8 | FALSE | 0.22 | FALSE |
| Murwada | 18 | 2012 | 34.5 | Zn | 0.28 | FALSE | 0.23 | FALSE | 0.08 | FALSE | 0.28 | FALSE | 0.13 | FALSE |
| Murwada | 18 | 2011 | 0.046 | Cd | 0.15 | FALSE | 0.04 | FALSE | 0 | FALSE | 0.07 | FALSE | 0.01 | FALSE |
| Murwada | 18 | 2011 | 28.9 | Cr | 0.36 | FALSE | 0.36 | FALSE | 0.08 | FALSE | 0.55 | FALSE | 0.18 | FALSE |
| Murwada | 18 | 2011 | 25.6 | Cu | 0.95 | FALSE | 0.75 | FALSE | 0.09 | FALSE | 1.37 | TRUE | 0.24 | FALSE |
| Murwada | 18 | 2011 | 18 | Ni | 0.5 | FALSE | 0.86 | FALSE | 0.35 | FALSE | NA | NA | NA | NA |
| Murwada | 18 | 2011 | 3.61 | Pb | 0.09 | FALSE | 0.08 | FALSE | 0.02 | FALSE | 0.12 | FALSE | 0.03 | FALSE |
| Murwada | 18 | 2011 | 33.8 | Zn | 0.28 | FALSE | 0.23 | FALSE | 0.08 | FALSE | 0.27 | FALSE | 0.12 | FALSE |
| Murwada | 18 | 2010 | 0.079 | Cd | 0.26 | FALSE | 0.07 | FALSE | 0.01 | FALSE | 0.11 | FALSE | 0.02 | FALSE |
| Murwada | 18 | 2010 | 28.9 | Cr | 0.36 | FALSE | 0.36 | FALSE | 0.08 | FALSE | 0.55 | FALSE | 0.18 | FALSE |
| Murwada | 18 | 2010 | 3.3 | Cu | 0.12 | FALSE | 0.1 | FALSE | 0.01 | FALSE | 0.18 | FALSE | 0.03 | FALSE |
| Murwada | 18 | 2010 | 1.5 | Ni | 0.04 | FALSE | 0.07 | FALSE | 0.03 | FALSE | NA | NA | NA | NA |
| Murwada | 18 | 2010 | 0.37 | Pb | 0.01 | FALSE | 0.01 | FALSE | 0 | FALSE | 0.01 | FALSE | 0 | FALSE |
| Murwada | 18 | 2010 | 9.9 | Zn | 0.08 | FALSE | 0.07 | FALSE | 0.02 | FALSE | 0.08 | FALSE | 0.04 | FALSE |
| Murwada | 18 | 2009 | 0.156 | Cd | 0.5 | FALSE | 0.13 | FALSE | 0.02 | FALSE | 0.22 | FALSE | 0.04 | FALSE |
| Murwada | 18 | 2009 | 66.2 | Cr | 0.82 | FALSE | 0.82 | FALSE | 0.18 | FALSE | 1.27 | TRUE | 0.41 | FALSE |
| Murwada | 18 | 2009 | 21.8 | Cu | 0.81 | FALSE | 0.64 | FALSE | 0.08 | FALSE | 1.16 | TRUE | 0.2 | FALSE |
| Murwada | 18 | 2009 | 16.6 | Ni | 0.46 | FALSE | 0.79 | FALSE | 0.32 | FALSE | NA | NA | NA | NA |
| Murwada | 18 | 2009 | 1.14 | Pb | 0.03 | FALSE | 0.02 | FALSE | 0.01 | FALSE | 0.04 | FALSE | 0.01 | FALSE |
| Murwada | 18 | 2009 | 8.5 | Zn | 0.07 | FALSE | 0.06 | FALSE | 0.02 | FALSE | 0.07 | FALSE | 0.03 | FALSE |
| Murwada | 18 | 2008 | 0.016 | Cd | 0.05 | FALSE | 0.01 | FALSE | 0 | FALSE | 0.02 | FALSE | 0 | FALSE |
| Murwada | 18 | 2008 | 0.9 | Cu | 0.03 | FALSE | 0.03 | FALSE | 0 | FALSE | 0.05 | FALSE | 0.01 | FALSE |
| Murwada | 18 | 2008 | 0.25 | Pb | 0.01 | FALSE | 0.01 | FALSE | 0 | FALSE | 0.01 | FALSE | 0 | FALSE |
| Murwada | 18 | 2008 | 1.5 | Zn | 0.01 | FALSE | 0.01 | FALSE | 0 | FALSE | 0.01 | FALSE | 0.01 | FALSE |
| Murwada | 18 | 2007 | 9.2 | Cr | 0.11 | FALSE | 0.11 | FALSE | 0.02 | FALSE | 0.18 | FALSE | 0.06 | FALSE |
| Murwada | 18 | 2007 | 15.5 | Cu | 0.58 | FALSE | 0.46 | FALSE | 0.06 | FALSE | 0.83 | FALSE | 0.14 | FALSE |
| Murwada | 18 | 2007 | 0.78 | Pb | 0.02 | FALSE | 0.02 | FALSE | 0 | FALSE | 0.03 | FALSE | 0.01 | FALSE |
| Bartafi | 19 | 2016 | 0.07 | Cd | 0.22 | FALSE | 0.06 | FALSE | 0.01 | FALSE | 0.1 | FALSE | 0.02 | FALSE |
| Bartafi | 19 | 2016 | 34.2 | Cr | 0.42 | FALSE | 0.42 | FALSE | 0.09 | FALSE | 0.65 | FALSE | 0.21 | FALSE |
| Bartafi | 19 | 2016 | 17.2 | Cu | 0.64 | FALSE | 0.51 | FALSE | 0.06 | FALSE | 0.92 | FALSE | 0.16 | FALSE |
| Bartafi | 19 | 2016 | 8.1 | Ni | 0.22 | FALSE | 0.39 | FALSE | 0.16 | FALSE | NA | NA | NA | NA |
| Bartafi | 19 | 2016 | 0.96 | Pb | 0.03 | FALSE | 0.02 | FALSE | 0 | FALSE | 0.03 | FALSE | 0.01 | FALSE |
| Bartafi | 19 | 2016 | 16 | Zn | 0.13 | FALSE | 0.11 | FALSE | 0.04 | FALSE | 0.13 | FALSE | 0.06 | FALSE |
| Bartafi | 19 | 2015 | 0.048 | Cd | 0.16 | FALSE | 0.04 | FALSE | 0.01 | FALSE | 0.07 | FALSE | 0.01 | FALSE |
| Bartafi | 19 | 2015 | 255 | Cr | 3.14 | TRUE | 3.14 | TRUE | 0.69 | FALSE | 4.87 | TRUE | 1.59 | TRUE |
| Bartafi | 19 | 2015 | 17.3 | Cu | 0.64 | FALSE | 0.51 | FALSE | 0.06 | FALSE | 0.93 | FALSE | 0.16 | FALSE |
| Bartafi | 19 | 2015 | 9.8 | Ni | 0.27 | FALSE | 0.47 | FALSE | 0.19 | FALSE | NA | NA | NA | NA |
| Bartafi | 19 | 2015 | 19.1 | Pb | 0.5 | FALSE | 0.41 | FALSE | 0.09 | FALSE | 0.63 | FALSE | 0.17 | FALSE |
| Bartafi | 19 | 2015 | 4.8 | Zn | 0.04 | FALSE | 0.03 | FALSE | 0.01 | FALSE | 0.04 | FALSE | 0.02 | FALSE |
| Bartafi | 19 | 2014 | 0.104 | Cd | 0.33 | FALSE | 0.09 | FALSE | 0.01 | FALSE | 0.15 | FALSE | 0.02 | FALSE |
| Bartafi | 19 | 2014 | 499 | Cr | 6.16 | TRUE | 6.16 | TRUE | 1.35 | TRUE | 9.54 | TRUE | 3.12 | TRUE |
| Bartafi | 19 | 2014 | 38.9 | Cu | 1.44 | TRUE | 1.14 | TRUE | 0.14 | FALSE | 2.08 | TRUE | 0.36 | FALSE |
| Bartafi | 19 | 2014 | 14.7 | Ni | 0.41 | FALSE | 0.7 | FALSE | 0.28 | FALSE | NA | NA | NA | NA |
| Bartafi | 19 | 2014 | 2.81 | Pb | 0.07 | FALSE | 0.06 | FALSE | 0.01 | FALSE | 0.09 | FALSE | 0.03 | FALSE |
| Bartafi | 19 | 2014 | 20.5 | Zn | 0.17 | FALSE | 0.14 | FALSE | 0.05 | FALSE | 0.16 | FALSE | 0.08 | FALSE |
| Bartafi | 19 | 2013 | 0.088 | Cd | 0.29 | FALSE | 0.07 | FALSE | 0.01 | FALSE | 0.13 | FALSE | 0.02 | FALSE |
| Bartafi | 19 | 2013 | 249 | Cr | 3.08 | TRUE | 3.08 | TRUE | 0.67 | FALSE | 4.77 | TRUE | 1.56 | TRUE |
| Bartafi | 19 | 2013 | 22.8 | Cu | 0.85 | FALSE | 0.67 | FALSE | 0.08 | FALSE | 1.22 | TRUE | 0.21 | FALSE |
| Bartafi | 19 | 2013 | 17.9 | Ni | 0.5 | FALSE | 0.86 | FALSE | 0.35 | FALSE | NA | NA | NA | NA |
| Bartafi | 19 | 2013 | 8.96 | Pb | 0.24 | FALSE | 0.19 | FALSE | 0.04 | FALSE | 0.3 | FALSE | 0.08 | FALSE |
| Bartafi | 19 | 2013 | 12.4 | Zn | 0.1 | FALSE | 0.08 | FALSE | 0.03 | FALSE | 0.1 | FALSE | 0.05 | FALSE |
| Bartafi | 19 | 2012 | 0.072 | Cd | 0.23 | FALSE | 0.06 | FALSE | 0.01 | FALSE | 0.1 | FALSE | 0.02 | FALSE |
| Bartafi | 19 | 2012 | 40.5 | Cr | 0.5 | FALSE | 0.5 | FALSE | 0.11 | FALSE | 0.77 | FALSE | 0.25 | FALSE |
| Bartafi | 19 | 2012 | 27.1 | Cu | 1 | TRUE | 0.8 | FALSE | 0.1 | FALSE | 1.45 | TRUE | 0.25 | FALSE |
| Bartafi | 19 | 2012 | 20 | Ni | 0.56 | FALSE | 0.96 | FALSE | 0.39 | FALSE | NA | NA | NA | NA |
| Bartafi | 19 | 2012 | 19.6 | Pb | 0.51 | FALSE | 0.42 | FALSE | 0.09 | FALSE | 0.65 | FALSE | 0.17 | FALSE |
| Bartafi | 19 | 2012 | 20 | Zn | 0.16 | FALSE | 0.13 | FALSE | 0.05 | FALSE | 0.16 | FALSE | 0.07 | FALSE |
| Bartafi | 19 | 2011 | 0.037 | Cd | 0.12 | FALSE | 0.03 | FALSE | 0 | FALSE | 0.05 | FALSE | 0.01 | FALSE |
| Bartafi | 19 | 2011 | 201 | Cr | 2.49 | TRUE | 2.49 | TRUE | 0.54 | FALSE | 3.85 | TRUE | 1.26 | TRUE |
| Bartafi | 19 | 2011 | 17 | Cu | 0.63 | FALSE | 0.5 | FALSE | 0.06 | FALSE | 0.91 | FALSE | 0.16 | FALSE |
| Bartafi | 19 | 2011 | 15.7 | Ni | 0.44 | FALSE | 0.75 | FALSE | 0.3 | FALSE | NA | NA | NA | NA |
| Bartafi | 19 | 2011 | 0.28 | Pb | 0.01 | FALSE | 0.01 | FALSE | 0 | FALSE | 0.01 | FALSE | 0 | FALSE |
| Bartafi | 19 | 2011 | 28.2 | Zn | 0.23 | FALSE | 0.19 | FALSE | 0.07 | FALSE | 0.23 | FALSE | 0.1 | FALSE |
| Bartafi | 19 | 2010 | 0.138 | Cd | 0.44 | FALSE | 0.11 | FALSE | 0.01 | FALSE | 0.2 | FALSE | 0.03 | FALSE |
| Bartafi | 19 | 2010 | 168 | Cr | 2.08 | TRUE | 2.08 | TRUE | 0.46 | FALSE | 3.22 | TRUE | 1.05 | TRUE |
| Bartafi | 19 | 2010 | 15.6 | Cu | 0.58 | FALSE | 0.46 | FALSE | 0.06 | FALSE | 0.83 | FALSE | 0.14 | FALSE |
| Bartafi | 19 | 2010 | 6.6 | Ni | 0.18 | FALSE | 0.32 | FALSE | 0.13 | FALSE | NA | NA | NA | NA |
| Bartafi | 19 | 2010 | 3.99 | Pb | 0.1 | FALSE | 0.09 | FALSE | 0.02 | FALSE | 0.13 | FALSE | 0.04 | FALSE |
| Bartafi | 19 | 2010 | 22.6 | Zn | 0.19 | FALSE | 0.15 | FALSE | 0.06 | FALSE | 0.18 | FALSE | 0.08 | FALSE |
| Bartafi | 19 | 2009 | 0.122 | Cd | 0.39 | FALSE | 0.1 | FALSE | 0.01 | FALSE | 0.17 | FALSE | 0.03 | FALSE |
| Bartafi | 19 | 2009 | 69.2 | Cr | 0.85 | FALSE | 0.85 | FALSE | 0.19 | FALSE | 1.32 | TRUE | 0.43 | FALSE |
| Bartafi | 19 | 2009 | 14.9 | Cu | 0.55 | FALSE | 0.44 | FALSE | 0.06 | FALSE | 0.8 | FALSE | 0.14 | FALSE |
| Bartafi | 19 | 2009 | 5.6 | Ni | 0.16 | FALSE | 0.27 | FALSE | 0.11 | FALSE | NA | NA | NA | NA |
| Bartafi | 19 | 2009 | 1.09 | Pb | 0.03 | FALSE | 0.02 | FALSE | 0 | FALSE | 0.04 | FALSE | 0.01 | FALSE |
| Bartafi | 19 | 2009 | 5.8 | Zn | 0.05 | FALSE | 0.04 | FALSE | 0.01 | FALSE | 0.05 | FALSE | 0.02 | FALSE |
| Bartafi | 19 | 2008 | 0.006 | Cd | 0.02 | FALSE | 0 | FALSE | 0 | FALSE | 0.01 | FALSE | 0 | FALSE |
| Bartafi | 19 | 2008 | 8.3 | Cr | 0.1 | FALSE | 0.1 | FALSE | 0.02 | FALSE | 0.16 | FALSE | 0.05 | FALSE |
| Bartafi | 19 | 2008 | 0.7 | Cu | 0.03 | FALSE | 0.02 | FALSE | 0 | FALSE | 0.04 | FALSE | 0.01 | FALSE |
| Bartafi | 19 | 2008 | 1.1 | Pb | 0.03 | FALSE | 0.02 | FALSE | 0.01 | FALSE | 0.04 | FALSE | 0.01 | FALSE |
| Bartafi | 19 | 2007 | 82.4 | Cr | 1.02 | TRUE | 1.02 | TRUE | 0.22 | FALSE | 1.57 | TRUE | 0.51 | FALSE |
| Bartafi | 19 | 2007 | 9.5 | Cu | 0.35 | FALSE | 0.28 | FALSE | 0.04 | FALSE | 0.51 | FALSE | 0.09 | FALSE |
| Bartafi | 19 | 2007 | 13.1 | Ni | 0.36 | FALSE | 0.63 | FALSE | 0.25 | FALSE | NA | NA | NA | NA |
| Bartafi | 19 | 2007 | 5.09 | Pb | 0.13 | FALSE | 0.11 | FALSE | 0.02 | FALSE | 0.17 | FALSE | 0.05 | FALSE |
| Bartafi | 19 | 2007 | 4.8 | Zn | 0.04 | FALSE | 0.03 | FALSE | 0.01 | FALSE | 0.04 | FALSE | 0.02 | FALSE |
| Gasar | 20 | 2016 | 0.128 | Cd | 0.41 | FALSE | 0.11 | FALSE | 0.01 | FALSE | 0.18 | FALSE | 0.03 | FALSE |
| Gasar | 20 | 2016 | 51.9 | Cr | 0.64 | FALSE | 0.64 | FALSE | 0.14 | FALSE | 0.99 | FALSE | 0.32 | FALSE |
| Gasar | 20 | 2016 | 16.2 | Cu | 0.6 | FALSE | 0.48 | FALSE | 0.06 | FALSE | 0.86 | FALSE | 0.15 | FALSE |
| Gasar | 20 | 2016 | 36 | Ni | 1 | TRUE | 1.72 | TRUE | 0.7 | FALSE | NA | NA | NA | NA |
| Gasar | 20 | 2016 | 1.55 | Pb | 0.04 | FALSE | 0.03 | FALSE | 0.01 | FALSE | 0.05 | FALSE | 0.01 | FALSE |
| Gasar | 20 | 2016 | 19.4 | Zn | 0.16 | FALSE | 0.13 | FALSE | 0.05 | FALSE | 0.16 | FALSE | 0.07 | FALSE |
| Gasar | 20 | 2015 | 0.085 | Cd | 0.27 | FALSE | 0.07 | FALSE | 0.01 | FALSE | 0.12 | FALSE | 0.02 | FALSE |
| Gasar | 20 | 2015 | 45.7 | Cr | 0.56 | FALSE | 0.56 | FALSE | 0.12 | FALSE | 0.87 | FALSE | 0.29 | FALSE |
| Gasar | 20 | 2015 | 16.7 | Cu | 0.62 | FALSE | 0.49 | FALSE | 0.06 | FALSE | 0.9 | FALSE | 0.16 | FALSE |
| Gasar | 20 | 2015 | 35.9 | Ni | 1 | FALSE | 1.72 | TRUE | 0.7 | FALSE | NA | NA | NA | NA |
| Gasar | 20 | 2015 | 4.84 | Pb | 0.13 | FALSE | 0.1 | FALSE | 0.02 | FALSE | 0.16 | FALSE | 0.04 | FALSE |
| Gasar | 20 | 2015 | 17 | Zn | 0.14 | FALSE | 0.11 | FALSE | 0.04 | FALSE | 0.14 | FALSE | 0.06 | FALSE |
| Gasar | 20 | 2014 | 0.112 | Cd | 0.36 | FALSE | 0.09 | FALSE | 0.01 | FALSE | 0.16 | FALSE | 0.03 | FALSE |
| Gasar | 20 | 2014 | 55 | Cr | 0.68 | FALSE | 0.68 | FALSE | 0.15 | FALSE | 1.05 | TRUE | 0.34 | FALSE |
| Gasar | 20 | 2014 | 20 | Cu | 0.74 | FALSE | 0.59 | FALSE | 0.07 | FALSE | 1.07 | TRUE | 0.19 | FALSE |
| Gasar | 20 | 2014 | 51.7 | Ni | 1.44 | TRUE | 2.47 | TRUE | 1 | TRUE | NA | NA | NA | NA |
| Gasar | 20 | 2014 | 1.55 | Pb | 0.04 | FALSE | 0.03 | FALSE | 0.01 | FALSE | 0.05 | FALSE | 0.01 | FALSE |
| Gasar | 20 | 2014 | 25.9 | Zn | 0.21 | FALSE | 0.17 | FALSE | 0.06 | FALSE | 0.21 | FALSE | 0.1 | FALSE |
| Gasar | 20 | 2013 | 0.097 | Cd | 0.31 | FALSE | 0.08 | FALSE | 0.01 | FALSE | 0.14 | FALSE | 0.02 | FALSE |
| Gasar | 20 | 2013 | 49.6 | Cr | 0.61 | FALSE | 0.61 | FALSE | 0.13 | FALSE | 0.95 | FALSE | 0.31 | FALSE |
| Gasar | 20 | 2013 | 16.1 | Cu | 0.6 | FALSE | 0.47 | FALSE | 0.06 | FALSE | 0.86 | FALSE | 0.15 | FALSE |
| Gasar | 20 | 2013 | 37 | Ni | 1.03 | TRUE | 1.77 | TRUE | 0.72 | FALSE | NA | NA | NA | NA |
| Gasar | 20 | 2013 | 4.32 | Pb | 0.11 | FALSE | 0.09 | FALSE | 0.02 | FALSE | 0.14 | FALSE | 0.04 | FALSE |
| Gasar | 20 | 2013 | 18.5 | Zn | 0.15 | FALSE | 0.12 | FALSE | 0.05 | FALSE | 0.15 | FALSE | 0.07 | FALSE |
| Gasar | 20 | 2012 | 0.065 | Cd | 0.21 | FALSE | 0.05 | FALSE | 0.01 | FALSE | 0.09 | FALSE | 0.02 | FALSE |
| Gasar | 20 | 2012 | 59 | Cr | 0.73 | FALSE | 0.73 | FALSE | 0.16 | FALSE | 1.13 | TRUE | 0.37 | FALSE |
| Gasar | 20 | 2012 | 11.7 | Cu | 0.43 | FALSE | 0.35 | FALSE | 0.04 | FALSE | 0.63 | FALSE | 0.11 | FALSE |
| Gasar | 20 | 2012 | 26.2 | Ni | 0.73 | FALSE | 1.25 | TRUE | 0.51 | FALSE | NA | NA | NA | NA |
| Gasar | 20 | 2012 | 1.86 | Pb | 0.05 | FALSE | 0.04 | FALSE | 0.01 | FALSE | 0.06 | FALSE | 0.02 | FALSE |
| Gasar | 20 | 2012 | 26.2 | Zn | 0.21 | FALSE | 0.17 | FALSE | 0.06 | FALSE | 0.21 | FALSE | 0.1 | FALSE |
| Gasar | 20 | 2011 | 0.064 | Cd | 0.21 | FALSE | 0.05 | FALSE | 0.01 | FALSE | 0.09 | FALSE | 0.02 | FALSE |
| Gasar | 20 | 2011 | 63.9 | Cr | 0.79 | FALSE | 0.79 | FALSE | 0.17 | FALSE | 1.22 | TRUE | 0.4 | FALSE |
| Gasar | 20 | 2011 | 21.7 | Cu | 0.8 | FALSE | 0.64 | FALSE | 0.08 | FALSE | 1.16 | TRUE | 0.2 | FALSE |
| Gasar | 20 | 2011 | 42.7 | Ni | 1.19 | TRUE | 2.04 | TRUE | 0.83 | FALSE | NA | NA | NA | NA |
| Gasar | 20 | 2011 | 0.72 | Pb | 0.02 | FALSE | 0.02 | FALSE | 0 | FALSE | 0.02 | FALSE | 0.01 | FALSE |
| Gasar | 20 | 2011 | 39.4 | Zn | 0.32 | FALSE | 0.26 | FALSE | 0.1 | FALSE | 0.32 | FALSE | 0.15 | FALSE |
| Gasar | 20 | 2010 | 0.18 | Cd | 0.58 | FALSE | 0.15 | FALSE | 0.02 | FALSE | 0.26 | FALSE | 0.04 | FALSE |
| Gasar | 20 | 2010 | 78.2 | Cr | 0.97 | FALSE | 0.97 | FALSE | 0.21 | FALSE | 1.5 | TRUE | 0.49 | FALSE |
| Gasar | 20 | 2010 | 18.1 | Cu | 0.67 | FALSE | 0.53 | FALSE | 0.07 | FALSE | 0.97 | FALSE | 0.17 | FALSE |
| Gasar | 20 | 2010 | 29.2 | Ni | 0.81 | FALSE | 1.39 | TRUE | 0.57 | FALSE | NA | NA | NA | NA |
| Gasar | 20 | 2010 | 1.11 | Pb | 0.03 | FALSE | 0.02 | FALSE | 0.01 | FALSE | 0.04 | FALSE | 0.01 | FALSE |
| Gasar | 20 | 2010 | 19.6 | Zn | 0.16 | FALSE | 0.13 | FALSE | 0.05 | FALSE | 0.16 | FALSE | 0.07 | FALSE |
| Gasar | 20 | 2009 | 0.166 | Cd | 0.54 | FALSE | 0.14 | FALSE | 0.02 | FALSE | 0.24 | FALSE | 0.04 | FALSE |
| Gasar | 20 | 2009 | 66.1 | Cr | 0.82 | FALSE | 0.82 | FALSE | 0.18 | FALSE | 1.26 | TRUE | 0.41 | FALSE |
| Gasar | 20 | 2009 | 14.4 | Cu | 0.53 | FALSE | 0.42 | FALSE | 0.05 | FALSE | 0.77 | FALSE | 0.13 | FALSE |
| Gasar | 20 | 2009 | 28.1 | Ni | 0.78 | FALSE | 1.34 | TRUE | 0.54 | FALSE | NA | NA | NA | NA |
| Gasar | 20 | 2009 | 2.2 | Pb | 0.06 | FALSE | 0.05 | FALSE | 0.01 | FALSE | 0.07 | FALSE | 0.02 | FALSE |
| Gasar | 20 | 2009 | 15.4 | Zn | 0.13 | FALSE | 0.1 | FALSE | 0.04 | FALSE | 0.12 | FALSE | 0.06 | FALSE |
| Gasar | 20 | 2008 | 0.2 | Cd | 0.65 | FALSE | 0.17 | FALSE | 0.02 | FALSE | 0.29 | FALSE | 0.05 | FALSE |
| Gasar | 20 | 2008 | 32.7 | Cr | 0.4 | FALSE | 0.4 | FALSE | 0.09 | FALSE | 0.63 | FALSE | 0.2 | FALSE |
| Gasar | 20 | 2008 | 9 | Cu | 0.33 | FALSE | 0.26 | FALSE | 0.03 | FALSE | 0.48 | FALSE | 0.08 | FALSE |
| Gasar | 20 | 2008 | 13.1 | Ni | 0.36 | FALSE | 0.63 | FALSE | 0.25 | FALSE | NA | NA | NA | NA |
| Gasar | 20 | 2008 | 0.93 | Pb | 0.02 | FALSE | 0.02 | FALSE | 0 | FALSE | 0.03 | FALSE | 0.01 | FALSE |
| Gasar | 20 | 2008 | 8.2 | Zn | 0.07 | FALSE | 0.05 | FALSE | 0.02 | FALSE | 0.07 | FALSE | 0.03 | FALSE |
| Gasar | 20 | 2007 | 15.9 | Cr | 0.2 | FALSE | 0.2 | FALSE | 0.04 | FALSE | 0.3 | FALSE | 0.1 | FALSE |
| Gasar | 20 | 2007 | 9.7 | Cu | 0.36 | FALSE | 0.29 | FALSE | 0.04 | FALSE | 0.52 | FALSE | 0.09 | FALSE |
| Gasar | 20 | 2007 | 12.1 | Ni | 0.34 | FALSE | 0.58 | FALSE | 0.24 | FALSE | NA | NA | NA | NA |
| Gasar | 20 | 2007 | 1.11 | Pb | 0.03 | FALSE | 0.02 | FALSE | 0.01 | FALSE | 0.04 | FALSE | 0.01 | FALSE |
| Umm Al-Na'asan | 21 | 2020 | 0.039 | Cd | 0.13 | FALSE | 0.03 | FALSE | 0 | FALSE | 0.06 | FALSE | 0.01 | FALSE |
| Umm Al-Na'asan | 21 | 2020 | 66.2 | Cr | 0.82 | FALSE | 0.82 | FALSE | 0.18 | FALSE | 1.27 | TRUE | 0.41 | FALSE |
| Umm Al-Na'asan | 21 | 2020 | 10.4 | Cu | 0.38 | FALSE | 0.3 | FALSE | 0.04 | FALSE | 0.55 | FALSE | 0.1 | FALSE |
| Umm Al-Na'asan | 21 | 2020 | 29.2 | Ni | 0.81 | FALSE | 1.4 | TRUE | 0.57 | FALSE | NA | NA | NA | NA |
| Umm Al-Na'asan | 21 | 2020 | 1.05 | Pb | 0.03 | FALSE | 0.02 | FALSE | 0 | FALSE | 0.03 | FALSE | 0.01 | FALSE |
| Umm Al-Na'asan | 21 | 2020 | 25.2 | Zn | 0.21 | FALSE | 0.17 | FALSE | 0.06 | FALSE | 0.2 | FALSE | 0.09 | FALSE |
| Umm Al-Na'asan | 21 | 2019 | 0.06 | Cd | 0.19 | FALSE | 0.05 | FALSE | 0.01 | FALSE | 0.09 | FALSE | 0.01 | FALSE |
| Umm Al-Na'asan | 21 | 2019 | 66.5 | Cr | 0.82 | FALSE | 0.82 | FALSE | 0.18 | FALSE | 1.27 | TRUE | 0.42 | FALSE |
| Umm Al-Na'asan | 21 | 2019 | 9.2 | Cu | 0.34 | FALSE | 0.27 | FALSE | 0.03 | FALSE | 0.49 | FALSE | 0.09 | FALSE |
| Umm Al-Na'asan | 21 | 2019 | 29.9 | Ni | 0.83 | FALSE | 1.43 | TRUE | 0.58 | FALSE | NA | NA | NA | NA |
| Umm Al-Na'asan | 21 | 2019 | 3.21 | Pb | 0.08 | FALSE | 0.07 | FALSE | 0.01 | FALSE | 0.11 | FALSE | 0.03 | FALSE |
| Umm Al-Na'asan | 21 | 2019 | 24.3 | Zn | 0.2 | FALSE | 0.16 | FALSE | 0.06 | FALSE | 0.2 | FALSE | 0.09 | FALSE |
| Umm Al-Na'asan | 21 | 2018 | 0.07 | Cd | 0.23 | FALSE | 0.06 | FALSE | 0.01 | FALSE | 0.1 | FALSE | 0.02 | FALSE |
| Umm Al-Na'asan | 21 | 2018 | 74.5 | Cr | 0.92 | FALSE | 0.92 | FALSE | 0.2 | FALSE | 1.42 | TRUE | 0.47 | FALSE |
| Umm Al-Na'asan | 21 | 2018 | 9.3 | Cu | 0.34 | FALSE | 0.27 | FALSE | 0.03 | FALSE | 0.5 | FALSE | 0.09 | FALSE |
| Umm Al-Na'asan | 21 | 2018 | 26.7 | Ni | 0.74 | FALSE | 1.28 | TRUE | 0.52 | FALSE | NA | NA | NA | NA |
| Umm Al-Na'asan | 21 | 2018 | 1 | Pb | 0.03 | FALSE | 0.02 | FALSE | 0 | FALSE | 0.03 | FALSE | 0.01 | FALSE |
| Umm Al-Na'asan | 21 | 2018 | 22.4 | Zn | 0.18 | FALSE | 0.15 | FALSE | 0.05 | FALSE | 0.18 | FALSE | 0.08 | FALSE |
| Umm Al-Na'asan | 21 | 2017 | 0.084 | Cd | 0.27 | FALSE | 0.07 | FALSE | 0.01 | FALSE | 0.12 | FALSE | 0.02 | FALSE |
| Umm Al-Na'asan | 21 | 2017 | 87.9 | Cr | 1.08 | TRUE | 1.08 | TRUE | 0.24 | FALSE | 1.68 | TRUE | 0.55 | FALSE |
| Umm Al-Na'asan | 21 | 2017 | 11.1 | Cu | 0.41 | FALSE | 0.33 | FALSE | 0.04 | FALSE | 0.6 | FALSE | 0.1 | FALSE |
| Umm Al-Na'asan | 21 | 2017 | 28 | Ni | 0.78 | FALSE | 1.34 | TRUE | 0.54 | FALSE | NA | NA | NA | NA |
| Umm Al-Na'asan | 21 | 2017 | 1.92 | Pb | 0.05 | FALSE | 0.04 | FALSE | 0.01 | FALSE | 0.06 | FALSE | 0.02 | FALSE |
| Umm Al-Na'asan | 21 | 2017 | 21.3 | Zn | 0.17 | FALSE | 0.14 | FALSE | 0.05 | FALSE | 0.17 | FALSE | 0.08 | FALSE |
| Umm Al-Na'asan | 21 | 2016 | 0.098 | Cd | 0.32 | FALSE | 0.08 | FALSE | 0.01 | FALSE | 0.14 | FALSE | 0.02 | FALSE |
| Umm Al-Na'asan | 21 | 2016 | 66.2 | Cr | 0.82 | FALSE | 0.82 | FALSE | 0.18 | FALSE | 1.27 | TRUE | 0.41 | FALSE |
| Umm Al-Na'asan | 21 | 2016 | 12.4 | Cu | 0.46 | FALSE | 0.36 | FALSE | 0.05 | FALSE | 0.66 | FALSE | 0.11 | FALSE |
| Umm Al-Na'asan | 21 | 2016 | 37.9 | Ni | 1.05 | TRUE | 1.81 | TRUE | 0.73 | FALSE | NA | NA | NA | NA |
| Umm Al-Na'asan | 21 | 2016 | 1.79 | Pb | 0.05 | FALSE | 0.04 | FALSE | 0.01 | FALSE | 0.06 | FALSE | 0.02 | FALSE |
| Umm Al-Na'asan | 21 | 2016 | 17.4 | Zn | 0.14 | FALSE | 0.12 | FALSE | 0.04 | FALSE | 0.14 | FALSE | 0.06 | FALSE |
| Umm Al-Na'asan | 21 | 2015 | 0.064 | Cd | 0.21 | FALSE | 0.05 | FALSE | 0.01 | FALSE | 0.09 | FALSE | 0.02 | FALSE |
| Umm Al-Na'asan | 21 | 2015 | 68 | Cr | 0.84 | FALSE | 0.84 | FALSE | 0.18 | FALSE | 1.3 | TRUE | 0.42 | FALSE |
| Umm Al-Na'asan | 21 | 2015 | 11.8 | Cu | 0.44 | FALSE | 0.35 | FALSE | 0.04 | FALSE | 0.63 | FALSE | 0.11 | FALSE |
| Umm Al-Na'asan | 21 | 2015 | 39.3 | Ni | 1.09 | TRUE | 1.88 | TRUE | 0.76 | FALSE | NA | NA | NA | NA |
| Umm Al-Na'asan | 21 | 2015 | 1.49 | Pb | 0.04 | FALSE | 0.03 | FALSE | 0.01 | FALSE | 0.05 | FALSE | 0.01 | FALSE |
| Umm Al-Na'asan | 21 | 2015 | 14.7 | Zn | 0.12 | FALSE | 0.1 | FALSE | 0.04 | FALSE | 0.12 | FALSE | 0.05 | FALSE |
| Umm Al-Na'asan | 21 | 2014 | 0.099 | Cd | 0.32 | FALSE | 0.08 | FALSE | 0.01 | FALSE | 0.14 | FALSE | 0.02 | FALSE |
| Umm Al-Na'asan | 21 | 2014 | 64.3 | Cr | 0.79 | FALSE | 0.79 | FALSE | 0.17 | FALSE | 1.23 | TRUE | 0.4 | FALSE |
| Umm Al-Na'asan | 21 | 2014 | 13.4 | Cu | 0.5 | FALSE | 0.39 | FALSE | 0.05 | FALSE | 0.72 | FALSE | 0.12 | FALSE |
| Umm Al-Na'asan | 21 | 2014 | 54.4 | Ni | 1.51 | TRUE | 2.6 | TRUE | 1.05 | TRUE | NA | NA | NA | NA |
| Umm Al-Na'asan | 21 | 2014 | 2.46 | Pb | 0.06 | FALSE | 0.05 | FALSE | 0.01 | FALSE | 0.08 | FALSE | 0.02 | FALSE |
| Umm Al-Na'asan | 21 | 2014 | 22.7 | Zn | 0.19 | FALSE | 0.15 | FALSE | 0.06 | FALSE | 0.18 | FALSE | 0.08 | FALSE |
| Umm Al-Na'asan | 21 | 2013 | 0.077 | Cd | 0.25 | FALSE | 0.06 | FALSE | 0.01 | FALSE | 0.11 | FALSE | 0.02 | FALSE |
| Umm Al-Na'asan | 21 | 2013 | 57.6 | Cr | 0.71 | FALSE | 0.71 | FALSE | 0.16 | FALSE | 1.1 | TRUE | 0.36 | FALSE |
| Umm Al-Na'asan | 21 | 2013 | 11.5 | Cu | 0.42 | FALSE | 0.34 | FALSE | 0.04 | FALSE | 0.61 | FALSE | 0.11 | FALSE |
| Umm Al-Na'asan | 21 | 2013 | 34.1 | Ni | 0.95 | FALSE | 1.63 | TRUE | 0.66 | FALSE | NA | NA | NA | NA |
| Umm Al-Na'asan | 21 | 2013 | 3 | Pb | 0.08 | FALSE | 0.06 | FALSE | 0.01 | FALSE | 0.1 | FALSE | 0.03 | FALSE |
| Umm Al-Na'asan | 21 | 2013 | 40.9 | Zn | 0.34 | FALSE | 0.27 | FALSE | 0.1 | FALSE | 0.33 | FALSE | 0.15 | FALSE |
| Umm Al-Na'asan | 21 | 2012 | 0.051 | Cd | 0.17 | FALSE | 0.04 | FALSE | 0.01 | FALSE | 0.07 | FALSE | 0.01 | FALSE |
| Umm Al-Na'asan | 21 | 2012 | 61.4 | Cr | 0.76 | FALSE | 0.76 | FALSE | 0.17 | FALSE | 1.17 | TRUE | 0.38 | FALSE |
| Umm Al-Na'asan | 21 | 2012 | 7.9 | Cu | 0.29 | FALSE | 0.23 | FALSE | 0.03 | FALSE | 0.42 | FALSE | 0.07 | FALSE |
| Umm Al-Na'asan | 21 | 2012 | 26 | Ni | 0.72 | FALSE | 1.24 | TRUE | 0.5 | FALSE | NA | NA | NA | NA |
| Umm Al-Na'asan | 21 | 2012 | 0.21 | Pb | 0.01 | FALSE | 0 | FALSE | 0 | FALSE | 0.01 | FALSE | 0 | FALSE |
| Umm Al-Na'asan | 21 | 2012 | 26 | Zn | 0.21 | FALSE | 0.17 | FALSE | 0.06 | FALSE | 0.21 | FALSE | 0.1 | FALSE |
| Umm Al-Na'asan | 21 | 2011 | 0.046 | Cd | 0.15 | FALSE | 0.04 | FALSE | 0 | FALSE | 0.07 | FALSE | 0.01 | FALSE |
| Umm Al-Na'asan | 21 | 2011 | 71.4 | Cr | 0.88 | FALSE | 0.88 | FALSE | 0.19 | FALSE | 1.37 | TRUE | 0.45 | FALSE |
| Umm Al-Na'asan | 21 | 2011 | 14.1 | Cu | 0.52 | FALSE | 0.42 | FALSE | 0.05 | FALSE | 0.76 | FALSE | 0.13 | FALSE |
| Umm Al-Na'asan | 21 | 2011 | 39.7 | Ni | 1.1 | TRUE | 1.9 | TRUE | 0.77 | FALSE | NA | NA | NA | NA |
| Umm Al-Na'asan | 21 | 2011 | 0.69 | Pb | 0.02 | FALSE | 0.01 | FALSE | 0 | FALSE | 0.02 | FALSE | 0.01 | FALSE |
| Umm Al-Na'asan | 21 | 2011 | 38.3 | Zn | 0.31 | FALSE | 0.26 | FALSE | 0.09 | FALSE | 0.31 | FALSE | 0.14 | FALSE |
| Umm Al-Na'asan | 21 | 2010 | 0.13 | Cd | 0.42 | FALSE | 0.11 | FALSE | 0.01 | FALSE | 0.19 | FALSE | 0.03 | FALSE |
| Umm Al-Na'asan | 21 | 2010 | 80.3 | Cr | 0.99 | FALSE | 0.99 | FALSE | 0.22 | FALSE | 1.54 | TRUE | 0.5 | FALSE |
| Umm Al-Na'asan | 21 | 2010 | 9.4 | Cu | 0.35 | FALSE | 0.28 | FALSE | 0.03 | FALSE | 0.5 | FALSE | 0.09 | FALSE |
| Umm Al-Na'asan | 21 | 2010 | 21.2 | Ni | 0.59 | FALSE | 1.01 | TRUE | 0.41 | FALSE | NA | NA | NA | NA |
| Umm Al-Na'asan | 21 | 2010 | 1.21 | Pb | 0.03 | FALSE | 0.03 | FALSE | 0.01 | FALSE | 0.04 | FALSE | 0.01 | FALSE |
| Umm Al-Na'asan | 21 | 2010 | 21 | Zn | 0.17 | FALSE | 0.14 | FALSE | 0.05 | FALSE | 0.17 | FALSE | 0.08 | FALSE |
| Umm Al-Na'asan | 21 | 2009 | 0.128 | Cd | 0.41 | FALSE | 0.11 | FALSE | 0.01 | FALSE | 0.18 | FALSE | 0.03 | FALSE |
| Umm Al-Na'asan | 21 | 2009 | 67.4 | Cr | 0.83 | FALSE | 0.83 | FALSE | 0.18 | FALSE | 1.29 | TRUE | 0.42 | FALSE |
| Umm Al-Na'asan | 21 | 2009 | 11.2 | Cu | 0.41 | FALSE | 0.33 | FALSE | 0.04 | FALSE | 0.6 | FALSE | 0.1 | FALSE |
| Umm Al-Na'asan | 21 | 2009 | 31.4 | Ni | 0.87 | FALSE | 1.5 | TRUE | 0.61 | FALSE | NA | NA | NA | NA |
| Umm Al-Na'asan | 21 | 2009 | 1.66 | Pb | 0.04 | FALSE | 0.04 | FALSE | 0.01 | FALSE | 0.05 | FALSE | 0.01 | FALSE |
| Umm Al-Na'asan | 21 | 2009 | 15.5 | Zn | 0.13 | FALSE | 0.1 | FALSE | 0.04 | FALSE | 0.12 | FALSE | 0.06 | FALSE |
| Umm Al-Na'asan | 21 | 2008 | 0.035 | Cd | 0.11 | FALSE | 0.03 | FALSE | 0 | FALSE | 0.05 | FALSE | 0.01 | FALSE |
| Umm Al-Na'asan | 21 | 2008 | 61.3 | Cr | 0.76 | FALSE | 0.76 | FALSE | 0.17 | FALSE | 1.17 | TRUE | 0.38 | FALSE |
| Umm Al-Na'asan | 21 | 2008 | 7.1 | Cu | 0.26 | FALSE | 0.21 | FALSE | 0.03 | FALSE | 0.38 | FALSE | 0.07 | FALSE |
| Umm Al-Na'asan | 21 | 2008 | 28.5 | Ni | 0.79 | FALSE | 1.36 | TRUE | 0.55 | FALSE | NA | NA | NA | NA |
| Umm Al-Na'asan | 21 | 2008 | 1.12 | Pb | 0.03 | FALSE | 0.02 | FALSE | 0.01 | FALSE | 0.04 | FALSE | 0.01 | FALSE |
| Umm Al-Na'asan | 21 | 2008 | 10.1 | Zn | 0.08 | FALSE | 0.07 | FALSE | 0.02 | FALSE | 0.08 | FALSE | 0.04 | FALSE |
| Umm Al-Na'asan | 21 | 2007 | 64.1 | Cr | 0.79 | FALSE | 0.79 | FALSE | 0.17 | FALSE | 1.23 | TRUE | 0.4 | FALSE |
| Umm Al-Na'asan | 21 | 2007 | 15.1 | Cu | 0.56 | FALSE | 0.44 | FALSE | 0.06 | FALSE | 0.81 | FALSE | 0.14 | FALSE |
| Umm Al-Na'asan | 21 | 2007 | 30.3 | Ni | 0.84 | FALSE | 1.45 | TRUE | 0.59 | FALSE | NA | NA | NA | NA |
| Umm Al-Na'asan | 21 | 2007 | 2.47 | Pb | 0.06 | FALSE | 0.05 | FALSE | 0.01 | FALSE | 0.08 | FALSE | 0.02 | FALSE |
| Umm Al-Na'asan | 21 | 2007 | 10.9 | Zn | 0.09 | FALSE | 0.07 | FALSE | 0.03 | FALSE | 0.09 | FALSE | 0.04 | FALSE |
| Ya'suf | 22 | 2020 | 0.075 | Cd | 0.24 | FALSE | 0.06 | FALSE | 0.01 | FALSE | 0.11 | FALSE | 0.02 | FALSE |
| Ya'suf | 22 | 2020 | 36.7 | Cr | 0.45 | FALSE | 0.45 | FALSE | 0.1 | FALSE | 0.7 | FALSE | 0.23 | FALSE |
| Ya'suf | 22 | 2020 | 6.8 | Cu | 0.25 | FALSE | 0.2 | FALSE | 0.03 | FALSE | 0.36 | FALSE | 0.06 | FALSE |
| Ya'suf | 22 | 2020 | 16.5 | Ni | 0.46 | FALSE | 0.79 | FALSE | 0.32 | FALSE | NA | NA | NA | NA |
| Ya'suf | 22 | 2020 | 0.61 | Pb | 0.02 | FALSE | 0.01 | FALSE | 0 | FALSE | 0.02 | FALSE | 0.01 | FALSE |
| Ya'suf | 22 | 2020 | 21.3 | Zn | 0.17 | FALSE | 0.14 | FALSE | 0.05 | FALSE | 0.17 | FALSE | 0.08 | FALSE |
| Ya'suf | 22 | 2019 | 0.055 | Cd | 0.18 | FALSE | 0.05 | FALSE | 0.01 | FALSE | 0.08 | FALSE | 0.01 | FALSE |
| Ya'suf | 22 | 2019 | 69.4 | Cr | 0.86 | FALSE | 0.86 | FALSE | 0.19 | FALSE | 1.33 | TRUE | 0.43 | FALSE |
| Ya'suf | 22 | 2019 | 6.6 | Cu | 0.24 | FALSE | 0.19 | FALSE | 0.02 | FALSE | 0.35 | FALSE | 0.06 | FALSE |
| Ya'suf | 22 | 2019 | 22.1 | Ni | 0.61 | FALSE | 1.06 | TRUE | 0.43 | FALSE | NA | NA | NA | NA |
| Ya'suf | 22 | 2019 | 4.25 | Pb | 0.11 | FALSE | 0.09 | FALSE | 0.02 | FALSE | 0.14 | FALSE | 0.04 | FALSE |
| Ya'suf | 22 | 2019 | 24.3 | Zn | 0.2 | FALSE | 0.16 | FALSE | 0.06 | FALSE | 0.2 | FALSE | 0.09 | FALSE |
| Ya'suf | 22 | 2018 | 0.072 | Cd | 0.23 | FALSE | 0.06 | FALSE | 0.01 | FALSE | 0.1 | FALSE | 0.02 | FALSE |
| Ya'suf | 22 | 2018 | 137 | Cr | 1.69 | TRUE | 1.69 | TRUE | 0.37 | FALSE | 2.62 | TRUE | 0.86 | FALSE |
| Ya'suf | 22 | 2018 | 21 | Cu | 0.78 | FALSE | 0.62 | FALSE | 0.08 | FALSE | 1.12 | TRUE | 0.19 | FALSE |
| Ya'suf | 22 | 2018 | 18.4 | Ni | 0.51 | FALSE | 0.88 | FALSE | 0.36 | FALSE | NA | NA | NA | NA |
| Ya'suf | 22 | 2018 | 1.3 | Pb | 0.03 | FALSE | 0.03 | FALSE | 0.01 | FALSE | 0.04 | FALSE | 0.01 | FALSE |
| Ya'suf | 22 | 2018 | 22.1 | Zn | 0.18 | FALSE | 0.15 | FALSE | 0.05 | FALSE | 0.18 | FALSE | 0.08 | FALSE |
| Ya'suf | 22 | 2017 | 0.084 | Cd | 0.27 | FALSE | 0.07 | FALSE | 0.01 | FALSE | 0.12 | FALSE | 0.02 | FALSE |
| Ya'suf | 22 | 2017 | 163 | Cr | 2.01 | TRUE | 2.01 | TRUE | 0.44 | FALSE | 3.12 | TRUE | 1.02 | TRUE |
| Ya'suf | 22 | 2017 | 20.5 | Cu | 0.76 | FALSE | 0.6 | FALSE | 0.08 | FALSE | 1.09 | TRUE | 0.19 | FALSE |
| Ya'suf | 22 | 2017 | 26.9 | Ni | 0.75 | FALSE | 1.28 | TRUE | 0.52 | FALSE | NA | NA | NA | NA |
| Ya'suf | 22 | 2017 | 1.63 | Pb | 0.04 | FALSE | 0.03 | FALSE | 0.01 | FALSE | 0.05 | FALSE | 0.01 | FALSE |
| Ya'suf | 22 | 2017 | 20.1 | Zn | 0.17 | FALSE | 0.13 | FALSE | 0.05 | FALSE | 0.16 | FALSE | 0.07 | FALSE |
| Ya'suf | 22 | 2016 | 0.128 | Cd | 0.41 | FALSE | 0.11 | FALSE | 0.01 | FALSE | 0.18 | FALSE | 0.03 | FALSE |
| Ya'suf | 22 | 2016 | 50.3 | Cr | 0.62 | FALSE | 0.62 | FALSE | 0.14 | FALSE | 0.96 | FALSE | 0.31 | FALSE |
| Ya'suf | 22 | 2016 | 9.2 | Cu | 0.34 | FALSE | 0.27 | FALSE | 0.03 | FALSE | 0.49 | FALSE | 0.09 | FALSE |
| Ya'suf | 22 | 2016 | 25.6 | Ni | 0.71 | FALSE | 1.23 | TRUE | 0.5 | FALSE | NA | NA | NA | NA |
| Ya'suf | 22 | 2016 | 1.45 | Pb | 0.04 | FALSE | 0.03 | FALSE | 0.01 | FALSE | 0.05 | FALSE | 0.01 | FALSE |
| Ya'suf | 22 | 2016 | 12.9 | Zn | 0.11 | FALSE | 0.09 | FALSE | 0.03 | FALSE | 0.1 | FALSE | 0.05 | FALSE |
| Ya'suf | 22 | 2015 | 0.056 | Cd | 0.18 | FALSE | 0.05 | FALSE | 0.01 | FALSE | 0.08 | FALSE | 0.01 | FALSE |
| Ya'suf | 22 | 2015 | 222 | Cr | 2.75 | TRUE | 2.75 | TRUE | 0.6 | FALSE | 4.25 | TRUE | 1.39 | TRUE |
| Ya'suf | 22 | 2015 | 9.3 | Cu | 0.34 | FALSE | 0.27 | FALSE | 0.03 | FALSE | 0.5 | FALSE | 0.09 | FALSE |
| Ya'suf | 22 | 2015 | 26.2 | Ni | 0.73 | FALSE | 1.25 | TRUE | 0.51 | FALSE | NA | NA | NA | NA |
| Ya'suf | 22 | 2015 | 4.95 | Pb | 0.13 | FALSE | 0.11 | FALSE | 0.02 | FALSE | 0.16 | FALSE | 0.04 | FALSE |
| Ya'suf | 22 | 2015 | 12.7 | Zn | 0.1 | FALSE | 0.08 | FALSE | 0.03 | FALSE | 0.1 | FALSE | 0.05 | FALSE |
| Ya'suf | 22 | 2014 | 0.108 | Cd | 0.35 | FALSE | 0.09 | FALSE | 0.01 | FALSE | 0.15 | FALSE | 0.03 | FALSE |
| Ya'suf | 22 | 2014 | 54.1 | Cr | 0.67 | FALSE | 0.67 | FALSE | 0.15 | FALSE | 1.03 | TRUE | 0.34 | FALSE |
| Ya'suf | 22 | 2014 | 21 | Cu | 0.78 | FALSE | 0.62 | FALSE | 0.08 | FALSE | 1.12 | TRUE | 0.19 | FALSE |
| Ya'suf | 22 | 2014 | 44.9 | Ni | 1.25 | TRUE | 2.15 | TRUE | 0.87 | FALSE | NA | NA | NA | NA |
| Ya'suf | 22 | 2014 | 1.77 | Pb | 0.05 | FALSE | 0.04 | FALSE | 0.01 | FALSE | 0.06 | FALSE | 0.02 | FALSE |
| Ya'suf | 22 | 2014 | 21.7 | Zn | 0.18 | FALSE | 0.14 | FALSE | 0.05 | FALSE | 0.18 | FALSE | 0.08 | FALSE |
| Ya'suf | 22 | 2013 | 0.095 | Cd | 0.31 | FALSE | 0.08 | FALSE | 0.01 | FALSE | 0.14 | FALSE | 0.02 | FALSE |
| Ya'suf | 22 | 2013 | 35.9 | Cr | 0.44 | FALSE | 0.44 | FALSE | 0.1 | FALSE | 0.69 | FALSE | 0.22 | FALSE |
| Ya'suf | 22 | 2013 | 11.5 | Cu | 0.43 | FALSE | 0.34 | FALSE | 0.04 | FALSE | 0.62 | FALSE | 0.11 | FALSE |
| Ya'suf | 22 | 2013 | 29.7 | Ni | 0.82 | FALSE | 1.42 | TRUE | 0.57 | FALSE | NA | NA | NA | NA |
| Ya'suf | 22 | 2013 | 3.56 | Pb | 0.09 | FALSE | 0.08 | FALSE | 0.02 | FALSE | 0.12 | FALSE | 0.03 | FALSE |
| Ya'suf | 22 | 2013 | 16.7 | Zn | 0.14 | FALSE | 0.11 | FALSE | 0.04 | FALSE | 0.13 | FALSE | 0.06 | FALSE |
| Ya'suf | 22 | 2012 | 0.056 | Cd | 0.18 | FALSE | 0.05 | FALSE | 0.01 | FALSE | 0.08 | FALSE | 0.01 | FALSE |
| Ya'suf | 22 | 2012 | 66.7 | Cr | 0.82 | FALSE | 0.82 | FALSE | 0.18 | FALSE | 1.28 | TRUE | 0.42 | FALSE |
| Ya'suf | 22 | 2012 | 8.2 | Cu | 0.3 | FALSE | 0.24 | FALSE | 0.03 | FALSE | 0.44 | FALSE | 0.08 | FALSE |
| Ya'suf | 22 | 2012 | 23.6 | Ni | 0.66 | FALSE | 1.13 | TRUE | 0.46 | FALSE | NA | NA | NA | NA |
| Ya'suf | 22 | 2012 | 6.71 | Pb | 0.18 | FALSE | 0.14 | FALSE | 0.03 | FALSE | 0.22 | FALSE | 0.06 | FALSE |
| Ya'suf | 22 | 2012 | 23.6 | Zn | 0.19 | FALSE | 0.16 | FALSE | 0.06 | FALSE | 0.19 | FALSE | 0.09 | FALSE |
| Ya'suf | 22 | 2011 | 0.048 | Cd | 0.16 | FALSE | 0.04 | FALSE | 0.01 | FALSE | 0.07 | FALSE | 0.01 | FALSE |
| Ya'suf | 22 | 2011 | 67.5 | Cr | 0.83 | FALSE | 0.83 | FALSE | 0.18 | FALSE | 1.29 | TRUE | 0.42 | FALSE |
| Ya'suf | 22 | 2011 | 18.3 | Cu | 0.68 | FALSE | 0.54 | FALSE | 0.07 | FALSE | 0.98 | FALSE | 0.17 | FALSE |
| Ya'suf | 22 | 2011 | 31.5 | Ni | 0.88 | FALSE | 1.51 | TRUE | 0.61 | FALSE | NA | NA | NA | NA |
| Ya'suf | 22 | 2011 | 0.3 | Pb | 0.01 | FALSE | 0.01 | FALSE | 0 | FALSE | 0.01 | FALSE | 0 | FALSE |
| Ya'suf | 22 | 2011 | 37.7 | Zn | 0.31 | FALSE | 0.25 | FALSE | 0.09 | FALSE | 0.3 | FALSE | 0.14 | FALSE |
| Ya'suf | 22 | 2010 | 0.174 | Cd | 0.56 | FALSE | 0.14 | FALSE | 0.02 | FALSE | 0.25 | FALSE | 0.04 | FALSE |
| Ya'suf | 22 | 2010 | 80.8 | Cr | 1 | FALSE | 1 | FALSE | 0.22 | FALSE | 1.54 | TRUE | 0.5 | FALSE |
| Ya'suf | 22 | 2010 | 10.1 | Cu | 0.37 | FALSE | 0.3 | FALSE | 0.04 | FALSE | 0.54 | FALSE | 0.09 | FALSE |
| Ya'suf | 22 | 2010 | 21.1 | Ni | 0.59 | FALSE | 1.01 | TRUE | 0.41 | FALSE | NA | NA | NA | NA |
| Ya'suf | 22 | 2010 | 1.67 | Pb | 0.04 | FALSE | 0.04 | FALSE | 0.01 | FALSE | 0.06 | FALSE | 0.01 | FALSE |
| Ya'suf | 22 | 2010 | 27.1 | Zn | 0.22 | FALSE | 0.18 | FALSE | 0.07 | FALSE | 0.22 | FALSE | 0.1 | FALSE |
| Ya'suf | 22 | 2009 | 0.1 | Cd | 0.32 | FALSE | 0.08 | FALSE | 0.01 | FALSE | 0.14 | FALSE | 0.02 | FALSE |
| Ya'suf | 22 | 2009 | 96.1 | Cr | 1.19 | TRUE | 1.19 | TRUE | 0.26 | FALSE | 1.84 | TRUE | 0.6 | FALSE |
| Ya'suf | 22 | 2009 | 19.4 | Cu | 0.72 | FALSE | 0.57 | FALSE | 0.07 | FALSE | 1.04 | TRUE | 0.18 | FALSE |
| Ya'suf | 22 | 2009 | 23.7 | Ni | 0.66 | FALSE | 1.13 | TRUE | 0.46 | FALSE | NA | NA | NA | NA |
| Ya'suf | 22 | 2009 | 2.33 | Pb | 0.06 | FALSE | 0.05 | FALSE | 0.01 | FALSE | 0.08 | FALSE | 0.02 | FALSE |
| Ya'suf | 22 | 2009 | 16.6 | Zn | 0.14 | FALSE | 0.11 | FALSE | 0.04 | FALSE | 0.13 | FALSE | 0.06 | FALSE |
| Ya'suf | 22 | 2008 | 0.092 | Cd | 0.3 | FALSE | 0.08 | FALSE | 0.01 | FALSE | 0.13 | FALSE | 0.02 | FALSE |
| Ya'suf | 22 | 2008 | 528 | Cr | 6.52 | TRUE | 6.52 | TRUE | 1.43 | TRUE | 10.1 | TRUE | 3.3 | TRUE |
| Ya'suf | 22 | 2008 | 3.8 | Cu | 0.14 | FALSE | 0.11 | FALSE | 0.01 | FALSE | 0.21 | FALSE | 0.04 | FALSE |
| Ya'suf | 22 | 2008 | 12.5 | Ni | 0.35 | FALSE | 0.6 | FALSE | 0.24 | FALSE | NA | NA | NA | NA |
| Ya'suf | 22 | 2008 | 1.85 | Pb | 0.05 | FALSE | 0.04 | FALSE | 0.01 | FALSE | 0.06 | FALSE | 0.02 | FALSE |
| Ya'suf | 22 | 2008 | 9.9 | Zn | 0.08 | FALSE | 0.07 | FALSE | 0.02 | FALSE | 0.08 | FALSE | 0.04 | FALSE |
| Ya'suf | 22 | 2007 | 40.6 | Cr | 0.5 | FALSE | 0.5 | FALSE | 0.11 | FALSE | 0.78 | FALSE | 0.25 | FALSE |
| Ya'suf | 22 | 2007 | 3.8 | Cu | 0.14 | FALSE | 0.11 | FALSE | 0.01 | FALSE | 0.2 | FALSE | 0.03 | FALSE |
| Ya'suf | 22 | 2007 | 9.2 | Ni | 0.25 | FALSE | 0.44 | FALSE | 0.18 | FALSE | NA | NA | NA | NA |
| Ya'suf | 22 | 2007 | 7.4 | Pb | 0.19 | FALSE | 0.16 | FALSE | 0.03 | FALSE | 0.25 | FALSE | 0.07 | FALSE |
| Ya'suf | 22 | 2007 | 0.7 | Zn | 0.01 | FALSE | 0 | FALSE | 0 | FALSE | 0.01 | FALSE | 0 | FALSE |
| Al-Jasra | 23 | 2020 | 0.101 | Cd | 0.32 | FALSE | 0.08 | FALSE | 0.01 | FALSE | 0.14 | FALSE | 0.02 | FALSE |
| Al-Jasra | 23 | 2020 | 77.2 | Cr | 0.95 | FALSE | 0.95 | FALSE | 0.21 | FALSE | 1.48 | TRUE | 0.48 | FALSE |
| Al-Jasra | 23 | 2020 | 276 | Cu | 10.21 | TRUE | 8.11 | TRUE | 1.02 | TRUE | 14.74 | TRUE | 2.55 | TRUE |
| Al-Jasra | 23 | 2020 | 14.5 | Ni | 0.4 | FALSE | 0.69 | FALSE | 0.28 | FALSE | NA | NA | NA | NA |
| Al-Jasra | 23 | 2020 | 1.69 | Pb | 0.04 | FALSE | 0.04 | FALSE | 0.01 | FALSE | 0.06 | FALSE | 0.02 | FALSE |
| Al-Jasra | 23 | 2020 | 37.5 | Zn | 0.31 | FALSE | 0.25 | FALSE | 0.09 | FALSE | 0.3 | FALSE | 0.14 | FALSE |
| Al-Jasra | 23 | 2019 | 0.069 | Cd | 0.22 | FALSE | 0.06 | FALSE | 0.01 | FALSE | 0.1 | FALSE | 0.02 | FALSE |
| Al-Jasra | 23 | 2019 | 85.2 | Cr | 1.05 | TRUE | 1.05 | TRUE | 0.23 | FALSE | 1.63 | TRUE | 0.53 | FALSE |
| Al-Jasra | 23 | 2019 | 6.5 | Cu | 0.24 | FALSE | 0.19 | FALSE | 0.02 | FALSE | 0.35 | FALSE | 0.06 | FALSE |
| Al-Jasra | 23 | 2019 | 17.2 | Ni | 0.48 | FALSE | 0.82 | FALSE | 0.33 | FALSE | NA | NA | NA | NA |
| Al-Jasra | 23 | 2019 | 4.15 | Pb | 0.11 | FALSE | 0.09 | FALSE | 0.02 | FALSE | 0.14 | FALSE | 0.04 | FALSE |
| Al-Jasra | 23 | 2019 | 33.7 | Zn | 0.28 | FALSE | 0.22 | FALSE | 0.08 | FALSE | 0.27 | FALSE | 0.12 | FALSE |
| Al-Jasra | 23 | 2018 | 0.077 | Cd | 0.25 | FALSE | 0.06 | FALSE | 0.01 | FALSE | 0.11 | FALSE | 0.02 | FALSE |
| Al-Jasra | 23 | 2018 | 176 | Cr | 2.17 | TRUE | 2.17 | TRUE | 0.48 | FALSE | 3.36 | TRUE | 1.1 | TRUE |
| Al-Jasra | 23 | 2018 | 5.8 | Cu | 0.21 | FALSE | 0.17 | FALSE | 0.02 | FALSE | 0.31 | FALSE | 0.05 | FALSE |
| Al-Jasra | 23 | 2018 | 15.8 | Ni | 0.44 | FALSE | 0.75 | FALSE | 0.31 | FALSE | NA | NA | NA | NA |
| Al-Jasra | 23 | 2018 | 1.2 | Pb | 0.03 | FALSE | 0.03 | FALSE | 0.01 | FALSE | 0.04 | FALSE | 0.01 | FALSE |
| Al-Jasra | 23 | 2018 | 27.9 | Zn | 0.23 | FALSE | 0.19 | FALSE | 0.07 | FALSE | 0.23 | FALSE | 0.1 | FALSE |
| Al-Jasra | 23 | 2017 | 0.102 | Cd | 0.33 | FALSE | 0.08 | FALSE | 0.01 | FALSE | 0.15 | FALSE | 0.02 | FALSE |
| Al-Jasra | 23 | 2017 | 72 | Cr | 0.89 | FALSE | 0.89 | FALSE | 0.19 | FALSE | 1.38 | TRUE | 0.45 | FALSE |
| Al-Jasra | 23 | 2017 | 26.4 | Cu | 0.98 | FALSE | 0.78 | FALSE | 0.1 | FALSE | 1.41 | TRUE | 0.24 | FALSE |
| Al-Jasra | 23 | 2017 | 23.6 | Ni | 0.65 | FALSE | 1.13 | TRUE | 0.46 | FALSE | NA | NA | NA | NA |
| Al-Jasra | 23 | 2017 | 1.34 | Pb | 0.04 | FALSE | 0.03 | FALSE | 0.01 | FALSE | 0.04 | FALSE | 0.01 | FALSE |
| Al-Jasra | 23 | 2017 | 27.2 | Zn | 0.22 | FALSE | 0.18 | FALSE | 0.07 | FALSE | 0.22 | FALSE | 0.1 | FALSE |
| Al-Jasra | 23 | 2016 | 0.138 | Cd | 0.45 | FALSE | 0.12 | FALSE | 0.01 | FALSE | 0.2 | FALSE | 0.03 | FALSE |
| Al-Jasra | 23 | 2016 | 240 | Cr | 2.96 | TRUE | 2.96 | TRUE | 0.65 | FALSE | 4.59 | TRUE | 1.5 | TRUE |
| Al-Jasra | 23 | 2016 | 11.5 | Cu | 0.43 | FALSE | 0.34 | FALSE | 0.04 | FALSE | 0.61 | FALSE | 0.11 | FALSE |
| Al-Jasra | 23 | 2016 | 14.3 | Ni | 0.4 | FALSE | 0.68 | FALSE | 0.28 | FALSE | NA | NA | NA | NA |
| Al-Jasra | 23 | 2016 | 2.04 | Pb | 0.05 | FALSE | 0.04 | FALSE | 0.01 | FALSE | 0.07 | FALSE | 0.02 | FALSE |
| Al-Jasra | 23 | 2016 | 25.9 | Zn | 0.21 | FALSE | 0.17 | FALSE | 0.06 | FALSE | 0.21 | FALSE | 0.1 | FALSE |
| Al-Jasra | 23 | 2015 | 0.067 | Cd | 0.22 | FALSE | 0.06 | FALSE | 0.01 | FALSE | 0.1 | FALSE | 0.02 | FALSE |
| Al-Jasra | 23 | 2015 | 181 | Cr | 2.24 | TRUE | 2.24 | TRUE | 0.49 | FALSE | 3.46 | TRUE | 1.13 | TRUE |
| Al-Jasra | 23 | 2015 | 11.7 | Cu | 0.43 | FALSE | 0.34 | FALSE | 0.04 | FALSE | 0.62 | FALSE | 0.11 | FALSE |
| Al-Jasra | 23 | 2015 | 16.1 | Ni | 0.45 | FALSE | 0.77 | FALSE | 0.31 | FALSE | NA | NA | NA | NA |
| Al-Jasra | 23 | 2015 | 4.83 | Pb | 0.13 | FALSE | 0.1 | FALSE | 0.02 | FALSE | 0.16 | FALSE | 0.04 | FALSE |
| Al-Jasra | 23 | 2015 | 19.5 | Zn | 0.16 | FALSE | 0.13 | FALSE | 0.05 | FALSE | 0.16 | FALSE | 0.07 | FALSE |
| Al-Jasra | 23 | 2014 | 0.114 | Cd | 0.37 | FALSE | 0.1 | FALSE | 0.01 | FALSE | 0.16 | FALSE | 0.03 | FALSE |
| Al-Jasra | 23 | 2014 | 49 | Cr | 0.61 | FALSE | 0.61 | FALSE | 0.13 | FALSE | 0.94 | FALSE | 0.31 | FALSE |
| Al-Jasra | 23 | 2014 | 14.5 | Cu | 0.54 | FALSE | 0.43 | FALSE | 0.05 | FALSE | 0.77 | FALSE | 0.13 | FALSE |
| Al-Jasra | 23 | 2014 | 30 | Ni | 0.83 | FALSE | 1.44 | TRUE | 0.58 | FALSE | NA | NA | NA | NA |
| Al-Jasra | 23 | 2014 | 1.78 | Pb | 0.05 | FALSE | 0.04 | FALSE | 0.01 | FALSE | 0.06 | FALSE | 0.02 | FALSE |
| Al-Jasra | 23 | 2014 | 30.6 | Zn | 0.25 | FALSE | 0.2 | FALSE | 0.07 | FALSE | 0.25 | FALSE | 0.11 | FALSE |
| Al-Jasra | 23 | 2013 | 0.107 | Cd | 0.35 | FALSE | 0.09 | FALSE | 0.01 | FALSE | 0.15 | FALSE | 0.03 | FALSE |
| Al-Jasra | 23 | 2013 | 34.7 | Cr | 0.43 | FALSE | 0.43 | FALSE | 0.09 | FALSE | 0.66 | FALSE | 0.22 | FALSE |
| Al-Jasra | 23 | 2013 | 24 | Cu | 0.89 | FALSE | 0.71 | FALSE | 0.09 | FALSE | 1.29 | TRUE | 0.22 | FALSE |
| Al-Jasra | 23 | 2013 | 18 | Ni | 0.5 | FALSE | 0.86 | FALSE | 0.35 | FALSE | NA | NA | NA | NA |
| Al-Jasra | 23 | 2013 | 4.73 | Pb | 0.12 | FALSE | 0.1 | FALSE | 0.02 | FALSE | 0.16 | FALSE | 0.04 | FALSE |
| Al-Jasra | 23 | 2013 | 24.7 | Zn | 0.2 | FALSE | 0.16 | FALSE | 0.06 | FALSE | 0.2 | FALSE | 0.09 | FALSE |
| Al-Jasra | 23 | 2012 | 0.065 | Cd | 0.21 | FALSE | 0.05 | FALSE | 0.01 | FALSE | 0.09 | FALSE | 0.02 | FALSE |
| Al-Jasra | 23 | 2012 | 70.6 | Cr | 0.87 | FALSE | 0.87 | FALSE | 0.19 | FALSE | 1.35 | TRUE | 0.44 | FALSE |
| Al-Jasra | 23 | 2012 | 8.6 | Cu | 0.32 | FALSE | 0.25 | FALSE | 0.03 | FALSE | 0.46 | FALSE | 0.08 | FALSE |
| Al-Jasra | 23 | 2012 | 35.3 | Ni | 0.98 | FALSE | 1.69 | TRUE | 0.68 | FALSE | NA | NA | NA | NA |
| Al-Jasra | 23 | 2012 | 1.93 | Pb | 0.05 | FALSE | 0.04 | FALSE | 0.01 | FALSE | 0.06 | FALSE | 0.02 | FALSE |
| Al-Jasra | 23 | 2012 | 35.3 | Zn | 0.29 | FALSE | 0.24 | FALSE | 0.09 | FALSE | 0.28 | FALSE | 0.13 | FALSE |
| Al-Jasra | 23 | 2011 | 0.081 | Cd | 0.26 | FALSE | 0.07 | FALSE | 0.01 | FALSE | 0.12 | FALSE | 0.02 | FALSE |
| Al-Jasra | 23 | 2011 | 45.7 | Cr | 0.56 | FALSE | 0.56 | FALSE | 0.12 | FALSE | 0.87 | FALSE | 0.29 | FALSE |
| Al-Jasra | 23 | 2011 | 15.8 | Cu | 0.59 | FALSE | 0.47 | FALSE | 0.06 | FALSE | 0.85 | FALSE | 0.15 | FALSE |
| Al-Jasra | 23 | 2011 | 27.9 | Ni | 0.77 | FALSE | 1.33 | TRUE | 0.54 | FALSE | NA | NA | NA | NA |
| Al-Jasra | 23 | 2011 | 0.42 | Pb | 0.01 | FALSE | 0.01 | FALSE | 0 | FALSE | 0.01 | FALSE | 0 | FALSE |
| Al-Jasra | 23 | 2011 | 46.6 | Zn | 0.38 | FALSE | 0.31 | FALSE | 0.11 | FALSE | 0.38 | FALSE | 0.17 | FALSE |
| Al-Jasra | 23 | 2010 | 0.114 | Cd | 0.37 | FALSE | 0.1 | FALSE | 0.01 | FALSE | 0.16 | FALSE | 0.03 | FALSE |
| Al-Jasra | 23 | 2010 | 131 | Cr | 1.62 | TRUE | 1.62 | TRUE | 0.35 | FALSE | 2.51 | TRUE | 0.82 | FALSE |
| Al-Jasra | 23 | 2010 | 11 | Cu | 0.41 | FALSE | 0.32 | FALSE | 0.04 | FALSE | 0.59 | FALSE | 0.1 | FALSE |
| Al-Jasra | 23 | 2010 | 29 | Ni | 0.8 | FALSE | 1.39 | TRUE | 0.56 | FALSE | NA | NA | NA | NA |
| Al-Jasra | 23 | 2010 | 2.07 | Pb | 0.05 | FALSE | 0.04 | FALSE | 0.01 | FALSE | 0.07 | FALSE | 0.02 | FALSE |
| Al-Jasra | 23 | 2010 | 29.1 | Zn | 0.24 | FALSE | 0.19 | FALSE | 0.07 | FALSE | 0.23 | FALSE | 0.11 | FALSE |
| Al-Jasra | 23 | 2009 | 0.313 | Cd | 1.01 | TRUE | 0.26 | FALSE | 0.03 | FALSE | 0.45 | FALSE | 0.07 | FALSE |
| Al-Jasra | 23 | 2009 | 92.6 | Cr | 1.14 | TRUE | 1.14 | TRUE | 0.25 | FALSE | 1.77 | TRUE | 0.58 | FALSE |
| Al-Jasra | 23 | 2009 | 57.3 | Cu | 2.12 | TRUE | 1.69 | TRUE | 0.21 | FALSE | 3.06 | TRUE | 0.53 | FALSE |
| Al-Jasra | 23 | 2009 | 17.4 | Ni | 0.48 | FALSE | 0.83 | FALSE | 0.34 | FALSE | NA | NA | NA | NA |
| Al-Jasra | 23 | 2009 | 2.08 | Pb | 0.05 | FALSE | 0.04 | FALSE | 0.01 | FALSE | 0.07 | FALSE | 0.02 | FALSE |
| Al-Jasra | 23 | 2009 | 24.6 | Zn | 0.2 | FALSE | 0.16 | FALSE | 0.06 | FALSE | 0.2 | FALSE | 0.09 | FALSE |
| Al-Jasra | 23 | 2008 | 0.139 | Cd | 0.45 | FALSE | 0.12 | FALSE | 0.01 | FALSE | 0.2 | FALSE | 0.03 | FALSE |
| Al-Jasra | 23 | 2008 | 57 | Cr | 0.7 | FALSE | 0.7 | FALSE | 0.15 | FALSE | 1.09 | TRUE | 0.36 | FALSE |
| Al-Jasra | 23 | 2008 | 29.2 | Cu | 1.08 | TRUE | 0.86 | FALSE | 0.11 | FALSE | 1.56 | TRUE | 0.27 | FALSE |
| Al-Jasra | 23 | 2008 | 9.1 | Ni | 0.25 | FALSE | 0.43 | FALSE | 0.18 | FALSE | NA | NA | NA | NA |
| Al-Jasra | 23 | 2008 | 1.67 | Pb | 0.04 | FALSE | 0.04 | FALSE | 0.01 | FALSE | 0.06 | FALSE | 0.01 | FALSE |
| Al-Jasra | 23 | 2008 | 17.2 | Zn | 0.14 | FALSE | 0.11 | FALSE | 0.04 | FALSE | 0.14 | FALSE | 0.06 | FALSE |
| Al-Jasra | 23 | 2007 | 107 | Cr | 1.33 | TRUE | 1.33 | TRUE | 0.29 | FALSE | 2.05 | TRUE | 0.67 | FALSE |
| Al-Jasra | 23 | 2007 | 29 | Cu | 1.07 | TRUE | 0.85 | FALSE | 0.11 | FALSE | 1.55 | TRUE | 0.27 | FALSE |
| Al-Jasra | 23 | 2007 | 16.5 | Ni | 0.46 | FALSE | 0.79 | FALSE | 0.32 | FALSE | NA | NA | NA | NA |
| Al-Jasra | 23 | 2007 | 7.93 | Pb | 0.21 | FALSE | 0.17 | FALSE | 0.04 | FALSE | 0.26 | FALSE | 0.07 | FALSE |
| Al-Jasra | 23 | 2007 | 21.1 | Zn | 0.17 | FALSE | 0.14 | FALSE | 0.05 | FALSE | 0.17 | FALSE | 0.08 | FALSE |
| Shtaya | 24 | 2020 | 0.075 | Cd | 0.24 | FALSE | 0.06 | FALSE | 0.01 | FALSE | 0.11 | FALSE | 0.02 | FALSE |
| Shtaya | 24 | 2020 | 24.3 | Cr | 0.3 | FALSE | 0.3 | FALSE | 0.07 | FALSE | 0.46 | FALSE | 0.15 | FALSE |
| Shtaya | 24 | 2020 | 85.3 | Cu | 3.16 | TRUE | 2.51 | TRUE | 0.32 | FALSE | 4.56 | TRUE | 0.79 | FALSE |
| Shtaya | 24 | 2020 | 8.1 | Ni | 0.22 | FALSE | 0.39 | FALSE | 0.16 | FALSE | NA | NA | NA | NA |
| Shtaya | 24 | 2020 | 2.36 | Pb | 0.06 | FALSE | 0.05 | FALSE | 0.01 | FALSE | 0.08 | FALSE | 0.02 | FALSE |
| Shtaya | 24 | 2020 | 14.8 | Zn | 0.12 | FALSE | 0.1 | FALSE | 0.04 | FALSE | 0.12 | FALSE | 0.05 | FALSE |
| Shtaya | 24 | 2019 | 0.069 | Cd | 0.22 | FALSE | 0.06 | FALSE | 0.01 | FALSE | 0.1 | FALSE | 0.02 | FALSE |
| Shtaya | 24 | 2019 | 31.6 | Cr | 0.39 | FALSE | 0.39 | FALSE | 0.09 | FALSE | 0.6 | FALSE | 0.2 | FALSE |
| Shtaya | 24 | 2019 | 280 | Cu | 10.36 | TRUE | 8.23 | TRUE | 1.04 | TRUE | 14.96 | TRUE | 2.59 | TRUE |
| Shtaya | 24 | 2019 | 11.6 | Ni | 0.32 | FALSE | 0.56 | FALSE | 0.22 | FALSE | NA | NA | NA | NA |
| Shtaya | 24 | 2019 | 7.51 | Pb | 0.2 | FALSE | 0.16 | FALSE | 0.03 | FALSE | 0.25 | FALSE | 0.07 | FALSE |
| Shtaya | 24 | 2019 | 17.4 | Zn | 0.14 | FALSE | 0.12 | FALSE | 0.04 | FALSE | 0.14 | FALSE | 0.06 | FALSE |
| Shtaya | 24 | 2018 | 0.073 | Cd | 0.24 | FALSE | 0.06 | FALSE | 0.01 | FALSE | 0.1 | FALSE | 0.02 | FALSE |
| Shtaya | 24 | 2018 | 70.2 | Cr | 0.87 | FALSE | 0.87 | FALSE | 0.19 | FALSE | 1.34 | TRUE | 0.44 | FALSE |
| Shtaya | 24 | 2018 | 103 | Cu | 3.81 | TRUE | 3.02 | TRUE | 0.38 | FALSE | 5.5 | TRUE | 0.95 | FALSE |
| Shtaya | 24 | 2018 | 12.4 | Ni | 0.35 | FALSE | 0.59 | FALSE | 0.24 | FALSE | NA | NA | NA | NA |
| Shtaya | 24 | 2018 | 3.64 | Pb | 0.1 | FALSE | 0.08 | FALSE | 0.02 | FALSE | 0.12 | FALSE | 0.03 | FALSE |
| Shtaya | 24 | 2018 | 18.2 | Zn | 0.15 | FALSE | 0.12 | FALSE | 0.04 | FALSE | 0.15 | FALSE | 0.07 | FALSE |
| Shtaya | 24 | 2017 | 0.066 | Cd | 0.21 | FALSE | 0.06 | FALSE | 0.01 | FALSE | 0.09 | FALSE | 0.02 | FALSE |
| Shtaya | 24 | 2017 | 76.8 | Cr | 0.95 | FALSE | 0.95 | FALSE | 0.21 | FALSE | 1.47 | TRUE | 0.48 | FALSE |
| Shtaya | 24 | 2017 | 46 | Cu | 1.7 | TRUE | 1.35 | TRUE | 0.17 | FALSE | 2.46 | TRUE | 0.43 | FALSE |
| Shtaya | 24 | 2017 | 5.8 | Ni | 0.16 | FALSE | 0.28 | FALSE | 0.11 | FALSE | NA | NA | NA | NA |
| Shtaya | 24 | 2017 | 8.21 | Pb | 0.22 | FALSE | 0.18 | FALSE | 0.04 | FALSE | 0.27 | FALSE | 0.07 | FALSE |
| Shtaya | 24 | 2017 | 22 | Zn | 0.18 | FALSE | 0.15 | FALSE | 0.05 | FALSE | 0.18 | FALSE | 0.08 | FALSE |
| Shtaya | 24 | 2016 | 0.065 | Cd | 0.21 | FALSE | 0.05 | FALSE | 0.01 | FALSE | 0.09 | FALSE | 0.02 | FALSE |
| Shtaya | 24 | 2016 | 68.3 | Cr | 0.84 | FALSE | 0.84 | FALSE | 0.18 | FALSE | 1.31 | TRUE | 0.43 | FALSE |
| Shtaya | 24 | 2016 | 7.9 | Cu | 0.29 | FALSE | 0.23 | FALSE | 0.03 | FALSE | 0.42 | FALSE | 0.07 | FALSE |
| Shtaya | 24 | 2016 | 17.1 | Ni | 0.47 | FALSE | 0.82 | FALSE | 0.33 | FALSE | NA | NA | NA | NA |
| Shtaya | 24 | 2016 | 2.27 | Pb | 0.06 | FALSE | 0.05 | FALSE | 0.01 | FALSE | 0.08 | FALSE | 0.02 | FALSE |
| Shtaya | 24 | 2016 | 11.8 | Zn | 0.1 | FALSE | 0.08 | FALSE | 0.03 | FALSE | 0.09 | FALSE | 0.04 | FALSE |
| Shtaya | 24 | 2015 | 0.046 | Cd | 0.15 | FALSE | 0.04 | FALSE | 0 | FALSE | 0.07 | FALSE | 0.01 | FALSE |
| Shtaya | 24 | 2015 | 89.9 | Cr | 1.11 | TRUE | 1.11 | TRUE | 0.24 | FALSE | 1.72 | TRUE | 0.56 | FALSE |
| Shtaya | 24 | 2015 | 8 | Cu | 0.3 | FALSE | 0.24 | FALSE | 0.03 | FALSE | 0.43 | FALSE | 0.07 | FALSE |
| Shtaya | 24 | 2015 | 6.6 | Ni | 0.18 | FALSE | 0.32 | FALSE | 0.13 | FALSE | NA | NA | NA | NA |
| Shtaya | 24 | 2015 | 9.1 | Pb | 0.24 | FALSE | 0.19 | FALSE | 0.04 | FALSE | 0.3 | FALSE | 0.08 | FALSE |
| Shtaya | 24 | 2015 | 4.8 | Zn | 0.04 | FALSE | 0.03 | FALSE | 0.01 | FALSE | 0.04 | FALSE | 0.02 | FALSE |
| Bulthama | 25 | 2015 | 0.098 | Cd | 0.32 | FALSE | 0.08 | FALSE | 0.01 | FALSE | 0.14 | FALSE | 0.02 | FALSE |
| Bulthama | 25 | 2015 | 161 | Cr | 1.98 | TRUE | 1.98 | TRUE | 0.43 | FALSE | 3.07 | TRUE | 1 | TRUE |
| Bulthama | 25 | 2015 | 25.9 | Cu | 0.96 | FALSE | 0.76 | FALSE | 0.1 | FALSE | 1.38 | TRUE | 0.24 | FALSE |
| Bulthama | 25 | 2015 | 26.7 | Ni | 0.74 | FALSE | 1.28 | TRUE | 0.52 | FALSE | NA | NA | NA | NA |
| Bulthama | 25 | 2015 | 19.7 | Pb | 0.52 | FALSE | 0.42 | FALSE | 0.09 | FALSE | 0.65 | FALSE | 0.18 | FALSE |
| Bulthama | 25 | 2015 | 7.7 | Zn | 0.06 | FALSE | 0.05 | FALSE | 0.02 | FALSE | 0.06 | FALSE | 0.03 | FALSE |
| Bulthama | 25 | 2014 | 0.138 | Cd | 0.44 | FALSE | 0.11 | FALSE | 0.01 | FALSE | 0.2 | FALSE | 0.03 | FALSE |
| Bulthama | 25 | 2014 | 50.8 | Cr | 0.63 | FALSE | 0.63 | FALSE | 0.14 | FALSE | 0.97 | FALSE | 0.32 | FALSE |
| Bulthama | 25 | 2014 | 16 | Cu | 0.59 | FALSE | 0.47 | FALSE | 0.06 | FALSE | 0.85 | FALSE | 0.15 | FALSE |
| Bulthama | 25 | 2014 | 41.4 | Ni | 1.15 | TRUE | 1.98 | TRUE | 0.8 | FALSE | NA | NA | NA | NA |
| Bulthama | 25 | 2014 | 2.03 | Pb | 0.05 | FALSE | 0.04 | FALSE | 0.01 | FALSE | 0.07 | FALSE | 0.02 | FALSE |
| Bulthama | 25 | 2014 | 18.6 | Zn | 0.15 | FALSE | 0.12 | FALSE | 0.05 | FALSE | 0.15 | FALSE | 0.07 | FALSE |
| West Jarim | 26 | 2016 | 0.052 | Cd | 0.17 | FALSE | 0.04 | FALSE | 0.01 | FALSE | 0.07 | FALSE | 0.01 | FALSE |
| West Jarim | 26 | 2016 | 27.4 | Cr | 0.34 | FALSE | 0.34 | FALSE | 0.07 | FALSE | 0.52 | FALSE | 0.17 | FALSE |
| West Jarim | 26 | 2016 | 6.4 | Cu | 0.24 | FALSE | 0.19 | FALSE | 0.02 | FALSE | 0.34 | FALSE | 0.06 | FALSE |
| West Jarim | 26 | 2016 | 7.7 | Ni | 0.21 | FALSE | 0.37 | FALSE | 0.15 | FALSE | NA | NA | NA | NA |
| West Jarim | 26 | 2016 | 1.12 | Pb | 0.03 | FALSE | 0.02 | FALSE | 0.01 | FALSE | 0.04 | FALSE | 0.01 | FALSE |
| West Jarim | 26 | 2016 | 14.5 | Zn | 0.12 | FALSE | 0.1 | FALSE | 0.04 | FALSE | 0.12 | FALSE | 0.05 | FALSE |
| West Jarim | 26 | 2015 | 0.07 | Cd | 0.23 | FALSE | 0.06 | FALSE | 0.01 | FALSE | 0.1 | FALSE | 0.02 | FALSE |
| West Jarim | 26 | 2015 | 41.2 | Cr | 0.51 | FALSE | 0.51 | FALSE | 0.11 | FALSE | 0.79 | FALSE | 0.26 | FALSE |
| West Jarim | 26 | 2015 | 6.6 | Cu | 0.24 | FALSE | 0.19 | FALSE | 0.02 | FALSE | 0.35 | FALSE | 0.06 | FALSE |
| West Jarim | 26 | 2015 | 17.9 | Ni | 0.5 | FALSE | 0.86 | FALSE | 0.35 | FALSE | NA | NA | NA | NA |
| West Jarim | 26 | 2015 | 0.67 | Pb | 0.02 | FALSE | 0.01 | FALSE | 0 | FALSE | 0.02 | FALSE | 0.01 | FALSE |
| West Jarim | 26 | 2015 | 14.4 | Zn | 0.12 | FALSE | 0.1 | FALSE | 0.04 | FALSE | 0.12 | FALSE | 0.05 | FALSE |
| West Jarim | 26 | 2014 | 0.104 | Cd | 0.33 | FALSE | 0.09 | FALSE | 0.01 | FALSE | 0.15 | FALSE | 0.02 | FALSE |
| West Jarim | 26 | 2014 | 34.7 | Cr | 0.43 | FALSE | 0.43 | FALSE | 0.09 | FALSE | 0.66 | FALSE | 0.22 | FALSE |
| West Jarim | 26 | 2014 | 6.4 | Cu | 0.24 | FALSE | 0.19 | FALSE | 0.02 | FALSE | 0.34 | FALSE | 0.06 | FALSE |
| West Jarim | 26 | 2014 | 18.9 | Ni | 0.52 | FALSE | 0.9 | FALSE | 0.37 | FALSE | NA | NA | NA | NA |
| West Jarim | 26 | 2014 | 1.34 | Pb | 0.04 | FALSE | 0.03 | FALSE | 0.01 | FALSE | 0.04 | FALSE | 0.01 | FALSE |
| West Jarim | 26 | 2014 | 19.1 | Zn | 0.16 | FALSE | 0.13 | FALSE | 0.05 | FALSE | 0.15 | FALSE | 0.07 | FALSE |
| Jetty 2 | 27 | 2015 | 0.143 | Cd | 0.46 | FALSE | 0.12 | FALSE | 0.01 | FALSE | 0.2 | FALSE | 0.03 | FALSE |
| Jetty 2 | 27 | 2015 | 140 | Cr | 1.72 | TRUE | 1.72 | TRUE | 0.38 | FALSE | 2.67 | TRUE | 0.87 | FALSE |
| Jetty 2 | 27 | 2015 | 54 | Cu | 2 | TRUE | 1.59 | TRUE | 0.2 | FALSE | 2.89 | TRUE | 0.5 | FALSE |
| Jetty 2 | 27 | 2015 | 17.3 | Ni | 0.48 | FALSE | 0.83 | FALSE | 0.34 | FALSE | NA | NA | NA | NA |
| Jetty 2 | 27 | 2015 | 4 | Pb | 0.11 | FALSE | 0.09 | FALSE | 0.02 | FALSE | 0.13 | FALSE | 0.04 | FALSE |
| Jetty 2 | 27 | 2015 | 78.9 | Zn | 0.65 | FALSE | 0.53 | FALSE | 0.19 | FALSE | 0.64 | FALSE | 0.29 | FALSE |
| Jetty 3 | 28 | 2020 | 0.07 | Cd | 0.23 | FALSE | 0.06 | FALSE | 0.01 | FALSE | 0.1 | FALSE | 0.02 | FALSE |
| Jetty 3 | 28 | 2020 | 57.6 | Cr | 0.71 | FALSE | 0.71 | FALSE | 0.16 | FALSE | 1.1 | TRUE | 0.36 | FALSE |
| Jetty 3 | 28 | 2020 | 27.3 | Cu | 1.01 | TRUE | 0.8 | FALSE | 0.1 | FALSE | 1.46 | TRUE | 0.25 | FALSE |
| Jetty 3 | 28 | 2020 | 17.1 | Ni | 0.48 | FALSE | 0.82 | FALSE | 0.33 | FALSE | NA | NA | NA | NA |
| Jetty 3 | 28 | 2020 | 1.84 | Pb | 0.05 | FALSE | 0.04 | FALSE | 0.01 | FALSE | 0.06 | FALSE | 0.02 | FALSE |
| Jetty 3 | 28 | 2020 | 49.7 | Zn | 0.41 | FALSE | 0.33 | FALSE | 0.12 | FALSE | 0.4 | FALSE | 0.18 | FALSE |
| Jetty 3 | 28 | 2019 | 0.135 | Cd | 0.44 | FALSE | 0.11 | FALSE | 0.01 | FALSE | 0.19 | FALSE | 0.03 | FALSE |
| Jetty 3 | 28 | 2019 | 67.4 | Cr | 0.83 | FALSE | 0.83 | FALSE | 0.18 | FALSE | 1.29 | TRUE | 0.42 | FALSE |
| Jetty 3 | 28 | 2019 | 21.2 | Cu | 0.79 | FALSE | 0.62 | FALSE | 0.08 | FALSE | 1.13 | TRUE | 0.2 | FALSE |
| Jetty 3 | 28 | 2019 | 18 | Ni | 0.5 | FALSE | 0.86 | FALSE | 0.35 | FALSE | NA | NA | NA | NA |
| Jetty 3 | 28 | 2019 | 5.75 | Pb | 0.15 | FALSE | 0.12 | FALSE | 0.03 | FALSE | 0.19 | FALSE | 0.05 | FALSE |
| Jetty 3 | 28 | 2019 | 53.8 | Zn | 0.44 | FALSE | 0.36 | FALSE | 0.13 | FALSE | 0.43 | FALSE | 0.2 | FALSE |
| Jetty 3 | 28 | 2018 | 0.156 | Cd | 0.5 | FALSE | 0.13 | FALSE | 0.02 | FALSE | 0.22 | FALSE | 0.04 | FALSE |
| Jetty 3 | 28 | 2018 | 71.7 | Cr | 0.89 | FALSE | 0.89 | FALSE | 0.19 | FALSE | 1.37 | TRUE | 0.45 | FALSE |
| Jetty 3 | 28 | 2018 | 22.8 | Cu | 0.84 | FALSE | 0.67 | FALSE | 0.08 | FALSE | 1.22 | TRUE | 0.21 | FALSE |
| Jetty 3 | 28 | 2018 | 17.1 | Ni | 0.47 | FALSE | 0.82 | FALSE | 0.33 | FALSE | NA | NA | NA | NA |
| Jetty 3 | 28 | 2018 | 2.09 | Pb | 0.06 | FALSE | 0.04 | FALSE | 0.01 | FALSE | 0.07 | FALSE | 0.02 | FALSE |
| Jetty 3 | 28 | 2018 | 52.5 | Zn | 0.43 | FALSE | 0.35 | FALSE | 0.13 | FALSE | 0.42 | FALSE | 0.19 | FALSE |
| Jetty 3 | 28 | 2017 | 0.128 | Cd | 0.41 | FALSE | 0.11 | FALSE | 0.01 | FALSE | 0.18 | FALSE | 0.03 | FALSE |
| Jetty 3 | 28 | 2017 | 78.4 | Cr | 0.97 | FALSE | 0.97 | FALSE | 0.21 | FALSE | 1.5 | TRUE | 0.49 | FALSE |
| Jetty 3 | 28 | 2017 | 27.9 | Cu | 1.03 | TRUE | 0.82 | FALSE | 0.1 | FALSE | 1.49 | TRUE | 0.26 | FALSE |
| Jetty 3 | 28 | 2017 | 25.2 | Ni | 0.7 | FALSE | 1.21 | TRUE | 0.49 | FALSE | NA | NA | NA | NA |
| Jetty 3 | 28 | 2017 | 2.31 | Pb | 0.06 | FALSE | 0.05 | FALSE | 0.01 | FALSE | 0.08 | FALSE | 0.02 | FALSE |
| Jetty 3 | 28 | 2017 | 43.1 | Zn | 0.35 | FALSE | 0.29 | FALSE | 0.11 | FALSE | 0.35 | FALSE | 0.16 | FALSE |
| Jetty 3 | 28 | 2016 | 0.122 | Cd | 0.39 | FALSE | 0.1 | FALSE | 0.01 | FALSE | 0.17 | FALSE | 0.03 | FALSE |
| Jetty 3 | 28 | 2016 | 81.9 | Cr | 1.01 | TRUE | 1.01 | TRUE | 0.22 | FALSE | 1.57 | TRUE | 0.51 | FALSE |
| Jetty 3 | 28 | 2016 | 52.2 | Cu | 1.93 | TRUE | 1.54 | TRUE | 0.19 | FALSE | 2.79 | TRUE | 0.48 | FALSE |
| Jetty 3 | 28 | 2016 | 18.4 | Ni | 0.51 | FALSE | 0.88 | FALSE | 0.36 | FALSE | NA | NA | NA | NA |
| Jetty 3 | 28 | 2016 | 1.79 | Pb | 0.05 | FALSE | 0.04 | FALSE | 0.01 | FALSE | 0.06 | FALSE | 0.02 | FALSE |
| Jetty 3 | 28 | 2016 | 35.9 | Zn | 0.29 | FALSE | 0.24 | FALSE | 0.09 | FALSE | 0.29 | FALSE | 0.13 | FALSE |
| Wharf Area | 29 | 2020 | 0.082 | Cd | 0.26 | FALSE | 0.07 | FALSE | 0.01 | FALSE | 0.12 | FALSE | 0.02 | FALSE |
| Wharf Area | 29 | 2020 | 41.9 | Cr | 0.52 | FALSE | 0.52 | FALSE | 0.11 | FALSE | 0.8 | FALSE | 0.26 | FALSE |
| Wharf Area | 29 | 2020 | 24.7 | Cu | 0.91 | FALSE | 0.73 | FALSE | 0.09 | FALSE | 1.32 | TRUE | 0.23 | FALSE |
| Wharf Area | 29 | 2020 | 12.9 | Ni | 0.36 | FALSE | 0.62 | FALSE | 0.25 | FALSE | NA | NA | NA | NA |
| Wharf Area | 29 | 2020 | 2.37 | Pb | 0.06 | FALSE | 0.05 | FALSE | 0.01 | FALSE | 0.08 | FALSE | 0.02 | FALSE |
| Wharf Area | 29 | 2020 | 29.5 | Zn | 0.24 | FALSE | 0.2 | FALSE | 0.07 | FALSE | 0.24 | FALSE | 0.11 | FALSE |
| Wharf Area | 29 | 2019 | 0.059 | Cd | 0.19 | FALSE | 0.05 | FALSE | 0.01 | FALSE | 0.08 | FALSE | 0.01 | FALSE |
| Wharf Area | 29 | 2019 | 115 | Cr | 1.42 | TRUE | 1.42 | TRUE | 0.31 | FALSE | 2.2 | TRUE | 0.72 | FALSE |
| Wharf Area | 29 | 2019 | 25.2 | Cu | 0.93 | FALSE | 0.74 | FALSE | 0.09 | FALSE | 1.35 | TRUE | 0.23 | FALSE |
| Wharf Area | 29 | 2019 | 41.5 | Ni | 1.15 | TRUE | 1.99 | TRUE | 0.8 | FALSE | NA | NA | NA | NA |
| Wharf Area | 29 | 2019 | 6.28 | Pb | 0.17 | FALSE | 0.13 | FALSE | 0.03 | FALSE | 0.21 | FALSE | 0.06 | FALSE |
| Wharf Area | 29 | 2019 | 27.6 | Zn | 0.23 | FALSE | 0.18 | FALSE | 0.07 | FALSE | 0.22 | FALSE | 0.1 | FALSE |
| Wharf Area | 29 | 2018 | 0.075 | Cd | 0.24 | FALSE | 0.06 | FALSE | 0.01 | FALSE | 0.11 | FALSE | 0.02 | FALSE |
| Wharf Area | 29 | 2018 | 47.4 | Cr | 0.59 | FALSE | 0.59 | FALSE | 0.13 | FALSE | 0.91 | FALSE | 0.3 | FALSE |
| Wharf Area | 29 | 2018 | 38.5 | Cu | 1.43 | TRUE | 1.13 | TRUE | 0.14 | FALSE | 2.06 | TRUE | 0.36 | FALSE |
| Wharf Area | 29 | 2018 | 13.3 | Ni | 0.37 | FALSE | 0.64 | FALSE | 0.26 | FALSE | NA | NA | NA | NA |
| Wharf Area | 29 | 2018 | 1.85 | Pb | 0.05 | FALSE | 0.04 | FALSE | 0.01 | FALSE | 0.06 | FALSE | 0.02 | FALSE |
| Wharf Area | 29 | 2018 | 29.4 | Zn | 0.24 | FALSE | 0.2 | FALSE | 0.07 | FALSE | 0.24 | FALSE | 0.11 | FALSE |
| Wharf Area | 29 | 2017 | 0.076 | Cd | 0.25 | FALSE | 0.06 | FALSE | 0.01 | FALSE | 0.11 | FALSE | 0.02 | FALSE |
| Wharf Area | 29 | 2017 | 48.8 | Cr | 0.6 | FALSE | 0.6 | FALSE | 0.13 | FALSE | 0.93 | FALSE | 0.3 | FALSE |
| Wharf Area | 29 | 2017 | 22.9 | Cu | 0.85 | FALSE | 0.67 | FALSE | 0.08 | FALSE | 1.23 | TRUE | 0.21 | FALSE |
| Wharf Area | 29 | 2017 | 23.5 | Ni | 0.65 | FALSE | 1.12 | TRUE | 0.46 | FALSE | NA | NA | NA | NA |
| Wharf Area | 29 | 2017 | 2.4 | Pb | 0.06 | FALSE | 0.05 | FALSE | 0.01 | FALSE | 0.08 | FALSE | 0.02 | FALSE |
| Wharf Area | 29 | 2017 | 31.6 | Zn | 0.26 | FALSE | 0.21 | FALSE | 0.08 | FALSE | 0.25 | FALSE | 0.12 | FALSE |

*MEC is the Measured Environmental Concentrations in mg kg^-1^; BAC is the Background Assessment Concentration; ERL is the Effects Range Low; ERM is the Effects Range Median; ISQG is the Canadian Interim Sediment Quality Guidelines; PEL is the Probable Effects Levels.

Table S6. Measured Environmental Concentrations (MEC) of Al, Fe and Mn

| Station | Site No. | | Year | MEC (mg kg^-1^) | Determinant |
| --- | --- | --- | --- | --- | --- |
| Noon | | 1 | 2016 | 5600 | Al |
| Noon | | 1 | 2016 | 3100 | Fe |
| Noon | | 1 | 2016 | 78.9 | Mn |
| Noon | | 1 | 2015 | 8990 | Al |
| Noon | | 1 | 2015 | 9530 | Fe |
| Noon | | 1 | 2015 | 217 | Mn |
| Noon | | 1 | 2014 | 5850 | Al |
| Noon | | 1 | 2014 | 3540 | Fe |
| Noon | | 1 | 2014 | 51.7 | Mn |
| Noon | | 1 | 2013 | 6920 | Al |
| Noon | | 1 | 2013 | 5430 | Fe |
| Noon | | 1 | 2013 | 109 | Mn |
| Noon | | 1 | 2012 | 9760 | Al |
| Noon | | 1 | 2012 | 6340 | Fe |
| Noon | | 1 | 2012 | 103 | Mn |
| Noon | | 1 | 2011 | 12700 | Al |
| Noon | | 1 | 2011 | 7200 | Fe |
| Noon | | 1 | 2011 | 122 | Mn |
| Noon | | 1 | 2010 | 10500 | Al |
| Noon | | 1 | 2010 | 2530 | Fe |
| Noon | | 1 | 2010 | 108 | Mn |
| Noon | | 1 | 2009 | 14300 | Al |
| Noon | | 1 | 2009 | 2980 | Fe |
| Noon | | 1 | 2009 | 54.6 | Mn |
| Noon | | 1 | 2008 | 115 | Mn |
| Noon | | 1 | 2007 | 109 | Mn |
| Mashtan | | 2 | 2020 | 4260 | Al |
| Mashtan | | 2 | 2020 | 3070 | Fe |
| Mashtan | | 2 | 2020 | 61.4 | Mn |
| Mashtan | | 2 | 2019 | 5010 | Al |
| Mashtan | | 2 | 2019 | 3220 | Fe |
| Mashtan | | 2 | 2019 | 71.9 | Mn |
| Mashtan | | 2 | 2018 | 10000 | Al |
| Mashtan | | 2 | 2018 | 3680 | Fe |
| Mashtan | | 2 | 2018 | 135 | Mn |
| Mashtan | | 2 | 2017 | 2600 | Al |
| Mashtan | | 2 | 2017 | 2030 | Fe |
| Mashtan | | 2 | 2017 | 52.3 | Mn |
| Mashtan | | 2 | 2016 | 9230 | Al |
| Mashtan | | 2 | 2016 | 4990 | Fe |
| Mashtan | | 2 | 2016 | 80.3 | Mn |
| Mashtan | | 2 | 2015 | 5760 | Al |
| Mashtan | | 2 | 2015 | 4360 | Fe |
| Mashtan | | 2 | 2015 | 70.6 | Mn |
| Mashtan | | 2 | 2014 | 5410 | Al |
| Mashtan | | 2 | 2014 | 2550 | Fe |
| Mashtan | | 2 | 2014 | 71.7 | Mn |
| Mashtan | | 2 | 2013 | 8750 | Al |
| Mashtan | | 2 | 2013 | 4190 | Fe |
| Mashtan | | 2 | 2013 | 167 | Mn |
| Mashtan | | 2 | 2012 | 6040 | Al |
| Mashtan | | 2 | 2012 | 3230 | Fe |
| Mashtan | | 2 | 2012 | 62.1 | Mn |
| Mashtan | | 2 | 2011 | 4540 | Al |
| Mashtan | | 2 | 2011 | 5110 | Fe |
| Mashtan | | 2 | 2011 | 71.2 | Mn |
| Mashtan | | 2 | 2010 | 9590 | Al |
| Mashtan | | 2 | 2010 | 2540 | Fe |
| Mashtan | | 2 | 2010 | 79.8 | Mn |
| Mashtan | | 2 | 2009 | 20300 | Al |
| Mashtan | | 2 | 2009 | 4730 | Fe |
| Mashtan | | 2 | 2009 | 128 | Mn |
| Mashtan | | 2 | 2008 | 96.2 | Mn |
| Mashtan | | 2 | 2007 | 154 | Mn |
| Jabari | | 3 | 2016 | 6090 | Al |
| Jabari | | 3 | 2016 | 5690 | Fe |
| Jabari | | 3 | 2016 | 91.2 | Mn |
| Jabari | | 3 | 2015 | 6800 | Al |
| Jabari | | 3 | 2015 | 6350 | Fe |
| Jabari | | 3 | 2015 | 101 | Mn |
| Jabari | | 3 | 2014 | 7530 | Al |
| Jabari | | 3 | 2014 | 4650 | Fe |
| Jabari | | 3 | 2014 | 95.9 | Mn |
| Jabari | | 3 | 2013 | 5900 | Al |
| Jabari | | 3 | 2013 | 4920 | Fe |
| Jabari | | 3 | 2013 | 87.4 | Mn |
| Jabari | | 3 | 2012 | 7950 | Al |
| Jabari | | 3 | 2012 | 4570 | Fe |
| Jabari | | 3 | 2012 | 96.3 | Mn |
| Jabari | | 3 | 2011 | 11900 | Al |
| Jabari | | 3 | 2011 | 5090 | Fe |
| Jabari | | 3 | 2011 | 82.5 | Mn |
| Jabari | | 3 | 2010 | 8070 | Al |
| Jabari | | 3 | 2010 | 4870 | Fe |
| Jabari | | 3 | 2010 | 78.8 | Mn |
| Jabari | | 3 | 2009 | 14000 | Al |
| Jabari | | 3 | 2009 | 4280 | Fe |
| Jabari | | 3 | 2009 | 71.7 | Mn |
| Jabari | | 3 | 2008 | 84.2 | Mn |
| Jabari | | 3 | 2007 | 81.8 | Mn |
| Tugailib | | 4 | 2015 | 3560 | Al |
| Tugailib | | 4 | 2015 | 3420 | Fe |
| Tugailib | | 4 | 2015 | 54.1 | Mn |
| Tugailib | | 4 | 2014 | 4260 | Al |
| Tugailib | | 4 | 2014 | 2510 | Fe |
| Tugailib | | 4 | 2014 | 98.3 | Mn |
| Tugailib | | 4 | 2013 | 5680 | Al |
| Tugailib | | 4 | 2013 | 4240 | Fe |
| Tugailib | | 4 | 2013 | 170 | Mn |
| Tugailib | | 4 | 2012 | 5140 | Al |
| Tugailib | | 4 | 2012 | 3500 | Fe |
| Tugailib | | 4 | 2012 | 57.5 | Mn |
| Tugailib | | 4 | 2011 | 7370 | Al |
| Tugailib | | 4 | 2011 | 4470 | Fe |
| Tugailib | | 4 | 2011 | 51.6 | Mn |
| Tugailib | | 4 | 2010 | 7050 | Al |
| Tugailib | | 4 | 2010 | 4140 | Fe |
| Tugailib | | 4 | 2010 | 95.2 | Mn |
| Tugailib | | 4 | 2009 | 10900 | Al |
| Tugailib | | 4 | 2009 | 3460 | Fe |
| Tugailib | | 4 | 2009 | 49 | Mn |
| Tugailib | | 4 | 2008 | 93.7 | Mn |
| Tugailib | | 4 | 2007 | 34.4 | Mn |
| Ghumais | | 5 | 2020 | 4620 | Al |
| Ghumais | | 5 | 2020 | 5110 | Fe |
| Ghumais | | 5 | 2020 | 110 | Mn |
| Ghumais | | 5 | 2019 | 6700 | Al |
| Ghumais | | 5 | 2019 | 5970 | Fe |
| Ghumais | | 5 | 2019 | 116 | Mn |
| Ghumais | | 5 | 2018 | 7110 | Al |
| Ghumais | | 5 | 2018 | 5320 | Fe |
| Ghumais | | 5 | 2018 | 103 | Mn |
| Ghumais | | 5 | 2017 | 4560 | Al |
| Ghumais | | 5 | 2017 | 6210 | Fe |
| Ghumais | | 5 | 2017 | 120 | Mn |
| Ghumais | | 5 | 2016 | 7690 | Al |
| Ghumais | | 5 | 2016 | 6390 | Fe |
| Ghumais | | 5 | 2016 | 91.5 | Mn |
| Ghumais | | 5 | 2015 | 6800 | Al |
| Ghumais | | 5 | 2015 | 8100 | Fe |
| Ghumais | | 5 | 2015 | 114 | Mn |
| Ghumais | | 5 | 2014 | 7250 | Al |
| Ghumais | | 5 | 2014 | 5350 | Fe |
| Ghumais | | 5 | 2014 | 52.2 | Mn |
| Ghumais | | 5 | 2013 | 6620 | Al |
| Ghumais | | 5 | 2013 | 6070 | Fe |
| Ghumais | | 5 | 2013 | 106 | Mn |
| Ghumais | | 5 | 2012 | 8370 | Al |
| Ghumais | | 5 | 2012 | 5620 | Fe |
| Ghumais | | 5 | 2012 | 116 | Mn |
| Ghumais | | 5 | 2011 | 9350 | Al |
| Ghumais | | 5 | 2011 | 7000 | Fe |
| Ghumais | | 5 | 2011 | 93.6 | Mn |
| Ghumais | | 5 | 2010 | 6220 | Al |
| Ghumais | | 5 | 2010 | 4930 | Fe |
| Ghumais | | 5 | 2010 | 80.2 | Mn |
| Ghumais | | 5 | 2009 | 13300 | Al |
| Ghumais | | 5 | 2009 | 8030 | Fe |
| Ghumais | | 5 | 2009 | 110 | Mn |
| Ghumais | | 5 | 2008 | 105 | Mn |
| Ghumais | | 5 | 2007 | 110 | Mn |
| Askar | | 6 | 2020 | 5420 | Al |
| Askar | | 6 | 2020 | 4040 | Fe |
| Askar | | 6 | 2020 | 73.6 | Mn |
| Askar | | 6 | 2019 | 6400 | Al |
| Askar | | 6 | 2019 | 4310 | Fe |
| Askar | | 6 | 2019 | 75.5 | Mn |
| Askar | | 6 | 2018 | 6370 | Al |
| Askar | | 6 | 2018 | 3900 | Fe |
| Askar | | 6 | 2018 | 73.2 | Mn |
| Askar | | 6 | 2017 | 3950 | Al |
| Askar | | 6 | 2017 | 3770 | Fe |
| Askar | | 6 | 2017 | 78.3 | Mn |
| Askar | | 6 | 2016 | 5660 | Al |
| Askar | | 6 | 2016 | 5290 | Fe |
| Askar | | 6 | 2016 | 72.3 | Mn |
| Askar | | 6 | 2015 | 6550 | Al |
| Askar | | 6 | 2015 | 6200 | Fe |
| Askar | | 6 | 2015 | 87.9 | Mn |
| Askar | | 6 | 2014 | 6150 | Al |
| Askar | | 6 | 2014 | 4070 | Fe |
| Askar | | 6 | 2014 | 80 | Mn |
| Askar | | 6 | 2013 | 5180 | Al |
| Askar | | 6 | 2013 | 4680 | Fe |
| Askar | | 6 | 2013 | 74.2 | Mn |
| Askar | | 6 | 2012 | 6770 | Al |
| Askar | | 6 | 2012 | 6400 | Fe |
| Askar | | 6 | 2012 | 102 | Mn |
| Askar | | 6 | 2011 | 7260 | Al |
| Askar | | 6 | 2011 | 3880 | Fe |
| Askar | | 6 | 2011 | 73.2 | Mn |
| Askar | | 6 | 2010 | 8300 | Al |
| Askar | | 6 | 2010 | 4010 | Fe |
| Askar | | 6 | 2010 | 91.5 | Mn |
| Askar | | 6 | 2009 | 12300 | Al |
| Askar | | 6 | 2009 | 3750 | Fe |
| Askar | | 6 | 2009 | 62.3 | Mn |
| Askar | | 6 | 2008 | 59.4 | Mn |
| Askar | | 6 | 2007 | 66.2 | Mn |
| Msoor | | 7 | 2016 | 4750 | Al |
| Msoor | | 7 | 2016 | 4160 | Fe |
| Msoor | | 7 | 2016 | 46.2 | Mn |
| Msoor | | 7 | 2015 | 4680 | Al |
| Msoor | | 7 | 2015 | 6270 | Fe |
| Msoor | | 7 | 2015 | 68.2 | Mn |
| Msoor | | 7 | 2014 | 4750 | Al |
| Msoor | | 7 | 2014 | 3890 | Fe |
| Msoor | | 7 | 2014 | 35.7 | Mn |
| Msoor | | 7 | 2013 | 3850 | Al |
| Msoor | | 7 | 2013 | 4640 | Fe |
| Msoor | | 7 | 2013 | 50.8 | Mn |
| Msoor | | 7 | 2012 | 4860 | Al |
| Msoor | | 7 | 2012 | 3950 | Fe |
| Msoor | | 7 | 2012 | 43.7 | Mn |
| Msoor | | 7 | 2011 | 2230 | Al |
| Msoor | | 7 | 2011 | 3450 | Fe |
| Msoor | | 7 | 2011 | 44.3 | Mn |
| Msoor | | 7 | 2010 | 4810 | Al |
| Msoor | | 7 | 2010 | 4080 | Fe |
| Msoor | | 7 | 2010 | 53.8 | Mn |
| Msoor | | 7 | 2009 | 12800 | Al |
| Msoor | | 7 | 2009 | 4350 | Fe |
| Msoor | | 7 | 2009 | 47.8 | Mn |
| Msoor | | 7 | 2008 | 48.6 | Mn |
| Msoor | | 7 | 2007 | 56.2 | Mn |
| Refinery Area | | 8 | 2020 | 6270 | Al |
| Refinery Area | | 8 | 2020 | 5750 | Fe |
| Refinery Area | | 8 | 2020 | 97.4 | Mn |
| Refinery Area | | 8 | 2019 | 6810 | Al |
| Refinery Area | | 8 | 2019 | 5290 | Fe |
| Refinery Area | | 8 | 2019 | 90.2 | Mn |
| Refinery Area | | 8 | 2018 | 7400 | Al |
| Refinery Area | | 8 | 2018 | 4940 | Fe |
| Refinery Area | | 8 | 2018 | 84 | Mn |
| Refinery Area | | 8 | 2017 | 4250 | Al |
| Refinery Area | | 8 | 2017 | 4830 | Fe |
| Refinery Area | | 8 | 2017 | 95.5 | Mn |
| Refinery Area | | 8 | 2016 | 6990 | Al |
| Refinery Area | | 8 | 2016 | 7020 | Fe |
| Refinery Area | | 8 | 2016 | 90.7 | Mn |
| Refinery Area | | 8 | 2015 | 7360 | Al |
| Refinery Area | | 8 | 2015 | 8030 | Fe |
| Refinery Area | | 8 | 2015 | 96 | Mn |
| Refinery Area | | 8 | 2014 | 7900 | Al |
| Refinery Area | | 8 | 2014 | 5540 | Fe |
| Refinery Area | | 8 | 2014 | 93.7 | Mn |
| Refinery Area | | 8 | 2013 | 7160 | Al |
| Refinery Area | | 8 | 2013 | 7230 | Fe |
| Refinery Area | | 8 | 2013 | 90.4 | Mn |
| Refinery Area | | 8 | 2012 | 8530 | Al |
| Refinery Area | | 8 | 2012 | 9160 | Fe |
| Refinery Area | | 8 | 2012 | 119 | Mn |
| Refinery Area | | 8 | 2011 | 10400 | Al |
| Refinery Area | | 8 | 2011 | 6880 | Fe |
| Refinery Area | | 8 | 2011 | 78.1 | Mn |
| Refinery Area | | 8 | 2010 | 10200 | Al |
| Refinery Area | | 8 | 2010 | 5970 | Fe |
| Refinery Area | | 8 | 2010 | 98 | Mn |
| Refinery Area | | 8 | 2009 | 16300 | Al |
| Refinery Area | | 8 | 2009 | 5200 | Fe |
| Refinery Area | | 8 | 2009 | 79.5 | Mn |
| Refinery Area | | 8 | 2008 | 85.3 | Mn |
| Refinery Area | | 8 | 2007 | 99.1 | Mn |
| Gaha | | 9 | 2016 | 3560 | Al |
| Gaha | | 9 | 2016 | 8110 | Fe |
| Gaha | | 9 | 2016 | 37.9 | Mn |
| Gaha | | 9 | 2015 | 3380 | Al |
| Gaha | | 9 | 2015 | 11500 | Fe |
| Gaha | | 9 | 2015 | 60.9 | Mn |
| Gaha | | 9 | 2014 | 3050 | Al |
| Gaha | | 9 | 2014 | 6500 | Fe |
| Gaha | | 9 | 2014 | 79 | Mn |
| Gaha | | 9 | 2013 | 3200 | Al |
| Gaha | | 9 | 2013 | 10300 | Fe |
| Gaha | | 9 | 2013 | 44.4 | Mn |
| Gaha | | 9 | 2012 | 3480 | Al |
| Gaha | | 9 | 2012 | 12500 | Fe |
| Gaha | | 9 | 2012 | 48.8 | Mn |
| Gaha | | 9 | 2011 | 4780 | Al |
| Gaha | | 9 | 2011 | 11000 | Fe |
| Gaha | | 9 | 2011 | 32.7 | Mn |
| Gaha | | 9 | 2010 | 3490 | Al |
| Gaha | | 9 | 2010 | 4300 | Fe |
| Gaha | | 9 | 2010 | 44.8 | Mn |
| Gaha | | 9 | 2009 | 4410 | Al |
| Gaha | | 9 | 2009 | 9910 | Fe |
| Gaha | | 9 | 2009 | 36.9 | Mn |
| Gaha | | 9 | 2007 | 12.4 | Mn |
| Suhain | | 10 | 2016 | 2510 | Al |
| Suhain | | 10 | 2016 | 7850 | Fe |
| Suhain | | 10 | 2016 | 45.9 | Mn |
| Suhain | | 10 | 2015 | 1600 | Al |
| Suhain | | 10 | 2015 | 1460 | Fe |
| Suhain | | 10 | 2015 | 23.8 | Mn |
| Suhain | | 10 | 2014 | 4690 | Al |
| Suhain | | 10 | 2014 | 10600 | Fe |
| Suhain | | 10 | 2014 | 59.1 | Mn |
| Suhain | | 10 | 2013 | 3660 | Al |
| Suhain | | 10 | 2013 | 10400 | Fe |
| Suhain | | 10 | 2013 | 58.8 | Mn |
| Suhain | | 10 | 2012 | 2760 | Al |
| Suhain | | 10 | 2012 | 10400 | Fe |
| Suhain | | 10 | 2012 | 29.8 | Mn |
| Suhain | | 10 | 2011 | 5020 | Al |
| Suhain | | 10 | 2011 | 9740 | Fe |
| Suhain | | 10 | 2011 | 37.8 | Mn |
| Suhain | | 10 | 2010 | 6440 | Al |
| Suhain | | 10 | 2010 | 6870 | Fe |
| Suhain | | 10 | 2010 | 65 | Mn |
| Suhain | | 10 | 2009 | 8190 | Al |
| Suhain | | 10 | 2009 | 24100 | Fe |
| Suhain | | 10 | 2009 | 58 | Mn |
| Suhain | | 10 | 2008 | 13.6 | Mn |
| Suhain | | 10 | 2007 | 14.5 | Mn |
| Duwaimil | | 11 | 2015 | 3930 | Al |
| Duwaimil | | 11 | 2015 | 8680 | Fe |
| Duwaimil | | 11 | 2015 | 74.1 | Mn |
| Duwaimil | | 11 | 2014 | 4280 | Al |
| Duwaimil | | 11 | 2014 | 5400 | Fe |
| Duwaimil | | 11 | 2014 | 97.4 | Mn |
| Duwaimil | | 11 | 2013 | 3220 | Al |
| Duwaimil | | 11 | 2013 | 7350 | Fe |
| Duwaimil | | 11 | 2013 | 59.5 | Mn |
| Gazara | | 12 | 2020 | 6200 | Al |
| Gazara | | 12 | 2020 | 5620 | Fe |
| Gazara | | 12 | 2020 | 103 | Mn |
| Gazara | | 12 | 2019 | 7000 | Al |
| Gazara | | 12 | 2019 | 5540 | Fe |
| Gazara | | 12 | 2019 | 98.7 | Mn |
| Gazara | | 12 | 2018 | 7410 | Al |
| Gazara | | 12 | 2018 | 5750 | Fe |
| Gazara | | 12 | 2018 | 99.1 | Mn |
| Gazara | | 12 | 2017 | 5790 | Al |
| Gazara | | 12 | 2017 | 6230 | Fe |
| Gazara | | 12 | 2017 | 112 | Mn |
| Gazara | | 12 | 2016 | 8270 | Al |
| Gazara | | 12 | 2016 | 6860 | Fe |
| Gazara | | 12 | 2016 | 98.5 | Mn |
| Gazara | | 12 | 2015 | 7030 | Al |
| Gazara | | 12 | 2015 | 7990 | Fe |
| Gazara | | 12 | 2015 | 105 | Mn |
| Gazara | | 12 | 2014 | 6700 | Al |
| Gazara | | 12 | 2014 | 5770 | Fe |
| Gazara | | 12 | 2014 | 51 | Mn |
| Gazara | | 12 | 2013 | 5440 | Al |
| Gazara | | 12 | 2013 | 7460 | Fe |
| Gazara | | 12 | 2013 | 99.2 | Mn |
| Gazara | | 12 | 2012 | 4120 | Al |
| Gazara | | 12 | 2012 | 5580 | Fe |
| Gazara | | 12 | 2012 | 68.7 | Mn |
| Gazara | | 12 | 2011 | 8770 | Al |
| Gazara | | 12 | 2011 | 5110 | Fe |
| Gazara | | 12 | 2011 | 75.8 | Mn |
| Gazara | | 12 | 2010 | 9390 | Al |
| Gazara | | 12 | 2010 | 3320 | Fe |
| Gazara | | 12 | 2010 | 105 | Mn |
| Gazara | | 12 | 2009 | 12000 | Al |
| Gazara | | 12 | 2009 | 6160 | Fe |
| Gazara | | 12 | 2009 | 77.9 | Mn |
| Dam | | 13 | 2016 | 6470 | Al |
| Dam | | 13 | 2016 | 4600 | Fe |
| Dam | | 13 | 2016 | 80.8 | Mn |
| Dam | | 13 | 2015 | 5960 | Al |
| Dam | | 13 | 2015 | 5940 | Fe |
| Dam | | 13 | 2015 | 97.5 | Mn |
| Dam | | 13 | 2014 | 5080 | Al |
| Dam | | 13 | 2014 | 3410 | Fe |
| Dam | | 13 | 2014 | 109 | Mn |
| Dam | | 13 | 2013 | 3240 | Al |
| Dam | | 13 | 2013 | 2640 | Fe |
| Dam | | 13 | 2013 | 50.1 | Mn |
| Dam | | 13 | 2011 | 6540 | Al |
| Dam | | 13 | 2011 | 3380 | Fe |
| Dam | | 13 | 2011 | 57.2 | Mn |
| Dam | | 13 | 2010 | 4970 | Al |
| Dam | | 13 | 2010 | 1530 | Fe |
| Dam | | 13 | 2010 | 67.2 | Mn |
| Dam | | 13 | 2009 | 11800 | Al |
| Dam | | 13 | 2009 | 4320 | Fe |
| Dam | | 13 | 2009 | 73.8 | Mn |
| Jaradah | | 14 | 2020 | 3400 | Al |
| Jaradah | | 14 | 2020 | 2810 | Fe |
| Jaradah | | 14 | 2020 | 55.3 | Mn |
| Jaradah | | 14 | 2019 | 3750 | Al |
| Jaradah | | 14 | 2019 | 2920 | Fe |
| Jaradah | | 14 | 2019 | 51.8 | Mn |
| Jaradah | | 14 | 2018 | 5330 | Al |
| Jaradah | | 14 | 2018 | 3660 | Fe |
| Jaradah | | 14 | 2018 | 65.1 | Mn |
| Jaradah | | 14 | 2017 | 2960 | Al |
| Jaradah | | 14 | 2017 | 3030 | Fe |
| Jaradah | | 14 | 2017 | 62.2 | Mn |
| Jaradah | | 14 | 2016 | 4900 | Al |
| Jaradah | | 14 | 2016 | 3520 | Fe |
| Jaradah | | 14 | 2016 | 51 | Mn |
| Jaradah | | 14 | 2015 | 4100 | Al |
| Jaradah | | 14 | 2015 | 3800 | Fe |
| Jaradah | | 14 | 2015 | 61.2 | Mn |
| Jaradah | | 14 | 2014 | 4070 | Al |
| Jaradah | | 14 | 2014 | 2660 | Fe |
| Jaradah | | 14 | 2014 | 60.5 | Mn |
| Jaradah | | 14 | 2013 | 2170 | Al |
| Jaradah | | 14 | 2013 | 1790 | Fe |
| Jaradah | | 14 | 2013 | 33.4 | Mn |
| Jaradah | | 14 | 2012 | 4590 | Al |
| Jaradah | | 14 | 2012 | 4020 | Fe |
| Jaradah | | 14 | 2012 | 65.2 | Mn |
| Jaradah | | 14 | 2011 | 4820 | Al |
| Jaradah | | 14 | 2011 | 3120 | Fe |
| Jaradah | | 14 | 2011 | 41.7 | Mn |
| Jaradah | | 14 | 2010 | 5920 | Al |
| Jaradah | | 14 | 2010 | 1380 | Fe |
| Jaradah | | 14 | 2010 | 58.3 | Mn |
| Jaradah | | 14 | 2009 | 7200 | Al |
| Jaradah | | 14 | 2009 | 3070 | Fe |
| Jaradah | | 14 | 2009 | 46.6 | Mn |
| Jaradah | | 14 | 2008 | 63 | Mn |
| Jaradah | | 14 | 2007 | 84.7 | Mn |
| Jetty 1 | | 15 | 2014 | 8650 | Al |
| Jetty 1 | | 15 | 2014 | 6090 | Fe |
| Jetty 1 | | 15 | 2014 | 75.2 | Mn |
| Jetty 1 | | 15 | 2013 | 7360 | Al |
| Jetty 1 | | 15 | 2013 | 7580 | Fe |
| Jetty 1 | | 15 | 2013 | 109 | Mn |
| Jetty 1 | | 15 | 2012 | 6590 | Al |
| Jetty 1 | | 15 | 2012 | 5630 | Fe |
| Jetty 1 | | 15 | 2012 | 80.3 | Mn |
| Jetty 1 | | 15 | 2011 | 6530 | Al |
| Jetty 1 | | 15 | 2011 | 5050 | Fe |
| Jetty 1 | | 15 | 2011 | 67.1 | Mn |
| Jetty 1 | | 15 | 2010 | 11600 | Al |
| Jetty 1 | | 15 | 2010 | 11800 | Fe |
| Jetty 1 | | 15 | 2010 | 155 | Mn |
| Jetty 1 | | 15 | 2009 | 18800 | Al |
| Jetty 1 | | 15 | 2009 | 6570 | Fe |
| Jetty 1 | | 15 | 2009 | 93.9 | Mn |
| Jetty 1 | | 15 | 2008 | 122 | Mn |
| Jetty 1 | | 15 | 2007 | 125 | Mn |
| Al-Jarim | | 16 | 2020 | 4050 | Al |
| Al-Jarim | | 16 | 2020 | 3470 | Fe |
| Al-Jarim | | 16 | 2020 | 77.2 | Mn |
| Al-Jarim | | 16 | 2019 | 5080 | Al |
| Al-Jarim | | 16 | 2019 | 4030 | Fe |
| Al-Jarim | | 16 | 2019 | 80.3 | Mn |
| Al-Jarim | | 16 | 2018 | 5250 | Al |
| Al-Jarim | | 16 | 2018 | 3090 | Fe |
| Al-Jarim | | 16 | 2018 | 68.2 | Mn |
| Al-Jarim | | 16 | 2017 | 4180 | Al |
| Al-Jarim | | 16 | 2017 | 4060 | Fe |
| Al-Jarim | | 16 | 2017 | 94.3 | Mn |
| Al-Jarim | | 16 | 2016 | 4470 | Al |
| Al-Jarim | | 16 | 2016 | 3900 | Fe |
| Al-Jarim | | 16 | 2016 | 77.3 | Mn |
| Al-Jarim | | 16 | 2015 | 6600 | Al |
| Al-Jarim | | 16 | 2015 | 5270 | Fe |
| Al-Jarim | | 16 | 2015 | 91.6 | Mn |
| Al-Jarim | | 16 | 2014 | 5240 | Al |
| Al-Jarim | | 16 | 2014 | 3370 | Fe |
| Al-Jarim | | 16 | 2014 | 29.7 | Mn |
| Al-Jarim | | 16 | 2013 | 4100 | Al |
| Al-Jarim | | 16 | 2013 | 3800 | Fe |
| Al-Jarim | | 16 | 2013 | 79.2 | Mn |
| Al-Jarim | | 16 | 2012 | 8360 | Al |
| Al-Jarim | | 16 | 2012 | 4390 | Fe |
| Al-Jarim | | 16 | 2012 | 87.8 | Mn |
| Al-Jarim | | 16 | 2011 | 8930 | Al |
| Al-Jarim | | 16 | 2011 | 4240 | Fe |
| Al-Jarim | | 16 | 2011 | 76.2 | Mn |
| Al-Jarim | | 16 | 2010 | 5070 | Al |
| Al-Jarim | | 16 | 2010 | 2310 | Fe |
| Al-Jarim | | 16 | 2010 | 45.6 | Mn |
| Al-Jarim | | 16 | 2009 | 4690 | Al |
| Al-Jarim | | 16 | 2009 | 2120 | Fe |
| Al-Jarim | | 16 | 2009 | 33.8 | Mn |
| Al-Jarim | | 16 | 2008 | 57.8 | Mn |
| Al-Jarim | | 16 | 2007 | 8.1 | Mn |
| Khorfasht | | 17 | 2016 | 2470 | Al |
| Khorfasht | | 17 | 2016 | 1800 | Fe |
| Khorfasht | | 17 | 2016 | 53.2 | Mn |
| Khorfasht | | 17 | 2015 | 2370 | Al |
| Khorfasht | | 17 | 2015 | 1530 | Fe |
| Khorfasht | | 17 | 2015 | 51.9 | Mn |
| Khorfasht | | 17 | 2014 | 1490 | Al |
| Khorfasht | | 17 | 2014 | 873 | Fe |
| Khorfasht | | 17 | 2014 | 60.3 | Mn |
| Khorfasht | | 17 | 2013 | 2310 | Al |
| Khorfasht | | 17 | 2013 | 2460 | Fe |
| Khorfasht | | 17 | 2013 | 50.5 | Mn |
| Khorfasht | | 17 | 2012 | 1500 | Al |
| Khorfasht | | 17 | 2012 | 923 | Fe |
| Khorfasht | | 17 | 2012 | 24 | Mn |
| Khorfasht | | 17 | 2011 | 5400 | Al |
| Khorfasht | | 17 | 2011 | 2650 | Fe |
| Khorfasht | | 17 | 2011 | 49.1 | Mn |
| Khorfasht | | 17 | 2010 | 4710 | Al |
| Khorfasht | | 17 | 2010 | 2720 | Fe |
| Khorfasht | | 17 | 2010 | 64.5 | Mn |
| Khorfasht | | 17 | 2009 | 4760 | Al |
| Khorfasht | | 17 | 2009 | 2220 | Fe |
| Khorfasht | | 17 | 2009 | 56.6 | Mn |
| Khorfasht | | 17 | 2008 | 204 | Mn |
| Khorfasht | | 17 | 2007 | 107 | Mn |
| Murwada | | 18 | 2020 | 784 | Al |
| Murwada | | 18 | 2020 | 1040 | Fe |
| Murwada | | 18 | 2020 | 17.9 | Mn |
| Murwada | | 18 | 2019 | 542 | Al |
| Murwada | | 18 | 2019 | 1030 | Fe |
| Murwada | | 18 | 2019 | 13.5 | Mn |
| Murwada | | 18 | 2018 | 1110 | Al |
| Murwada | | 18 | 2018 | 953 | Fe |
| Murwada | | 18 | 2018 | 26.2 | Mn |
| Murwada | | 18 | 2017 | 853 | Al |
| Murwada | | 18 | 2017 | 1040 | Fe |
| Murwada | | 18 | 2017 | 29.8 | Mn |
| Murwada | | 18 | 2016 | 2900 | Al |
| Murwada | | 18 | 2016 | 1760 | Fe |
| Murwada | | 18 | 2016 | 46.4 | Mn |
| Murwada | | 18 | 2015 | 1880 | Al |
| Murwada | | 18 | 2015 | 1650 | Fe |
| Murwada | | 18 | 2015 | 41.9 | Mn |
| Murwada | | 18 | 2014 | 1990 | Al |
| Murwada | | 18 | 2014 | 1880 | Fe |
| Murwada | | 18 | 2014 | 60.3 | Mn |
| Murwada | | 18 | 2013 | 811 | Al |
| Murwada | | 18 | 2013 | 847 | Fe |
| Murwada | | 18 | 2013 | 20.7 | Mn |
| Murwada | | 18 | 2012 | 1930 | Al |
| Murwada | | 18 | 2012 | 2260 | Fe |
| Murwada | | 18 | 2012 | 26.7 | Mn |
| Murwada | | 18 | 2011 | 876 | Al |
| Murwada | | 18 | 2011 | 777 | Fe |
| Murwada | | 18 | 2011 | 19.7 | Mn |
| Murwada | | 18 | 2010 | 557 | Al |
| Murwada | | 18 | 2010 | 372 | Fe |
| Murwada | | 18 | 2010 | 15.8 | Mn |
| Murwada | | 18 | 2009 | 3940 | Al |
| Murwada | | 18 | 2009 | 1860 | Fe |
| Murwada | | 18 | 2009 | 38.3 | Mn |
| Murwada | | 18 | 2008 | 4.2 | Mn |
| Murwada | | 18 | 2007 | 16.9 | Mn |
| Bartafi | | 19 | 2016 | 2390 | Al |
| Bartafi | | 19 | 2016 | 1810 | Fe |
| Bartafi | | 19 | 2016 | 45.2 | Mn |
| Bartafi | | 19 | 2015 | 2520 | Al |
| Bartafi | | 19 | 2015 | 2070 | Fe |
| Bartafi | | 19 | 2015 | 72.9 | Mn |
| Bartafi | | 19 | 2014 | 4720 | Al |
| Bartafi | | 19 | 2014 | 4600 | Fe |
| Bartafi | | 19 | 2014 | 211 | Mn |
| Bartafi | | 19 | 2013 | 2310 | Al |
| Bartafi | | 19 | 2013 | 3260 | Fe |
| Bartafi | | 19 | 2013 | 145 | Mn |
| Bartafi | | 19 | 2012 | 3350 | Al |
| Bartafi | | 19 | 2012 | 1690 | Fe |
| Bartafi | | 19 | 2012 | 35.6 | Mn |
| Bartafi | | 19 | 2011 | 4310 | Al |
| Bartafi | | 19 | 2011 | 2300 | Fe |
| Bartafi | | 19 | 2011 | 69.9 | Mn |
| Bartafi | | 19 | 2010 | 5750 | Al |
| Bartafi | | 19 | 2010 | 3370 | Fe |
| Bartafi | | 19 | 2010 | 89.6 | Mn |
| Bartafi | | 19 | 2009 | 14100 | Al |
| Bartafi | | 19 | 2009 | 2360 | Fe |
| Bartafi | | 19 | 2009 | 68 | Mn |
| Bartafi | | 19 | 2008 | 31.3 | Mn |
| Bartafi | | 19 | 2007 | 183 | Mn |
| Gasar | | 20 | 2016 | 8940 | Al |
| Gasar | | 20 | 2016 | 6390 | Fe |
| Gasar | | 20 | 2016 | 95.4 | Mn |
| Gasar | | 20 | 2015 | 8800 | Al |
| Gasar | | 20 | 2015 | 6350 | Fe |
| Gasar | | 20 | 2015 | 107 | Mn |
| Gasar | | 20 | 2014 | 9760 | Al |
| Gasar | | 20 | 2014 | 5270 | Fe |
| Gasar | | 20 | 2014 | 107 | Mn |
| Gasar | | 20 | 2013 | 6390 | Al |
| Gasar | | 20 | 2013 | 4820 | Fe |
| Gasar | | 20 | 2013 | 109 | Mn |
| Gasar | | 20 | 2012 | 8790 | Al |
| Gasar | | 20 | 2012 | 5520 | Fe |
| Gasar | | 20 | 2012 | 93.8 | Mn |
| Gasar | | 20 | 2011 | 8980 | Al |
| Gasar | | 20 | 2011 | 6170 | Fe |
| Gasar | | 20 | 2011 | 83.9 | Mn |
| Gasar | | 20 | 2010 | 8750 | Al |
| Gasar | | 20 | 2010 | 4970 | Fe |
| Gasar | | 20 | 2010 | 89.1 | Mn |
| Gasar | | 20 | 2009 | 20400 | Al |
| Gasar | | 20 | 2009 | 5810 | Fe |
| Gasar | | 20 | 2009 | 94.4 | Mn |
| Gasar | | 20 | 2008 | 95.9 | Mn |
| Gasar | | 20 | 2007 | 35.9 | Mn |
| Umm Al-Na'asan | | 21 | 2020 | 9660 | Al |
| Umm Al-Na'asan | | 21 | 2020 | 7610 | Fe |
| Umm Al-Na'asan | | 21 | 2020 | 167 | Mn |
| Umm Al-Na'asan | | 21 | 2019 | 9510 | Al |
| Umm Al-Na'asan | | 21 | 2019 | 6530 | Fe |
| Umm Al-Na'asan | | 21 | 2019 | 151 | Mn |
| Umm Al-Na'asan | | 21 | 2018 | 10900 | Al |
| Umm Al-Na'asan | | 21 | 2018 | 5450 | Fe |
| Umm Al-Na'asan | | 21 | 2018 | 133 | Mn |
| Umm Al-Na'asan | | 21 | 2017 | 8330 | Al |
| Umm Al-Na'asan | | 21 | 2017 | 7180 | Fe |
| Umm Al-Na'asan | | 21 | 2017 | 169 | Mn |
| Umm Al-Na'asan | | 21 | 2016 | 10800 | Al |
| Umm Al-Na'asan | | 21 | 2016 | 7800 | Fe |
| Umm Al-Na'asan | | 21 | 2016 | 136 | Mn |
| Umm Al-Na'asan | | 21 | 2015 | 10700 | Al |
| Umm Al-Na'asan | | 21 | 2015 | 7830 | Fe |
| Umm Al-Na'asan | | 21 | 2015 | 147 | Mn |
| Umm Al-Na'asan | | 21 | 2014 | 9920 | Al |
| Umm Al-Na'asan | | 21 | 2014 | 5750 | Fe |
| Umm Al-Na'asan | | 21 | 2014 | 118 | Mn |
| Umm Al-Na'asan | | 21 | 2013 | 7510 | Al |
| Umm Al-Na'asan | | 21 | 2013 | 5590 | Fe |
| Umm Al-Na'asan | | 21 | 2013 | 147 | Mn |
| Umm Al-Na'asan | | 21 | 2012 | 11600 | Al |
| Umm Al-Na'asan | | 21 | 2012 | 6430 | Fe |
| Umm Al-Na'asan | | 21 | 2012 | 149 | Mn |
| Umm Al-Na'asan | | 21 | 2011 | 10800 | Al |
| Umm Al-Na'asan | | 21 | 2011 | 5740 | Fe |
| Umm Al-Na'asan | | 21 | 2011 | 107 | Mn |
| Umm Al-Na'asan | | 21 | 2010 | 12300 | Al |
| Umm Al-Na'asan | | 21 | 2010 | 6660 | Fe |
| Umm Al-Na'asan | | 21 | 2010 | 115 | Mn |
| Umm Al-Na'asan | | 21 | 2009 | 18600 | Al |
| Umm Al-Na'asan | | 21 | 2009 | 6230 | Fe |
| Umm Al-Na'asan | | 21 | 2009 | 118 | Mn |
| Umm Al-Na'asan | | 21 | 2008 | 156 | Mn |
| Umm Al-Na'asan | | 21 | 2007 | 155 | Mn |
| Ya'suf | | 22 | 2020 | 5440 | Al |
| Ya'suf | | 22 | 2020 | 3860 | Fe |
| Ya'suf | | 22 | 2020 | 89.3 | Mn |
| Ya'suf | | 22 | 2019 | 7470 | Al |
| Ya'suf | | 22 | 2019 | 5000 | Fe |
| Ya'suf | | 22 | 2019 | 102 | Mn |
| Ya'suf | | 22 | 2018 | 9440 | Al |
| Ya'suf | | 22 | 2018 | 3770 | Fe |
| Ya'suf | | 22 | 2018 | 102 | Mn |
| Ya'suf | | 22 | 2017 | 6680 | Al |
| Ya'suf | | 22 | 2017 | 5150 | Fe |
| Ya'suf | | 22 | 2017 | 135 | Mn |
| Ya'suf | | 22 | 2016 | 7110 | Al |
| Ya'suf | | 22 | 2016 | 4790 | Fe |
| Ya'suf | | 22 | 2016 | 78.7 | Mn |
| Ya'suf | | 22 | 2015 | 15700 | Al |
| Ya'suf | | 22 | 2015 | 7210 | Fe |
| Ya'suf | | 22 | 2015 | 176 | Mn |
| Ya'suf | | 22 | 2014 | 7430 | Al |
| Ya'suf | | 22 | 2014 | 3890 | Fe |
| Ya'suf | | 22 | 2014 | 148 | Mn |
| Ya'suf | | 22 | 2013 | 6200 | Al |
| Ya'suf | | 22 | 2013 | 5600 | Fe |
| Ya'suf | | 22 | 2013 | 96.4 | Mn |
| Ya'suf | | 22 | 2012 | 7540 | Al |
| Ya'suf | | 22 | 2012 | 4710 | Fe |
| Ya'suf | | 22 | 2012 | 68.6 | Mn |
| Ya'suf | | 22 | 2011 | 8840 | Al |
| Ya'suf | | 22 | 2011 | 5720 | Fe |
| Ya'suf | | 22 | 2011 | 84 | Mn |
| Ya'suf | | 22 | 2010 | 15100 | Al |
| Ya'suf | | 22 | 2010 | 9560 | Fe |
| Ya'suf | | 22 | 2010 | 112 | Mn |
| Ya'suf | | 22 | 2009 | 12900 | Al |
| Ya'suf | | 22 | 2009 | 4040 | Fe |
| Ya'suf | | 22 | 2009 | 82.4 | Mn |
| Ya'suf | | 22 | 2008 | 290 | Mn |
| Ya'suf | | 22 | 2007 | 69.5 | Mn |
| Al-Jasra | | 23 | 2020 | 6170 | Al |
| Al-Jasra | | 23 | 2020 | 4240 | Fe |
| Al-Jasra | | 23 | 2020 | 106 | Mn |
| Al-Jasra | | 23 | 2019 | 6550 | Al |
| Al-Jasra | | 23 | 2019 | 4770 | Fe |
| Al-Jasra | | 23 | 2019 | 97.4 | Mn |
| Al-Jasra | | 23 | 2018 | 8540 | Al |
| Al-Jasra | | 23 | 2018 | 5030 | Fe |
| Al-Jasra | | 23 | 2018 | 113 | Mn |
| Al-Jasra | | 23 | 2017 | 4870 | Al |
| Al-Jasra | | 23 | 2017 | 3900 | Fe |
| Al-Jasra | | 23 | 2017 | 86.5 | Mn |
| Al-Jasra | | 23 | 2016 | 6290 | Al |
| Al-Jasra | | 23 | 2016 | 4840 | Fe |
| Al-Jasra | | 23 | 2016 | 68.9 | Mn |
| Al-Jasra | | 23 | 2015 | 6980 | Al |
| Al-Jasra | | 23 | 2015 | 4680 | Fe |
| Al-Jasra | | 23 | 2015 | 104 | Mn |
| Al-Jasra | | 23 | 2014 | 6340 | Al |
| Al-Jasra | | 23 | 2014 | 3440 | Fe |
| Al-Jasra | | 23 | 2014 | 91.1 | Mn |
| Al-Jasra | | 23 | 2013 | 5210 | Al |
| Al-Jasra | | 23 | 2013 | 5010 | Fe |
| Al-Jasra | | 23 | 2013 | 83.3 | Mn |
| Al-Jasra | | 23 | 2012 | 7580 | Al |
| Al-Jasra | | 23 | 2012 | 4870 | Fe |
| Al-Jasra | | 23 | 2012 | 82.1 | Mn |
| Al-Jasra | | 23 | 2011 | 13000 | Al |
| Al-Jasra | | 23 | 2011 | 5060 | Fe |
| Al-Jasra | | 23 | 2011 | 77.3 | Mn |
| Al-Jasra | | 23 | 2010 | 6510 | Al |
| Al-Jasra | | 23 | 2010 | 3980 | Fe |
| Al-Jasra | | 23 | 2010 | 70.4 | Mn |
| Al-Jasra | | 23 | 2009 | 9300 | Al |
| Al-Jasra | | 23 | 2009 | 3280 | Fe |
| Al-Jasra | | 23 | 2009 | 82.5 | Mn |
| Al-Jasra | | 23 | 2008 | 89.6 | Mn |
| Al-Jasra | | 23 | 2007 | 121 | Mn |
| Shtaya | | 24 | 2020 | 1840 | Al |
| Shtaya | | 24 | 2020 | 1730 | Fe |
| Shtaya | | 24 | 2020 | 38.2 | Mn |
| Shtaya | | 24 | 2019 | 2410 | Al |
| Shtaya | | 24 | 2019 | 1940 | Fe |
| Shtaya | | 24 | 2019 | 37.4 | Mn |
| Shtaya | | 24 | 2018 | 4600 | Al |
| Shtaya | | 24 | 2018 | 2290 | Fe |
| Shtaya | | 24 | 2018 | 49.5 | Mn |
| Shtaya | | 24 | 2017 | 1460 | Al |
| Shtaya | | 24 | 2017 | 2470 | Fe |
| Shtaya | | 24 | 2017 | 48.5 | Mn |
| Shtaya | | 24 | 2016 | 2600 | Al |
| Shtaya | | 24 | 2016 | 2160 | Fe |
| Shtaya | | 24 | 2016 | 37.8 | Mn |
| Shtaya | | 24 | 2015 | 2570 | Al |
| Shtaya | | 24 | 2015 | 2060 | Fe |
| Shtaya | | 24 | 2015 | 42.7 | Mn |
| Bulthama | | 25 | 2015 | 6920 | Al |
| Bulthama | | 25 | 2015 | 4250 | Fe |
| Bulthama | | 25 | 2015 | 118 | Mn |
| Bulthama | | 25 | 2014 | 6010 | Al |
| Bulthama | | 25 | 2014 | 3600 | Fe |
| Bulthama | | 25 | 2014 | 98.4 | Mn |
| West Jarim | | 26 | 2016 | 4100 | Al |
| West Jarim | | 26 | 2016 | 2850 | Fe |
| West Jarim | | 26 | 2016 | 61.4 | Mn |
| West Jarim | | 26 | 2015 | 4140 | Al |
| West Jarim | | 26 | 2015 | 3660 | Fe |
| West Jarim | | 26 | 2015 | 72.6 | Mn |
| West Jarim | | 26 | 2014 | 3530 | Al |
| West Jarim | | 26 | 2014 | 2140 | Fe |
| West Jarim | | 26 | 2014 | 59 | Mn |
| Jetty 2 | | 27 | 2015 | 7070 | Al |
| Jetty 2 | | 27 | 2015 | 9090 | Fe |
| Jetty 2 | | 27 | 2015 | 126 | Mn |
| Jetty 3 | | 28 | 2020 | 6410 | Al |
| Jetty 3 | | 28 | 2020 | 5960 | Fe |
| Jetty 3 | | 28 | 2020 | 126 | Mn |
| Jetty 3 | | 28 | 2019 | 6550 | Al |
| Jetty 3 | | 28 | 2019 | 5520 | Fe |
| Jetty 3 | | 28 | 2019 | 122 | Mn |
| Jetty 3 | | 28 | 2018 | 7140 | Al |
| Jetty 3 | | 28 | 2018 | 5380 | Fe |
| Jetty 3 | | 28 | 2018 | 108 | Mn |
| Jetty 3 | | 28 | 2017 | 5080 | Al |
| Jetty 3 | | 28 | 2017 | 4760 | Fe |
| Jetty 3 | | 28 | 2017 | 122 | Mn |
| Jetty 3 | | 28 | 2016 | 6710 | Al |
| Jetty 3 | | 28 | 2016 | 3960 | Fe |
| Jetty 3 | | 28 | 2016 | 93.4 | Mn |
| Wharf Area | | 29 | 2020 | 4850 | Al |
| Wharf Area | | 29 | 2020 | 19800 | Fe |
| Wharf Area | | 29 | 2020 | 90.2 | Mn |
| Wharf Area | | 29 | 2019 | 4820 | Al |
| Wharf Area | | 29 | 2019 | 23100 | Fe |
| Wharf Area | | 29 | 2019 | 97.2 | Mn |
| Wharf Area | | 29 | 2018 | 4990 | Al |
| Wharf Area | | 29 | 2018 | 13100 | Fe |
| Wharf Area | | 29 | 2018 | 72.8 | Mn |
| Wharf Area | | 29 | 2017 | 4000 | Al |
| Wharf Area | | 29 | 2017 | 18200 | Fe |
| Wharf Area | | 29 | 2017 | 84.7 | Mn |
