# Supplementary material for: Spatial and temporal analysis of the risks posed by metal contamination in coastal and marine sediments of Bahrain
Source: Environ Monit Assess. 2022 Jan 6;194(2):62. doi: 10.1007/s10661-021-09722-7 (PMC8739313; doi:10.1007/s10661-021-09722-7)
Supplement: Supplementary file 2 — Supplementary file2 (PDF 704 KB) [file 10661_2021_9722_MOESM2_ESM.pdf]

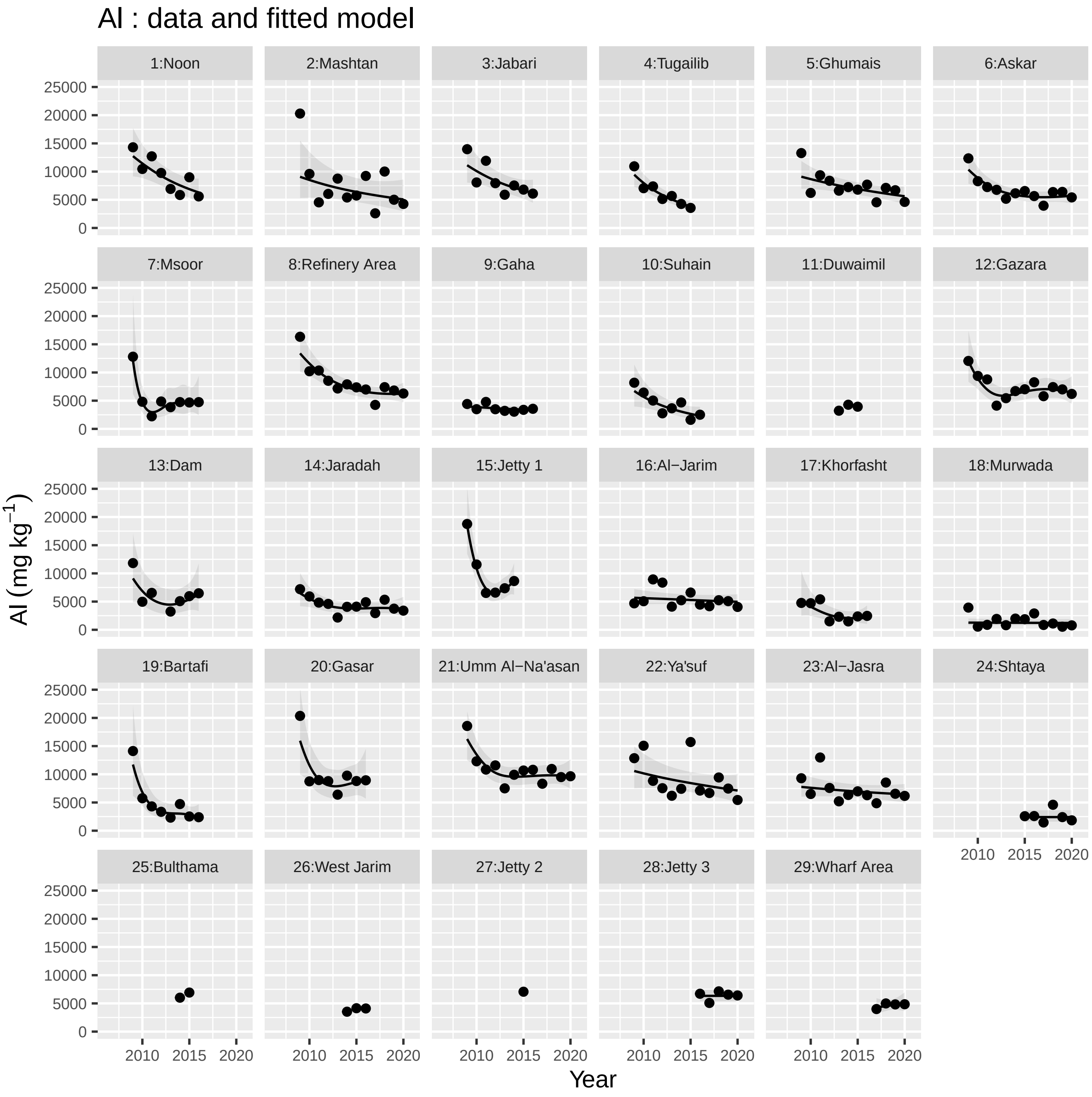
Al : data and fitted model
1:Noon
2:Mashtan
3:Jabari
4:Tugailib
5:Ghumais
6:Askar
7:Msoor
8:Refinery Area
9:Gaha
10:Suhain
11:Duwaimil
12:Gazara
13:Dam
14:Jaradah
15:Jetty 1
16:Al−Jarim
17:Khorfasht
18:Murwada
19:Bartafi
20:Gasar
21:Umm Al−Na'asan
22:Ya'suf
23:Al−Jasra
24:Shtaya
25:Bulthama
26:West Jarim
27:Jetty 2
28:Jetty 3
29:Wharf Area
25000
20000
15000
10000
5000
0
2010
2015
2020
Al (mg kg^{-1})
Year

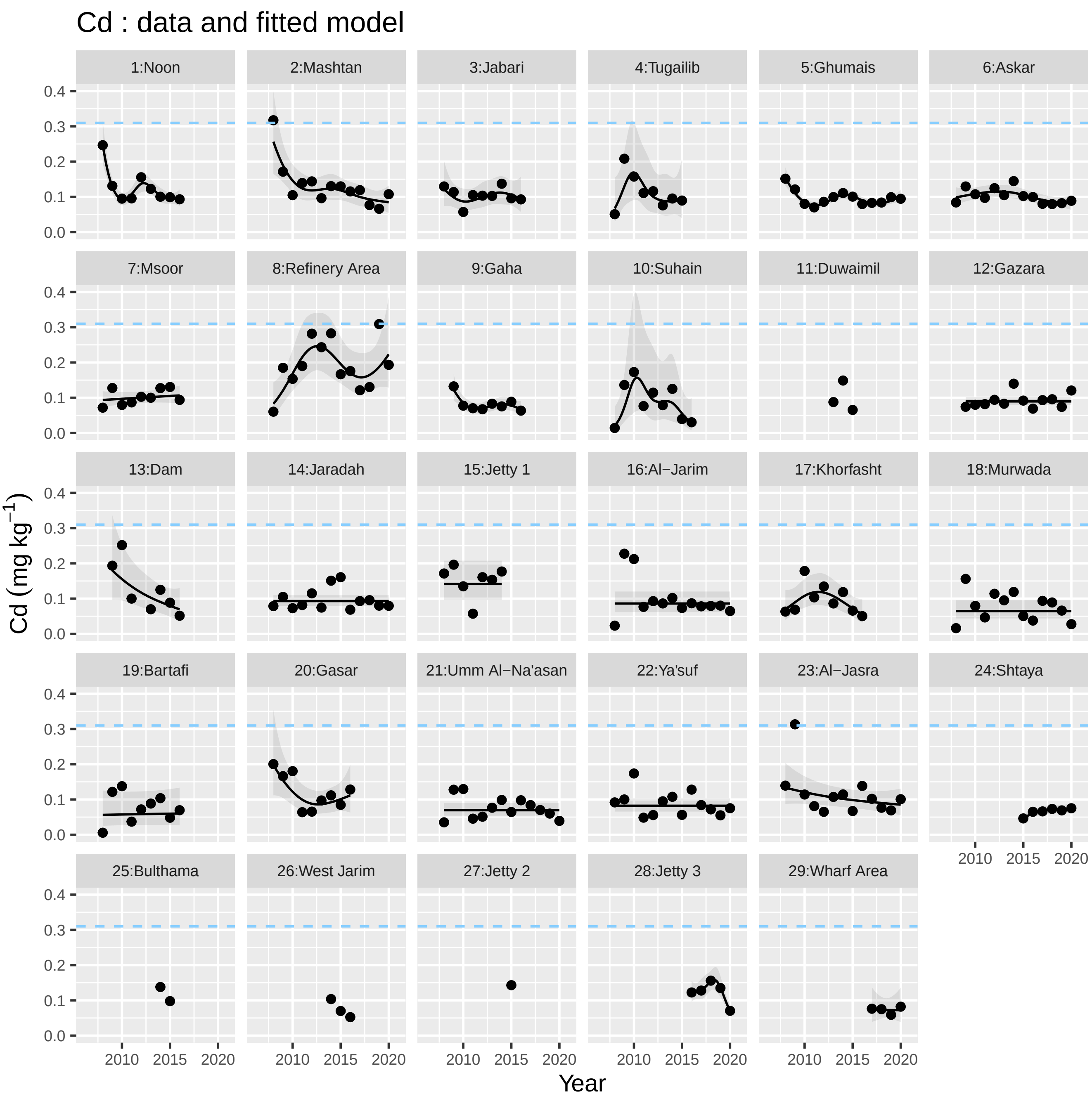

Cd : data and fitted model
1:Noon
2:Mashtan
3:Jabari
4:Tugailib
5:Ghumais
6:Askar
7:Msoor
8:Refinery Area
9:Gaha
10:Suhain
11:Duwaimil
12:Gazara
13:Dam
14:Jaradah
15:Jetty 1
16:Al−Jarim
17:Khorfasht
18:Murwada
19:Bartafi
20:Gasar
21:Umm Al-Na'asan
22:Ya'suf
23:Al−Jasra
24:Shtaya
25:Bulthama
26:West Jarim
27:Jetty 2
28:Jetty 3
29:Wharf Area
0.0
0.1
0.2
0.3
0.4
2010
2015
2020
Cd (mg kg^{-1})
Year

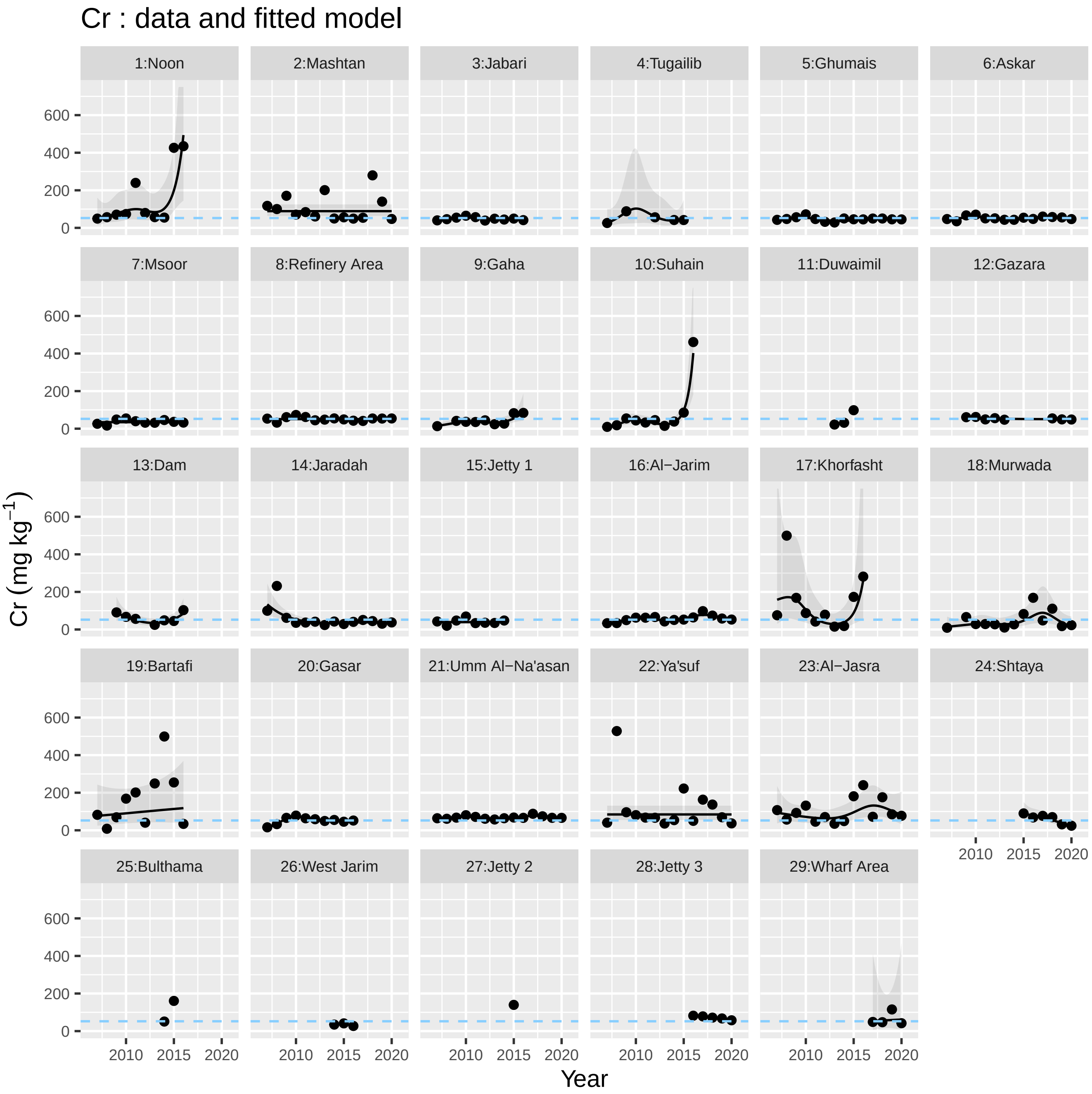
Cr : data and fitted model
1:Noon
2:Mashtan
3:Jabari
4:Tugailib
5:Ghumais
6:Askar
7:Msoor
8:Refinery Area
9:Gaha
10:Suhain
11:Duwaimil
12:Gazara
13:Dam
14:Jaradah
15:Jetty 1
16:Al−Jarim
17:Khorfasht
18:Murwada
19:Bartafi
20:Gasar
21:Umm Al−Na'asan
22:Ya'suf
23:Al−Jasra
24:Shtaya
25:Bulthama
26:West Jarim
27:Jetty 2
28:Jetty 3
29:Wharf Area
0
200
400
600
2010
2015
2020
Cr (mg kg^{-1})
Year

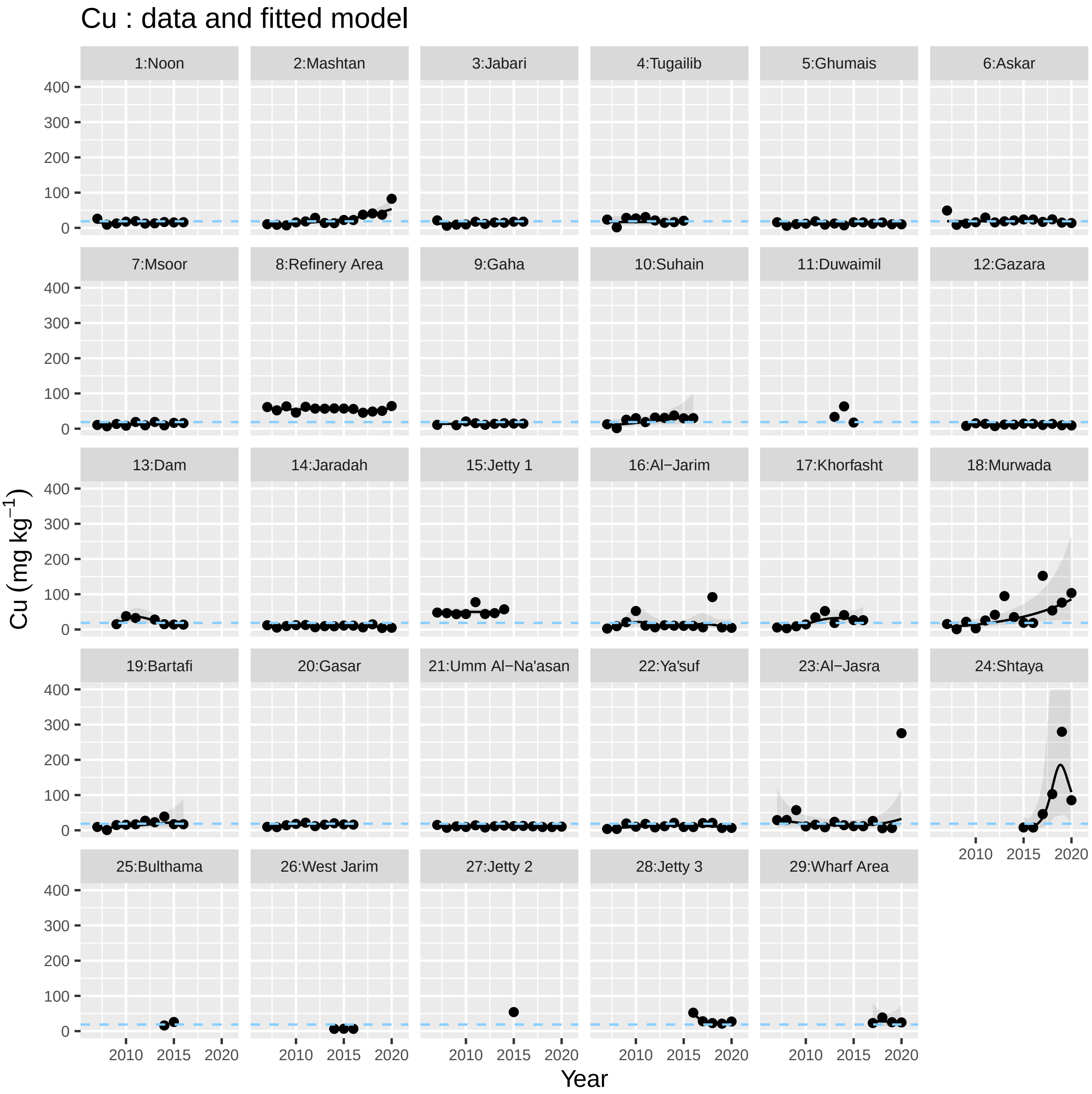
Cu : data and fitted model
1:Noon
2:Mashtan
3:Jabari
4:Tugailib
5:Ghumais
6:Askar
7:Msoor
8:Refinery Area
9:Gaha
10:Suhain
11:Duwaimil
12:Gazara
13:Dam
14:Jaradah
15:Jetty 1
16:Al−Jarim
17:Khorfasht
18:Murwada
19:Bartafi
20:Gasar
21:Umm Al−Na'asan
22:Ya'suf
23:Al−Jasra
24:Shtaya
25:Bulthama
26:West Jarim
27:Jetty 2
28:Jetty 3
29:Wharf Area
Cu (mg kg^{-1})
0
100
200
300
400
2010
2015
2020
Year

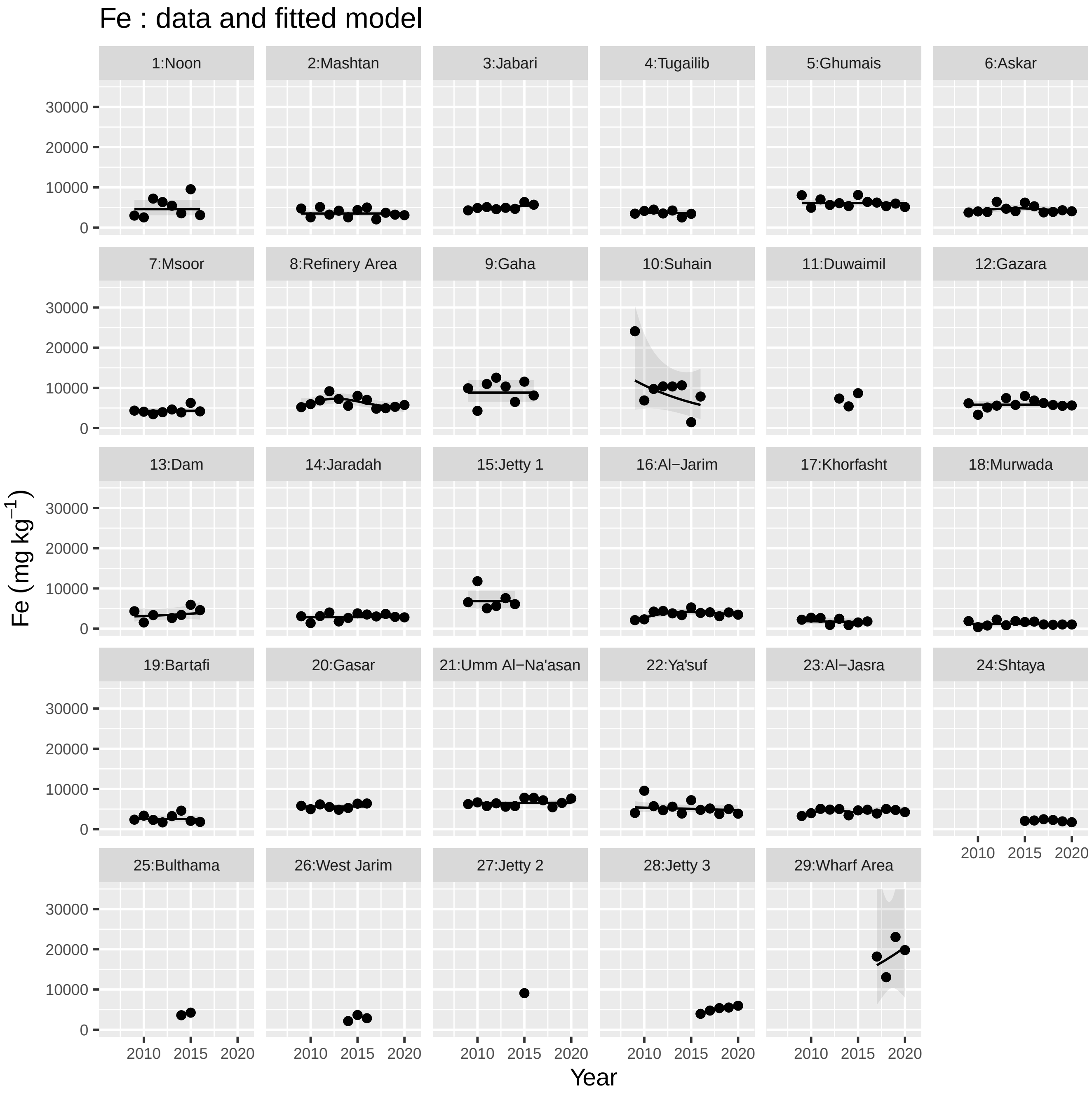

Fe : data and fitted model
1:Noon
2:Mashtan
3:Jabari
4:Tugailib
5:Ghumais
6:Askar
7:Msoor
8:Refinery Area
9:Gaha
10:Suhain
11:Duwaimil
12:Gazara
13:Dam
14:Jaradah
15:Jetty 1
16:Al−Jarim
17:Khorfasht
18:Murwada
19:Bartafi
20:Gasar
21:Umm Al−Na'asan
22:Ya'suf
23:Al−Jasra
24:Shtaya
25:Bulthama
26:West Jarim
27:Jetty 2
28:Jetty 3
29:Wharf Area
Fe (mg kg^{-1})
Year
0
10000
20000
30000
2010
2015
2020

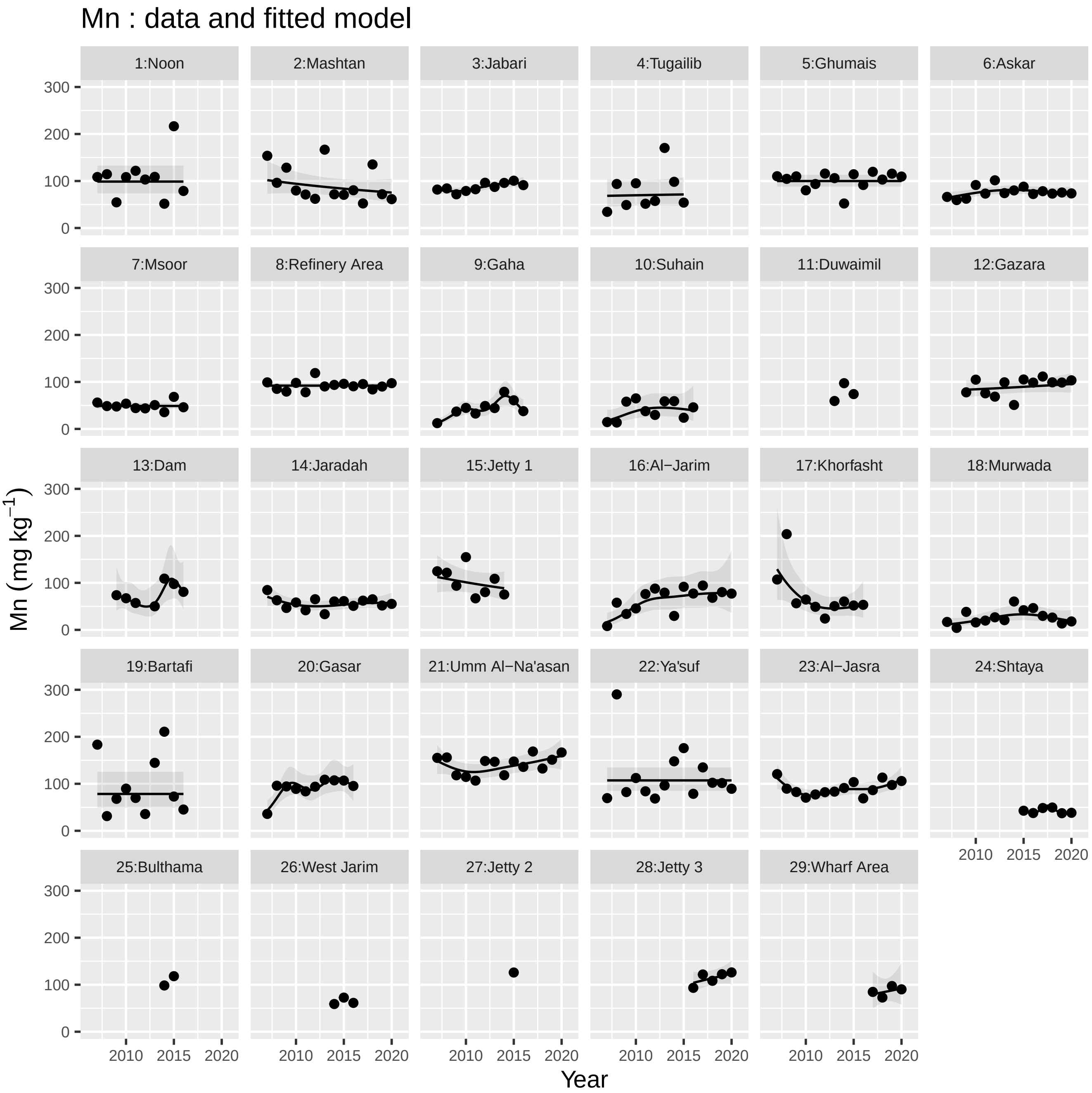
Mn : data and fitted model
1:Noon
2:Mashtan
3:Jabari
4:Tugailib
5:Ghumais
6:Askar
7:Msoor
8:Refinery Area
9:Gaha
10:Suhain
11:Duwaimil
12:Gazara
13:Dam
14:Jaradah
15:Jetty 1
16:Al−Jarim
17:Khorfasht
18:Murwada
19:Bartafi
20:Gasar
21:Umm Al−Na'asan
22:Ya'suf
23:Al−Jasra
24:Shtaya
25:Bulthama
26:West Jarim
27:Jetty 2
28:Jetty 3
29:Wharf Area
Mn (mg kg−1)
0
100
200
300
2010
2015
2020
Year

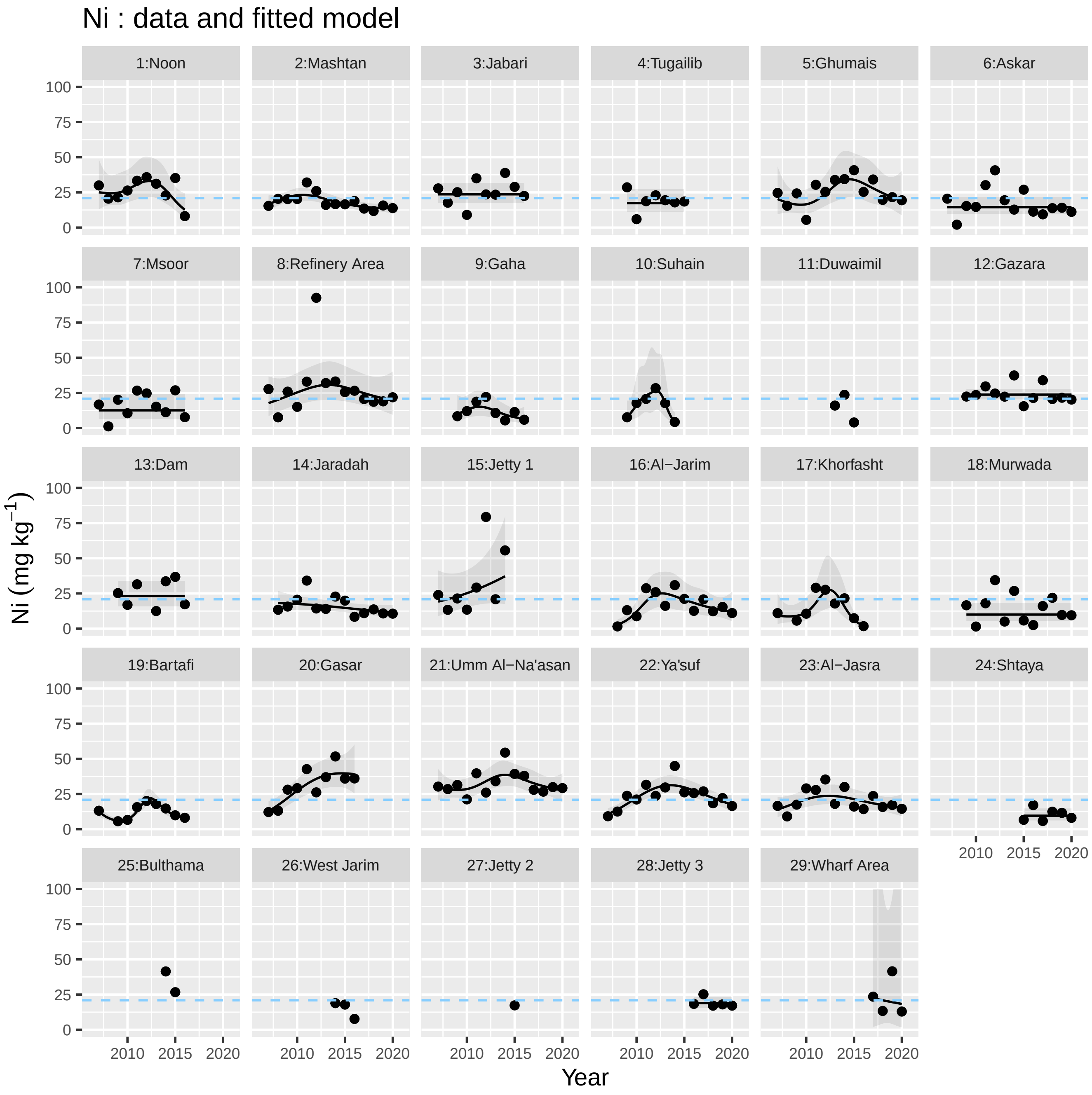

Ni : data and fitted model
1:Noon
2:Mashtan
3:Jabari
4:Tugailib
5:Ghumais
6:Askar
7:Msoor
8:Refinery Area
9:Gaha
10:Suhain
11:Duwaimil
12:Gazara
13:Dam
14:Jaradah
15:Jetty 1
16:Al−Jarim
17:Khorfasht
18:Murwada
19:Bartafi
20:Gasar
21:Umm Al-Na'asan
22:Ya'suf
23:Al−Jasra
24:Shtaya
25:Bulthama
26:West Jarim
27:Jetty 2
28:Jetty 3
29:Wharf Area
0
25
50
75
100
2010
2015
2020
Ni (mg kg^{-1})
Year

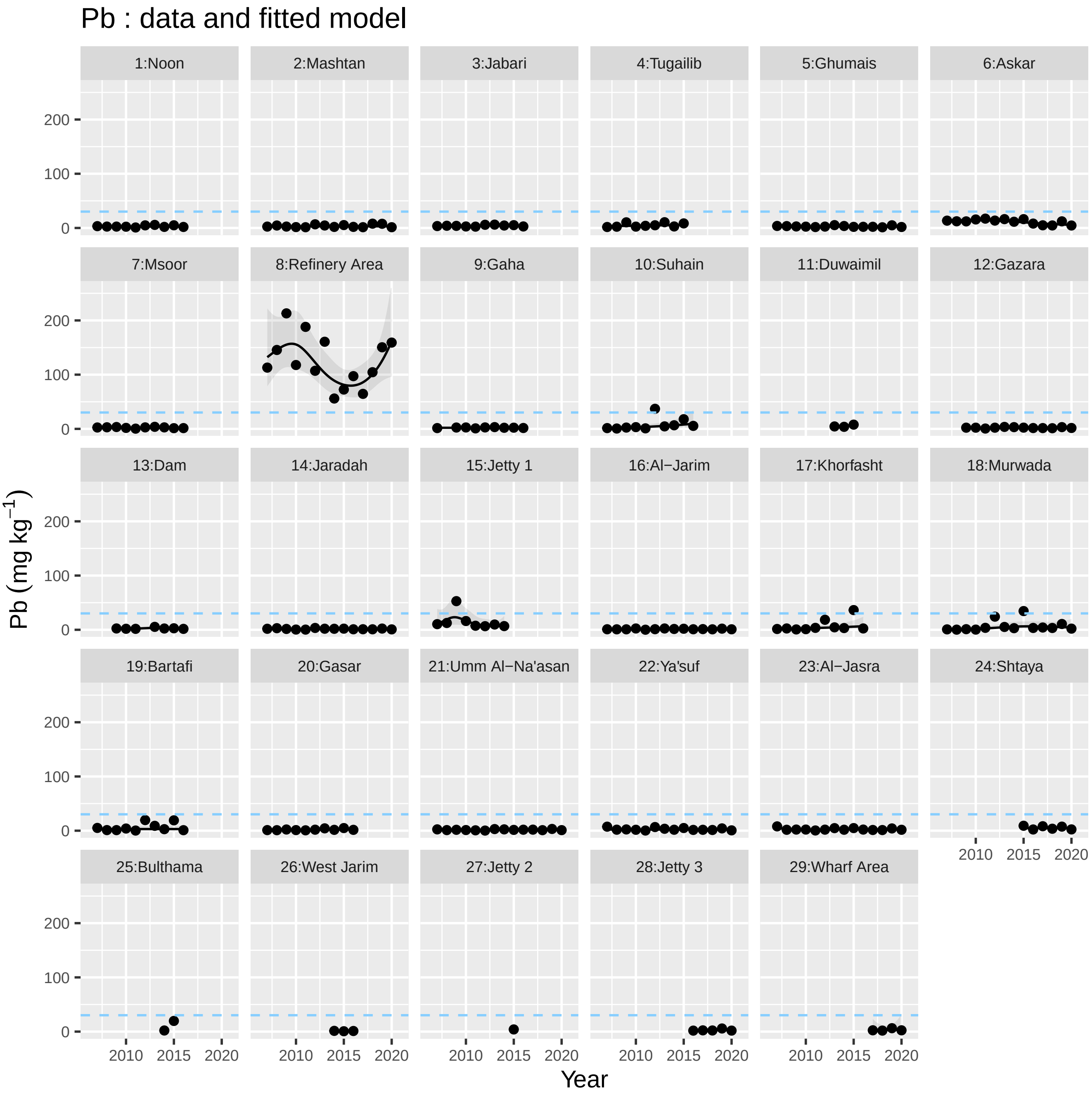

Pb : data and fitted model
1:Noon
2:Mashtan
3:Jabari
4:Tugailib
5:Ghumais
6:Askar
7:Msoor
8:Refinery Area
9:Gaha
10:Suhain
11:Duwaimil
12:Gazara
13:Dam
14:Jaradah
15:Jetty 1
16:Al−Jarim
17:Khorfasht
18:Murwada
19:Bartafi
20:Gasar
21:Umm Al−Na'asan
22:Ya'suf
23:Al−Jasra
24:Shtaya
25:Bulthama
26:West Jarim
27:Jetty 2
28:Jetty 3
29:Wharf Area
0
100
200
2010
2015
2020
Pb (mg kg−1)
Year

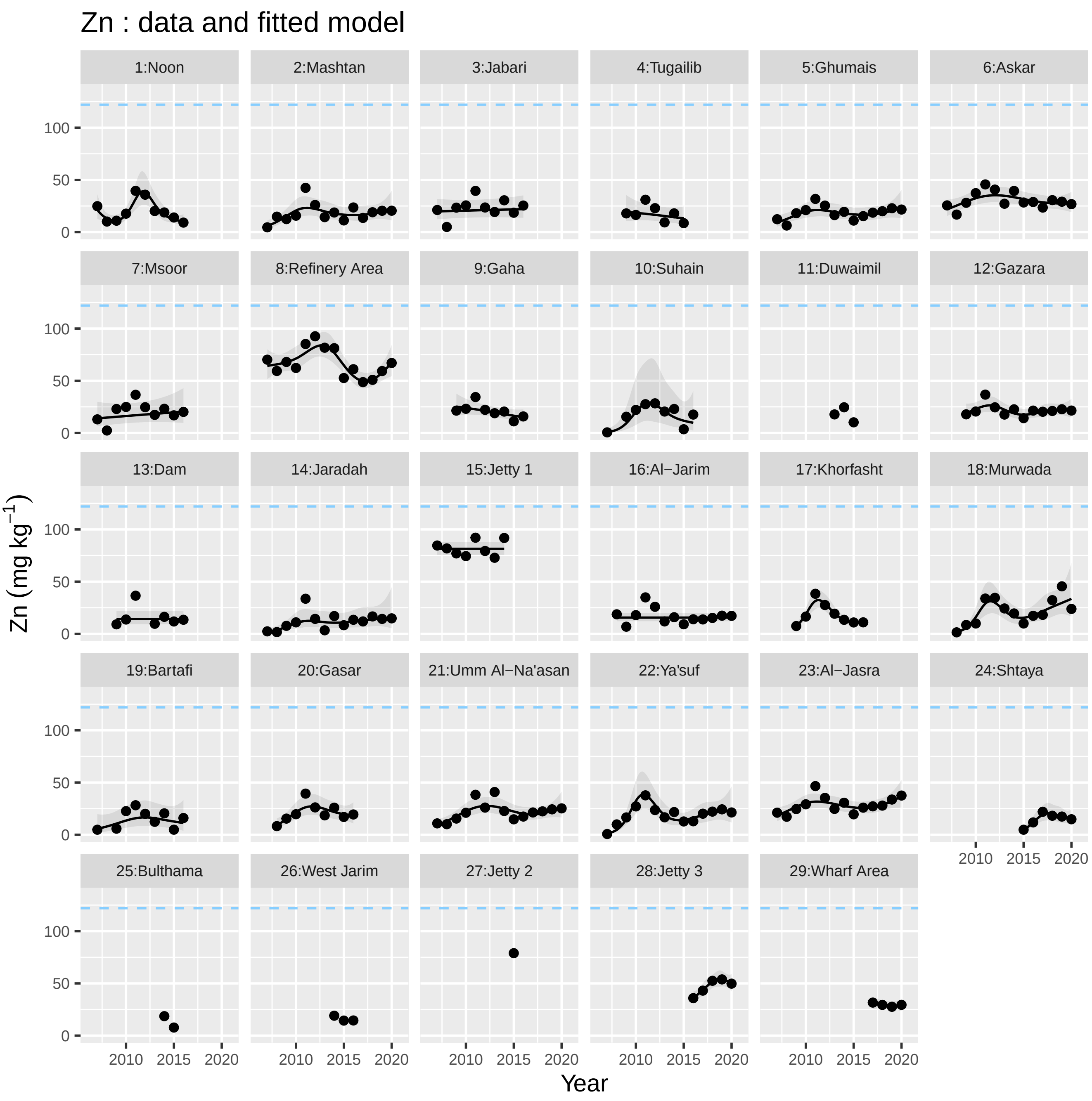
Zn : data and fitted model
1:Noon
2:Mashtan
3:Jabari
4:Tugailib
5:Ghumais
6:Askar
7:Msoor
8:Refinery Area
9:Gaha
10:Suhain
11:Duwaimil
12:Gazara
13:Dam
14:Jaradah
15:Jetty 1
16:Al−Jarim
17:Khorfasht
18:Murwada
19:Bartafi
20:Gasar
21:Umm Al−Na'asan
22:Ya'suf
23:Al−Jasra
24:Shtaya
25:Bulthama
26:West Jarim
27:Jetty 2
28:Jetty 3
29:Wharf Area
0
50
100
2010
2015
2020
Zn (mg kg−1)
Year
